# Fibroblast growth factor receptor (FGFR) alterations in squamous differentiated bladder cancer: a putative therapeutic target for a small subgroup

**DOI:** 10.18632/oncotarget.12198

**Published:** 2016-09-22

**Authors:** Philipp H. Baldia, Angela Maurer, Timon Heide, Michael Rose, Robert Stoehr, Arndt Hartmann, Sarah V. Williams, Margaret A. Knowles, Ruth Knuechel, Nadine T. Gaisa

**Affiliations:** ^1^ Institute of Pathology, RWTH Aachen University, Aachen, Germany; ^2^ Institute of Pathology, University Hospital Erlangen, Friedrich-Alexander University, Erlangen-Nuremberg, Erlangen, Germany; ^3^ Section of Molecular Oncology, Leeds Institute of Cancer and Pathology, University of Leeds, St. James's University Hospital, United Kingdom

**Keywords:** fibroblast growth factor receptor, FGFR1, FGFR2, FGFR3, squamous bladder cancer

## Abstract

Although drugable fibroblast growth factor receptor (FGFR) alterations in squamous cell carcinomas (SCC) of various entities are well known, little is known about FGFR modifications in squamous differentiated bladder cancer. Therefore, our study evaluated FGFR1-3 alterations as a putative therapeutic target in this subgroup. We analyzed 73 squamous differentiated bladder cancers (*n* = 10 pT2, *n* = 55 pT3, *n* = 8 pT4) for FGFR1-3 protein expression, *FGFR1-3* copy number variations, *FGFR3* chromosomal rearrangements (fluorescence *in situ* hybridization (FISH)) and *FGFR3* mutations (SNapShot analysis). Only single cases displayed enhanced protein expression, most frequently FGFR3 overexpression (9.4% (6/64)). FISH showed no amplifications of *FGFR1*, *2* or *3*. Break apart events were only slightly above the cut off in 12.1% (8/66) of cases and no *FGFR3-TACC3* rearrangements could be proven by qPCR. *FGFR3* mutations (p.S249C) were found in 8.5% (6/71) of tumors and were significantly associated with FGFR3 protein overexpression (*p* < 0.001), and unfavourable clinical outcome (*p* = 0.001). Our findings are consistent with the results of the TCGA data set for the “squamous-like” subtype of bladder cancer (*n* = 85), which revealed reduced overall expression of *FGFR1* and *FGFR2* in tumors compared to normal tissue, while expression of *FGFR3* remained high. In the TCGA “squamous-like” subtype *FGFR3* mutations were found in 4.9% and correlated with high *FGFR3* RNA expression. Mutations of *FGFR1* and *FGFR2* were less frequent (2.4% and 1.2%). Hence, our comprehensive study provides novel insights into a subgroup of squamous differentiated bladder tumors that hold clues for novel therapeutic regimens and may benefit from FGFR3-targeted therapies.

## INTRODUCTION

Bladder cancer is histopathologically a heterogeneous group comprising urothelial carcinoma (UC), squamous cell carcinoma (SCC), adenocarcinoma and neuroendocrine tumors (small cell carcinoma). About 90% of bladder cancers in western countries are histologically classified as UC, and SCC comprise < 3% of the tumors [1]. However, recent cluster analysis of whole genome expression data has identified breast cancer-like “basal” and “luminal” types of muscle invasive UC with a distinct “squamous-like” subtype [2–4]. This subtype shows high level expression of high molecular weight keratins (KRT5, KRT6, KRT14), EGFR and an invasive/metastatic phenotype with shorter survival times similar to SCC of the bladder. So far, cystectomy is the main treatment strategy for muscle invasive UC, SCC or mixed UC with squamous differentiation. The value of neoadjuvant chemotherapy remains controversial, as on the one hand there is evidence for superiority in cases with mixed histopathology and on the other hand the consequent delay of cystectomy in squamous differentiated tumors is associated with poor response rates [5].

At the molecular level, UC and squamous carcinomas of other sites (e.g. lung and head and neck) share pathways such as fibroblast growth factor receptor (FGFR)-signaling [6]. This tyrosine kinase receptor family comprises four different FGFRs (FGFR1-4), which control cell survival and differentiation mainly via the Ras/MAPK, STAT and PI3K pathway [7]. In UC, pathway activation results primarily from point mutated *FGFR3*, which is particularly frequent in low grade non-invasive bladder cancers [8]. *FGFR3* mutation and protein overexpression in invasive UC is less frequent (12.6%) [3]. Previous work from our group showed that amplification of *FGFR* genes is rare in UC (1.6% *FGFR1*, 0.8% *FGFR2*, 3.4% *FGFR3*) [9], and also that the recently discovered *FGFR3* gene fusions (resulting in *FGFR3-TACC3* or *FGFR3-BAlAP2L1*) were only found in a small subgroup of UCs (2/32, 6.25%) [10]. Other FGFR-driven cancers with squamous differentiation include squamous carcinomas of the lung and head and neck [6]. In squamous lung cancer, amplification of *FGFR1* was found in up to 22% of cases [11]. This was associated with reduced cell growth *in vitro* in cells treated with a small molecule inhibitor [12]. However, little is known about FGFR activation in squamous differentiated bladder cancer. Hence, we systematically screened a cohort of squamous differentiated specimens and publically available datasets of “squamous-like” bladder cancers for expression, amplification, mutation and chromosomal rearrangement of *FGFRs* (*FGFR1-3*), in order to evaluate putative pathway activation. Our aim was to evaluate FGFRs as potential therapeutic targets in squamous differentiated bladder cancer, a tumor group in which disease management remains inadequate.

## RESULTS

### *FGFR1*, *FGFR2* and *FGFR3* gene amplification in squamous differentiated bladder cancer

Fluorescence *in situ* hybridization (FISH) was successfully evaluated in a total of 68 samples for *FGFR1,* 65 samples for *FGFR2* and 64 samples for *FGFR3*. Among all suitable samples, no amplification of *FGFR1*, *FGFR2* or *FGFR3* matching the criteria defined by Schildhaus et al. [13] were found (Supplementary Data S1).

Similar to a previous study from our group we then analyzed the cells for polysomy, choosing a cut-off of three centromere signals for polysomy [9]. We identified only 4/68 (5.9%), 3/65 (4.6%) and 0/64 polysomic cases for chromosome 8 (*FGFR1*), 10 (*FGFR2*) and 4 (*FGFR3*), respectively, indicating no considerable amplification bias due to polysomy (Supplementary Data S1).

### *FGFR3*-rearrangement analysis (break apart FISH and cDNA fragment analysis)

**Figure 1:**
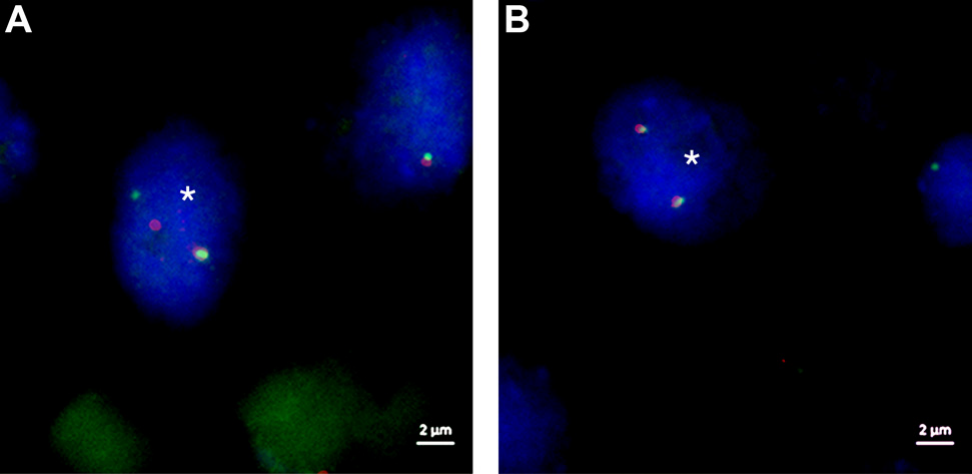
FISH images of *ZytoLight* SPEC^TM^ *FGFR3* Dual Color Break Apart Probe sample ID 51 (**A**) Tumor cell nucleus (*) with a classical break apart event of chromosome region 4p16.3 displaying one single orange (proximal to *FGFR3*) and one single green (distal to *FGFR3*) signal as well as one fusion (adjacent orange-green) signal. (**B**) Tumor cell with normal chromosome region 4p16.3 showing two fusion signals (adjacent orange-green signals) per nucleus. DAPI counterstain, original magnification 1000-fold, white scale bar equals 2 μm.

FISH analysis for *FGFR3* rearrangement was effectively performed on a total of 66 samples. For each tissue microarray we evaluated cores of normal urothelium, showing a mean of 4.22 break apart events. According to Wolff et al. we calculated a cut off for positive cases by using the Microsoft Excel β-inverse function BETAINV [14]. Tumor samples were scored as positive, if nine or more break apart events in 60 tumor cell nuclei were found (Figure 1, Supplementary Data S1). We identified only eight slightly positive samples (ranging from 9 to 11 break apart events, mean 9.5 events/sample). There was no overlap with the polysomic samples mentioned above. Additional cDNA fragment analysis of FISH-positive cases showed sufficient *FGFR3-*cDNA in two available frozen samples (ID #2, #18), but no *FGFR3-TACC3*-fusion product could be verified.

### *FGFR3* mutation analysis and its clinical impact

**Figure 2:**
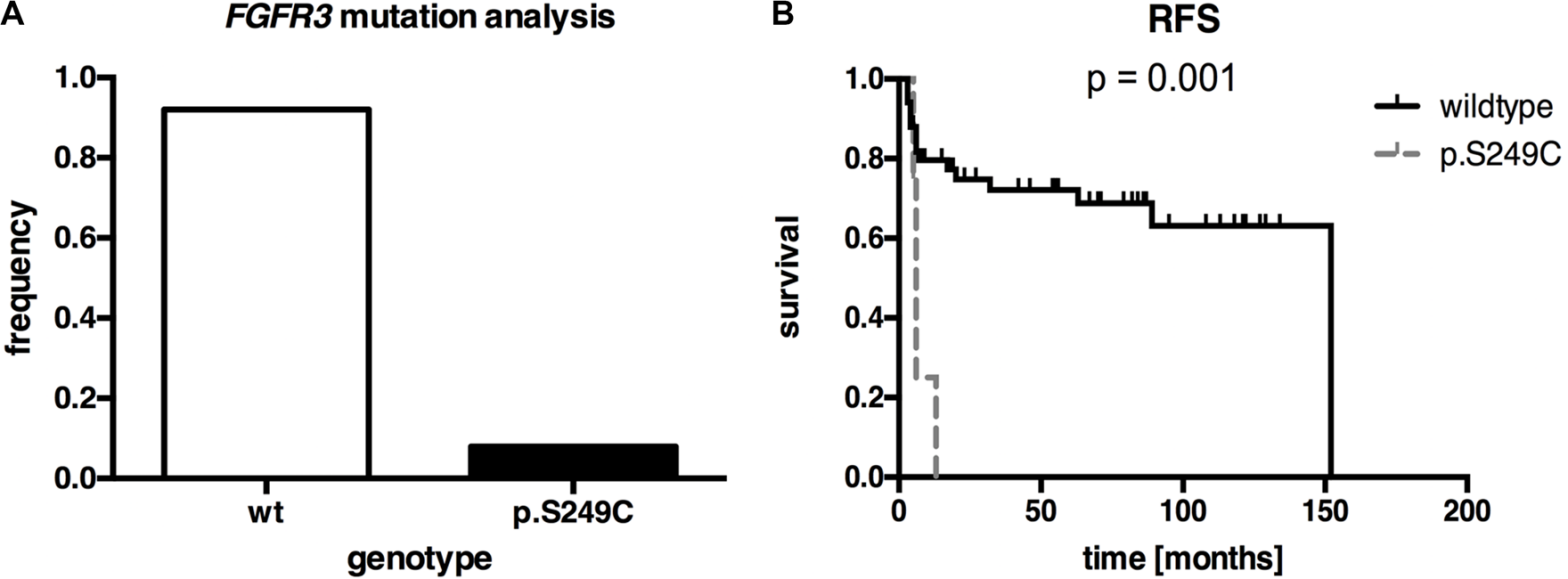
*FGFR3* mutation analysis of our squamous differentiated bladder cancer samples (*n* = 71) (**A**) Frequency of *FGFR3* mutations (8.5%) in our squamous bladder tumors. (**B**) Univariate Kaplan-Meier survival curve illustrating recurrence-free survival (RFS) of patients with *FGFR3* mutation (dashed gray line) compared to non-mutated tumors (black line). Vertical lines: censored cases.

We investigated the squamous differentiated bladder cancers for activating *FGFR3* point mutations. SNapShot^®^-analysis showed *FGFR3* mutations in 6 of 71 samples (8.5%, all p.S249C, *n* = 3 pure squamous carcinomas and *n* = 3 mixed carcinomas) (Figure 2A, Supplementary Data S1). Next, we determined whether there were associations between *FGFR3* mutation and clinico-pathological characteristics. A close association of *FGFR3* mutation and FGFR3 protein overexpression (*p* < 0.001; Fisher's exact test) was demonstrated. No correlations were found with stage, grade, age at diagnosis or gender (Table 1). Even though *FGFR3* mutation was rare in this cohort, we analyzed the recurrence-free survival as a clinical indicator that is known being associated with *FGFR3* mutation in bladder cancer [8]. Kaplan-Meier analysis revealed that patients with a p.S249C *FGFR3* mutation showed a significantly (*p* = 0.001) shorter recurrence-free survival (mean RFS: 7.5 months ± 1.8; 95% CI: 3.9 to 11.1) compared with those with non-mutated tumors (mean RFS: 105.8 months ± 10.2; 95% CI: 85.8 to 125.7) (Figure 2B, Table 2). The calculated Cox regression model (including the potentially prognostic parameters grade, tumor stage, nodal status and metastasis) confirmed the clinical impact of *FGFR3* mutation on recurrence-free survival (Supplementary Data S2). Squamous differentiated bladder cancer patients displaying a p.S249C mutation had a 4.4-fold increased risk for tumor relapse (multivariate hazard ratio (HR): 4.4, 95% CI: 1.0 to 21.8, *p* = 0.046). However, mutation status had no significant influence on disease-specific survival or overall survival in our patient cohort (data not shown).

**Table 1:** Clinico-pathological and demographic data of our squamous differentiated bladder cancer samples cohort in relation to *FGFR3* mutation status

variable	patients (*n*^a^)	non-mutated (*n*)	mutated (*n*)	*p*-value^b^
patient age (years)				
0–67.5	36	34	2	0.377
> 67.5	35	31	4	
gender				
female	39	35	4	0.549
male	32	30	2	
tumor stage				
pT1-2	9	9	0	0.333
pT3-4	62	56	6	
grade				
G1-2	21	20	1	0.472
G3-4	50	45	5	
nodal status				
N0	52	48	4	0.877
N1	11	10	1	
FGFR3 expression				
low expression (Tomlinson-Score < 3)	55	52	3	**< 0.001**
high expression (Tomlinson-Score = 3)	6	3	3	

Bold-face indicates significant results.

a Variations in number due to limited histopathological, experimental or clinical follow up data.

b Calculated by Fisher's exact test.

**Table 2:** Clinico-pathological data of our squamous differentiated bladder cancer samples in regard to recurrence-free survival

variable	patients (*n*^a^)	recurrence (*n*)		*p*-value^b^
		yes	no	
metastasis status				
no metastasis	49	13	36	**< 0.001**
metastasis	7	7	0	
tumor stage				
pT1-2	8	0	8	0.038
pT3-4	55	20	35	
grade				
G1-2	20	5	15	0.185
G3-4	43	15	28	
nodal status				
N0	51	17	34	0.176
N1	6	2	4	
mutational status				
wildtype	57	16	41	**0.001**
p.S249C	5	4	1	
FGFR3 expression				
low expression (Tomlinson-Score < 3)	50	14	36	0.144
high expression (Tomlinson-Score = 3)	5	3	2	

Bold-face indicates significant results.

a Variations in number due to limited histopathological, experimental or clinical follow up data.

a Calculated by log-rank test.

### FGFR1-3 protein expression in bladder cancer

**Figure 3:**
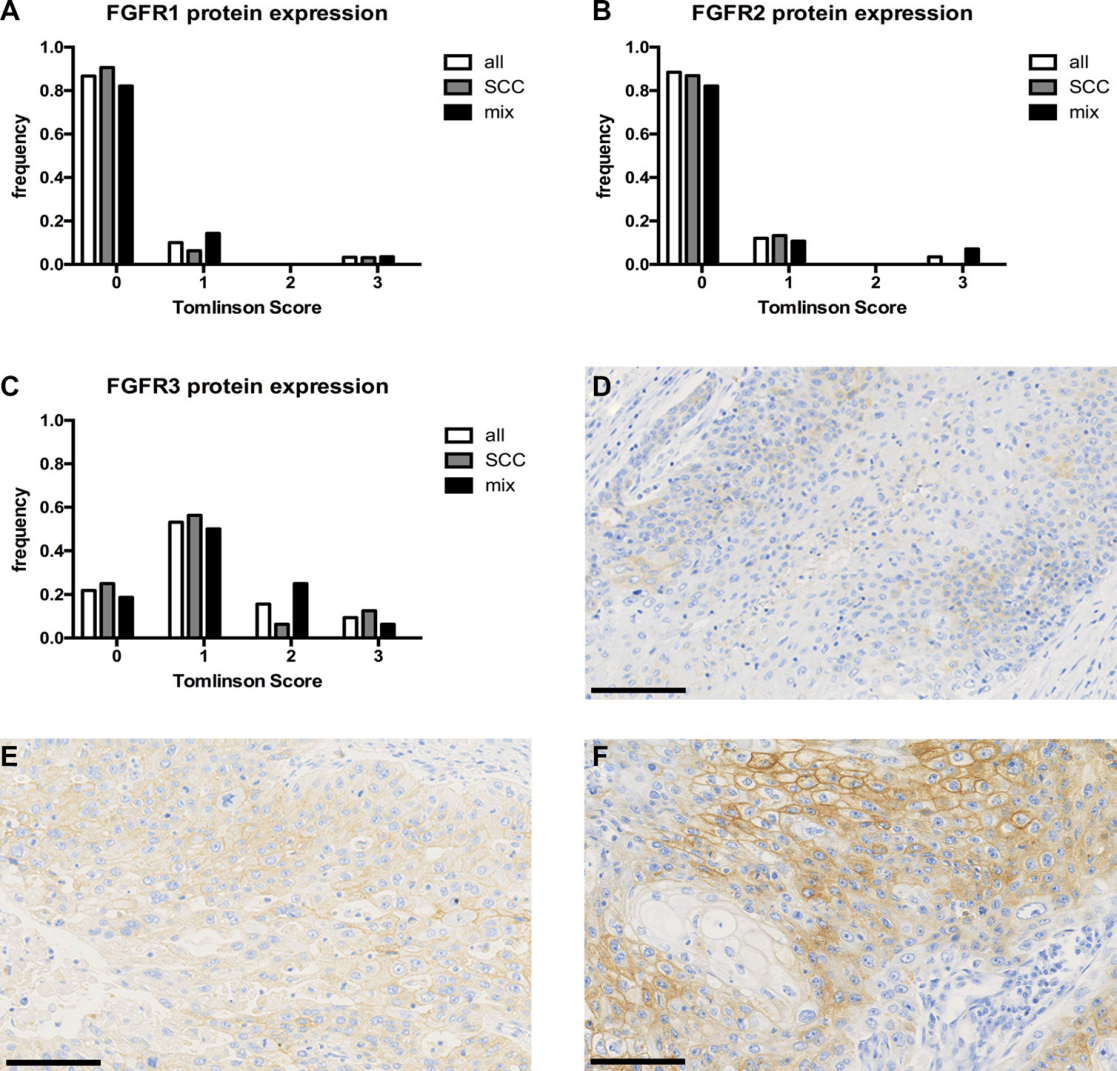
FGFR1-3 protein expression of our squamous differentiated bladder cancer samples Distribution of protein expression measured by Tomlinson Score 0-3 for FGFR1 (**A**) *n* = 60, FGFR2 (**B**) *n* = 58, FGFR3 (**C**) *n* = 64, respectively. (**D**–**F**) anti-FGFR3 staining with examples of Tomlinson Score 1(D), 2(E) and 3(F), original magnification 200-fold. Black scale bar equals 100 μm.

Protein expression was analyzed by immunohistochemical staining and 60, 58 and 64 TMA cores for FGFR1, FGFR2 and FGFR3 could be scored. Evaluation criteria used to define FGFR protein expression were 0 (no expression), 1 (weak expression), 2 (intermediate expression) and 3 (strong expression) as reported by Tomlinson et al. [15]. FGFR1 expression was very low, with 52/60 (86.7%) tumors exhibiting no expression, 6/60 (10%) with weak expression and 2/60 (3.3%) tumors showing strong expression. FGFR2 expression was similarly low: 49/58 (84.5%) displayed no, 7/58 (12.1%) weak and 2/58 (3.4%) strong positivity. FGFR3 protein expression was negative in 14/64 (21.9%) cases, weak in 34/64 (53.1%), intermediate in 10/64 (15.6%) and strong in 6/64 (9.4%) of the examined cores (Figure 3). There was no statistical correlation of FGFR1-3 expression with any clinico-pathological parameter (see Supplementary Data S3).

### External validation of FGFR alterations in an independent cohort of bladder cancer (TCGA)

**Figure 4:**
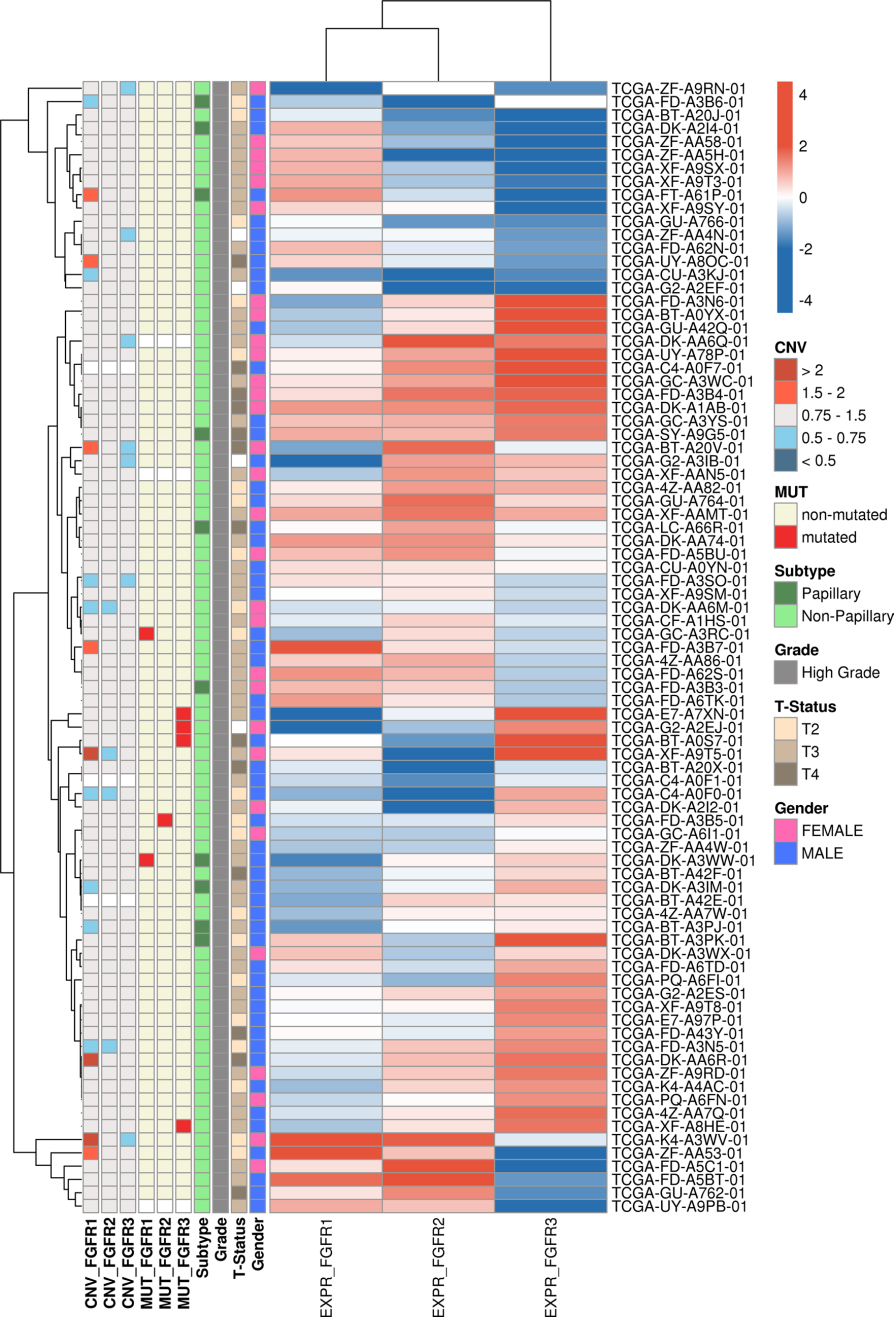
Heatmap showing the *FGFR1, FGFR2* and *FGFR3* mRNA expression in the 85 “squamous-like” bladder cancer samples identified by hierarchical cluster analysis of the entire TCGA cohort Samples are annotated with gender, stage, grade, histological subtype as well as mutational status and CNVs for *FGFR1-3*.

After integration of all currently available bladder cancer datasets new hierarchical cluster analysis based on the mRNA expression of the entire TCGA cohort identified 85 “squamous-like” bladder cancer samples (Figure 4). The new cluster of “squamous-like” tumor samples was consistent with the results previously published [16]. Mutation and CNV data was available for *n* = 82 samples. Mutations of *FGFR1*, *FGFR2* and *FGFR3* were present in 2/82 (2.4%), 1/82 (1.2%), and 4/82 (4.9%) of tumors.

**Figure 5:**
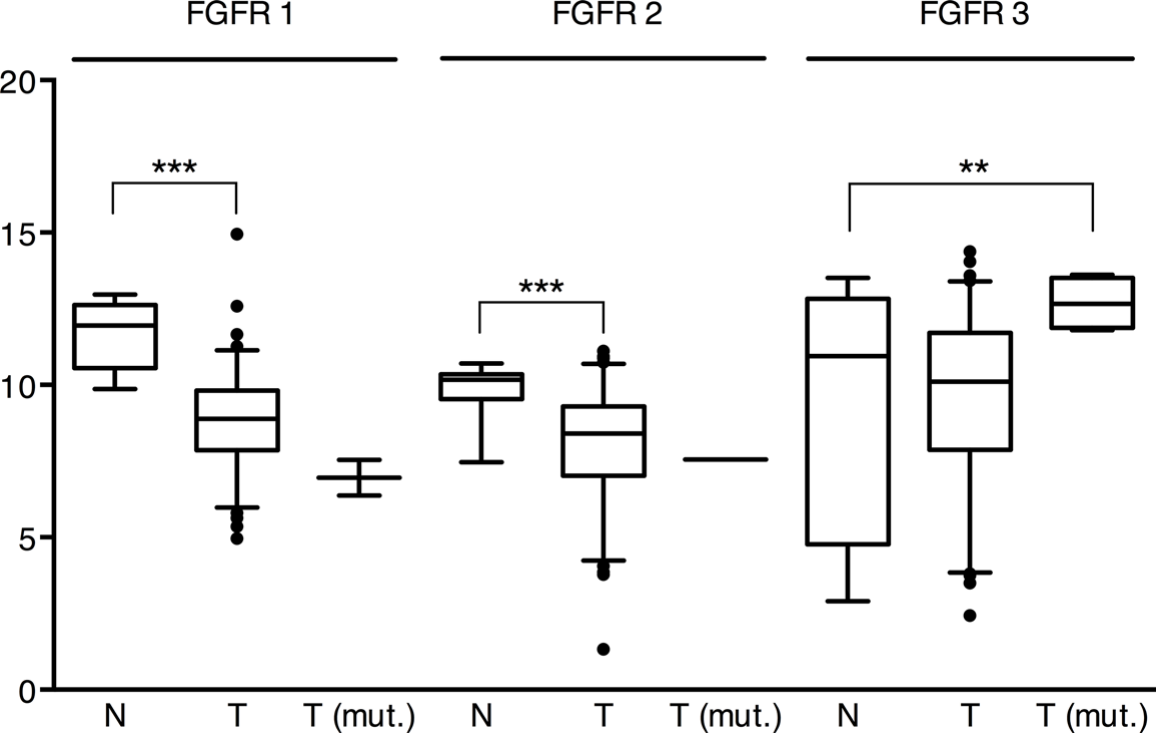
mRNA expression of *FGFR1, 2* and *3* in the “squamous-like” bladder cancer subtype of the TCGA cohort Pairwise applied Welch's *t*-tests indicate that the average expression of *FGFR1* and *FGFR2* was significantly lower (*p* < 10^−6^) in non-mutated tumors (T) compared to normal tissue (*N*), while *FGFR3* mRNA expression in mutated tumors (T(mut.)) was significantly higher (*p* = 0.02). ** = *p* < 0.01, *** = *p* < 0.001.

The average expression of *FGFR1* and *FGFR2* was significantly lower (*p* < 10^−6^) in “squamous-like” tumors compared to normal tissues (95% CI; factor 4.8–10 (*FGFR1*) and 2.5–5.1 (*FGFR2*)) (Figure 5). Furthermore, there was a significant positive correlation between the expression of *FGFR1-FGFR2* (*ρ* = 0.24, *p* = 0.026) and *FGFR2-FGFR3* (*ρ* = 0.23, *p* = 0.031) in tumor tissues, while *FGFR1* expression was inversely correlated with *FGFR3* expression (*ρ* = −0.30, *p* = 0.005). Compared to normal tissues, the average expression of *FGFR3* was significantly higher in tumors with concomitant *FGFR3* mutation (*p* = 0.006), but not in those without (Figure 5). Due to the small number of *FGFR3* mutated tumors a reliable estimate of the effect size was not possible (95% CI; factor 2-34).

The calculated copy numbers of *FGFR1*, *FGFR2* and *FGFR3* deviated from the normal genotype of blood and normal tissue samples in the majority of the “squamous-like” tumors, indicating the general genomic instability of progressed cancers. High level amplifications of *FGFR1*, *FGFR2* and *FGFR3* segments (average of gene copies per chromosome > 2) were detected in 3/82 (3.7%), 0/82 (0%) and 0/82 (0%) of the tumors (Figure 4). Futhermore, 5/82 (6.1%) patients had a less distinctive elevated copy-number of *FGFR1* (average gene copies per chromosome > 1.5). There was no evidence for deep deletion (average gene copies per chromosome < 0.5) of the *FGFR1, FGFR2* and *FGFR3* genes.

## DISCUSSION

Aberrant activation of tyrosine kinase signaling is frequent in various tumors and offers several possibilities for targeted therapies. We focused on FGFRs, since FGFR alterations have been found in SCC tumors in other tissues and *FGFR3* is frequently activated in UC [6, 8]. Our study is the first comprehensive analysis of FGFR alterations potentially contributing to the development of squamous differentiated bladder cancer. We initially studied gene copy number alterations, i.e. *FGFR* amplifications, as a known mechanism of enhanced *FGFR* gene activation. While *FGFR1* amplifications have been found in up to 22% of squamous cell carcinomas of the lung and about 15% in head and neck cancers, we found neither *FGFR1*, *FGFR2*, nor *FGFR3* amplifications by FISH in squamous differentiated bladder cancer [11, 17]. In a previous study of UC by our group we detected only low numbers of amplifications varying from 1.6%, 0.8% and 3.4% respectively for *FGFR1*, *FGFR2* and *FGFR3* [9]. Thus, our data do not indicate *FGFR* amplification as a major mechanism of pathway activation in these bladder cancer subtypes. Contrary to our negative *FGFR1*-FISH results, Helsten et al. found up to 7% of *FGFR1* amplification in their high throughput next-generation sequencing pan-cancer study [6]. However, 3% of *FGFR3* amplifications are comparable with our previous findings in UC.

Besides *FGFR* amplification, recent studies have identified *FGFR* gene fusion products such as *FGFR3-TACC3* as an additional oncogenic mechanism and a putative target for tyrosine kinase inhibitors in a variety of tumors e.g. adeno- and squamous cell carcinoma of the lung, glioblastoma multiforme and bladder cancer [10, 18, 19]. Full-length *FGFR3-TACC3* gene fusion products led to anchorage independent growth and constitutive activation, resulting in increased phosphorylation compared to *FGFR3* wild type receptors [10]. In addition, the fusion products are reported to stimulate proliferation by inducing chromosomal instability and aneuploidy [19]. *FGFR3-TACC3* fusions were found in 2.3% (3/131) samples of the TCGA bladder cancer cohort [3] and overall *FGFR* fusions were reported by Helsten et al. in 6% of their bladder cancer specimens (*FGFR3-TACC3* fusions 3%) [6]. We found eight tumors with only slightly enhanced break apart events by FISH analysis. However, further successful analysis was limited to cryo samples only (*n* = 2), and fusion products could not be confirmed by PCR. Therefore, it remains unresolved whether visible break apart events might be caused by section artifacts or might be unverified due to limited PCR performance using fragmented paraffin-derived RNA or the presence of other fusion partners. Nevertheless, *FGFR3-TACC3* fusions seem to be infrequent alterations in squamous differentiated bladder cancers.

The most common mechanism leading to *FGFR3* activation in bladder cancer is activating point mutation of the gene. By creating two unpaired cysteine residues, the common point mutations induce a ligand-independent receptor activation by forming disulfide-linked receptor dimers, leading to activation of the Ras/MAPK, STAT or PI3K pathway [7]. *FGFR3* mutations are reported in up to 50% of cancers of all stages from the lower and upper urinary tract with p.S249C being the most common mutation, found in 61% of cases [7]. Mutation is inversely correlated with tumor stage and grade [15], and mutated tumors are associated with a favorable clinical outcome [8, 15, 20]. Despite the high mutation frequencies in urothelial carcinoma, the role of *FGFR3* mutations in squamous differentiated bladder cancers is not well defined. In our study 6 of 71 (8.5%) tumor samples (*n* = 3 pure squamous carcinomas and *n* = 3 mixed carcinomas) contained an *FGFR3* p.S249C mutation, all of them being grade 2 or higher and stage 3 or higher. This is slightly less than the frequency in invasive urothelial carcinomas reported by the TCGA project (12%) [3] and the *n* = 126 UC samples of Helsten et al. (15%) [6]. Thus, we suggest that *FGFR3* mutation (and *FGFR3* mRNA expression) plays a minor role in squamous tumorigenesis. However, our survival analysis revealed an association between *FGFR3* mutation and an increased risk of recurrence in squamous differentiated bladder cancers which has also been shown previously for low grade non-invasive UC [8]. Analysis of all invasive bladder cancer TCGA samples displayed a worse prognosis for tumors with p.S249C mutation, whereas other *FGFR3* mutations were associated with good prognosis (data not shown). Thus, mutation analysis potentially allows a further stratification of patients, and should be further evaluated in larger cohorts of invasive tumors.

Interestingly, there was no difference between pure squamous cell carcinoma and mixed carcinomas with squamous differentiation, and validation by a subgroup analysis of the “squamous-like” TCGA subgroup showing 4.9% of *FGFR3* mutations corroborated our results. In addition, mRNA expression analysis of the “squamous-like” TCGA subgroup confirmed generally low levels of *FGFR1-3* mRNA in tumors, except in *FGFR3* mutated ones. Consistent with these findings, five of the six tumors (83.3%) harboring *FGFR3* mutations also showed enhanced FGFR3 protein expression, i.e. Tomlinson Score 2 or 3. This observation is consistent with the reported correlation of FGFR3 overexpression and mutation in urothelial bladder cancer [15, 21]. Overall, protein expression of FGFR1, 2 and 3 was weak and we could not verify an overexpression of FGFR2 in squamous bladder cancers (18%) as reported by Youssef et al. in their squamous bladder cancer cohort from Egypt [22]. However, irrespective of the presence of *FGFR3* mutation, increased FGFR3 protein expression was found in 16/64 (25%) of our squamous differentiated cancers, mostly in G3 tumors (75%). The mechanism of protein overexpression in wild-type tumors is still not fully understood [15]. Statistical analysis of our cohort showed no significant association of FGFR3 overexpression (i.e. Tomlinson Score 3) with recurrence free (RFS), disease specific (DSS) or overall survival (OS) (data not shown). This is in line with previous results of our group for FGFR3 in urothelial carcinomas [9]. However, Sung et al. reported a worse prognosis (OS, DSS) of FGFR3 overexpressing muscle-invasive bladder cancers treated with adjuvant chemotherapy [23]. Due to lack of treatment information for our cohort we could not further analyze refined subgroups, but probably such a subgroup would benefit from targeted anti-FGFR3 therapy.

In light of our novel findings, we suggest a minor role for FGFR alterations in the small subgroup of non-bilharzial squamous bladder cancers. However, bearing in mind that recurrence-free survival is an indicator for disease severity and risk of progression, *FGFR3* mutations in squamous differentiated bladder tumors may indicate potential for FGFR inhibitor treatment in these tumors. This will be particularly interesting as some FGFR inhibitors have been approved by the FDA for the treatment of solid cancers. Currently five clinical trials (www.clinicaltrials.gov [24]; identifier NCT02401542, NCT02529553, NCT02278978, NCT01732107, NCT01004224) are assessing the effects of FGFR inhibitors in bladder and other cancers.

## MATERIALS AND METHODS

### Patient samples, data and ethics

Tissue microarrays (TMA) of previously characterized formalin-fixed, paraffin-embedded squamous differentiated bladder cancer specimens were used [25]. The residual TMAs contained 34 cases of pure squamous cell carcinomas (5 pT2, 24 pT3, 5 pT4) and 39 cases with mixed squamous and urothelial differentiation (5 pT2, 31 pT3, 3 pT4). Mixed tumors used for subsequent analyses contained at least 33.3% of squamous cells. Varying numbers of evaluated cases were due to limited experimental or clinical follow up data.

The local Ethics Committee approved the anonymous use of samples and clinico-pathological data (EK 173/06, 9/12). Clinico-pathological and follow-up data of patients are shown in Supplementary Data S4.

### Fluorescence *in situ* hybridization analysis (FISH)

Hybridization of *ZytoLight* Dual Color Probes SPEC *FGFR1/CEN 8,* SPEC *FGFR2/CEN 10,* SPEC *FGFR3/CEN 4* and *ZytoLight* SPEC *FGFR3* Dual Color Break Apart Probe (Zytovision, Bremerhaven, Germany) onto 3 μm TMA sections was performed according to the manufacturer's protocols. Slides were reviewed on a Zeiss Axiovert 135 fluorescence microscope (Carl Zeiss, Oberkochen, Germany), and Diskus Software (Büro Hilgers, Königswinter, Germany) was used to capture images from different channels/filters (AHF ZyGreen F36-720, AHF ZyOrange F36-740, AHF DAPI, AHF F56-700). The numbers of *FGFR* signals and centromere signals were counted in 60 nuclei of tumor cells at high magnification (x1000), and the *FGFR*/centromere ratio was classified into high- and low-level amplifications or a normal ratio as previously defined [13]. Likewise, 60 tumor cell nuclei were analyzed for break apart events (split signals with minimal distance of two signals or single signals). Specimens were classified “positive for *FGFR3* rearrangements” according to a calculated cut off value, determined on 60 nuclei of normal urothelium, using the Microsoft Excel BETAINV function reported by Wolff et al. [14].

### DNA isolation and *FGFR3* mutation analysis

As described previously, tumor DNA was extracted from microdissected serial sections of the residual tissue blocks using QIAamp™ DNA Mini Kit (Qiagen, Hilden, Germany) [9]. Analysis of 11 known activating *FGFR3* point mutations was performed by SNaPshot^®^ Multiplex System assay (Applied Biosystems, Foster City, USA) as described previously [26, 27].

### RNA-isolation, cDNA-synthesis and PCR-fragment analysis for *FGFR3* gene fusions

RNA was extracted from either microdissected FFPE samples using the RNEasy FFPE Kit (Qiagen, Hilden, Germany) or microdissected from frozen tissue using the Arcturus^®^ PicoPure^®^ RNA Isolation Kit (Applied Biosystems/Life Technologies, Foster City, USA) according to the manufacturer's instructions. cDNA was synthesized with Promega Kit A3500 (Promega, Madison, WI, USA).

*FGFR3-TACC3* fusions were assessed by PCR, adapted from Williams et al. [10], using an *FGFR3* forward primer (positioned in *FGFR3* exon 18) and four different reverse primers localized in exons 4, 9, 11 and 13 of *TACC3* covering the most common *FGFR3-TACC3* fusions (for primer sequences and expected PCR product sizes see Supplementary Data S5A and S5B). RT4 cell line cDNA was used as a positive control for the fusion PCRs and cDNA quality was tested with a control PCR (both primers localized on *FGFR3*: *FGFR3* 14F and *FGFR3* 16R) resulting in a 400 bp product.

### Immunohistochemical analysis of FGFR1-3 protein expression

Immunohistochemical staining for FGFR1, FGFR2 and FGFR3 was performed on 3 μm TMA sections after heat-induced antigen retrieval (EnVision™ FLEX Target Retrieval Solution, Low pH, K8005, DAKO PT-Link, DAKO, Hamburg, Germany) according to the manufacturer's protocols. The primary antibodies (anti-FGFR3, mouse monoclonal clone B9, dilution 1:25 (Santa Cruz Biotechnology, Heidelberg, Germany), anti-FGFR2, mouse monoclonal clone 1G3, dilution 1:400 (www.antikoerper-online.de, Aachen, Germany) and anti-FGFR1, rabbit polyclonal, dilution 1:100 (Sigma, St. Louis, MO, USA)) were linked with DAKO EnVision™FLEX system and visualized with DAKO Liquid DAB Substrate Chromogen System in a DAKO Autostainer plus (K8024, K3468, DAKO). FGFR1, FGFR2 and FGFR3 positivity was assessed according to a semi-quantitative scoring system reported by Tomlinson et al. [15].

### Validation of *FGFR* alterations in an independent set of “squamous-like” bladder cancers

For external validation datasets of a “squamous-like” subtype of chemotherapy-naive, high-grade muscle-invasive urothelial bladder carcinomas from The Cancer Genome Atlas (TCGA) were used [3]. Subtyping was performed based on mRNA expression information (Illumina Genome Analyzer and Illumina HiSeq 2000 RNASeqV2 Platforms; Level 3) of all currently accessible tumor samples (https://tcga-data.nci.nih.gov/tcga/findArchives.htm) [28] as previously published (for details see Supplementary Data S6) [16]. This approach identified 85 bladder cancer samples with a “squamous-like” gene expression profile from all 408 bladder cancer samples. For clinico-pathological parameters of the TCGA cohort see Supplementary Data S7.

Data on gene copy number variations (CNV; Affymetrix Genome-Wide SNP Array 6.0 with a fixed probe set and Illumina HiSeq Platforms; Level 3), somatic mutations (Illumina Genome Analyzer and Illumina HiSeq2000 DNASeq Platform; Level 3), and mRNA expression were analyzed. For samples with CNV data from both platforms or multiple segments within the gene region, values were summarized by extracting the maximum absolute value. Deviating from this one tile that was based on two probes, likely caused by a SNP, was removed for CNV analysis of FGFR1 in one sample (TCGA-ZF-AA4N-01). Data on somatic mutations were joined and a gene was assessed as mutated, if at least one non-silent mutation was reported within the gene body. Quantile normalized mRNA expression values were log^2^-transformed to reduce skewness prior to any statistical tests.

### Statistical analysis

Statistical analyzes of our experimental data were accomplished with SPSS software version 22.0 (SPSS Inc., Chicago, USA). Two-sided *p*-values less than 0.05 were considered significant. Statistical associations between clinico-pathological and molecular factors were determined by Fisher's exact test. Survival curves for recurrence-free survival (RFS), disease-specific survival (DSS) and overall survival (OS) were calculated using the Kaplan-Meier method with log-rank statistics. RFS/DSS/OS were measured from surgery until local or distant relapse/tumor death/death and were censored for patients alive without evidence of relapse/tumor related death/death at the last follow-up. Multivariate Cox-regression analysis was performed to test for an independent prognostic value of *FGFR3* mutations.

The evaluation of TCGA data was performed in R [29]. For cluster analysis routines from additional software packages were used (Supplemental Digital Content 4) [30–34]. Pairwise comparison of mRNA expression between groups was tested by Welch's *t*-test. Correlations between the expression of genes were determined by Spearman rank correlation coefficient.

## SUPPLEMENTARY MATERIALS TABLES








