# Supplementary material for: Fibroblast growth factor receptor (FGFR) alterations in squamous differentiated bladder cancer: a putative therapeutic target for a small subgroup
Source: Oncotarget. 2016 Sep 22;7(44):71429–39. doi: 10.18632/oncotarget.12198 (PMC5342089; doi:10.18632/oncotarget.12198)
Supplement: Supplementary file 1 [file oncotarget-07-71429-s001.pdf]

# Fibroblast growth factor receptor (FGFR) alterations in squamous differentiated bladder cancer: a putative therapeutic target for a small subgroup

## Supplementary Materials

**Supplementary Data S1: FISH (*FGFR1*, *FGFR2* and *FGFR3*) and mutation data (*FGFR3*) for our squamous differentiated bladder cancer samples.** See Supplementary_Data_S1

**Supplementary Data S2: Multivariate Cox regression analysis including all factors potentially influencing recurrence free survival (RFS)**

| variable | HR | *p*-value | 95%CI | |
|---|---|---|---|---|
| | | | lower | upper |
| mutation status | **4.378** | **0.046** | 1.031 | 21.769 |
| tumor stage | 264993.6 | 0.971 | < 0.001 | – |
| grade | 1.334 | 0.621 | 0.425 | 4.189 |
| nodal status | 0.569 | 0.610 | 0.065 | 4.969 |
| metastasis status | 2.815 | 0.078 | 0.891 | 8.889 |

RFS = recurrence-free survival, HR = hazard ratio, CI = confidence interval.

**Supplementary Data S3: Clinico-pathological and demographic details of our squamous differentiated bladder cancer sample cohort in relation to FGFR3 expression**

| variable | patients ($n^a$) | low expression ($n$) | high expression ($n$) | $p$-value[b] |
|---|---|---|---|---|
| patient age (years) | | | | |
| 0–67.5 | 30 | 26 | 4 | 0.330 |
| > 67.5 | 33 | 31 | 2 | |
| gender | | | | |
| female | 34 | 30 | 4 | 0.515 |
| male | 29 | 27 | 2 | |
| tumor stage | | | | |
| pT1–2 | 6 | 6 | 0 | 0.407 |
| pT3–4 | 57 | 51 | 6 | |
| grade | | | | |
| G1–2 | 15 | 13 | 2 | 0.568 |
| G3–4 | 48 | 44 | 4 | |
| nodal status | | | | |
| N0 | 47 | 41 | 6 | 0.261 |
| N1 | 9 | 9 | 0 | |
| metastasis status | | | | |
| no metastasis | 55 | 50 | 5 | 0.532 |
| metastasis | 4 | 4 | 0 | |
| mutation status | | | | |
| non-mutated | 55 | 52 | 3 | **0.001** |
| mutated | 6 | 3 | 3 | |

Bold-face indicates significant results.

[a]Variations in number due to limited histopathological, experimental or clinical follow up data.

[b]Calculated by Fisher's exact test.

**Supplementary Data S4: Clinico-pathological and follow-up data of our squamous differentiated bladder cancer cohort.** See Supplementary_Data_S4

**Supplementary Data S5A: PCR primer sequences for cDNA fragment analysis of FGFR3-TACC3 fusion products**

| Primer name | Forward primer (5′−3′) | Reverse primer (5′−3′) |
|---|---|---|
| FGFR3 14F | GTACGTGCTGGTGGAGTACG | |
| FGFR3 16R | | ACGTCACTCTGGTGAGTGTAGA |
| FGFR3 18F | AGAGGCCCACCTTCAAGC | |
| TACC3 4R 5′ | | GCCAGACACTTTTCCTGGAG |
| TACC3 9R | | TGCTCCCAAGAAATCGAACT |
| TACC3 11R | | CTCACACCTGCTCCTCAGC |
| TACC3 13R | | TTTCTGGATTTCAGCTTTGG |

**Supplementary Data S5B: Approximate PCR product size of FGFR3-TACC3 fusion genes (using forward primer FGFR3 18F)**

| point of fusion in FGFR3 | reverse primer | | | |
|---|---|---|---|---|
| | TACC3 4R 5′ | TACC3 9R | TACC3 11R | TACC3 13R |
| Ex 4 (RT4) | 282 bp | 1681 bp | 1866 bp | 2005 bp |
| Ex 4 (distal) | – | 551 bp | 736 bp | 875 bp |
| Ex 8 | – | 283 bp | 468 bp | 607 bp |
| Ex 9 (mid) | – | – | ~280 bp | ~419 bp |
| Ex 10 | – | – | 236 bp | 375 bp |
| Ex 11 | – | – | 131 bp | 270 bp |

**Supplementary Data S6: Description of the hierarchial pan-cancer cluster analysis used for the identification of “squamous-like” bladder cancer samples of the current TCGA cohort.**

See Supplementary_Data_S6

**Supplementary Data S7: Clinico-pathological parameters of the identified "squamous-like" bladder cancer subgroup samples of the TCGA cohort**

| variable | patients ($n$; ∑=85) |
|---|---|
| patient age (years) | |
| 0–67 | 41 |
| > 67 | 44 |
| gender | |
| female | 30 |
| male | 55 |
| tumor stage | |
| pT1–2 | 21 |
| pT3–4 | 60 |
| n.a. | 4 |
| nodal status | |
| N0 | 54 |
| N1–3 | 25 |
| Nx | 6 |
| metastasis status | |
| M0 | 36 |
| Mx | 49 |
| histologic grade | |
| high grade | 85 |
| subtype | |
| non-papillary | 75 |
| papillary | 10 |

*n* = number, n.a. = not available.
