# Supplementary material for: Fibroblast growth factor receptor (FGFR) alterations in squamous differentiated bladder cancer: a putative therapeutic target for a small subgroup
Source: Oncotarget. 2016 Sep 22;7(44):71429–39. doi: 10.18632/oncotarget.12198 (PMC5342089; doi:10.18632/oncotarget.12198)
Supplement: Supplementary file 2 [file oncotarget-07-71429-s002.docx]

**Supplementary Data 1** – FISH (*FGFR1*, *FGFR2* and *FGFR3*) and mutation data (*FGFR3*) for our squamous differentiated bladder cancer samples

| **sample number** | **ratio *FGFR1/CEN8*** | **ratio *FGFR2/CEN10*** | **ratio *FGFR3/CEN4*** | **number of SPEC^TM^ *FGFR3* break aparts** | **polysomy** | **mutational status** |
| --- | --- | --- | --- | --- | --- | --- |
| 1 | 0.99 | 0.69 | 0.86 | 9 | yes: Chr 8,10 | wt |
| 2 | 0.71 | 0.85 | 0.97 | 11 | no | wt |
| 3 | 1.04 | 0.6 | n/a | n/a | yes: Chr 8, 10 | wt |
| 4 | 0.97 | 0.97 | 1.03 | n/a | no | wt |
| 5 | 0.63 | 1.09 | 0.89 | n/a | no | wt |
| 6 | 0.84 | 0.64 | 1.06 | 4 | no | wt |
| 7 | 1.07 | 0.98 | 0.98 | 6 | no | p.S249C |
| 8 | 1.03 | 0.93 | 0.96 | 2 | no | wt |
| 9 | 0.61 | 0.82 | 0.93 | 3 | no | wt |
| 10 | 0.65 | 0.88 | 0.99 | 3 | no | wt |
| 11 | 0.95 | 0.76 | 0.98 | n/a | no | wt |
| 12 | 1.03 | 0.71 | 0.99 | 4 | no | p.S249C |
| 13 | 0.77 | 0.77 | 0.97 | n/a | no | wt |
| 14 | 0.95 | n/a | 0.78 | n/a | yes: Chr 8 | wt |
| 15 | 0.9 | 0.96 | 0.95 | 7 | no | wt |
| 16 | 0.93 | 0.9 | 0.98 | n/a | no | wt |
| 17 | 0.39 | 0.98 | 0.97 | 5 | no | wt |
| 18 | 0.78 | 1.04 | 0.91 | 11 | no | wt |
| 19 | 0.87 | n/a | n/a | n/a | no | wt |
| 20 | 0.98 | n/a | n/a | n/a | yes: Chr 8 | wt |
| 21 | 0.94 | 0.85 | 1.11 | 5 | no | wt |
| 22 | 1.05 | 0.91 | 1.02 | 4 | no | wt |
| 23 | 0.86 | 0.76 | 1.4 | 6 | no | wt |
| 24 | n/a | 0.86 | 1.11 | 7 | no | wt |
| 25 | 1.15 | 1.02 | 0.99 | 4 | no | wt |
| 26 | 1.02 | 0.91 | 1.02 | 3 | no | wt |
| 27 | 1.02 | 0.86 | 0.89 | 8 | no | wt |
| 28 | n/a | n/a | n/a | n/a | no | wt |
| 29 | 1.02 | 0.96 | 0.97 | 6 | no | wt |
| 30 | 0.98 | 0.81 | 1.14 | 8 | no | wt |
| 31 | 0.92 | 0.93 | n/a | 9 | no | n/a |
| 32 | 1.08 | 1.04 | 1.1 | 4 | no | wt |
| 33 | 0.98 | 0.98 | 1.02 | 9 | no | wt |
| 34 | 0.91 | 0.95 | n/a | 9 | no | wt |
| 35 | 1.03 | 0.86 | n/a | n/a | no | wt |
| 36 | 1.03 | 1.07 | 0.93 | 3 | no | wt |
| 37 | 0.97 | 1.0 | 1.25 | 3 | no | wt |
| 38 | 1.05 | 1.01 | 0.98 | 3 | no | wt |
| 39 | 0.99 | 0.57 | 0.99 | 7 | no | wt |
| 40 | 0.62 | 0.84 | 1.06 | 5 | no | wt |
| 41 | 1.06 | 0.73 | 1.02 | 6 | no | wt |
| 42 | 0.86 | 0.63 | 1.0 | 8 | no | wt |
| 43 | n/a | 1.06 | 1.02 | 5 | no | wt |
| 44 | 0.81 | 0.85 | 0.95 | 4 | no | p.S249C |
| 45 | 1.01 | 0.98 | 1.14 | 2 | no | wt |
| 46 | 0.86 | 1.23 | 0.83 | 5 | no | p.S249C |
| 47 | 0.82 | n/a | 0.97 | 7 | no | wt |
| 48 | 0.96 | 0.76 | 0.97 | 2 | no | wt |
| 49 | 1.06 | 1.07 | 0.96 | 5 | no | wt |
| 50 | 1.02 | 0.95 | 1.03 | 2 | no | wt |
| 51 | 0.99 | 0.95 | 0.98 | 9 | no | wt |
| 52 | 1.07 | 0.97 | 1.03 | 1 | no | p.S249C |
| 53 | n/a | n/a | n/a | 6 | no | wt |
| 54 | 0.87 | 1.01 | 1.02 | 9 | no | wt |
| 55 | 1.67 | 0.49 | 0.97 | 6 | no | wt |
| 56 | 0.93 | 0.8 | 1.07 | 6 | no | wt |
| 57 | 0.97 | 1.0 | 1.21 | 5 | no | wt |
| 58 | 1.02 | 0.89 | 0.71 | 8 | no | p.S249C |
| 59 | 1.08 | 0.99 | 1.10 | 5 | no | wt |
| 60 | 1.07 | n/a | 1.0 | n/a | no | wt |
| 61 | 1.68 | 0.85 | 0.95 | n/a | no | wt |
| 62 | 0.92 | 0.97 | 1.01 | 5 | no | wt |
| 63 | 0.91 | 0.87 | 1.08 | 7 | no | wt |
| 64 | 0.72 | 0.79 | 0.93 | 7 | no | wt |
| 65 | 0.87 | 0.62 | 1.02 | 3 | no | wt |
| 66 | 1.02 | 0.88 | 0.95 | n/a | no | wt |
| 67 | 1.03 | 0.76 | 1.15 | 6 | no | wt |
| 68 | 0.91 | 0.95 | 1.1 | n/a | no | wt |
| 69 | 1.0 | 1.0 | 0.93 | 6 | no | wt |
| 70 | 1.15 | 1.0 | 0.93 | 7 | no | wt |
| 71 | 1.05 | 0.86 | 0.93 | 5 | yes: Chr 10 | wt |
| 72 | 0.99 | 1.0 | 0.85 | 4 | no | n/a |
| 73 | n/a | n/a | n/a | n/a | n/a | wt |

CEN=centromere, SPEC^TM^=single copy probe, n/a=not available, wt=wild type, Chr=chromosome
