# Supplementary material for: Fibroblast growth factor receptor (FGFR) alterations in squamous differentiated bladder cancer: a putative therapeutic target for a small subgroup
Source: Oncotarget. 2016 Sep 22;7(44):71429–39. doi: 10.18632/oncotarget.12198 (PMC5342089; doi:10.18632/oncotarget.12198)
Supplement: Supplementary file 3 [file oncotarget-07-71429-s003.docx]

**Supplementary Data 4:** Clinico-pathological and follow-up data of our squamous differentiated bladder cancer cohort

| **sample number** | **gender** | **age** | **grade** | | **stage** | | **RFS [months]** | **type of recurrence** | **DSS [months]** | **OS [months]** | **death of tumor** |
| --- | --- | --- | --- | --- | --- | --- | --- | --- | --- | --- | --- |
| 1 | m | 77 | | 2 | | 3 | 17 | local + LN |  | 17 | no |
| 2 | m | 57 | | 3 | | 3 | 77 | lung + LN |  | 89 | no |
| 3 | f | 67 | | 3 | | 3a |  |  |  | 87 | no |
| 4 | f | 76 | | 3 | | 3b | 4 | local |  | 6 | no |
| 5 | f | 71 | | 3 | | 3 |  |  |  | 15 | no |
| 6 | m | 71 | | 3 | | 3a |  |  |  | 1 | no |
| 7 | f | 86 | | 3 | | 3 |  |  | 0 | 0 | yes |
| 8 | f | 78 | | 3 | | 4 | 3 | bone | 4 | 4 | yes |
| 9 | f | 46 | | 2 | | 3 |  |  |  | 8 | no |
| 10 | f | 56 | | 2 | | 3 |  |  |  | 3 | no |
| 11 | m | 78 | | 3 | | 3 |  |  |  | 23 | no |
| 12 | f | 81 | | 3 | | 4 | 13 | local |  | 13 | no |
| 13 | f | 62 | | 3 | | 3b |  |  |  | 108 | no |
| 14 | f | 62 | | 3 | | 3a |  |  |  | 18 | no |
| 15 | f | 77 | | 3 | | 3b |  |  |  | 2 | no |
| 16 | f | 54 | | 3 | | 4 | 6 | colon + LN |  | 8 | no |
| 17 | m | 76 | | 3 | | 2b |  |  |  | 84 | no |
| 18 | f | 35 | | 3 | | 3b |  |  |  | 3 | no |
| 19 | f | 66 | | 3 | | 3a | 20 | ureter + LN |  | 35 | no |
| 20 | f | 47 | | 2 | | 2 |  |  |  | 19 | no |
| 21 | m | 52 | | 3 | | 4b |  |  |  | 5 | no |
| 22 | m | 74 | | 2 | | 3b |  |  |  | 127 | no |
| 23 | f | 76 | | 3 | | 3b |  |  | 19 | 19 | n/a |
| 24 | f | 75 | | 3 | | 3a | 3 | local + LN | 6 | 6 | yes |
| 25 | f | 80 | | 3 | | 3a |  |  | n/a | 5 | n/a |
| 26 | m | 48 | | 3 | | 3b |  |  |  | 118 | no |
| 27 | m | 53 | | 3 | | 3 |  |  |  | 95 | no |
| 28 | f | 76 | | 2 | | 2a |  |  |  | 134 | no |
| 29 | f | 79 | | 3 | | 3a | n/a |  | 4 | 4 | yes |
| 30 | f | 53 | | 3 | | 3a |  |  |  | 42 | no |
| 31 | m | 79 | | 3 | | 2 | n/a |  | 8 | 8 | yes |
| 32 | f | 56 | | 3 | | 3b | n/a |  | 2 | 2 | yes |
| 33 | f | 70 | | 2 | | 2 | n/a |  |  | 140 | n/a |
| 34 | f | 43 | | 2 | | 3a |  |  |  | 70 | no |
| 35 | m | 63 | | 2 | | 2b |  |  |  | 82 | no |
| 36 | f | 64 | | 2 | | 3a |  |  |  | 1 | no |
| 37 | m | 55 | | 2 | | 3a |  |  |  | 86 | no |
| 38 | f | 58 | | 2 | | 2b |  |  |  | 67 | no |
| 39 | m | 49 | | 3 | | 4b | 3 | local | 11 | 11 | yes |
| 40 | m | 70 | | 3 | | 3b |  |  |  | 89 | no |
| 41 | m | 62 | | 3 | | 2b |  |  |  | 79 | no |
| 42 | f | 88 | | 3 | | 3a | 3 | local | 5 | 5 | yes |
| 43 | m | 59 | | 3 | | 3b | 63 | lung + bone | 68 | 68 | yes |
| 44 | m | 59 | | 3 | | 3b | 6 | local + LN | 8 | 8 | yes |
| 45 | m | 58 | | 3 | | 3a |  |  |  | 122 | no |
| 46 | f | 74 | | 3 | | 4a | 5 | lung + bone | 10 | 10 | yes |
| 47 | f | 61 | | 4 | | 3a |  |  | 27 | 27 | yes |
| 48 | m | 68 | | 2 | | 2b |  |  |  | 56 | no |
| 49 | f | 34 | | 3 | | 3b |  |  |  | 71 | no |
| 50 | f | 65 | | 2 | | 4a |  |  |  | 46 | no |
| 51 | m | 57 | | 2 | | 3b |  |  |  | 1 | no |
| 52 | m | 75 | | 2 | | 4 | 6 | LN + lung | 20 | 20 | yes |
| 53 | m | 68 | | 2 | | 3b | 152 | LN + lung + bone + adrenal gland | 164 | 164 | yes |
| 54 | f | 61 | | 2 | | 3b |  |  |  | 54 | no |
| 55 | m | 75 | | 2 | | 3b | 4 | pelvis | 7 | 7 | yes |
| 56 | m | 68 | | 3 | | 3b | 4 | lung + pancreas | 15 | 15 | yes |
| 57 | m | 33 | | 3 | | 3b |  |  |  | 129 | no |
| 58 | f | 61 | | 3 | | 3a | n/a |  | n/a | n/a | n/a |
| 59 | m | 65 | | 3 | | 3a | 0 | LN + lung | 3 | 3 | yes |
| 60 | f | 79 | | 2 | | 3a |  |  |  | 113 | no |
| 61 | m | 77 | | 3 | | 3b |  |  |  | 7 | no |
| 62 | m | 61 | | 3 | | 3a |  |  |  | 121 | no |
| 63 | f | 80 | | 4 | | 3b |  |  |  | 5 | no |
| 64 | f | 68 | | 3 | | 3b | n/a |  | 12 | 12 | yes |
| 65 | m | 67 | | 2 | | 3a | 32 | lung + bone + liver | 52 | 52 | yes |
| 66 | m | 82 | | 3 | | 3b |  |  |  | 3 | no |
| 67 | f | 83 | | 3 | | 3b | n/a |  | 6 | 6 | yes |
| 68 | m | 52 | | 3 | | 3a |  |  |  | 82 | no |
| 69 | m | 81 | | 3 | | 3a | 7 | pelvis |  | 7 | no |
| 70 | f | 80 | | 3 | | 3b | 6 | pelvis, ileocoecal | 7 | 7 | yes |
| 71 | f | 87 | | 3 | | 2a |  |  |  | 55 | no |
| 72 | m | 70 | | 3 | | 3b |  |  |  | 115 | no |
| 73 | m | 78 | | 3 | | 3a |  |  |  | 9 | no |

m=male, f=female, RFS=recurrence free survival, DSS=disease specific survival, OS=overall survival, LN=lymph node, n/a = no data available
