# Supplementary material for: Fibroblast growth factor receptor (FGFR) alterations in squamous differentiated bladder cancer: a putative therapeutic target for a small subgroup
Source: Oncotarget. 2016 Sep 22;7(44):71429–39. doi: 10.18632/oncotarget.12198 (PMC5342089; doi:10.18632/oncotarget.12198)
Supplement: Supplementary file 4 [file oncotarget-07-71429-s004.docx]

**Supplementary Data 6:** Description of the hierarchical pan-cancer cluster analysis used for the identification of “squamous-like” bladder cancer samples of the current TCGA cohort

In order to identify bladder cancer samples with a squamous-like subtype in the TCGA cohort a hierarchical cluster analysis on mRNA expression was used as described by others (Hoadley et al; 2014) [1]. The recently available datasets (see below Table S1) on mRNA expression in 5378 tumor samples from 12 entities from The Cancer Genome Atlas (TCGA) were used for this approach. Quantile normalized gene expression values (RSEM) were log2 transformed and treated as not available if they had a value of zero. Variations between the Illumina GAIIx (COAD, READ, UCEC, LAML) and Illumina HiSeq (all except of LAML) platform were estimated based on data from 19 COAD samples that were measured on both platforms.

For this the gene wise median log2-transformed gene expression in this set was calculated for both platforms. The gene-wise difference of the medians between the platforms was then subtracted from GAIIx samples. Thereafter all samples were merged. For 20 samples that were present in both datasets the average of both platforms was calculated.

We used a dataset consisting of the samples that were used in the original published Pan-Cancer Analysis to select the top 6,000 most variable (standard deviation) genes that were measured in at least 70% of the samples.

Expression data on these 6,000 genes was then extracted from all recently available 5378 samples. A hierarchical cluster analysis was applied to these samples. As in the original publication the ConsensusClusterPlus package (Version 1.24.0) for R (Version 3.2.3) [2,3] was used for this purpose. Additionally, faster implementations of the hierarchical clustering algorithms from the flashClust package (Version 1.1.2) were used [4]. Distances between samples were calculated from spearman correlation coefficients. A consensus of 1000 iterative created clusters (80% of samples, average linkage, 2-22 cluster) was calculated by average linkage.

The formation of 11 main-clusters was observable when 16 main clusters were used as previously described [1]. We needed to define a higher number of clusters (20 instead of 16) due to the higher number of samples and the consequently larger amount of outliers (see below Table S2 and Table S3). Summarized, our cluster analysis with 1800 additional samples is in concordance with the previously published analysis. The clustering that we obtained for all recently available samples and their their previous clustering can be found in Table S4. We selected the 85 bladder cancer samples that clustered in cluster number 2 which contains the majority of HNSC and LUSC samples for our analysis of “squamous-like” bladder cancers.

**References:**

[1] Hoadley KA, Yau C, Wolf DM, et al. Multiplatform analysis of 12 cancer types reveals molecular classification within and across tissues of origin. Cell. 2014; 158: 929-944.

[2] R: A language and environment for statistical computing [computer program]. Version 3.2.3. R Foundation for Statistical Computing, Vienna, Austria; 2016.

[3] ConsensusClusterPlus [computer program]. R package version 1.24.0. Wilkerson M and Waltman P; 2013.

[4] Langfelder P, Horvath S. Fast R Functions for Robust Correlations and Hierarchical Clustering. J Stat Softw. 2012; 46.

Table S1: Summary of TCGA RNASeqV2 datasets that were used for hierarchical cluster analysis. Samples were measured on the Illumina GAIIx (GAIIx) or Illumina HiSeq (HiSeq) platform. Some COAD (19) and UCEC (1) samples were measured on both platforms

| Disease | Abbrevation | Platform | Tumor samples | Version |
| --- | --- | --- | --- | --- |
| Bladder Urothelial Carcinoma | BLCA | HiSeq | 408 | 1.18.0 |
| Breast invasive carcinoma | BRCA | HiSeq | 1095 | 1.11.0 |
| Colon adenocarcinoma | COAD | GAIIx / HiSeq | 193 / 286 (19) | 1.1.0 / 1.12.0 |
| Glioblastoma multiforme | GBM | HiSeq | 156 | 1.2.0 |
| Head and Neck squamous cell carcinoma | HNSC | HiSeq | 520 | 1.9.0 |
| Kidney renal clear cell carcinoma | KIRC | HiSeq | 533 | 1.6.0 |
| Acute Myeloid Leukemia | LAML | GAIIx | 173 | 1.2.0 |
| Lung adenocarcinoma | LUAD | HiSeq | 515 | 1.14.0 |
| Lung squamous cell carcinoma | LUSC | HiSeq | 502 | 1.9.0 |
| Ovarian serous cystadenocarcinoma | OV | HiSeq | 305 | 1.2.0 |
| Rectum adenocarcinoma | READ | GAIIx / HiSeq | 72 / 94 | 1.0.0 / 1.9.0 |
| Uterine Corpus Endometrial Carcinoma | UCEC | GAIIx / HiSeq | 370 / 176 (1) | 1.0.0 / 1.12.0 |

Table S2: Confusion matrix of previously designated clusters in columns and new obtained clusters in rows for the samples

| Cluster | 1 | 2 | 3 | 4 | 5 | 6 | 7 | 8 | 9 | 10 | 11 | 12 | 13 | 14 | 15 | 16 |
| --- | --- | --- | --- | --- | --- | --- | --- | --- | --- | --- | --- | --- | --- | --- | --- | --- |
| 1 | 76 | - | - | 6 | - | 1 | - | - | - | - | - | - | - | - | - | - |
| 2 | 1 | 1 | - | 554 | 1 | - | - | 1 | - | - | - | - | - | - | - | - |
| 3 | - | 7 | 1 | 1 | - | 171 | - | - | - | - | - | - | - | - | - | 5 |
| 4 | - | - | 9 | - | - | - | - | - | - | - | - | - | - | - | - | - |
| 5 | - | 2 | - | 1 | - | - | - | - | - | - | - | - | - | - | - | - |
| 6 | - | - | - | - | - | - | 3 | - | - | - | - | - | 176 | - | - | - |
| 7 | - | - | - | - | - | - | - | - | - | - | - | - | - | - | 264 | - |
| 8 | - | - | - | 3 | - | - | - | - | - | - | - | 9 | - | - | - | - |
| 9 | - | - | - | - | - | - | 695 | 1 | - | - | - | - | - | - | - | - |
| 10 | - | - | - | - | - | - | - | 135 | - | - | - | - | - | - | - | - |
| 11 | - | - | - | - | - | - | - | - | - | - | - | - | - | - | - | - |
| 12 | - | - | - | - | - | - | 3 | - | - | - | - | - | - | - | - | - |
| 13 | 2 | - | - | - | - | 1 | - | - | - | - | - | - | - | - | - | - |
| 14 | - | - | - | - | - | 1 | - | - | 476 | - | - | - | - | - | - | - |
| 15 | - | - | - | - | 1 | - | - | - | - | 352 | - | - | - | - | - | - |
| 16 | - | - | - | - | - | - | - | - | - | - | 353 | - | 1 | - | 4 | 1 |
| 17 | - | - | - | - | - | - | - | - | - | - | - | - | - | - | - | - |
| 18 | - | - | - | - | - | - | - | - | - | 1 | - | - | - | 1 | - | 1 |
| 19 | - | - | - | - | - | - | - | - | - | - | - | - | - | 1 | - | - |
| 20 | - | - | - | - | - | - | - | - | - | - | 3 | - | - | - | - | 252 |

Table S3: Confusion matrix for clustering of tumor samples in the designated mRNA clusters. Cluster 2 mainly composed of tumor samples with a squamous type. The 85 BLCA samples that cluster together with these samples were designated squamous-like in the current study

| Entity/  Cluster | 1 | 2 | 3 | 4 | 5 | 6 | 7 | 8 | 9 | 10 | 11 | 12 | 13 | 14 | 15 | 16 | 17 | 18 | 19 | 20 |
| --- | --- | --- | --- | --- | --- | --- | --- | --- | --- | --- | --- | --- | --- | --- | --- | --- | --- | --- | --- | --- |
| BLCA | 290 | 85 | 18 | 3 | 6 | 4 | 1 | 1 | - | - | - | - | - | - | - | - | - | - | - | - |
| BRCA | - | 3 | 8 | - | - | 2 | - | - | 895 | 182 | 1 | 4 | - | - | - | - | - | - | - | - |
| COAD | - | - | 2 | - | - | - | 458 | - | - | - | - | - | - | - | - | - | - | - | - | - |
| GBM | - | - | 156 | - | - | - | - | - | - | - | - | - | - | - | - | - | - | - | - | - |
| HNSC | - | 511 | 6 | 1 | - | - | - | - | - | - | 1 | - | 1 | - | - | - | - | - | - | - |
| KIRC | - | - | 4 | - | - | - | - | - | - | - | - | - | - | 529 | - | - | - | - | - | - |
| LAML | - | - |  | - | - | 173 | - | - | - | - | - | - | - | - | - | - | - | - | - | - |
| LUAD | 1 | 9 | 3 | 1 | - | 1 | - | 3 | - | - | - | - | - | 1 | 491 | 1 | 1 | 2 | 1 | - |
| LUSC | 3 | 435 | 9 | 4 | - | 2 | - | 12 | - | - | - | - | - | - | 37 | - | - | - | - | - |
| OV | - | - | 5 | - | - | - | - | - | - | - | - | - | - | 1 | - | 2 | - | 1 | - | 296 |
| UCEC | - | - | - | - | - | - | 166 | - | - | - | - | - | - | - | - | - | - | - | - | - |
| READ | - | - | 10 | 3 | - | 1 | 2 | 1 | - | - | - | - | 2 | - | - | 514 | - | - | - | 12 |

Table S4: Results of pan-can expression based consensus clustering. Comparison of our K20_PanCan clusters with the K16_PanCan clusters of Hoadley et al. [1]

| Sample | Our Cluster (k=20) | TCGA Pan-Cancer Cluster (k=16) | Batch | Type |
| --- | --- | --- | --- | --- |
| TCGA-02-0047-01A-01R-1849-01 | 3 | 6 | 1 | Primary solid Tumor |
| TCGA-02-0055-01A-01R-1849-01 | 3 | 6 | 1 | Primary solid Tumor |
| TCGA-02-2483-01A-01R-1849-01 | 3 | 6 | 26 | Primary solid Tumor |
| TCGA-02-2485-01A-01R-1849-01 | 3 | 6 | 26 | Primary solid Tumor |
| TCGA-02-2486-01A-01R-1849-01 | 3 | 6 | 26 | Primary solid Tumor |
| TCGA-04-1348-01A-01R-1565-13 | 20 | 16 | 13 | Primary solid Tumor |
| TCGA-04-1357-01A-01R-1565-13 | 20 | 16 | 13 | Primary solid Tumor |
| TCGA-04-1362-01A-01R-1565-13 | 20 | 16 | 13 | Primary solid Tumor |
| TCGA-04-1364-01A-01R-1565-13 | 20 | 16 | 13 | Primary solid Tumor |
| TCGA-04-1365-01A-01R-1565-13 | 20 | 16 | 13 | Primary solid Tumor |
| TCGA-04-1514-01A-01R-1566-13 | 20 | 16 | 15 | Primary solid Tumor |
| TCGA-04-1519-01A-01R-1565-13 | 20 | 16 | 17 | Primary solid Tumor |
| TCGA-05-4244-01A-01R-1107-07 | 15 | 10 | 52 | Primary solid Tumor |
| TCGA-05-4249-01A-01R-1107-07 | 15 | 10 | 52 | Primary solid Tumor |
| TCGA-05-4250-01A-01R-1107-07 | 15 | 10 | 52 | Primary solid Tumor |
| TCGA-05-4382-01A-01R-1206-07 | 15 | 10 | 58 | Primary solid Tumor |
| TCGA-05-4384-01A-01R-1755-07 | 15 | 10 | 119 | Primary solid Tumor |
| TCGA-05-4389-01A-01R-1206-07 | 15 | 10 | 58 | Primary solid Tumor |
| TCGA-05-4390-01A-02R-1755-07 | 15 | 10 | 119 | Primary solid Tumor |
| TCGA-05-4395-01A-01R-1206-07 | 15 | 10 | 58 | Primary solid Tumor |
| TCGA-05-4396-01A-21R-1858-07 | 15 | 10 | 144 | Primary solid Tumor |
| TCGA-05-4397-01A-01R-1206-07 | 15 | 10 | 58 | Primary solid Tumor |
| TCGA-05-4398-01A-01R-1206-07 | 15 | 10 | 58 | Primary solid Tumor |
| TCGA-05-4402-01A-01R-1206-07 | 15 | 10 | 58 | Primary solid Tumor |
| TCGA-05-4403-01A-01R-1206-07 | 15 | 10 | 58 | Primary solid Tumor |
| TCGA-05-4405-01A-21R-1858-07 | 15 | 10 | 144 | Primary solid Tumor |
| TCGA-05-4410-01A-21R-1858-07 | 15 | 10 | 144 | Primary solid Tumor |
| TCGA-05-4415-01A-22R-1858-07 | 15 | 10 | 144 | Primary solid Tumor |
| TCGA-05-4417-01A-22R-1858-07 | 15 | 10 | 144 | Primary solid Tumor |
| TCGA-05-4418-01A-01R-1206-07 | 15 | 10 | 58 | Primary solid Tumor |
| TCGA-05-4420-01A-01R-1206-07 | 15 | 10 | 58 | Primary solid Tumor |
| TCGA-05-4422-01A-01R-1206-07 | 15 | 10 | 58 | Primary solid Tumor |
| TCGA-05-4424-01A-22R-1858-07 | 15 | 10 | 144 | Primary solid Tumor |
| TCGA-05-4425-01A-01R-1755-07 | 15 | 10 | 119 | Primary solid Tumor |
| TCGA-05-4426-01A-01R-1206-07 | 15 | 10 | 58 | Primary solid Tumor |
| TCGA-05-4427-01A-21R-1858-07 | 15 | 10 | 144 | Primary solid Tumor |
| TCGA-05-4430-01A-02R-1206-07 | 15 | 10 | 58 | Primary solid Tumor |
| TCGA-05-4432-01A-01R-1206-07 | 15 | 10 | 58 | Primary solid Tumor |
| TCGA-05-4433-01A-22R-1858-07 | 15 | 10 | 144 | Primary solid Tumor |
| TCGA-05-4434-01A-01R-1206-07 | 15 | 10 | 58 | Primary solid Tumor |
| TCGA-05-5420-01A-01R-1628-07 | 15 | 10 | 84 | Primary solid Tumor |
| TCGA-05-5423-01A-01R-1628-07 | 15 | 10 | 84 | Primary solid Tumor |
| TCGA-05-5425-01A-02R-1628-07 | 15 | 10 | 84 | Primary solid Tumor |
| TCGA-05-5428-01A-01R-1628-07 | 15 | 10 | 84 | Primary solid Tumor |
| TCGA-05-5429-01A-01R-1628-07 | 15 | 10 | 84 | Primary solid Tumor |
| TCGA-05-5715-01A-01R-1628-07 | 15 | 10 | 84 | Primary solid Tumor |
| TCGA-06-0125-01A-01R-1849-01 | 3 | 6 | 3 | Primary solid Tumor |
| TCGA-06-0129-01A-01R-1849-01 | 3 | 6 | 3 | Primary solid Tumor |
| TCGA-06-0130-01A-01R-1849-01 | 3 | 6 | 3 | Primary solid Tumor |
| TCGA-06-0132-01A-02R-1849-01 | 3 | 6 | 4 | Primary solid Tumor |
| TCGA-06-0138-01A-02R-1849-01 | 3 | 6 | 4 | Primary solid Tumor |
| TCGA-06-0141-01A-01R-1849-01 | 3 | 6 | 3 | Primary solid Tumor |
| TCGA-06-0156-01A-02R-1849-01 | 3 | 6 | 4 | Primary solid Tumor |
| TCGA-06-0156-01A-03R-1849-01 | 3 | 6 | 4 | Primary solid Tumor |
| TCGA-06-0157-01A-01R-1849-01 | 3 | 6 | 4 | Primary solid Tumor |
| TCGA-06-0158-01A-01R-1849-01 | 3 | 6 | 4 | Primary solid Tumor |
| TCGA-06-0168-01A-01R-1849-01 | 3 | 6 | 4 | Primary solid Tumor |
| TCGA-06-0174-01A-01R-1849-01 | 3 | 6 | 4 | Primary solid Tumor |
| TCGA-06-0178-01A-01R-1849-01 | 3 | 6 | 4 | Primary solid Tumor |
| TCGA-06-0184-01A-01R-1849-01 | 3 | 6 | 4 | Primary solid Tumor |
| TCGA-06-0187-01A-01R-1849-01 | 3 | 6 | 4 | Primary solid Tumor |
| TCGA-06-0190-01A-01R-1849-01 | 3 | 6 | 4 | Primary solid Tumor |
| TCGA-06-0210-01A-01R-1849-01 | 3 | 6 | 4 | Primary solid Tumor |
| TCGA-06-0211-01A-01R-1849-01 | 3 | 6 | 4 | Primary solid Tumor |
| TCGA-06-0211-01B-01R-1849-01 | 3 | 6 | 4 | Primary solid Tumor |
| TCGA-06-0219-01A-01R-1849-01 | 3 | 6 | 4 | Primary solid Tumor |
| TCGA-06-0238-01A-02R-1849-01 | 3 | 6 | 7 | Primary solid Tumor |
| TCGA-06-0644-01A-02R-1849-01 | 3 | 6 | 7 | Primary solid Tumor |
| TCGA-06-0645-01A-01R-1849-01 | 3 | 6 | 7 | Primary solid Tumor |
| TCGA-06-0646-01A-01R-1849-01 | 3 | 6 | 7 | Primary solid Tumor |
| TCGA-06-0649-01B-01R-1849-01 | 3 | 6 | 8 | Primary solid Tumor |
| TCGA-06-0686-01A-01R-1849-01 | 3 | 6 | 8 | Primary solid Tumor |
| TCGA-06-0743-01A-01R-1849-01 | 3 | 6 | 8 | Primary solid Tumor |
| TCGA-06-0744-01A-01R-1849-01 | 3 | 6 | 8 | Primary solid Tumor |
| TCGA-06-0745-01A-01R-1849-01 | 3 | 6 | 8 | Primary solid Tumor |
| TCGA-06-0747-01A-01R-1849-01 | 3 | 6 | 8 | Primary solid Tumor |
| TCGA-06-0749-01A-01R-1849-01 | 3 | 6 | 8 | Primary solid Tumor |
| TCGA-06-0750-01A-01R-1849-01 | 3 | 6 | 8 | Primary solid Tumor |
| TCGA-06-0878-01A-01R-1849-01 | 3 | 6 | 10 | Primary solid Tumor |
| TCGA-06-0882-01A-01R-1849-01 | 3 | 6 | 10 | Primary solid Tumor |
| TCGA-06-1804-01A-01R-1849-01 | 3 | 6 | 111 | Primary solid Tumor |
| TCGA-06-2557-01A-01R-1849-01 | 3 | 6 | 26 | Primary solid Tumor |
| TCGA-06-2558-01A-01R-1849-01 | 3 | 6 | 26 | Primary solid Tumor |
| TCGA-06-2559-01A-01R-1849-01 | 3 | 6 | 26 | Primary solid Tumor |
| TCGA-06-2561-01A-02R-1849-01 | 3 | 6 | 26 | Primary solid Tumor |
| TCGA-06-2562-01A-01R-1849-01 | 3 | 6 | 26 | Primary solid Tumor |
| TCGA-06-2563-01A-01R-1849-01 | 3 | 6 | 26 | Primary solid Tumor |
| TCGA-06-2564-01A-01R-1849-01 | 3 | 6 | 26 | Primary solid Tumor |
| TCGA-06-2565-01A-01R-1849-01 | 3 | 6 | 26 | Primary solid Tumor |
| TCGA-06-2567-01A-01R-1849-01 | 3 | 6 | 26 | Primary solid Tumor |
| TCGA-06-2569-01A-01R-1849-01 | 3 | 6 | 26 | Primary solid Tumor |
| TCGA-06-2570-01A-01R-1849-01 | 3 | 6 | 26 | Primary solid Tumor |
| TCGA-06-5408-01A-01R-1849-01 | 3 | 6 | 111 | Primary solid Tumor |
| TCGA-06-5410-01A-01R-1849-01 | 3 | 6 | 111 | Primary solid Tumor |
| TCGA-06-5411-01A-01R-1849-01 | 3 | 6 | 111 | Primary solid Tumor |
| TCGA-06-5412-01A-01R-1849-01 | 3 | 6 | 111 | Primary solid Tumor |
| TCGA-06-5413-01A-01R-1849-01 | 3 | 6 | 111 | Primary solid Tumor |
| TCGA-06-5414-01A-01R-1849-01 | 3 | 6 | 79 | Primary solid Tumor |
| TCGA-06-5415-01A-01R-1849-01 | 3 | 6 | 79 | Primary solid Tumor |
| TCGA-06-5416-01A-01R-1849-01 | 3 | 6 | 79 | Primary solid Tumor |
| TCGA-06-5417-01A-01R-1849-01 | 3 | 6 | 79 | Primary solid Tumor |
| TCGA-06-5418-01A-01R-1849-01 | 3 | 6 | 79 | Primary solid Tumor |
| TCGA-06-5856-01A-01R-1849-01 | 3 | 6 | 111 | Primary solid Tumor |
| TCGA-06-5858-01A-01R-1849-01 | 3 | 6 | 111 | Primary solid Tumor |
| TCGA-06-5859-01A-01R-1849-01 | 3 | 6 | 111 | Primary solid Tumor |
| TCGA-08-0386-01A-01R-1849-01 | 3 | 6 | 7 | Primary solid Tumor |
| TCGA-09-0364-01A-02R-1564-13 | 20 | 16 | 9 | Primary solid Tumor |
| TCGA-09-0366-01A-01R-1564-13 | 20 | 16 | 9 | Primary solid Tumor |
| TCGA-09-0367-01A-01R-1564-13 | 20 | 16 | 9 | Primary solid Tumor |
| TCGA-09-0369-01A-01R-1564-13 | 3 | 16 | 9 | Primary solid Tumor |
| TCGA-09-1659-01B-01R-1564-13 | 20 | NA | 17 | Primary solid Tumor |
| TCGA-09-1662-01A-01R-1566-13 | 20 | 16 | 17 | Primary solid Tumor |
| TCGA-09-1666-01A-01R-1566-13 | 20 | 16 | 17 | Primary solid Tumor |
| TCGA-09-1667-01C-01R-1566-13 | 20 | 16 | 17 | Primary solid Tumor |
| TCGA-09-1668-01B-01R-1566-13 | 20 | 16 | 17 | Primary solid Tumor |
| TCGA-09-1669-01A-01R-1566-13 | 20 | 16 | 17 | Primary solid Tumor |
| TCGA-09-1670-01A-01R-1566-13 | 20 | 16 | 18 | Primary solid Tumor |
| TCGA-09-1673-01A-01R-1566-13 | 20 | 16 | 18 | Primary solid Tumor |
| TCGA-09-1674-01A-01R-1566-13 | 20 | 16 | 18 | Primary solid Tumor |
| TCGA-09-2044-01B-01R-1568-13 | 20 | 16 | 24 | Primary solid Tumor |
| TCGA-09-2045-01A-01R-1568-13 | 20 | 16 | 24 | Primary solid Tumor |
| TCGA-09-2048-01A-01R-1568-13 | 20 | 16 | 24 | Primary solid Tumor |
| TCGA-09-2051-01A-01R-1568-13 | 20 | 16 | 24 | Primary solid Tumor |
| TCGA-09-2054-01A-01R-1568-13 | 3 | 16 | 22 | Primary solid Tumor |
| TCGA-09-2056-01B-01R-1568-13 | 20 | 16 | 22 | Primary solid Tumor |
| TCGA-10-0928-01A-02R-1564-13 | 20 | 16 | 11 | Primary solid Tumor |
| TCGA-10-0936-01A-01R-1564-13 | 20 | 16 | 11 | Primary solid Tumor |
| TCGA-12-0616-01A-01R-1849-01 | 3 | 6 | 7 | Primary solid Tumor |
| TCGA-12-0618-01A-01R-1849-01 | 3 | 6 | 7 | Primary solid Tumor |
| TCGA-12-0619-01A-01R-1849-01 | 3 | 6 | 7 | Primary solid Tumor |
| TCGA-12-0821-01A-01R-1849-01 | 3 | 6 | 10 | Primary solid Tumor |
| TCGA-12-1597-01B-01R-1849-01 | 3 | 6 | 38 | Primary solid Tumor |
| TCGA-12-3650-01A-01R-1849-01 | 3 | 6 | 38 | Primary solid Tumor |
| TCGA-12-3652-01A-01R-1849-01 | 3 | 6 | 38 | Primary solid Tumor |
| TCGA-12-3653-01A-01R-1849-01 | 3 | 6 | 38 | Primary solid Tumor |
| TCGA-12-5295-01A-01R-1849-01 | 3 | 6 | 79 | Primary solid Tumor |
| TCGA-12-5299-01A-02R-1849-01 | 3 | 6 | 79 | Primary solid Tumor |
| TCGA-13-0730-01A-01R-1564-13 | 3 | 16 | 9 | Primary solid Tumor |
| TCGA-13-0799-01A-01R-1564-13 | 20 | 16 | 9 | Primary solid Tumor |
| TCGA-13-0800-01A-01R-1564-13 | 20 | 16 | 9 | Primary solid Tumor |
| TCGA-13-0801-01A-01R-1564-13 | 20 | 16 | 9 | Primary solid Tumor |
| TCGA-13-0890-01A-01R-1564-13 | 20 | 16 | 11 | Primary solid Tumor |
| TCGA-13-0893-01B-01R-1565-13 | 20 | 16 | 13 | Primary solid Tumor |
| TCGA-13-0897-01A-01R-1564-13 | 20 | 16 | 11 | Primary solid Tumor |
| TCGA-13-0899-01A-01R-1564-13 | 20 | 16 | 11 | Primary solid Tumor |
| TCGA-13-0913-01A-01R-1564-13 | 20 | 16 | 11 | Primary solid Tumor |
| TCGA-13-0916-01A-01R-1564-13 | 20 | 16 | 11 | Primary solid Tumor |
| TCGA-13-0920-01A-01R-1564-13 | 20 | 16 | 11 | Primary solid Tumor |
| TCGA-13-0924-01A-01R-1564-13 | 20 | 16 | 11 | Primary solid Tumor |
| TCGA-13-1403-01A-01R-1565-13 | 20 | 16 | 13 | Primary solid Tumor |
| TCGA-13-1405-01A-01R-1565-13 | 20 | 16 | 13 | Primary solid Tumor |
| TCGA-13-1410-01A-01R-1565-13 | 20 | 16 | 13 | Primary solid Tumor |
| TCGA-13-1411-01A-01R-1565-13 | 20 | NA | 13 | Primary solid Tumor |
| TCGA-13-1481-01A-01R-1565-13 | 20 | 16 | 14 | Primary solid Tumor |
| TCGA-13-1497-01A-01R-1565-13 | 20 | 16 | 14 | Primary solid Tumor |
| TCGA-13-1498-01A-01R-1565-13 | 20 | 16 | 14 | Primary solid Tumor |
| TCGA-13-1505-01A-01R-1565-13 | 20 | 16 | 14 | Primary solid Tumor |
| TCGA-13-1506-01A-01R-1565-13 | 20 | 16 | 14 | Primary solid Tumor |
| TCGA-13-1507-01A-01R-1565-13 | 20 | 16 | 14 | Primary solid Tumor |
| TCGA-13-1511-01A-01R-1565-13 | 20 | 16 | 14 | Primary solid Tumor |
| TCGA-13-1512-01A-01R-1565-13 | 20 | 16 | 14 | Primary solid Tumor |
| TCGA-13-2060-01A-01R-1568-13 | 20 | 16 | 24 | Primary solid Tumor |
| TCGA-13-A5FT-01A-11R-A406-31 | 20 | NA | 409 | Primary solid Tumor |
| TCGA-14-0781-01B-01R-1849-01 | 3 | 6 | 111 | Primary solid Tumor |
| TCGA-14-0787-01A-01R-1849-01 | 3 | 6 | 10 | Primary solid Tumor |
| TCGA-14-0789-01A-01R-1849-01 | 3 | 6 | 10 | Primary solid Tumor |
| TCGA-14-0790-01B-01R-1849-01 | 3 | 6 | 26 | Primary solid Tumor |
| TCGA-14-0817-01A-01R-1849-01 | 3 | 6 | 10 | Primary solid Tumor |
| TCGA-14-0871-01A-01R-1849-01 | 3 | NA | 10 | Primary solid Tumor |
| TCGA-14-1034-01A-01R-1849-01 | 3 | 6 | 16 | Primary solid Tumor |
| TCGA-14-1823-01A-01R-1849-01 | 3 | 6 | 20 | Primary solid Tumor |
| TCGA-14-1825-01A-01R-1850-01 | 3 | 6 | 20 | Primary solid Tumor |
| TCGA-14-1829-01A-01R-1850-01 | 3 | 6 | 20 | Primary solid Tumor |
| TCGA-14-2554-01A-01R-1850-01 | 3 | 6 | 26 | Primary solid Tumor |
| TCGA-15-0742-01A-01R-1850-01 | 3 | 6 | 8 | Primary solid Tumor |
| TCGA-15-1444-01A-02R-1850-01 | 3 | 6 | 111 | Primary solid Tumor |
| TCGA-16-0846-01A-01R-1850-01 | 3 | 6 | 10 | Primary solid Tumor |
| TCGA-16-1045-01B-01R-1850-01 | 3 | 6 | 16 | Primary solid Tumor |
| TCGA-18-3406-01A-01R-0980-07 | 2 | 4 | 39 | Primary solid Tumor |
| TCGA-18-3407-01A-01R-0980-07 | 2 | 4 | 39 | Primary solid Tumor |
| TCGA-18-3408-01A-01R-0980-07 | 2 | 4 | 39 | Primary solid Tumor |
| TCGA-18-3409-01A-01R-0980-07 | 2 | 4 | 39 | Primary solid Tumor |
| TCGA-18-3410-01A-01R-0980-07 | 2 | 4 | 39 | Primary solid Tumor |
| TCGA-18-3411-01A-01R-0980-07 | 2 | 4 | 39 | Primary solid Tumor |
| TCGA-18-3412-01A-01R-0980-07 | 2 | 4 | 39 | Primary solid Tumor |
| TCGA-18-3414-01A-01R-0980-07 | 2 | 4 | 39 | Primary solid Tumor |
| TCGA-18-3415-01A-01R-0980-07 | 2 | 4 | 39 | Primary solid Tumor |
| TCGA-18-3416-01A-01R-0980-07 | 2 | 4 | 39 | Primary solid Tumor |
| TCGA-18-3417-01A-01R-1443-07 | 2 | 4 | 77 | Primary solid Tumor |
| TCGA-18-3419-01A-01R-0980-07 | 2 | 4 | 39 | Primary solid Tumor |
| TCGA-18-3421-01A-01R-0980-07 | 2 | 4 | 39 | Primary solid Tumor |
| TCGA-18-4083-01A-01R-1100-07 | 2 | 4 | 53 | Primary solid Tumor |
| TCGA-18-4086-01A-01R-1100-07 | 2 | 4 | 53 | Primary solid Tumor |
| TCGA-18-4721-01A-01R-1443-07 | 2 | 4 | 77 | Primary solid Tumor |
| TCGA-18-5592-01A-01R-1635-07 | 2 | 4 | 101 | Primary solid Tumor |
| TCGA-18-5595-01A-01R-1635-07 | 2 | 4 | 101 | Primary solid Tumor |
| TCGA-19-1390-01A-01R-1850-01 | 3 | 6 | 38 | Primary solid Tumor |
| TCGA-19-1787-01B-01R-1850-01 | 3 | 6 | 38 | Primary solid Tumor |
| TCGA-19-2619-01A-01R-1850-01 | 3 | 6 | 38 | Primary solid Tumor |
| TCGA-19-2620-01A-01R-1850-01 | 3 | 6 | 38 | Primary solid Tumor |
| TCGA-19-2624-01A-01R-1850-01 | 3 | 6 | 38 | Primary solid Tumor |
| TCGA-19-2625-01A-01R-1850-01 | 3 | 6 | 38 | Primary solid Tumor |
| TCGA-19-2629-01A-01R-1850-01 | 3 | 6 | 38 | Primary solid Tumor |
| TCGA-19-4065-01A-01R-2005-01 | 3 | 6 | 174 | Primary solid Tumor |
| TCGA-19-5960-01A-11R-1850-01 | 3 | 6 | 111 | Primary solid Tumor |
| TCGA-20-1682-01A-01R-1564-13 | 20 | 16 | 18 | Primary solid Tumor |
| TCGA-20-1683-01A-01R-1566-13 | 20 | 16 | 18 | Primary solid Tumor |
| TCGA-20-1684-01A-01R-1566-13 | 20 | 16 | 18 | Primary solid Tumor |
| TCGA-20-1685-01A-01R-1566-13 | 20 | 16 | 18 | Primary solid Tumor |
| TCGA-20-1687-01A-01R-1566-13 | 20 | 16 | 18 | Primary solid Tumor |
| TCGA-21-1070-01A-01R-0692-07 | 2 | 4 | 23 | Primary solid Tumor |
| TCGA-21-1071-01A-01R-0692-07 | 2 | 4 | 23 | Primary solid Tumor |
| TCGA-21-1072-01A-01R-0692-07 | 2 | 4 | 23 | Primary solid Tumor |
| TCGA-21-1075-01A-01R-0692-07 | 2 | 4 | 23 | Primary solid Tumor |
| TCGA-21-1076-01A-01R-0692-07 | 2 | 4 | 23 | Primary solid Tumor |
| TCGA-21-1076-01A-02R-0692-07 | 2 | 4 | 23 | Primary solid Tumor |
| TCGA-21-1077-01A-01R-0692-07 | 2 | 4 | 23 | Primary solid Tumor |
| TCGA-21-1078-01A-01R-0692-07 | 1 | 1 | 23 | Primary solid Tumor |
| TCGA-21-1079-01A-01R-0692-07 | 2 | 4 | 23 | Primary solid Tumor |
| TCGA-21-1080-01A-01R-0692-07 | 2 | 4 | 23 | Primary solid Tumor |
| TCGA-21-1081-01A-01R-0692-07 | 2 | 4 | 23 | Primary solid Tumor |
| TCGA-21-1082-01A-01R-0692-07 | 2 | 4 | 23 | Primary solid Tumor |
| TCGA-21-1083-01A-01R-0692-07 | 8 | 12 | 23 | Primary solid Tumor |
| TCGA-21-5782-01A-01R-1635-07 | 2 | 4 | 101 | Primary solid Tumor |
| TCGA-21-5783-01A-41R-2187-07 | 2 | 4 | 208 | Primary solid Tumor |
| TCGA-21-5784-01A-01R-1635-07 | 2 | 4 | 101 | Primary solid Tumor |
| TCGA-21-5786-01A-01R-1635-07 | 2 | 4 | 101 | Primary solid Tumor |
| TCGA-21-5787-01A-01R-1635-07 | 15 | 10 | 101 | Primary solid Tumor |
| TCGA-21-A5DI-01A-31R-A26W-07 | 2 | NA | 283 | Primary solid Tumor |
| TCGA-22-0940-01A-01R-0692-07 | 2 | 4 | 23 | Primary solid Tumor |
| TCGA-22-0944-01A-01R-0692-07 | 2 | 4 | 23 | Primary solid Tumor |
| TCGA-22-1000-01A-01R-A32Z-07 | 2 | NA | 23 | Primary solid Tumor |
| TCGA-22-1002-01A-01R-0692-07 | 2 | 4 | 23 | Primary solid Tumor |
| TCGA-22-1005-01A-01R-0692-07 | 15 | NA | 23 | Primary solid Tumor |
| TCGA-22-1011-01A-01R-0692-07 | 2 | 4 | 23 | Primary solid Tumor |
| TCGA-22-1012-01A-01R-0692-07 | 2 | 4 | 23 | Primary solid Tumor |
| TCGA-22-1016-01A-01R-0692-07 | 2 | 4 | 23 | Primary solid Tumor |
| TCGA-22-1017-01A-01R-0692-07 | 15 | 10 | 23 | Primary solid Tumor |
| TCGA-22-4591-01A-01R-1201-07 | 2 | 4 | 60 | Primary solid Tumor |
| TCGA-22-4593-01A-21R-1820-07 | 2 | 4 | 140 | Primary solid Tumor |
| TCGA-22-4594-01A-01R-1201-07 | 15 | 10 | 60 | Primary solid Tumor |
| TCGA-22-4595-01A-01R-1201-07 | 2 | 4 | 60 | Primary solid Tumor |
| TCGA-22-4596-01A-01R-1201-07 | 15 | 10 | 60 | Primary solid Tumor |
| TCGA-22-4599-01A-01R-1443-07 | 2 | 4 | 77 | Primary solid Tumor |
| TCGA-22-4601-01A-01R-1443-07 | 2 | 4 | 77 | Primary solid Tumor |
| TCGA-22-4604-01A-01R-1201-07 | 2 | 4 | 60 | Primary solid Tumor |
| TCGA-22-4605-01A-21R-2125-07 | 2 | 4 | 193 | Primary solid Tumor |
| TCGA-22-4607-01A-01R-1201-07 | 2 | 4 | 60 | Primary solid Tumor |
| TCGA-22-4609-01A-21R-2125-07 | 2 | NA | 193 | Primary solid Tumor |
| TCGA-22-4613-01A-01R-1443-07 | 2 | 4 | 77 | Primary solid Tumor |
| TCGA-22-5471-01A-01R-1635-07 | 2 | 4 | 101 | Primary solid Tumor |
| TCGA-22-5472-01A-01R-1635-07 | 2 | 4 | 101 | Primary solid Tumor |
| TCGA-22-5473-01A-01R-1635-07 | 2 | 4 | 101 | Primary solid Tumor |
| TCGA-22-5474-01A-01R-1635-07 | 2 | 4 | 101 | Primary solid Tumor |
| TCGA-22-5477-01A-01R-1635-07 | 2 | 4 | 101 | Primary solid Tumor |
| TCGA-22-5478-01A-01R-1635-07 | 2 | 4 | 101 | Primary solid Tumor |
| TCGA-22-5479-01A-31R-1949-07 | 2 | 4 | 159 | Primary solid Tumor |
| TCGA-22-5480-01A-01R-1635-07 | 2 | 4 | 101 | Primary solid Tumor |
| TCGA-22-5481-01A-31R-1949-07 | 8 | 12 | 159 | Primary solid Tumor |
| TCGA-22-5482-01A-01R-1635-07 | 2 | 4 | 101 | Primary solid Tumor |
| TCGA-22-5483-01A-01R-1820-07 | 4 | 3 | 140 | Primary solid Tumor |
| TCGA-22-5485-01A-01R-1635-07 | 2 | 4 | 101 | Primary solid Tumor |
| TCGA-22-5489-01A-01R-1635-07 | 2 | 4 | 101 | Primary solid Tumor |
| TCGA-22-5491-01A-01R-1635-07 | 2 | 4 | 101 | Primary solid Tumor |
| TCGA-22-5492-01A-01R-1635-07 | 2 | 4 | 101 | Primary solid Tumor |
| TCGA-22-A5C4-01A-12R-A27Q-07 | 2 | NA | 293 | Primary solid Tumor |
| TCGA-23-1023-01A-02R-1564-13 | 20 | 16 | 12 | Primary solid Tumor |
| TCGA-23-1023-01R-01R-1564-13 | 20 | NA | 12 | Recurrent Solid Tumor |
| TCGA-23-1026-01B-01R-1569-13 | 20 | 16 | 12 | Primary solid Tumor |
| TCGA-23-1027-01A-02R-1564-13 | 20 | 16 | 12 | Primary solid Tumor |
| TCGA-23-1029-01B-01R-1567-13 | 20 | 16 | 19 | Primary solid Tumor |
| TCGA-23-1109-01A-01R-1564-13 | 20 | 16 | 12 | Primary solid Tumor |
| TCGA-23-1111-01A-01R-1567-13 | 20 | 16 | 19 | Primary solid Tumor |
| TCGA-23-1114-01B-01R-1566-13 | 20 | 16 | 18 | Primary solid Tumor |
| TCGA-23-1120-01A-02R-1565-13 | 20 | 16 | 12 | Primary solid Tumor |
| TCGA-23-1122-01A-01R-1565-13 | 20 | 16 | 12 | Primary solid Tumor |
| TCGA-23-1123-01A-01R-1565-13 | 20 | 16 | 12 | Primary solid Tumor |
| TCGA-23-1809-01A-01R-1566-13 | 20 | 16 | 18 | Primary solid Tumor |
| TCGA-23-2077-01A-01R-1568-13 | 20 | 16 | 22 | Primary solid Tumor |
| TCGA-23-2078-01A-01R-1568-13 | 20 | NA | 22 | Primary solid Tumor |
| TCGA-23-2081-01A-01R-1568-13 | 20 | 16 | 22 | Primary solid Tumor |
| TCGA-23-2084-01A-02R-1568-13 | 20 | 16 | 22 | Primary solid Tumor |
| TCGA-24-0975-01A-02R-1565-13 | 20 | 16 | 12 | Primary solid Tumor |
| TCGA-24-1103-01A-01R-1565-13 | 20 | 16 | 12 | Primary solid Tumor |
| TCGA-24-1413-01A-01R-1565-13 | 20 | 16 | 13 | Primary solid Tumor |
| TCGA-24-1416-01A-01R-1565-13 | 20 | 16 | 14 | Primary solid Tumor |
| TCGA-24-1417-01A-01R-1565-13 | 20 | 16 | 14 | Primary solid Tumor |
| TCGA-24-1418-01A-01R-1565-13 | 20 | 16 | 14 | Primary solid Tumor |
| TCGA-24-1419-01A-01R-1565-13 | 20 | 16 | 14 | Primary solid Tumor |
| TCGA-24-1423-01A-01R-1565-13 | 20 | 16 | 14 | Primary solid Tumor |
| TCGA-24-1424-01A-01R-1565-13 | 20 | 16 | 14 | Primary solid Tumor |
| TCGA-24-1425-01A-02R-1566-13 | 20 | NA | 15 | Primary solid Tumor |
| TCGA-24-1427-01A-01R-1565-13 | 20 | 16 | 14 | Primary solid Tumor |
| TCGA-24-1428-01A-01R-1564-13 | 20 | 16 | 14 | Primary solid Tumor |
| TCGA-24-1430-01A-01R-1566-13 | 20 | 16 | 14 | Primary solid Tumor |
| TCGA-24-1434-01A-01R-1566-13 | 20 | NA | 14 | Primary solid Tumor |
| TCGA-24-1435-01A-01R-1566-13 | 20 | NA | 14 | Primary solid Tumor |
| TCGA-24-1436-01A-01R-1566-13 | 20 | 16 | 14 | Primary solid Tumor |
| TCGA-24-1463-01A-01R-1566-13 | 20 | NA | 14 | Primary solid Tumor |
| TCGA-24-1467-01A-01R-1566-13 | 20 | 16 | 14 | Primary solid Tumor |
| TCGA-24-1469-01A-01R-1566-13 | 20 | 16 | 15 | Primary solid Tumor |
| TCGA-24-1474-01A-01R-1566-13 | 20 | 16 | 15 | Primary solid Tumor |
| TCGA-24-1544-01A-01R-1566-13 | 20 | 16 | 17 | Primary solid Tumor |
| TCGA-24-1545-01A-01R-1566-13 | 20 | NA | 17 | Primary solid Tumor |
| TCGA-24-1546-01A-01R-1566-13 | 20 | NA | 17 | Primary solid Tumor |
| TCGA-24-1548-01A-01R-1566-13 | 20 | 16 | 17 | Primary solid Tumor |
| TCGA-24-1549-01A-01R-1566-13 | 20 | 16 | 15 | Primary solid Tumor |
| TCGA-24-1550-01A-01R-1566-13 | 3 | 16 | 15 | Primary solid Tumor |
| TCGA-24-1551-01A-01R-1566-13 | 20 | 16 | 15 | Primary solid Tumor |
| TCGA-24-1552-01A-01R-1566-13 | 20 | 16 | 15 | Primary solid Tumor |
| TCGA-24-1553-01A-01R-1566-13 | 20 | 16 | 15 | Primary solid Tumor |
| TCGA-24-1555-01A-01R-1566-13 | 20 | 16 | 15 | Primary solid Tumor |
| TCGA-24-1556-01A-01R-1566-13 | 20 | 16 | 17 | Primary solid Tumor |
| TCGA-24-1557-01A-01R-1566-13 | 20 | 16 | 17 | Primary solid Tumor |
| TCGA-24-1558-01A-01R-1566-13 | 20 | 16 | 17 | Primary solid Tumor |
| TCGA-24-1560-01A-01R-1566-13 | 20 | 16 | 17 | Primary solid Tumor |
| TCGA-24-1562-01A-01R-1566-13 | 20 | 16 | 15 | Primary solid Tumor |
| TCGA-24-1563-01A-01R-1566-13 | 20 | 16 | 15 | Primary solid Tumor |
| TCGA-24-1564-01A-01R-1566-13 | 20 | 16 | 15 | Primary solid Tumor |
| TCGA-24-1567-01A-01R-1566-13 | 20 | 16 | 17 | Primary solid Tumor |
| TCGA-24-1603-01A-01R-1566-13 | 20 | 16 | 15 | Primary solid Tumor |
| TCGA-24-1604-01A-01R-1566-13 | 20 | 16 | 15 | Primary solid Tumor |
| TCGA-24-1616-01A-01R-1566-13 | 20 | 16 | 15 | Primary solid Tumor |
| TCGA-24-1842-01A-01R-1567-13 | 20 | 16 | 19 | Primary solid Tumor |
| TCGA-24-1843-01A-01R-1567-13 | 20 | 16 | 19 | Primary solid Tumor |
| TCGA-24-1844-01A-01R-1567-13 | 20 | 16 | 19 | Primary solid Tumor |
| TCGA-24-1846-01A-01R-1567-13 | 20 | 16 | 19 | Primary solid Tumor |
| TCGA-24-1847-01A-01R-1566-13 | 20 | 16 | 18 | Primary solid Tumor |
| TCGA-24-1849-01A-01R-1567-13 | 20 | NA | 19 | Primary solid Tumor |
| TCGA-24-1850-01A-01R-1567-13 | 20 | 16 | 19 | Primary solid Tumor |
| TCGA-24-1924-01A-01R-1567-13 | 20 | 16 | 21 | Primary solid Tumor |
| TCGA-24-1928-01A-01R-1567-13 | 20 | NA | 21 | Primary solid Tumor |
| TCGA-24-1930-01A-01R-1567-13 | 20 | 16 | 21 | Primary solid Tumor |
| TCGA-24-2019-01A-02R-1568-13 | 20 | 16 | 22 | Primary solid Tumor |
| TCGA-24-2020-01A-01R-1567-13 | 18 | 16 | 21 | Primary solid Tumor |
| TCGA-24-2023-01A-01R-1567-13 | 20 | 16 | 21 | Primary solid Tumor |
| TCGA-24-2024-01A-02R-1568-13 | 20 | 16 | 22 | Primary solid Tumor |
| TCGA-24-2026-01A-01R-1567-13 | 20 | 16 | 21 | Primary solid Tumor |
| TCGA-24-2027-01A-01R-1567-13 | 20 | 16 | 21 | Primary solid Tumor |
| TCGA-24-2033-01A-01R-1568-13 | 20 | 16 | 22 | Primary solid Tumor |
| TCGA-24-2035-01A-01R-1568-13 | 20 | 16 | 22 | Primary solid Tumor |
| TCGA-24-2036-01A-01R-1568-13 | 20 | 16 | 22 | Primary solid Tumor |
| TCGA-24-2038-01A-01R-1568-13 | 20 | 16 | 22 | Primary solid Tumor |
| TCGA-24-2254-01A-01R-1568-13 | 20 | 16 | 22 | Primary solid Tumor |
| TCGA-24-2261-01A-01R-1568-13 | 20 | 16 | 22 | Primary solid Tumor |
| TCGA-24-2262-01A-01R-1568-13 | 20 | 16 | 24 | Primary solid Tumor |
| TCGA-24-2267-01A-01R-1568-13 | 20 | 16 | 24 | Primary solid Tumor |
| TCGA-24-2271-01A-01R-1568-13 | 20 | 16 | 24 | Primary solid Tumor |
| TCGA-24-2281-01A-01R-1568-13 | 20 | 16 | 24 | Primary solid Tumor |
| TCGA-24-2288-01A-01R-1568-13 | 20 | 16 | 24 | Primary solid Tumor |
| TCGA-24-2289-01A-01R-1568-13 | 20 | NA | 24 | Primary solid Tumor |
| TCGA-24-2290-01A-01R-1568-13 | 20 | 16 | 24 | Primary solid Tumor |
| TCGA-24-2293-01A-01R-1568-13 | 20 | NA | 24 | Primary solid Tumor |
| TCGA-24-2297-01A-01R-1568-13 | 20 | 16 | 24 | Primary solid Tumor |
| TCGA-24-2298-01A-01R-1569-13 | 20 | 16 | 24 | Primary solid Tumor |
| TCGA-25-1312-01A-01R-1565-13 | 20 | 16 | 13 | Primary solid Tumor |
| TCGA-25-1313-01A-01R-1565-13 | 20 | 16 | 13 | Primary solid Tumor |
| TCGA-25-1314-01A-01R-1565-13 | 20 | 16 | 13 | Primary solid Tumor |
| TCGA-25-1315-01A-01R-1565-13 | 20 | 16 | 13 | Primary solid Tumor |
| TCGA-25-1316-01A-01R-1565-13 | 20 | 16 | 13 | Primary solid Tumor |
| TCGA-25-1317-01A-01R-1565-13 | 20 | 16 | 13 | Primary solid Tumor |
| TCGA-25-1318-01A-01R-1565-13 | 20 | 16 | 13 | Primary solid Tumor |
| TCGA-25-1319-01A-01R-1565-13 | 20 | 16 | 13 | Primary solid Tumor |
| TCGA-25-1320-01A-01R-1565-13 | 20 | NA | 13 | Primary solid Tumor |
| TCGA-25-1321-01A-01R-1565-13 | 20 | 16 | 13 | Primary solid Tumor |
| TCGA-25-1322-01A-01R-1565-13 | 20 | 16 | 13 | Primary solid Tumor |
| TCGA-25-1323-01A-01R-1565-13 | 20 | 16 | 13 | Primary solid Tumor |
| TCGA-25-1324-01A-01R-1565-13 | 20 | 16 | 13 | Primary solid Tumor |
| TCGA-25-1326-01A-01R-1565-13 | 20 | 16 | 13 | Primary solid Tumor |
| TCGA-25-1328-01A-01R-1565-13 | 20 | 16 | 13 | Primary solid Tumor |
| TCGA-25-1329-01A-01R-1565-13 | 20 | 16 | 13 | Primary solid Tumor |
| TCGA-25-1623-01A-01R-1566-13 | 20 | 16 | 17 | Primary solid Tumor |
| TCGA-25-1625-01A-01R-1566-13 | 20 | 16 | 17 | Primary solid Tumor |
| TCGA-25-1626-01A-01R-1566-13 | 20 | NA | 17 | Primary solid Tumor |
| TCGA-25-1627-01A-01R-1566-13 | 20 | 16 | 17 | Primary solid Tumor |
| TCGA-25-1628-01A-01R-1566-13 | 20 | NA | 17 | Primary solid Tumor |
| TCGA-25-1630-01A-01R-1566-13 | 20 | 16 | 17 | Primary solid Tumor |
| TCGA-25-1631-01A-01R-1569-13 | 20 | 16 | 17 | Primary solid Tumor |
| TCGA-25-1632-01A-01R-1566-13 | 20 | 16 | 17 | Primary solid Tumor |
| TCGA-25-1633-01A-01R-1566-13 | 20 | 16 | 17 | Primary solid Tumor |
| TCGA-25-1634-01A-01R-1566-13 | 20 | 16 | 17 | Primary solid Tumor |
| TCGA-25-1635-01A-01R-1566-13 | 20 | 16 | 17 | Primary solid Tumor |
| TCGA-25-1870-01A-01R-1567-13 | 20 | 16 | 21 | Primary solid Tumor |
| TCGA-25-1871-01A-01R-1567-13 | 20 | 16 | 21 | Primary solid Tumor |
| TCGA-25-2042-01A-01R-1568-13 | 20 | NA | 24 | Primary solid Tumor |
| TCGA-25-2391-01A-01R-1569-13 | 20 | 16 | 24 | Primary solid Tumor |
| TCGA-25-2392-01A-01R-1569-13 | 20 | 16 | 24 | Primary solid Tumor |
| TCGA-25-2393-01A-01R-1569-13 | 20 | 16 | 24 | Primary solid Tumor |
| TCGA-25-2396-01A-01R-1569-13 | 20 | 16 | 24 | Primary solid Tumor |
| TCGA-25-2397-01A-01R-1569-13 | 20 | 16 | 24 | Primary solid Tumor |
| TCGA-25-2398-01A-01R-1569-13 | 20 | NA | 24 | Primary solid Tumor |
| TCGA-25-2399-01A-01R-1569-13 | 20 | 16 | 24 | Primary solid Tumor |
| TCGA-25-2400-01A-01R-1569-13 | 20 | 16 | 24 | Primary solid Tumor |
| TCGA-25-2401-01A-01R-1569-13 | 20 | NA | 24 | Primary solid Tumor |
| TCGA-25-2404-01A-01R-1569-13 | 20 | 16 | 24 | Primary solid Tumor |
| TCGA-25-2409-01A-01R-1569-13 | 20 | 16 | 24 | Primary solid Tumor |
| TCGA-26-1442-01A-01R-1850-01 | 3 | 6 | 111 | Primary solid Tumor |
| TCGA-26-5132-01A-01R-1850-01 | 3 | 6 | 79 | Primary solid Tumor |
| TCGA-26-5133-01A-01R-1850-01 | 3 | 6 | 79 | Primary solid Tumor |
| TCGA-26-5134-01A-01R-1850-01 | 3 | 6 | 79 | Primary solid Tumor |
| TCGA-26-5135-01A-01R-1850-01 | 3 | 6 | 79 | Primary solid Tumor |
| TCGA-26-5136-01B-01R-1850-01 | 3 | 6 | 79 | Primary solid Tumor |
| TCGA-26-5139-01A-01R-1850-01 | 3 | 6 | 79 | Primary solid Tumor |
| TCGA-27-1830-01A-01R-1850-01 | 3 | 6 | 20 | Primary solid Tumor |
| TCGA-27-1831-01A-01R-1850-01 | 3 | 6 | 26 | Primary solid Tumor |
| TCGA-27-1832-01A-01R-1850-01 | 3 | 6 | 20 | Primary solid Tumor |
| TCGA-27-1834-01A-01R-1850-01 | 3 | 6 | 20 | Primary solid Tumor |
| TCGA-27-1835-01A-01R-1850-01 | 3 | 6 | 26 | Primary solid Tumor |
| TCGA-27-1837-01A-01R-1850-01 | 3 | 6 | 26 | Primary solid Tumor |
| TCGA-27-2519-01A-01R-1850-01 | 3 | 6 | 26 | Primary solid Tumor |
| TCGA-27-2521-01A-01R-1850-01 | 3 | 6 | 26 | Primary solid Tumor |
| TCGA-27-2523-01A-01R-1850-01 | 3 | 6 | 26 | Primary solid Tumor |
| TCGA-27-2524-01A-01R-1850-01 | 3 | 6 | 26 | Primary solid Tumor |
| TCGA-27-2526-01A-01R-1850-01 | 3 | 6 | 26 | Primary solid Tumor |
| TCGA-27-2528-01A-01R-1850-01 | 3 | 6 | 26 | Primary solid Tumor |
| TCGA-28-1747-01C-01R-1850-01 | 3 | 6 | 26 | Primary solid Tumor |
| TCGA-28-1753-01A-01R-1850-01 | 3 | 6 | 26 | Primary solid Tumor |
| TCGA-28-2499-01A-01R-1850-01 | 3 | 6 | 26 | Primary solid Tumor |
| TCGA-28-2509-01A-01R-1850-01 | 3 | 6 | 26 | Primary solid Tumor |
| TCGA-28-2510-01A-01R-1850-01 | 3 | 6 | 111 | Primary solid Tumor |
| TCGA-28-2513-01A-01R-1850-01 | 3 | 6 | 26 | Primary solid Tumor |
| TCGA-28-2514-01A-02R-1850-01 | 3 | 6 | 26 | Primary solid Tumor |
| TCGA-28-5204-01A-01R-1850-01 | 3 | 6 | 79 | Primary solid Tumor |
| TCGA-28-5207-01A-01R-1850-01 | 3 | 6 | 79 | Primary solid Tumor |
| TCGA-28-5208-01A-01R-1850-01 | 3 | 6 | 79 | Primary solid Tumor |
| TCGA-28-5209-01A-01R-1850-01 | 3 | 6 | 79 | Primary solid Tumor |
| TCGA-28-5213-01A-01R-1850-01 | 3 | 6 | 79 | Primary solid Tumor |
| TCGA-28-5215-01A-01R-1850-01 | 3 | 6 | 79 | Primary solid Tumor |
| TCGA-28-5216-01A-01R-1850-01 | 3 | 6 | 79 | Primary solid Tumor |
| TCGA-28-5218-01A-01R-1850-01 | 3 | 6 | 79 | Primary solid Tumor |
| TCGA-28-5220-01A-01R-1850-01 | 3 | 6 | 79 | Primary solid Tumor |
| TCGA-29-1688-01A-01R-1566-13 | 20 | 16 | 18 | Primary solid Tumor |
| TCGA-29-1690-01A-01R-1566-13 | 20 | 16 | 18 | Primary solid Tumor |
| TCGA-29-1691-01A-01R-1566-13 | 20 | 16 | 18 | Primary solid Tumor |
| TCGA-29-1693-01A-01R-1567-13 | 20 | 16 | 18 | Primary solid Tumor |
| TCGA-29-1694-01A-01R-1567-13 | 20 | 16 | 18 | Primary solid Tumor |
| TCGA-29-1695-01A-01R-1567-13 | 20 | 16 | 18 | Primary solid Tumor |
| TCGA-29-1696-01A-01R-1567-13 | 20 | 16 | 18 | Primary solid Tumor |
| TCGA-29-1697-01A-01R-1567-13 | 20 | 16 | 18 | Primary solid Tumor |
| TCGA-29-1698-01A-01R-1567-13 | 20 | NA | 18 | Primary solid Tumor |
| TCGA-29-1699-01A-01R-1567-13 | 20 | 16 | 18 | Primary solid Tumor |
| TCGA-29-1701-01A-01R-1567-13 | 20 | 16 | 18 | Primary solid Tumor |
| TCGA-29-1702-01A-01R-1567-13 | 20 | 16 | 18 | Primary solid Tumor |
| TCGA-29-1703-01A-01R-1567-13 | 20 | 16 | 18 | Primary solid Tumor |
| TCGA-29-1705-01A-01R-1567-13 | 20 | 16 | 18 | Primary solid Tumor |
| TCGA-29-1710-01A-02R-1567-13 | 20 | 16 | 18 | Primary solid Tumor |
| TCGA-29-1711-01A-01R-1567-13 | 20 | 16 | 18 | Primary solid Tumor |
| TCGA-29-1761-01A-01R-1567-13 | 20 | 16 | 18 | Primary solid Tumor |
| TCGA-29-1762-01A-01R-1567-13 | 20 | 16 | 18 | Primary solid Tumor |
| TCGA-29-1763-01A-02R-1567-13 | 20 | 16 | 18 | Primary solid Tumor |
| TCGA-29-1770-01A-01R-1567-13 | 20 | 16 | 18 | Primary solid Tumor |
| TCGA-29-1776-01A-01R-1567-13 | 20 | 16 | 19 | Primary solid Tumor |
| TCGA-29-1778-01A-01R-1567-13 | 20 | 16 | 19 | Primary solid Tumor |
| TCGA-29-1781-01A-01R-1567-13 | 20 | 16 | 18 | Primary solid Tumor |
| TCGA-29-1783-01A-01R-1567-13 | 20 | 16 | 18 | Primary solid Tumor |
| TCGA-29-1784-01A-02R-1567-13 | 20 | 16 | 18 | Primary solid Tumor |
| TCGA-29-1785-01A-01R-1567-13 | 20 | 16 | 18 | Primary solid Tumor |
| TCGA-29-2414-01A-02R-1569-13 | 20 | 16 | 24 | Primary solid Tumor |
| TCGA-29-2425-01A-01R-1569-13 | 20 | 16 | 24 | Primary solid Tumor |
| TCGA-29-2427-01A-01R-1569-13 | 20 | 16 | 24 | Primary solid Tumor |
| TCGA-29-2428-01A-01R-1569-13 | 20 | 16 | 24 | Primary solid Tumor |
| TCGA-29-A5NZ-01A-11R-A406-31 | 20 | NA | 409 | Primary solid Tumor |
| TCGA-2E-A9G8-01A-11R-A40A-07 | 20 | NA | 421 | Primary solid Tumor |
| TCGA-2F-A9KO-01A-11R-A38B-07 | 1 | NA | 391 | Primary solid Tumor |
| TCGA-2F-A9KP-01A-11R-A38B-07 | 1 | NA | 391 | Primary solid Tumor |
| TCGA-2F-A9KQ-01A-11R-A38B-07 | 1 | NA | 391 | Primary solid Tumor |
| TCGA-2F-A9KR-01A-11R-A38B-07 | 1 | NA | 391 | Primary solid Tumor |
| TCGA-2F-A9KT-01A-11R-A38B-07 | 1 | NA | 391 | Primary solid Tumor |
| TCGA-2F-A9KW-01A-11R-A38B-07 | 1 | NA | 391 | Primary solid Tumor |
| TCGA-30-1718-01A-01R-1567-13 | 20 | 16 | 18 | Primary solid Tumor |
| TCGA-30-1855-01A-01R-1567-13 | 20 | 16 | 19 | Primary solid Tumor |
| TCGA-30-1857-01A-02R-1569-13 | 20 | NA | 19 | Primary solid Tumor |
| TCGA-30-1860-01A-01R-1568-13 | 20 | 16 | 21 | Primary solid Tumor |
| TCGA-30-1861-01A-01R-1568-13 | 20 | NA | 21 | Primary solid Tumor |
| TCGA-30-1862-01A-02R-1568-13 | 20 | 16 | 21 | Primary solid Tumor |
| TCGA-30-1891-01A-01R-1568-13 | 20 | 16 | 21 | Primary solid Tumor |
| TCGA-31-1944-01A-01R-1568-13 | 20 | 16 | 21 | Primary solid Tumor |
| TCGA-31-1946-01A-01R-1568-13 | 20 | NA | 21 | Primary solid Tumor |
| TCGA-31-1950-01A-01R-1568-13 | 20 | NA | 21 | Primary solid Tumor |
| TCGA-31-1951-01A-01R-1568-13 | 20 | 16 | 21 | Primary solid Tumor |
| TCGA-31-1953-01A-01R-1568-13 | 20 | 16 | 21 | Primary solid Tumor |
| TCGA-31-1956-01A-01R-1568-13 | 20 | 16 | 21 | Primary solid Tumor |
| TCGA-31-1959-01A-01R-1568-13 | 20 | NA | 21 | Primary solid Tumor |
| TCGA-32-1970-01A-01R-1850-01 | 3 | 6 | 26 | Primary solid Tumor |
| TCGA-32-1980-01A-01R-1850-01 | 3 | 6 | 111 | Primary solid Tumor |
| TCGA-32-1982-01A-01R-1850-01 | 3 | 6 | 26 | Primary solid Tumor |
| TCGA-32-2615-01A-01R-1850-01 | 3 | 6 | 38 | Primary solid Tumor |
| TCGA-32-2616-01A-01R-1850-01 | 3 | 6 | 38 | Primary solid Tumor |
| TCGA-32-2632-01A-01R-1850-01 | 3 | 6 | 38 | Primary solid Tumor |
| TCGA-32-2634-01A-01R-1850-01 | 3 | 6 | 38 | Primary solid Tumor |
| TCGA-32-2638-01A-01R-1850-01 | 3 | 6 | 38 | Primary solid Tumor |
| TCGA-32-4213-01A-01R-1850-01 | 3 | 6 | 62 | Primary solid Tumor |
| TCGA-32-5222-01A-01R-1850-01 | 3 | 6 | 79 | Primary solid Tumor |
| TCGA-33-4532-01A-01R-1201-07 | 2 | 4 | 60 | Primary solid Tumor |
| TCGA-33-4533-01A-01R-1201-07 | 3 | 6 | 60 | Primary solid Tumor |
| TCGA-33-4538-01A-01R-1201-07 | 2 | 4 | 60 | Primary solid Tumor |
| TCGA-33-4547-01A-01R-1201-07 | 2 | 4 | 60 | Primary solid Tumor |
| TCGA-33-4566-01A-01R-1443-07 | 15 | 10 | 77 | Primary solid Tumor |
| TCGA-33-4582-01A-01R-1443-07 | 2 | 4 | 77 | Primary solid Tumor |
| TCGA-33-4583-01A-01R-1443-07 | 2 | 4 | 77 | Primary solid Tumor |
| TCGA-33-4586-01A-01R-1443-07 | 2 | 4 | 77 | Primary solid Tumor |
| TCGA-33-4587-01A-11R-2125-07 | 8 | 12 | 193 | Primary solid Tumor |
| TCGA-33-4589-01A-01R-1443-07 | 2 | NA | 77 | Primary solid Tumor |
| TCGA-33-6737-01A-11R-1820-07 | 15 | 10 | 140 | Primary solid Tumor |
| TCGA-33-6738-01A-11R-1949-07 | 2 | 4 | 159 | Primary solid Tumor |
| TCGA-33-A4WN-01A-11R-A262-07 | 2 | NA | 276 | Primary solid Tumor |
| TCGA-33-A5GW-01A-11R-A27Q-07 | 2 | NA | 293 | Primary solid Tumor |
| TCGA-33-AAS8-01A-11R-A405-07 | 2 | NA | 415 | Primary solid Tumor |
| TCGA-33-AASB-01A-11R-A405-07 | 15 | NA | 415 | Primary solid Tumor |
| TCGA-33-AASD-01A-11R-A405-07 | 2 | NA | 415 | Primary solid Tumor |
| TCGA-33-AASI-01A-22R-A405-07 | 2 | NA | 415 | Primary solid Tumor |
| TCGA-33-AASJ-01A-11R-A405-07 | 2 | NA | 415 | Primary solid Tumor |
| TCGA-33-AASL-01A-11R-A405-07 | 2 | NA | 415 | Primary solid Tumor |
| TCGA-34-2596-01A-01R-0851-07 | 2 | 4 | 31 | Primary solid Tumor |
| TCGA-34-2600-01A-01R-0851-07 | 2 | 4 | 31 | Primary solid Tumor |
| TCGA-34-2608-01A-02R-0851-07 | 2 | 4 | 31 | Primary solid Tumor |
| TCGA-34-5231-01A-21R-1820-07 | 2 | 4 | 140 | Primary solid Tumor |
| TCGA-34-5232-01A-21R-1820-07 | 2 | 4 | 140 | Primary solid Tumor |
| TCGA-34-5234-01A-01R-1635-07 | 2 | 4 | 101 | Primary solid Tumor |
| TCGA-34-5236-01A-21R-1820-07 | 2 | 4 | 140 | Primary solid Tumor |
| TCGA-34-5239-01A-21R-1820-07 | 2 | 4 | 140 | Primary solid Tumor |
| TCGA-34-5240-01A-01R-1443-07 | 2 | 4 | 77 | Primary solid Tumor |
| TCGA-34-5241-01A-01R-1443-07 | 2 | 4 | 77 | Primary solid Tumor |
| TCGA-34-5927-01A-11R-1820-07 | 2 | 4 | 140 | Primary solid Tumor |
| TCGA-34-5928-01A-11R-1820-07 | 2 | 4 | 140 | Primary solid Tumor |
| TCGA-34-5929-01A-11R-1820-07 | 2 | 4 | 140 | Primary solid Tumor |
| TCGA-34-7107-01A-11R-1949-07 | 2 | 4 | 159 | Primary solid Tumor |
| TCGA-34-8454-01A-11R-2326-07 | 2 | NA | 233 | Primary solid Tumor |
| TCGA-34-8455-01A-11R-2326-07 | 2 | NA | 233 | Primary solid Tumor |
| TCGA-34-8456-01A-21R-2326-07 | 2 | NA | 233 | Primary solid Tumor |
| TCGA-34-A5IX-01A-12R-A27Q-07 | 2 | NA | 293 | Primary solid Tumor |
| TCGA-35-3615-01A-01R-0946-07 | 15 | 10 | 37 | Primary solid Tumor |
| TCGA-35-4122-01A-01R-1107-07 | 15 | 10 | 52 | Primary solid Tumor |
| TCGA-35-4123-01A-01R-1107-07 | 15 | 10 | 52 | Primary solid Tumor |
| TCGA-35-5375-01A-01R-1628-07 | 2 | 4 | 84 | Primary solid Tumor |
| TCGA-36-1568-01A-01R-1566-13 | 20 | 16 | 17 | Primary solid Tumor |
| TCGA-36-1569-01A-01R-1566-13 | 20 | NA | 17 | Primary solid Tumor |
| TCGA-36-1570-01A-01R-1566-13 | 20 | 16 | 17 | Primary solid Tumor |
| TCGA-36-1571-01A-01R-1566-13 | 20 | 16 | 17 | Primary solid Tumor |
| TCGA-36-1574-01A-01R-1566-13 | 20 | 16 | 17 | Primary solid Tumor |
| TCGA-36-1575-01A-01R-1566-13 | 20 | 16 | 17 | Primary solid Tumor |
| TCGA-36-1576-01A-01R-1566-13 | 20 | NA | 17 | Primary solid Tumor |
| TCGA-36-1577-01A-01R-1566-13 | 20 | 16 | 17 | Primary solid Tumor |
| TCGA-36-1578-01A-01R-1566-13 | 20 | 16 | 17 | Primary solid Tumor |
| TCGA-36-1580-01A-01R-1566-13 | 20 | 16 | 17 | Primary solid Tumor |
| TCGA-36-1581-01A-01R-1566-13 | 20 | 16 | 17 | Primary solid Tumor |
| TCGA-37-3783-01A-01R-1201-07 | 2 | 4 | 60 | Primary solid Tumor |
| TCGA-37-3789-01A-01R-0980-07 | 2 | 4 | 39 | Primary solid Tumor |
| TCGA-37-3792-01A-01R-0980-07 | 15 | 10 | 39 | Primary solid Tumor |
| TCGA-37-4129-01A-01R-1100-07 | 3 | 6 | 53 | Primary solid Tumor |
| TCGA-37-4130-01A-01R-1100-07 | 6 | 13 | 53 | Primary solid Tumor |
| TCGA-37-4132-01A-01R-1100-07 | 4 | 3 | 53 | Primary solid Tumor |
| TCGA-37-4133-01A-01R-1100-07 | 2 | 4 | 53 | Primary solid Tumor |
| TCGA-37-4135-01A-01R-1100-07 | 8 | 12 | 53 | Primary solid Tumor |
| TCGA-37-4141-01A-02R-1100-07 | 4 | 3 | 53 | Primary solid Tumor |
| TCGA-37-5819-01A-01R-1635-07 | 15 | 10 | 101 | Primary solid Tumor |
| TCGA-37-A5EL-01A-11R-A26W-07 | 2 | NA | 283 | Primary solid Tumor |
| TCGA-37-A5EM-01A-21R-A27Q-07 | 2 | NA | 293 | Primary solid Tumor |
| TCGA-37-A5EN-01A-21R-A26W-07 | 2 | NA | 283 | Primary solid Tumor |
| TCGA-38-4625-01A-01R-1206-07 | 15 | 10 | 58 | Primary solid Tumor |
| TCGA-38-4626-01A-01R-1206-07 | 15 | 10 | 58 | Primary solid Tumor |
| TCGA-38-4627-01A-01R-1206-07 | 15 | 10 | 58 | Primary solid Tumor |
| TCGA-38-4628-01A-01R-1206-07 | 15 | 10 | 58 | Primary solid Tumor |
| TCGA-38-4629-01A-02R-1206-07 | 15 | 10 | 58 | Primary solid Tumor |
| TCGA-38-4630-01A-01R-1206-07 | 16 | 11 | 58 | Primary solid Tumor |
| TCGA-38-4631-01A-01R-1755-07 | 15 | 10 | 119 | Primary solid Tumor |
| TCGA-38-4632-01A-01R-1755-07 | 15 | 10 | 119 | Primary solid Tumor |
| TCGA-38-6178-01A-11R-1755-07 | 15 | 10 | 119 | Primary solid Tumor |
| TCGA-38-7271-01A-11R-2039-07 | 15 | 10 | 166 | Primary solid Tumor |
| TCGA-38-A44F-01A-11R-A24H-07 | 15 | NA | 258 | Primary solid Tumor |
| TCGA-39-5011-01A-01R-1443-07 | 15 | 10 | 77 | Primary solid Tumor |
| TCGA-39-5016-01A-01R-1443-07 | 2 | 4 | 77 | Primary solid Tumor |
| TCGA-39-5019-01A-01R-1820-07 | 2 | 4 | 140 | Primary solid Tumor |
| TCGA-39-5021-01A-01R-1443-07 | 2 | 4 | 77 | Primary solid Tumor |
| TCGA-39-5022-01A-21R-1820-07 | 2 | NA | 140 | Primary solid Tumor |
| TCGA-39-5024-01A-21R-1820-07 | 2 | 4 | 140 | Primary solid Tumor |
| TCGA-39-5027-01A-21R-1820-07 | 2 | 4 | 140 | Primary solid Tumor |
| TCGA-39-5028-01A-01R-1443-07 | 2 | 4 | 77 | Primary solid Tumor |
| TCGA-39-5029-01A-01R-1443-07 | 2 | 4 | 77 | Primary solid Tumor |
| TCGA-39-5030-01A-01R-1443-07 | 2 | 4 | 77 | Primary solid Tumor |
| TCGA-39-5031-01A-01R-1443-07 | 2 | 4 | 77 | Primary solid Tumor |
| TCGA-39-5034-01A-01R-1443-07 | 15 | 10 | 77 | Primary solid Tumor |
| TCGA-39-5035-01A-01R-1443-07 | 2 | 4 | 77 | Primary solid Tumor |
| TCGA-39-5036-01A-01R-1443-07 | 2 | 4 | 77 | Primary solid Tumor |
| TCGA-39-5037-01A-01R-1443-07 | 2 | 4 | 77 | Primary solid Tumor |
| TCGA-39-5039-01A-01R-1443-07 | 2 | 4 | 77 | Primary solid Tumor |
| TCGA-39-5040-01A-21R-2125-07 | 1 | NA | 193 | Primary solid Tumor |
| TCGA-3C-AAAU-01A-11R-A41B-07 | 9 | NA | 379 | Primary solid Tumor |
| TCGA-3C-AALI-01A-11R-A41B-07 | 9 | NA | 379 | Primary solid Tumor |
| TCGA-3C-AALJ-01A-31R-A41B-07 | 9 | NA | 379 | Primary solid Tumor |
| TCGA-3C-AALK-01A-11R-A41B-07 | 9 | NA | 379 | Primary solid Tumor |
| TCGA-3L-AA1B-01A-11R-A37K-07 | 7 | NA | 385 | Primary solid Tumor |
| TCGA-3P-A9WA-01A-11R-A406-31 | 20 | NA | 409 | Primary solid Tumor |
| TCGA-3Z-A93Z-01A-11R-A37O-07 | 14 | NA | 387 | Primary solid Tumor |
| TCGA-41-2571-01A-01R-1850-01 | 3 | 6 | 38 | Primary solid Tumor |
| TCGA-41-2572-01A-01R-1850-01 | 3 | 6 | 62 | Primary solid Tumor |
| TCGA-41-3915-01A-01R-1850-01 | 3 | 6 | 62 | Primary solid Tumor |
| TCGA-41-4097-01A-01R-1850-01 | 3 | 6 | 62 | Primary solid Tumor |
| TCGA-41-5651-01A-01R-1850-01 | 3 | 6 | 111 | Primary solid Tumor |
| TCGA-43-2576-01A-01R-A32Z-07 | 15 | NA | 31 | Primary solid Tumor |
| TCGA-43-2578-01A-01R-0851-07 | 4 | 3 | 31 | Primary solid Tumor |
| TCGA-43-2581-01A-01R-0851-07 | 15 | 10 | 31 | Primary solid Tumor |
| TCGA-43-3394-01A-01R-0980-07 | 2 | 4 | 39 | Primary solid Tumor |
| TCGA-43-3920-01A-01R-0980-07 | 2 | 4 | 39 | Primary solid Tumor |
| TCGA-43-5668-01A-01R-1635-07 | 15 | 5 | 101 | Primary solid Tumor |
| TCGA-43-5670-01A-21R-2125-07 | 2 | NA | 193 | Primary solid Tumor |
| TCGA-43-6143-01A-11R-1820-07 | 8 | 12 | 140 | Primary solid Tumor |
| TCGA-43-6647-01A-11R-1820-07 | 2 | 4 | 140 | Primary solid Tumor |
| TCGA-43-6770-01A-11R-1820-07 | 2 | 4 | 140 | Primary solid Tumor |
| TCGA-43-6771-01A-11R-1820-07 | 2 | 4 | 140 | Primary solid Tumor |
| TCGA-43-6773-01A-41R-1949-07 | 2 | NA | 159 | Primary solid Tumor |
| TCGA-43-7656-01A-11R-2125-07 | 8 | 12 | 193 | Primary solid Tumor |
| TCGA-43-7657-01A-31R-2125-07 | 2 | NA | 193 | Primary solid Tumor |
| TCGA-43-7658-01A-11R-2125-07 | 8 | NA | 193 | Primary solid Tumor |
| TCGA-43-8115-01A-11R-2247-07 | 2 | NA | 214 | Primary solid Tumor |
| TCGA-43-8116-01A-11R-2247-07 | 2 | NA | 214 | Primary solid Tumor |
| TCGA-43-8118-01A-11R-2403-07 | 2 | NA | 243 | Primary solid Tumor |
| TCGA-43-A474-01A-11R-A24H-07 | 2 | NA | 259 | Primary solid Tumor |
| TCGA-43-A475-01A-11R-A24H-07 | 2 | NA | 259 | Primary solid Tumor |
| TCGA-43-A56U-01A-11R-A26W-07 | 2 | NA | 283 | Primary solid Tumor |
| TCGA-43-A56V-01A-11R-A26W-07 | 2 | NA | 283 | Primary solid Tumor |
| TCGA-44-2655-01A-01R-0946-07 | 15 | 10 | 37 | Primary solid Tumor |
| TCGA-44-2656-01A-02R-0946-07 | 15 | 10 | 37 | Primary solid Tumor |
| TCGA-44-2657-01A-01R-1107-07 | 15 | 10 | 52 | Primary solid Tumor |
| TCGA-44-2659-01A-01R-0946-07 | 15 | 10 | 37 | Primary solid Tumor |
| TCGA-44-2661-01A-01R-1107-07 | 15 | 10 | 52 | Primary solid Tumor |
| TCGA-44-2662-01A-01R-0946-07 | 15 | 10 | 37 | Primary solid Tumor |
| TCGA-44-2665-01A-01R-0946-07 | 15 | 10 | 37 | Primary solid Tumor |
| TCGA-44-2666-01A-01R-0946-07 | 15 | 10 | 37 | Primary solid Tumor |
| TCGA-44-2668-01A-01R-0946-07 | 15 | 10 | 37 | Primary solid Tumor |
| TCGA-44-3396-01A-01R-1206-07 | 15 | 10 | 58 | Primary solid Tumor |
| TCGA-44-3398-01A-01R-1107-07 | 15 | 10 | 52 | Primary solid Tumor |
| TCGA-44-3918-01A-01R-1107-07 | 15 | 10 | 52 | Primary solid Tumor |
| TCGA-44-3919-01A-02R-1107-07 | 15 | 10 | 52 | Primary solid Tumor |
| TCGA-44-4112-01A-01R-1107-07 | 15 | 10 | 52 | Primary solid Tumor |
| TCGA-44-5643-01A-01R-1628-07 | 2 | 4 | 84 | Primary solid Tumor |
| TCGA-44-5644-01A-21R-2039-07 | 15 | 10 | 166 | Primary solid Tumor |
| TCGA-44-5645-01A-01R-1628-07 | 15 | 10 | 84 | Primary solid Tumor |
| TCGA-44-6145-01A-11R-1755-07 | 15 | 10 | 119 | Primary solid Tumor |
| TCGA-44-6146-01A-11R-1755-07 | 15 | 10 | 119 | Primary solid Tumor |
| TCGA-44-6147-01A-11R-1755-07 | 15 | 10 | 119 | Primary solid Tumor |
| TCGA-44-6148-01A-11R-1755-07 | 15 | 10 | 119 | Primary solid Tumor |
| TCGA-44-6774-01A-21R-1858-07 | 15 | 10 | 144 | Primary solid Tumor |
| TCGA-44-6775-01A-11R-1858-07 | 15 | 10 | 144 | Primary solid Tumor |
| TCGA-44-6776-01A-11R-1858-07 | 15 | 10 | 144 | Primary solid Tumor |
| TCGA-44-6777-01A-11R-1858-07 | 15 | 10 | 144 | Primary solid Tumor |
| TCGA-44-6778-01A-11R-1858-07 | 15 | 10 | 144 | Primary solid Tumor |
| TCGA-44-6779-01A-11R-1858-07 | 15 | 10 | 144 | Primary solid Tumor |
| TCGA-44-7659-01A-11R-2066-07 | 15 | 10 | 183 | Primary solid Tumor |
| TCGA-44-7660-01A-11R-2066-07 | 8 | 4 | 183 | Primary solid Tumor |
| TCGA-44-7661-01A-11R-2066-07 | 15 | 10 | 183 | Primary solid Tumor |
| TCGA-44-7662-01A-11R-2066-07 | 15 | 10 | 183 | Primary solid Tumor |
| TCGA-44-7667-01A-31R-2066-07 | 15 | 10 | 183 | Primary solid Tumor |
| TCGA-44-7669-01A-21R-2066-07 | 15 | 10 | 183 | Primary solid Tumor |
| TCGA-44-7670-01A-11R-2066-07 | 15 | 10 | 183 | Primary solid Tumor |
| TCGA-44-7671-01A-11R-2066-07 | 15 | 10 | 183 | Primary solid Tumor |
| TCGA-44-7672-01A-11R-2066-07 | 15 | 10 | 183 | Primary solid Tumor |
| TCGA-44-8117-01A-11R-2241-07 | 15 | 10 | 213 | Primary solid Tumor |
| TCGA-44-8119-01A-11R-2241-07 | 15 | 10 | 213 | Primary solid Tumor |
| TCGA-44-8120-01A-11R-2241-07 | 15 | 10 | 213 | Primary solid Tumor |
| TCGA-44-A479-01A-31R-A24H-07 | 15 | NA | 258 | Primary solid Tumor |
| TCGA-44-A47A-01A-21R-A24H-07 | 15 | NA | 258 | Primary solid Tumor |
| TCGA-44-A47B-01A-11R-A24H-07 | 15 | NA | 258 | Primary solid Tumor |
| TCGA-44-A47G-01A-21R-A24H-07 | 15 | NA | 258 | Primary solid Tumor |
| TCGA-44-A4SS-01A-11R-A24X-07 | 15 | NA | 264 | Primary solid Tumor |
| TCGA-44-A4SU-01A-11R-A24X-07 | 15 | NA | 264 | Primary solid Tumor |
| TCGA-46-3765-01A-01R-0980-07 | 2 | 4 | 39 | Primary solid Tumor |
| TCGA-46-3766-01A-01R-0980-07 | 2 | 4 | 39 | Primary solid Tumor |
| TCGA-46-3767-01A-01R-0980-07 | 2 | 4 | 39 | Primary solid Tumor |
| TCGA-46-3768-01A-01R-0980-07 | 2 | 4 | 39 | Primary solid Tumor |
| TCGA-46-3769-01A-01R-0980-07 | 15 | 10 | 39 | Primary solid Tumor |
| TCGA-46-6025-01A-11R-1820-07 | 2 | 4 | 140 | Primary solid Tumor |
| TCGA-46-6026-01A-11R-1820-07 | 2 | 4 | 140 | Primary solid Tumor |
| TCGA-49-4486-01A-01R-1206-07 | 15 | 10 | 58 | Primary solid Tumor |
| TCGA-49-4487-01A-21R-1858-07 | 15 | 10 | 144 | Primary solid Tumor |
| TCGA-49-4488-01A-01R-1755-07 | 15 | 10 | 119 | Primary solid Tumor |
| TCGA-49-4490-01A-21R-1858-07 | 15 | 10 | 144 | Primary solid Tumor |
| TCGA-49-4494-01A-01R-1206-07 | 15 | 10 | 58 | Primary solid Tumor |
| TCGA-49-4501-01A-01R-1206-07 | 15 | 10 | 58 | Primary solid Tumor |
| TCGA-49-4505-01A-01R-1206-07 | 15 | 10 | 58 | Primary solid Tumor |
| TCGA-49-4506-01A-01R-1206-07 | 15 | 10 | 58 | Primary solid Tumor |
| TCGA-49-4507-01A-01R-1206-07 | 15 | 10 | 58 | Primary solid Tumor |
| TCGA-49-4510-01A-01R-1206-07 | 15 | 10 | 58 | Primary solid Tumor |
| TCGA-49-4512-01A-21R-1858-07 | 15 | 10 | 144 | Primary solid Tumor |
| TCGA-49-4514-01A-21R-1858-07 | 15 | 10 | 144 | Primary solid Tumor |
| TCGA-49-6742-01A-11R-1858-07 | 15 | 10 | 144 | Primary solid Tumor |
| TCGA-49-6743-01A-11R-1858-07 | 15 | 10 | 144 | Primary solid Tumor |
| TCGA-49-6744-01A-11R-1858-07 | 15 | 10 | 144 | Primary solid Tumor |
| TCGA-49-6745-01A-11R-1858-07 | 15 | 10 | 144 | Primary solid Tumor |
| TCGA-49-6761-01A-31R-1949-07 | 15 | 10 | 160 | Primary solid Tumor |
| TCGA-49-6767-01A-11R-1858-07 | 15 | 10 | 144 | Primary solid Tumor |
| TCGA-49-AAQV-01A-11R-A39D-07 | 15 | NA | 406 | Primary solid Tumor |
| TCGA-49-AAR0-01A-21R-A39D-07 | 15 | NA | 406 | Primary solid Tumor |
| TCGA-49-AAR2-01A-11R-A39D-07 | 15 | NA | 406 | Primary solid Tumor |
| TCGA-49-AAR3-01A-11R-A41B-07 | 15 | NA | 423 | Primary solid Tumor |
| TCGA-49-AAR4-01A-12R-A41B-07 | 15 | NA | 423 | Primary solid Tumor |
| TCGA-49-AAR9-01A-21R-A41B-07 | 17 | NA | 423 | Primary solid Tumor |
| TCGA-49-AARE-01A-11R-A41B-07 | 15 | NA | 423 | Primary solid Tumor |
| TCGA-49-AARN-01A-21R-A41B-07 | 15 | NA | 423 | Primary solid Tumor |
| TCGA-49-AARO-01A-12R-A41B-07 | 15 | NA | 423 | Primary solid Tumor |
| TCGA-49-AARQ-01A-11R-A41B-07 | 15 | NA | 423 | Primary solid Tumor |
| TCGA-49-AARR-01A-11R-A41B-07 | 15 | NA | 423 | Primary solid Tumor |
| TCGA-4B-A93V-01A-11R-A39D-07 | 15 | NA | 406 | Primary solid Tumor |
| TCGA-4E-A92E-01A-11R-A37O-07 | 16 | NA | 381 | Primary solid Tumor |
| TCGA-4H-AAAK-01A-12R-A41B-07 | 9 | NA | 379 | Primary solid Tumor |
| TCGA-4N-A93T-01A-11R-A37K-07 | 7 | NA | 385 | Primary solid Tumor |
| TCGA-4P-AA8J-01A-11R-A39I-07 | 2 | NA | 403 | Primary solid Tumor |
| TCGA-4T-AA8H-01A-11R-A41B-07 | 7 | NA | 422 | Primary solid Tumor |
| TCGA-4Z-AA7M-01A-11R-A39I-07 | 1 | NA | 401 | Primary solid Tumor |
| TCGA-4Z-AA7N-01A-11R-A39I-07 | 1 | NA | 401 | Primary solid Tumor |
| TCGA-4Z-AA7O-01A-31R-A39I-07 | 1 | NA | 401 | Primary solid Tumor |
| TCGA-4Z-AA7Q-01A-11R-A39I-07 | 2 | NA | 401 | Primary solid Tumor |
| TCGA-4Z-AA7R-01A-11R-A39I-07 | 1 | NA | 401 | Primary solid Tumor |
| TCGA-4Z-AA7S-01A-11R-A39I-07 | 1 | NA | 401 | Primary solid Tumor |
| TCGA-4Z-AA7W-01A-11R-A39I-07 | 2 | NA | 401 | Primary solid Tumor |
| TCGA-4Z-AA7Y-01A-11R-A39I-07 | 1 | NA | 401 | Primary solid Tumor |
| TCGA-4Z-AA80-01A-11R-A39I-07 | 1 | NA | 401 | Primary solid Tumor |
| TCGA-4Z-AA81-01A-11R-A39I-07 | 1 | NA | 401 | Primary solid Tumor |
| TCGA-4Z-AA82-01A-11R-A39I-07 | 2 | NA | 401 | Primary solid Tumor |
| TCGA-4Z-AA83-01A-11R-A39I-07 | 1 | NA | 401 | Primary solid Tumor |
| TCGA-4Z-AA84-01A-11R-A39I-07 | 1 | NA | 401 | Primary solid Tumor |
| TCGA-4Z-AA86-01A-11R-A39I-07 | 2 | NA | 401 | Primary solid Tumor |
| TCGA-4Z-AA87-01A-11R-A39I-07 | 1 | NA | 401 | Primary solid Tumor |
| TCGA-4Z-AA89-01A-11R-A39I-07 | 1 | NA | 401 | Primary solid Tumor |
| TCGA-50-5044-01A-21R-1858-07 | 15 | 10 | 144 | Primary solid Tumor |
| TCGA-50-5045-01A-01R-1628-07 | 15 | NA | 84 | Primary solid Tumor |
| TCGA-50-5049-01A-01R-1628-07 | 15 | 10 | 84 | Primary solid Tumor |
| TCGA-50-5051-01A-21R-1858-07 | 15 | 10 | 144 | Primary solid Tumor |
| TCGA-50-5055-01A-01R-1628-07 | 15 | 10 | 84 | Primary solid Tumor |
| TCGA-50-5066-01A-01R-1628-07 | 15 | 10 | 84 | Primary solid Tumor |
| TCGA-50-5068-01A-01R-1628-07 | 15 | 10 | 84 | Primary solid Tumor |
| TCGA-50-5072-01A-21R-1858-07 | 15 | 10 | 144 | Primary solid Tumor |
| TCGA-50-5930-01A-11R-1755-07 | 15 | NA | 119 | Primary solid Tumor |
| TCGA-50-5931-01A-11R-1755-07 | 8 | 12 | 119 | Primary solid Tumor |
| TCGA-50-5932-01A-11R-1755-07 | 15 | 10 | 119 | Primary solid Tumor |
| TCGA-50-5933-01A-11R-1755-07 | 15 | 10 | 119 | Primary solid Tumor |
| TCGA-50-5935-01A-11R-1755-07 | 15 | 10 | 119 | Primary solid Tumor |
| TCGA-50-5936-01A-11R-1628-07 | 15 | 10 | 84 | Primary solid Tumor |
| TCGA-50-5939-01A-11R-1628-07 | 15 | 10 | 84 | Primary solid Tumor |
| TCGA-50-5941-01A-11R-1755-07 | 15 | 10 | 119 | Primary solid Tumor |
| TCGA-50-5942-01A-21R-1755-07 | 15 | 10 | 119 | Primary solid Tumor |
| TCGA-50-5944-01A-11R-1755-07 | 15 | 10 | 119 | Primary solid Tumor |
| TCGA-50-5946-01A-11R-1755-07 | 15 | 10 | 119 | Primary solid Tumor |
| TCGA-50-6590-01A-12R-1858-07 | 15 | 10 | 144 | Primary solid Tumor |
| TCGA-50-6591-01A-11R-1755-07 | 3 | 6 | 119 | Primary solid Tumor |
| TCGA-50-6592-01A-11R-1755-07 | 15 | 10 | 119 | Primary solid Tumor |
| TCGA-50-6593-01A-11R-1755-07 | 15 | 10 | 119 | Primary solid Tumor |
| TCGA-50-6594-01A-11R-1755-07 | 15 | 10 | 119 | Primary solid Tumor |
| TCGA-50-6595-01A-12R-1858-07 | 2 | 4 | 144 | Primary solid Tumor |
| TCGA-50-6597-01A-11R-1858-07 | 15 | 10 | 144 | Primary solid Tumor |
| TCGA-50-6673-01A-11R-1949-07 | 15 | 10 | 160 | Primary solid Tumor |
| TCGA-50-7109-01A-11R-2039-07 | 15 | 10 | 166 | Primary solid Tumor |
| TCGA-50-8457-01A-11R-2326-07 | 15 | NA | 232 | Primary solid Tumor |
| TCGA-50-8459-01A-11R-2326-07 | 15 | NA | 232 | Primary solid Tumor |
| TCGA-50-8460-01A-11R-2326-07 | 15 | NA | 232 | Primary solid Tumor |
| TCGA-51-4079-01A-01R-1100-07 | 2 | 4 | 53 | Primary solid Tumor |
| TCGA-51-4080-01A-01R-1100-07 | 2 | 4 | 53 | Primary solid Tumor |
| TCGA-51-4081-01A-01R-1100-07 | 2 | 4 | 53 | Primary solid Tumor |
| TCGA-51-6867-01A-11R-2045-07 | 2 | 4 | 181 | Primary solid Tumor |
| TCGA-52-7622-01A-11R-2125-07 | 2 | NA | 193 | Primary solid Tumor |
| TCGA-52-7809-01A-21R-2125-07 | 2 | NA | 193 | Primary solid Tumor |
| TCGA-52-7810-01A-11R-2125-07 | 2 | NA | 193 | Primary solid Tumor |
| TCGA-52-7811-01A-11R-2125-07 | 2 | NA | 193 | Primary solid Tumor |
| TCGA-52-7812-01A-11R-2125-07 | 15 | NA | 193 | Primary solid Tumor |
| TCGA-53-7624-01A-11R-2066-07 | 1 | 1 | 183 | Primary solid Tumor |
| TCGA-53-7626-01A-12R-2066-07 | 15 | 10 | 183 | Primary solid Tumor |
| TCGA-53-7813-01A-11R-2170-07 | 15 | 10 | 196 | Primary solid Tumor |
| TCGA-53-A4EZ-01A-12R-A24X-07 | 15 | NA | 264 | Primary solid Tumor |
| TCGA-55-1592-01A-01R-0946-07 | 15 | 10 | 37 | Primary solid Tumor |
| TCGA-55-1594-01A-01R-0946-07 | 15 | 10 | 37 | Primary solid Tumor |
| TCGA-55-1595-01A-01R-0946-07 | 15 | 10 | 37 | Primary solid Tumor |
| TCGA-55-1596-01A-01R-0946-07 | 15 | 10 | 37 | Primary solid Tumor |
| TCGA-55-5899-01A-11R-1628-07 | 15 | 10 | 84 | Primary solid Tumor |
| TCGA-55-6543-01A-11R-1755-07 | 15 | 10 | 119 | Primary solid Tumor |
| TCGA-55-6642-01A-11R-1858-07 | 15 | 10 | 144 | Primary solid Tumor |
| TCGA-55-6712-01A-11R-1858-07 | 15 | 10 | 144 | Primary solid Tumor |
| TCGA-55-6968-01A-11R-1949-07 | 15 | 10 | 160 | Primary solid Tumor |
| TCGA-55-6969-01A-11R-1949-07 | 15 | 10 | 160 | Primary solid Tumor |
| TCGA-55-6970-01A-11R-1949-07 | 15 | 10 | 160 | Primary solid Tumor |
| TCGA-55-6971-01A-11R-1949-07 | 15 | 10 | 160 | Primary solid Tumor |
| TCGA-55-6972-01A-11R-1949-07 | 15 | 10 | 160 | Primary solid Tumor |
| TCGA-55-6975-01A-11R-1949-07 | 15 | 10 | 160 | Primary solid Tumor |
| TCGA-55-6978-01A-11R-1949-07 | 15 | 10 | 160 | Primary solid Tumor |
| TCGA-55-6979-01A-11R-1949-07 | 15 | 10 | 160 | Primary solid Tumor |
| TCGA-55-6980-01A-11R-1949-07 | 15 | 10 | 160 | Primary solid Tumor |
| TCGA-55-6981-01A-11R-1949-07 | 15 | 10 | 160 | Primary solid Tumor |
| TCGA-55-6982-01A-11R-1949-07 | 15 | 10 | 160 | Primary solid Tumor |
| TCGA-55-6983-01A-11R-1949-07 | 15 | 10 | 160 | Primary solid Tumor |
| TCGA-55-6984-01A-11R-1949-07 | 15 | 10 | 160 | Primary solid Tumor |
| TCGA-55-6985-01A-11R-1949-07 | 15 | 10 | 160 | Primary solid Tumor |
| TCGA-55-6986-01A-11R-1949-07 | 15 | 10 | 160 | Primary solid Tumor |
| TCGA-55-6987-01A-11R-1949-07 | 15 | 10 | 160 | Primary solid Tumor |
| TCGA-55-7227-01A-11R-2039-07 | 15 | 10 | 166 | Primary solid Tumor |
| TCGA-55-7281-01A-11R-2039-07 | 15 | 10 | 166 | Primary solid Tumor |
| TCGA-55-7283-01A-11R-2039-07 | 15 | 10 | 166 | Primary solid Tumor |
| TCGA-55-7284-01B-11R-2241-07 | 15 | 10 | 213 | Primary solid Tumor |
| TCGA-55-7570-01A-11R-2039-07 | 4 | 3 | 166 | Primary solid Tumor |
| TCGA-55-7573-01A-11R-2039-07 | 15 | 10 | 166 | Primary solid Tumor |
| TCGA-55-7574-01A-11R-2039-07 | 15 | 10 | 166 | Primary solid Tumor |
| TCGA-55-7576-01A-11R-2066-07 | 15 | 10 | 183 | Primary solid Tumor |
| TCGA-55-7724-01A-11R-2170-07 | 2 | 4 | 196 | Primary solid Tumor |
| TCGA-55-7725-01A-11R-2170-07 | 15 | 10 | 196 | Primary solid Tumor |
| TCGA-55-7726-01A-11R-2170-07 | 2 | 4 | 196 | Primary solid Tumor |
| TCGA-55-7727-01A-11R-2170-07 | 15 | 10 | 196 | Primary solid Tumor |
| TCGA-55-7728-01A-11R-2187-07 | 15 | 10 | 204 | Primary solid Tumor |
| TCGA-55-7815-01A-11R-2170-07 | 15 | 10 | 196 | Primary solid Tumor |
| TCGA-55-7816-01A-11R-2170-07 | 14 | NA | 196 | Primary solid Tumor |
| TCGA-55-7903-01A-11R-2170-07 | 15 | 10 | 196 | Primary solid Tumor |
| TCGA-55-7907-01A-11R-2170-07 | 15 | 10 | 196 | Primary solid Tumor |
| TCGA-55-7910-01A-11R-2170-07 | 15 | 10 | 196 | Primary solid Tumor |
| TCGA-55-7911-01A-11R-2170-07 | 15 | 10 | 196 | Primary solid Tumor |
| TCGA-55-7913-01B-11R-2241-07 | 15 | 10 | 213 | Primary solid Tumor |
| TCGA-55-7914-01A-11R-2170-07 | 15 | 10 | 196 | Primary solid Tumor |
| TCGA-55-7994-01A-11R-2187-07 | 15 | 10 | 204 | Primary solid Tumor |
| TCGA-55-7995-01A-11R-2187-07 | 15 | 10 | 204 | Primary solid Tumor |
| TCGA-55-8085-01A-11R-2241-07 | 15 | 10 | 213 | Primary solid Tumor |
| TCGA-55-8087-01A-11R-2241-07 | 15 | 10 | 213 | Primary solid Tumor |
| TCGA-55-8089-01A-11R-2241-07 | 15 | 10 | 213 | Primary solid Tumor |
| TCGA-55-8090-01A-11R-2241-07 | 15 | 10 | 213 | Primary solid Tumor |
| TCGA-55-8091-01A-11R-2241-07 | 15 | 10 | 213 | Primary solid Tumor |
| TCGA-55-8092-01A-11R-2241-07 | 15 | 10 | 213 | Primary solid Tumor |
| TCGA-55-8094-01A-11R-2241-07 | 15 | 10 | 213 | Primary solid Tumor |
| TCGA-55-8096-01A-11R-2241-07 | 15 | 10 | 213 | Primary solid Tumor |
| TCGA-55-8097-01A-11R-2241-07 | 15 | 10 | 213 | Primary solid Tumor |
| TCGA-55-8203-01A-11R-2241-07 | 15 | 10 | 213 | Primary solid Tumor |
| TCGA-55-8204-01A-11R-2241-07 | 2 | 4 | 213 | Primary solid Tumor |
| TCGA-55-8205-01A-11R-2241-07 | 15 | 10 | 213 | Primary solid Tumor |
| TCGA-55-8206-01A-11R-2241-07 | 15 | 10 | 213 | Primary solid Tumor |
| TCGA-55-8207-01A-11R-2241-07 | 15 | 10 | 213 | Primary solid Tumor |
| TCGA-55-8208-01A-11R-2241-07 | 15 | 10 | 213 | Primary solid Tumor |
| TCGA-55-8299-01A-11R-2287-07 | 15 | 10 | 222 | Primary solid Tumor |
| TCGA-55-8301-01A-11R-2287-07 | 15 | 10 | 222 | Primary solid Tumor |
| TCGA-55-8302-01A-11R-2326-07 | 15 | NA | 232 | Primary solid Tumor |
| TCGA-55-8505-01A-11R-2403-07 | 15 | NA | 238 | Primary solid Tumor |
| TCGA-55-8506-01A-11R-2403-07 | 15 | NA | 238 | Primary solid Tumor |
| TCGA-55-8507-01A-11R-2403-07 | 15 | NA | 238 | Primary solid Tumor |
| TCGA-55-8508-01A-11R-2403-07 | 15 | NA | 238 | Primary solid Tumor |
| TCGA-55-8510-01A-11R-2403-07 | 15 | NA | 238 | Primary solid Tumor |
| TCGA-55-8511-01A-11R-2403-07 | 15 | NA | 238 | Primary solid Tumor |
| TCGA-55-8512-01A-11R-2403-07 | 15 | NA | 238 | Primary solid Tumor |
| TCGA-55-8513-01A-11R-2403-07 | 15 | NA | 238 | Primary solid Tumor |
| TCGA-55-8514-01A-11R-2403-07 | 15 | NA | 238 | Primary solid Tumor |
| TCGA-55-8614-01A-11R-2403-07 | 2 | NA | 238 | Primary solid Tumor |
| TCGA-55-8615-01A-11R-2403-07 | 15 | NA | 238 | Primary solid Tumor |
| TCGA-55-8616-01A-11R-2403-07 | 15 | NA | 238 | Primary solid Tumor |
| TCGA-55-8619-01A-11R-2403-07 | 15 | NA | 238 | Primary solid Tumor |
| TCGA-55-8620-01A-11R-2403-07 | 15 | NA | 238 | Primary solid Tumor |
| TCGA-55-8621-01A-11R-2403-07 | 15 | NA | 238 | Primary solid Tumor |
| TCGA-55-A48X-01A-11R-A24H-07 | 15 | NA | 258 | Primary solid Tumor |
| TCGA-55-A48Y-01A-11R-A24H-07 | 15 | NA | 258 | Primary solid Tumor |
| TCGA-55-A48Z-01A-12R-A24X-07 | 15 | NA | 264 | Primary solid Tumor |
| TCGA-55-A490-01A-11R-A466-07 | 15 | NA | 258 | Primary solid Tumor |
| TCGA-55-A491-01A-11R-A24H-07 | 15 | NA | 258 | Primary solid Tumor |
| TCGA-55-A492-01A-11R-A24H-07 | 15 | NA | 258 | Primary solid Tumor |
| TCGA-55-A493-01A-11R-A24H-07 | 15 | NA | 258 | Primary solid Tumor |
| TCGA-55-A494-01A-11R-A24X-07 | 15 | NA | 264 | Primary solid Tumor |
| TCGA-55-A4DF-01A-11R-A24H-07 | 15 | NA | 258 | Primary solid Tumor |
| TCGA-55-A4DG-01A-11R-A24H-07 | 15 | NA | 258 | Primary solid Tumor |
| TCGA-55-A57B-01A-12R-A39D-07 | 15 | NA | 406 | Primary solid Tumor |
| TCGA-56-1622-01A-01R-0692-07 | 2 | 4 | 23 | Primary solid Tumor |
| TCGA-56-5897-01A-11R-1635-07 | 2 | 4 | 101 | Primary solid Tumor |
| TCGA-56-5898-01A-11R-1635-07 | 2 | 4 | 101 | Primary solid Tumor |
| TCGA-56-6545-01A-11R-1820-07 | 2 | 4 | 140 | Primary solid Tumor |
| TCGA-56-6546-01A-11R-1820-07 | 3 | 2 | 140 | Primary solid Tumor |
| TCGA-56-7221-01A-11R-2045-07 | 2 | 4 | 181 | Primary solid Tumor |
| TCGA-56-7222-01A-11R-2045-07 | 2 | 4 | 181 | Primary solid Tumor |
| TCGA-56-7223-01A-11R-2045-07 | 3 | 3 | 181 | Primary solid Tumor |
| TCGA-56-7579-01A-11R-2045-07 | 2 | 4 | 181 | Primary solid Tumor |
| TCGA-56-7580-01A-11R-2045-07 | 2 | 4 | 181 | Primary solid Tumor |
| TCGA-56-7582-01A-11R-2045-07 | 2 | 4 | 181 | Primary solid Tumor |
| TCGA-56-7730-01A-11R-2125-07 | 2 | NA | 193 | Primary solid Tumor |
| TCGA-56-7731-01A-11R-2125-07 | 2 | NA | 193 | Primary solid Tumor |
| TCGA-56-7822-01A-11R-2125-07 | 2 | 4 | 193 | Primary solid Tumor |
| TCGA-56-7823-01B-11R-2247-07 | 2 | NA | 214 | Primary solid Tumor |
| TCGA-56-8082-01A-11R-2247-07 | 2 | NA | 214 | Primary solid Tumor |
| TCGA-56-8083-01A-11R-2247-07 | 3 | NA | 214 | Primary solid Tumor |
| TCGA-56-8201-01A-11R-2247-07 | 2 | NA | 214 | Primary solid Tumor |
| TCGA-56-8304-01A-11R-2326-07 | 2 | NA | 233 | Primary solid Tumor |
| TCGA-56-8305-01A-11R-2296-07 | 2 | NA | 225 | Primary solid Tumor |
| TCGA-56-8307-01A-11R-2296-07 | 2 | NA | 225 | Primary solid Tumor |
| TCGA-56-8308-01A-11R-2296-07 | 2 | NA | 225 | Primary solid Tumor |
| TCGA-56-8309-01A-11R-2296-07 | 2 | NA | 225 | Primary solid Tumor |
| TCGA-56-8503-01A-11R-2403-07 | 2 | NA | 243 | Primary solid Tumor |
| TCGA-56-8504-01A-11R-2403-07 | 2 | NA | 243 | Primary solid Tumor |
| TCGA-56-8622-01A-11R-2403-07 | 2 | NA | 243 | Primary solid Tumor |
| TCGA-56-8623-01A-11R-A28V-07 | 15 | NA | 243 | Primary solid Tumor |
| TCGA-56-8624-01A-11R-2403-07 | 2 | NA | 243 | Primary solid Tumor |
| TCGA-56-8625-01A-11R-2403-07 | 2 | NA | 243 | Primary solid Tumor |
| TCGA-56-8626-01A-11R-2403-07 | 2 | NA | 243 | Primary solid Tumor |
| TCGA-56-8628-01A-11R-2403-07 | 2 | NA | 243 | Primary solid Tumor |
| TCGA-56-8629-01A-11R-2403-07 | 2 | NA | 243 | Primary solid Tumor |
| TCGA-56-A49D-01A-11R-A24H-07 | 2 | NA | 259 | Primary solid Tumor |
| TCGA-56-A4BW-01A-11R-A24H-07 | 2 | NA | 259 | Primary solid Tumor |
| TCGA-56-A4BX-01A-11R-A24H-07 | 2 | NA | 259 | Primary solid Tumor |
| TCGA-56-A4BY-01A-11R-A24H-07 | 2 | NA | 259 | Primary solid Tumor |
| TCGA-56-A4ZJ-01A-11R-A262-07 | 2 | NA | 276 | Primary solid Tumor |
| TCGA-56-A4ZK-01A-11R-A262-07 | 2 | NA | 276 | Primary solid Tumor |
| TCGA-56-A5DR-01A-11R-A27Q-07 | 2 | NA | 293 | Primary solid Tumor |
| TCGA-56-A5DS-01A-11R-A27Q-07 | 2 | NA | 293 | Primary solid Tumor |
| TCGA-56-A62T-01A-11R-A405-07 | 2 | NA | 415 | Primary solid Tumor |
| TCGA-57-1582-01A-01R-1566-13 | 20 | 16 | 17 | Primary solid Tumor |
| TCGA-57-1583-01A-01R-1566-13 | 20 | 16 | 17 | Primary solid Tumor |
| TCGA-57-1584-01A-01R-1566-13 | 20 | 16 | 17 | Primary solid Tumor |
| TCGA-57-1585-01A-01R-1566-13 | 20 | NA | 17 | Primary solid Tumor |
| TCGA-57-1993-01A-01R-1568-13 | 20 | 16 | 21 | Primary solid Tumor |
| TCGA-57-1994-01A-01R-1568-13 | 20 | 16 | 21 | Primary solid Tumor |
| TCGA-58-8386-01A-11R-2296-07 | 2 | NA | 225 | Primary solid Tumor |
| TCGA-58-8387-01A-11R-2296-07 | 2 | NA | 225 | Primary solid Tumor |
| TCGA-58-8388-01A-11R-2326-07 | 2 | NA | 233 | Primary solid Tumor |
| TCGA-58-8390-01A-11R-2326-07 | 2 | NA | 233 | Primary solid Tumor |
| TCGA-58-8391-01A-11R-2326-07 | 2 | NA | 233 | Primary solid Tumor |
| TCGA-58-8392-01A-11R-2326-07 | 2 | NA | 233 | Primary solid Tumor |
| TCGA-58-8393-01A-11R-2326-07 | 2 | NA | 233 | Primary solid Tumor |
| TCGA-58-A46J-01A-11R-A24H-07 | 2 | NA | 259 | Primary solid Tumor |
| TCGA-58-A46K-01A-11R-A24H-07 | 2 | NA | 259 | Primary solid Tumor |
| TCGA-58-A46L-01A-11R-A24H-07 | 2 | NA | 259 | Primary solid Tumor |
| TCGA-58-A46M-01A-11R-A24H-07 | 2 | NA | 259 | Primary solid Tumor |
| TCGA-58-A46N-01A-11R-A24H-07 | 2 | NA | 259 | Primary solid Tumor |
| TCGA-59-2348-01A-01R-1569-13 | 20 | 16 | 24 | Primary solid Tumor |
| TCGA-59-2350-01A-01R-1569-13 | 20 | 16 | 24 | Primary solid Tumor |
| TCGA-59-2351-01A-01R-1569-13 | 20 | 16 | 24 | Primary solid Tumor |
| TCGA-59-2352-01A-01R-1569-13 | 20 | 16 | 24 | Primary solid Tumor |
| TCGA-59-2354-01A-01R-1569-13 | 20 | NA | 24 | Primary solid Tumor |
| TCGA-59-2355-01A-01R-1569-13 | 20 | 16 | 24 | Primary solid Tumor |
| TCGA-59-2363-01A-01R-1569-13 | 20 | 16 | 24 | Primary solid Tumor |
| TCGA-59-A5PD-01A-11R-A406-31 | 20 | NA | 409 | Primary solid Tumor |
| TCGA-5B-A90C-01A-11R-A37O-07 | 16 | NA | 381 | Primary solid Tumor |
| TCGA-5L-AAT0-01A-12R-A41B-07 | 9 | NA | 379 | Primary solid Tumor |
| TCGA-5L-AAT1-01A-12R-A41B-07 | 9 | NA | 379 | Primary solid Tumor |
| TCGA-5M-AAT4-01A-11R-A41B-07 | 7 | NA | 422 | Primary solid Tumor |
| TCGA-5M-AAT5-01A-21R-A41B-07 | 7 | NA | 422 | Primary solid Tumor |
| TCGA-5M-AAT6-01A-11R-A41B-07 | 7 | NA | 422 | Primary solid Tumor |
| TCGA-5M-AATA-01A-31R-A41B-07 | 7 | NA | 422 | Primary solid Tumor |
| TCGA-5M-AATE-01A-11R-A41B-07 | 7 | NA | 422 | Primary solid Tumor |
| TCGA-5N-A9KI-01A-31R-A42T-07 | 1 | NA | 433 | Primary solid Tumor |
| TCGA-5N-A9KM-01A-11R-A42T-07 | 1 | NA | 433 | Primary solid Tumor |
| TCGA-5S-A9Q8-01A-11R-A40A-07 | 16 | NA | 421 | Primary solid Tumor |
| TCGA-5T-A9QA-01A-11R-A41B-07 | 9 | NA | 379 | Primary solid Tumor |
| TCGA-5X-AA5U-01A-11R-A406-31 | 20 | NA | 409 | Primary solid Tumor |
| TCGA-60-2695-01A-01R-0851-07 | 8 | 12 | 31 | Primary solid Tumor |
| TCGA-60-2696-01A-01R-0851-07 | 2 | 4 | 31 | Primary solid Tumor |
| TCGA-60-2697-01A-11R-2125-07 | 15 | NA | 193 | Primary solid Tumor |
| TCGA-60-2698-01A-01R-0851-07 | 2 | 4 | 31 | Primary solid Tumor |
| TCGA-60-2703-01A-11R-2045-07 | 2 | 4 | 181 | Primary solid Tumor |
| TCGA-60-2704-01A-11R-2045-07 | 2 | 4 | 181 | Primary solid Tumor |
| TCGA-60-2706-01A-01R-0851-07 | 15 | 10 | 31 | Primary solid Tumor |
| TCGA-60-2707-01A-01R-0851-07 | 2 | 4 | 31 | Primary solid Tumor |
| TCGA-60-2708-01A-01R-0851-07 | 2 | 4 | 31 | Primary solid Tumor |
| TCGA-60-2709-01A-21R-1820-07 | 2 | 4 | 140 | Primary solid Tumor |
| TCGA-60-2710-01A-01R-0851-07 | 2 | 4 | 31 | Primary solid Tumor |
| TCGA-60-2711-01A-01R-0851-07 | 2 | 4 | 31 | Primary solid Tumor |
| TCGA-60-2712-01A-01R-0851-07 | 2 | 4 | 31 | Primary solid Tumor |
| TCGA-60-2713-01A-01R-0851-07 | 2 | 4 | 31 | Primary solid Tumor |
| TCGA-60-2714-01A-01R-0851-07 | 15 | 10 | 31 | Primary solid Tumor |
| TCGA-60-2715-01A-01R-0851-07 | 2 | 4 | 31 | Primary solid Tumor |
| TCGA-60-2716-01A-01R-0851-07 | 1 | 4 | 31 | Primary solid Tumor |
| TCGA-60-2719-01A-01R-0851-07 | 2 | 4 | 31 | Primary solid Tumor |
| TCGA-60-2720-01A-01R-0851-07 | 2 | 4 | 31 | Primary solid Tumor |
| TCGA-60-2721-01A-01R-0851-07 | 2 | 4 | 31 | Primary solid Tumor |
| TCGA-60-2722-01A-01R-0851-07 | 2 | 4 | 31 | Primary solid Tumor |
| TCGA-60-2723-01A-01R-0851-07 | 2 | 4 | 31 | Primary solid Tumor |
| TCGA-60-2724-01A-01R-0851-07 | 2 | 4 | 31 | Primary solid Tumor |
| TCGA-60-2725-01A-01R-1201-07 | 2 | 4 | 60 | Primary solid Tumor |
| TCGA-60-2726-01A-01R-0851-07 | 2 | 4 | 31 | Primary solid Tumor |
| TCGA-61-1721-01A-01R-1569-13 | 14 | 6 | 21 | Primary solid Tumor |
| TCGA-61-1724-01A-01R-1568-13 | 20 | 16 | 21 | Primary solid Tumor |
| TCGA-61-1725-01A-01R-1567-13 | 20 | 16 | 19 | Primary solid Tumor |
| TCGA-61-1728-01A-01R-1568-13 | 20 | 16 | 21 | Primary solid Tumor |
| TCGA-61-1733-01A-01R-1567-13 | 20 | 16 | 19 | Primary solid Tumor |
| TCGA-61-1736-01B-01R-1568-13 | 20 | 16 | 22 | Primary solid Tumor |
| TCGA-61-1737-01A-01R-1567-13 | 20 | NA | 19 | Primary solid Tumor |
| TCGA-61-1738-01A-01R-1567-13 | 20 | 16 | 19 | Primary solid Tumor |
| TCGA-61-1740-01A-01R-1567-13 | 20 | 16 | 19 | Primary solid Tumor |
| TCGA-61-1741-01A-02R-1567-13 | 20 | 16 | 19 | Primary solid Tumor |
| TCGA-61-1743-01A-01R-1568-13 | 20 | 16 | 21 | Primary solid Tumor |
| TCGA-61-1900-01A-01R-1567-13 | 20 | 16 | 19 | Primary solid Tumor |
| TCGA-61-1907-01A-01R-1567-13 | 20 | 16 | 19 | Primary solid Tumor |
| TCGA-61-1910-01A-01R-1567-13 | 20 | 16 | 19 | Primary solid Tumor |
| TCGA-61-1914-01A-01R-1567-13 | 20 | 16 | 19 | Primary solid Tumor |
| TCGA-61-1917-01A-01R-1568-13 | 20 | 16 | 21 | Primary solid Tumor |
| TCGA-61-1918-01A-01R-1568-13 | 20 | 16 | 21 | Primary solid Tumor |
| TCGA-61-1919-01A-01R-1568-13 | 20 | 16 | 21 | Primary solid Tumor |
| TCGA-61-1995-01A-01R-1568-13 | 20 | 16 | 22 | Primary solid Tumor |
| TCGA-61-1998-01A-01R-1568-13 | 20 | 16 | 22 | Primary solid Tumor |
| TCGA-61-2000-01A-01R-1568-13 | 20 | 16 | 22 | Primary solid Tumor |
| TCGA-61-2002-01A-01R-1568-13 | 20 | NA | 22 | Primary solid Tumor |
| TCGA-61-2003-01A-01R-1568-13 | 20 | NA | 22 | Primary solid Tumor |
| TCGA-61-2008-01A-02R-1568-13 | 20 | 16 | 22 | Primary solid Tumor |
| TCGA-61-2009-01A-01R-1568-13 | 20 | 16 | 22 | Primary solid Tumor |
| TCGA-61-2012-01A-01R-1568-13 | 20 | 16 | 22 | Primary solid Tumor |
| TCGA-61-2016-01A-01R-1568-13 | 20 | 16 | 22 | Primary solid Tumor |
| TCGA-61-2088-01A-01R-1568-13 | 20 | 16 | 22 | Primary solid Tumor |
| TCGA-61-2092-01A-01R-1568-13 | 16 | 15 | 22 | Primary solid Tumor |
| TCGA-61-2094-01A-01R-1568-13 | 20 | 16 | 22 | Primary solid Tumor |
| TCGA-61-2095-01A-01R-1568-13 | 20 | NA | 22 | Primary solid Tumor |
| TCGA-61-2097-01A-02R-1568-13 | 20 | 16 | 22 | Primary solid Tumor |
| TCGA-61-2098-01A-01R-1568-13 | 20 | 16 | 22 | Primary solid Tumor |
| TCGA-61-2101-01A-01R-1568-13 | 20 | NA | 22 | Primary solid Tumor |
| TCGA-61-2102-01A-01R-1568-13 | 3 | 16 | 22 | Primary solid Tumor |
| TCGA-61-2104-01A-01R-1568-13 | 20 | 16 | 22 | Primary solid Tumor |
| TCGA-61-2109-01A-01R-1568-13 | 20 | 16 | 22 | Primary solid Tumor |
| TCGA-61-2110-01A-01R-1568-13 | 20 | 16 | 22 | Primary solid Tumor |
| TCGA-61-2111-01A-01R-1568-13 | 16 | 16 | 22 | Primary solid Tumor |
| TCGA-61-2113-01A-01R-1568-13 | 20 | 16 | 22 | Primary solid Tumor |
| TCGA-62-8394-01A-11R-2326-07 | 15 | NA | 232 | Primary solid Tumor |
| TCGA-62-8395-01A-11R-2326-07 | 15 | NA | 232 | Primary solid Tumor |
| TCGA-62-8397-01A-11R-2326-07 | 15 | NA | 232 | Primary solid Tumor |
| TCGA-62-8398-01A-11R-2326-07 | 15 | NA | 232 | Primary solid Tumor |
| TCGA-62-8399-01A-21R-2326-07 | 15 | NA | 232 | Primary solid Tumor |
| TCGA-62-8402-01A-11R-2326-07 | 15 | NA | 232 | Primary solid Tumor |
| TCGA-62-A46O-01A-11R-A24H-07 | 15 | NA | 258 | Primary solid Tumor |
| TCGA-62-A46P-01A-11R-A24H-07 | 15 | NA | 258 | Primary solid Tumor |
| TCGA-62-A46R-01A-11R-A24H-07 | 15 | NA | 258 | Primary solid Tumor |
| TCGA-62-A46S-01A-11R-A24H-07 | 15 | NA | 258 | Primary solid Tumor |
| TCGA-62-A46U-01A-11R-A24H-07 | 15 | NA | 258 | Primary solid Tumor |
| TCGA-62-A46V-01A-11R-A24H-07 | 15 | NA | 258 | Primary solid Tumor |
| TCGA-62-A46Y-01A-11R-A24H-07 | 15 | NA | 258 | Primary solid Tumor |
| TCGA-62-A470-01A-11R-A24H-07 | 15 | NA | 258 | Primary solid Tumor |
| TCGA-62-A471-01A-12R-A24H-07 | 15 | NA | 258 | Primary solid Tumor |
| TCGA-62-A472-01A-11R-A24H-07 | 15 | NA | 258 | Primary solid Tumor |
| TCGA-63-5128-01A-01R-1443-07 | 2 | 4 | 77 | Primary solid Tumor |
| TCGA-63-5131-01A-01R-1443-07 | 2 | 4 | 77 | Primary solid Tumor |
| TCGA-63-6202-01A-11R-1820-07 | 15 | 10 | 140 | Primary solid Tumor |
| TCGA-63-7020-01A-11R-1949-07 | 2 | 4 | 159 | Primary solid Tumor |
| TCGA-63-7021-01A-11R-1949-07 | 2 | 4 | 159 | Primary solid Tumor |
| TCGA-63-7022-01A-11R-1949-07 | 2 | 4 | 159 | Primary solid Tumor |
| TCGA-63-7023-01A-11R-1949-07 | 2 | 4 | 159 | Primary solid Tumor |
| TCGA-63-A5M9-01A-11R-A26W-07 | 2 | NA | 283 | Primary solid Tumor |
| TCGA-63-A5MB-01A-11R-A26W-07 | 2 | NA | 283 | Primary solid Tumor |
| TCGA-63-A5MG-01A-12R-A27Q-07 | 2 | NA | 293 | Primary solid Tumor |
| TCGA-63-A5MH-01A-12R-A27Q-07 | 2 | NA | 293 | Primary solid Tumor |
| TCGA-63-A5MI-01A-12R-A27Q-07 | 2 | NA | 293 | Primary solid Tumor |
| TCGA-63-A5MJ-01A-11R-A27Q-07 | 2 | NA | 293 | Primary solid Tumor |
| TCGA-63-A5ML-01A-31R-A27Q-07 | 2 | NA | 293 | Primary solid Tumor |
| TCGA-63-A5MM-01A-11R-A26W-07 | 2 | NA | 283 | Primary solid Tumor |
| TCGA-63-A5MN-01A-22R-A27Q-07 | 15 | NA | 293 | Primary solid Tumor |
| TCGA-63-A5MP-01A-11R-A26W-07 | 2 | NA | 283 | Primary solid Tumor |
| TCGA-63-A5MR-01A-31R-A27Q-07 | 2 | NA | 293 | Primary solid Tumor |
| TCGA-63-A5MS-01A-11R-A26W-07 | 2 | NA | 283 | Primary solid Tumor |
| TCGA-63-A5MT-01A-21R-A26W-07 | 2 | NA | 283 | Primary solid Tumor |
| TCGA-63-A5MU-01A-11R-A26W-07 | 2 | NA | 283 | Primary solid Tumor |
| TCGA-63-A5MV-01A-21R-A26W-07 | 2 | NA | 283 | Primary solid Tumor |
| TCGA-63-A5MW-01A-11R-A26W-07 | 3 | NA | 283 | Primary solid Tumor |
| TCGA-63-A5MY-01A-11R-A26W-07 | 2 | NA | 283 | Primary solid Tumor |
| TCGA-64-1676-01A-01R-0946-07 | 15 | 10 | 37 | Primary solid Tumor |
| TCGA-64-1677-01A-01R-0946-07 | 15 | 10 | 37 | Primary solid Tumor |
| TCGA-64-1678-01A-01R-0946-07 | 15 | 10 | 37 | Primary solid Tumor |
| TCGA-64-1679-01A-21R-2066-07 | 15 | 10 | 183 | Primary solid Tumor |
| TCGA-64-1680-01A-02R-0946-07 | 15 | 10 | 37 | Primary solid Tumor |
| TCGA-64-1681-01A-11R-2066-07 | 15 | 10 | 183 | Primary solid Tumor |
| TCGA-64-5774-01A-01R-1628-07 | 15 | 10 | 84 | Primary solid Tumor |
| TCGA-64-5775-01A-01R-1628-07 | 3 | 2 | 84 | Primary solid Tumor |
| TCGA-64-5778-01A-01R-1628-07 | 15 | 10 | 84 | Primary solid Tumor |
| TCGA-64-5779-01A-01R-1628-07 | 15 | 10 | 84 | Primary solid Tumor |
| TCGA-64-5781-01A-01R-1628-07 | 15 | 10 | 84 | Primary solid Tumor |
| TCGA-64-5815-01A-01R-1628-07 | 15 | 10 | 84 | Primary solid Tumor |
| TCGA-66-2727-01A-01R-0980-07 | 2 | 4 | 39 | Primary solid Tumor |
| TCGA-66-2734-01A-01R-0980-07 | 2 | 4 | 39 | Primary solid Tumor |
| TCGA-66-2737-01A-01R-0980-07 | 2 | 4 | 39 | Primary solid Tumor |
| TCGA-66-2742-01A-01R-0980-07 | 2 | 4 | 39 | Primary solid Tumor |
| TCGA-66-2744-01A-01R-0980-07 | 2 | 4 | 39 | Primary solid Tumor |
| TCGA-66-2753-01A-01R-0980-07 | 2 | 4 | 39 | Primary solid Tumor |
| TCGA-66-2754-01A-01R-0980-07 | 15 | 10 | 39 | Primary solid Tumor |
| TCGA-66-2755-01A-01R-0851-07 | 2 | 4 | 31 | Primary solid Tumor |
| TCGA-66-2756-01A-01R-0851-07 | 15 | 10 | 31 | Primary solid Tumor |
| TCGA-66-2757-01A-01R-0851-07 | 8 | 12 | 31 | Primary solid Tumor |
| TCGA-66-2758-01A-02R-0851-07 | 2 | 4 | 31 | Primary solid Tumor |
| TCGA-66-2759-01A-01R-0851-07 | 2 | 4 | 31 | Primary solid Tumor |
| TCGA-66-2763-01A-01R-0851-07 | 2 | 4 | 31 | Primary solid Tumor |
| TCGA-66-2765-01A-01R-0851-07 | 2 | 4 | 31 | Primary solid Tumor |
| TCGA-66-2766-01A-01R-0851-07 | 2 | 4 | 31 | Primary solid Tumor |
| TCGA-66-2767-01A-01R-0851-07 | 2 | 4 | 31 | Primary solid Tumor |
| TCGA-66-2768-01A-01R-0851-07 | 2 | 4 | 31 | Primary solid Tumor |
| TCGA-66-2769-01A-02R-0851-07 | 2 | 4 | 31 | Primary solid Tumor |
| TCGA-66-2770-01A-01R-0851-07 | 2 | 4 | 31 | Primary solid Tumor |
| TCGA-66-2771-01A-01R-0980-07 | 2 | 4 | 39 | Primary solid Tumor |
| TCGA-66-2773-01A-01R-1201-07 | 2 | 4 | 60 | Primary solid Tumor |
| TCGA-66-2777-01A-01R-1201-07 | 2 | 4 | 60 | Primary solid Tumor |
| TCGA-66-2778-01A-02R-0851-07 | 2 | 4 | 31 | Primary solid Tumor |
| TCGA-66-2780-01A-01R-0851-07 | 2 | 4 | 31 | Primary solid Tumor |
| TCGA-66-2781-01A-01R-0851-07 | 2 | 4 | 31 | Primary solid Tumor |
| TCGA-66-2782-01A-01R-0851-07 | 2 | 4 | 31 | Primary solid Tumor |
| TCGA-66-2783-01A-01R-1201-07 | 2 | 4 | 60 | Primary solid Tumor |
| TCGA-66-2785-01A-01R-0851-07 | 2 | 8 | 31 | Primary solid Tumor |
| TCGA-66-2786-01A-01R-0851-07 | 2 | 4 | 31 | Primary solid Tumor |
| TCGA-66-2787-01A-01R-0980-07 | 2 | 4 | 39 | Primary solid Tumor |
| TCGA-66-2788-01A-01R-0980-07 | 2 | 4 | 39 | Primary solid Tumor |
| TCGA-66-2789-01A-01R-0980-07 | 2 | 4 | 39 | Primary solid Tumor |
| TCGA-66-2790-01A-01R-0980-07 | 2 | 4 | 39 | Primary solid Tumor |
| TCGA-66-2791-01A-01R-0980-07 | 2 | 4 | 39 | Primary solid Tumor |
| TCGA-66-2792-01A-01R-0980-07 | 2 | 4 | 39 | Primary solid Tumor |
| TCGA-66-2793-01A-01R-1201-07 | 2 | 4 | 60 | Primary solid Tumor |
| TCGA-66-2794-01A-01R-1201-07 | 2 | 4 | 60 | Primary solid Tumor |
| TCGA-66-2795-01A-02R-0980-07 | 2 | 4 | 39 | Primary solid Tumor |
| TCGA-66-2800-01A-01R-1201-07 | 2 | 4 | 60 | Primary solid Tumor |
| TCGA-67-3770-01A-01R-0946-07 | 15 | 10 | 37 | Primary solid Tumor |
| TCGA-67-3771-01A-01R-0946-07 | 15 | 10 | 37 | Primary solid Tumor |
| TCGA-67-3772-01A-01R-0946-07 | 15 | 10 | 37 | Primary solid Tumor |
| TCGA-67-3773-01A-01R-0946-07 | 15 | 10 | 37 | Primary solid Tumor |
| TCGA-67-3774-01A-01R-0946-07 | 15 | 10 | 37 | Primary solid Tumor |
| TCGA-67-4679-01B-01R-1755-07 | 15 | 10 | 119 | Primary solid Tumor |
| TCGA-67-6215-01A-11R-1755-07 | 15 | 10 | 119 | Primary solid Tumor |
| TCGA-67-6216-01A-11R-1755-07 | 15 | 10 | 119 | Primary solid Tumor |
| TCGA-67-6217-01A-11R-1755-07 | 15 | 10 | 119 | Primary solid Tumor |
| TCGA-68-7755-01A-11R-2125-07 | 2 | NA | 193 | Primary solid Tumor |
| TCGA-68-7756-01A-11R-2125-07 | 2 | NA | 193 | Primary solid Tumor |
| TCGA-68-7757-01B-11R-2296-07 | 2 | NA | 225 | Primary solid Tumor |
| TCGA-68-8250-01A-11R-2296-07 | 2 | NA | 225 | Primary solid Tumor |
| TCGA-68-8251-01A-11R-2296-07 | 2 | NA | 225 | Primary solid Tumor |
| TCGA-68-A59I-01A-11R-A262-07 | 2 | NA | 276 | Primary solid Tumor |
| TCGA-68-A59J-01A-21R-A26W-07 | 2 | NA | 283 | Primary solid Tumor |
| TCGA-69-7760-01A-11R-2170-07 | 15 | 10 | 196 | Primary solid Tumor |
| TCGA-69-7761-01A-11R-2170-07 | 15 | 10 | 196 | Primary solid Tumor |
| TCGA-69-7763-01A-11R-2170-07 | 15 | 10 | 196 | Primary solid Tumor |
| TCGA-69-7764-01A-11R-2170-07 | 15 | 10 | 196 | Primary solid Tumor |
| TCGA-69-7765-01A-11R-2170-07 | 15 | 10 | 196 | Primary solid Tumor |
| TCGA-69-7973-01A-11R-2187-07 | 15 | 10 | 204 | Primary solid Tumor |
| TCGA-69-7974-01A-11R-2187-07 | 15 | 10 | 204 | Primary solid Tumor |
| TCGA-69-7978-01A-11R-2187-07 | 15 | 10 | 204 | Primary solid Tumor |
| TCGA-69-7979-01A-11R-2187-07 | 15 | 10 | 204 | Primary solid Tumor |
| TCGA-69-7980-01A-11R-2187-07 | 15 | 10 | 204 | Primary solid Tumor |
| TCGA-69-8253-01A-11R-2287-07 | 15 | 10 | 222 | Primary solid Tumor |
| TCGA-69-8254-01A-11R-2287-07 | 15 | 10 | 222 | Primary solid Tumor |
| TCGA-69-8255-01A-11R-2287-07 | 6 | 13 | 222 | Primary solid Tumor |
| TCGA-69-8453-01A-12R-2326-07 | 15 | NA | 232 | Primary solid Tumor |
| TCGA-69-A59K-01A-11R-A262-07 | 15 | NA | 278 | Primary solid Tumor |
| TCGA-6A-AB49-01A-12R-A405-07 | 2 | NA | 415 | Primary solid Tumor |
| TCGA-6D-AA2E-01A-11R-A37O-07 | 14 | NA | 387 | Primary solid Tumor |
| TCGA-70-6722-01A-11R-1820-07 | 2 | 4 | 140 | Primary solid Tumor |
| TCGA-70-6723-01A-11R-1820-07 | 2 | 4 | 140 | Primary solid Tumor |
| TCGA-71-6725-01A-11R-1858-07 | 15 | 10 | 144 | Primary solid Tumor |
| TCGA-71-8520-01A-11R-2403-07 | 15 | NA | 238 | Primary solid Tumor |
| TCGA-73-4658-01A-01R-1755-07 | 15 | 10 | 119 | Primary solid Tumor |
| TCGA-73-4659-01A-01R-1206-07 | 15 | 10 | 58 | Primary solid Tumor |
| TCGA-73-4662-01A-01R-1206-07 | 15 | 10 | 58 | Primary solid Tumor |
| TCGA-73-4666-01A-01R-1206-07 | 15 | 10 | 58 | Primary solid Tumor |
| TCGA-73-4668-01A-01R-1206-07 | 15 | 10 | 58 | Primary solid Tumor |
| TCGA-73-4670-01A-01R-1206-07 | 15 | 10 | 58 | Primary solid Tumor |
| TCGA-73-4675-01A-01R-1206-07 | 15 | 10 | 58 | Primary solid Tumor |
| TCGA-73-4676-01A-01R-1755-07 | 15 | 10 | 119 | Primary solid Tumor |
| TCGA-73-4677-01A-01R-1206-07 | 15 | 10 | 58 | Primary solid Tumor |
| TCGA-73-7498-01A-12R-2187-07 | 15 | 10 | 204 | Primary solid Tumor |
| TCGA-73-7499-01A-11R-2187-07 | 15 | 10 | 204 | Primary solid Tumor |
| TCGA-73-A9RS-01A-11R-A41B-07 | 15 | NA | 423 | Primary solid Tumor |
| TCGA-75-5122-01A-01R-1755-07 | 15 | 10 | 119 | Primary solid Tumor |
| TCGA-75-5125-01A-01R-1755-07 | 15 | 10 | 119 | Primary solid Tumor |
| TCGA-75-5126-01A-01R-1755-07 | 15 | 10 | 119 | Primary solid Tumor |
| TCGA-75-5146-01A-01R-1628-07 | 15 | 10 | 84 | Primary solid Tumor |
| TCGA-75-5147-01A-01R-1628-07 | 15 | 10 | 84 | Primary solid Tumor |
| TCGA-75-6203-01A-11R-1755-07 | 15 | 10 | 119 | Primary solid Tumor |
| TCGA-75-6205-01A-11R-1755-07 | 15 | 10 | 119 | Primary solid Tumor |
| TCGA-75-6206-01A-11R-1755-07 | 15 | 10 | 119 | Primary solid Tumor |
| TCGA-75-6207-01A-11R-1755-07 | 15 | 10 | 119 | Primary solid Tumor |
| TCGA-75-6211-01A-11R-1755-07 | 15 | 10 | 119 | Primary solid Tumor |
| TCGA-75-6212-01A-11R-1755-07 | 15 | 10 | 119 | Primary solid Tumor |
| TCGA-75-6214-01A-41R-1949-07 | 2 | 4 | 160 | Primary solid Tumor |
| TCGA-75-7025-01A-12R-1949-07 | 15 | 10 | 160 | Primary solid Tumor |
| TCGA-75-7027-01A-11R-1949-07 | 15 | 10 | 160 | Primary solid Tumor |
| TCGA-75-7030-01A-11R-1949-07 | 15 | 10 | 160 | Primary solid Tumor |
| TCGA-75-7031-01A-11R-1949-07 | 15 | 10 | 160 | Primary solid Tumor |
| TCGA-76-4925-01A-01R-1850-01 | 3 | 6 | 79 | Primary solid Tumor |
| TCGA-76-4926-01B-01R-1850-01 | 3 | 6 | 79 | Primary solid Tumor |
| TCGA-76-4927-01A-01R-1850-01 | 3 | 6 | 79 | Primary solid Tumor |
| TCGA-76-4928-01B-01R-1850-01 | 3 | 6 | 79 | Primary solid Tumor |
| TCGA-76-4929-01A-01R-1850-01 | 3 | 6 | 79 | Primary solid Tumor |
| TCGA-76-4931-01A-01R-1850-01 | 3 | 6 | 79 | Primary solid Tumor |
| TCGA-76-4932-01A-01R-1850-01 | 3 | 6 | 79 | Primary solid Tumor |
| TCGA-77-6842-01A-11R-1949-07 | 2 | 4 | 159 | Primary solid Tumor |
| TCGA-77-6843-01A-11R-1949-07 | 2 | 4 | 159 | Primary solid Tumor |
| TCGA-77-6844-01A-11R-1949-07 | 2 | 4 | 159 | Primary solid Tumor |
| TCGA-77-6845-01A-11R-1949-07 | 2 | 4 | 159 | Primary solid Tumor |
| TCGA-77-7138-01A-41R-2045-07 | 2 | 4 | 181 | Primary solid Tumor |
| TCGA-77-7139-01A-11R-2045-07 | 2 | 4 | 181 | Primary solid Tumor |
| TCGA-77-7140-01A-41R-2045-07 | 2 | 4 | 181 | Primary solid Tumor |
| TCGA-77-7141-01A-11R-2045-07 | 2 | 4 | 181 | Primary solid Tumor |
| TCGA-77-7142-01A-11R-2045-07 | 2 | 4 | 181 | Primary solid Tumor |
| TCGA-77-7335-01A-11R-2045-07 | 2 | 4 | 181 | Primary solid Tumor |
| TCGA-77-7337-01A-21R-2045-07 | 2 | 4 | 181 | Primary solid Tumor |
| TCGA-77-7338-01A-11R-2045-07 | 2 | 4 | 181 | Primary solid Tumor |
| TCGA-77-7463-01A-11R-2045-07 | 2 | 4 | 181 | Primary solid Tumor |
| TCGA-77-7465-01A-11R-2045-07 | 2 | 4 | 181 | Primary solid Tumor |
| TCGA-77-8007-01A-11R-2187-07 | 15 | 10 | 208 | Primary solid Tumor |
| TCGA-77-8008-01A-21R-2187-07 | 2 | 4 | 208 | Primary solid Tumor |
| TCGA-77-8009-01A-11R-2187-07 | 2 | 4 | 208 | Primary solid Tumor |
| TCGA-77-8128-01A-11R-2247-07 | 2 | NA | 214 | Primary solid Tumor |
| TCGA-77-8130-01A-11R-2247-07 | 2 | NA | 214 | Primary solid Tumor |
| TCGA-77-8131-01A-11R-2247-07 | 2 | NA | 214 | Primary solid Tumor |
| TCGA-77-8133-01A-12R-2247-07 | 2 | NA | 214 | Primary solid Tumor |
| TCGA-77-8136-01A-11R-2247-07 | 2 | NA | 214 | Primary solid Tumor |
| TCGA-77-8138-01A-11R-2247-07 | 2 | NA | 214 | Primary solid Tumor |
| TCGA-77-8139-01A-11R-2247-07 | 2 | NA | 214 | Primary solid Tumor |
| TCGA-77-8140-01A-11R-2247-07 | 2 | NA | 214 | Primary solid Tumor |
| TCGA-77-8143-01A-11R-2247-07 | 2 | NA | 214 | Primary solid Tumor |
| TCGA-77-8144-01A-11R-2247-07 | 2 | NA | 214 | Primary solid Tumor |
| TCGA-77-8145-01A-11R-2247-07 | 2 | NA | 214 | Primary solid Tumor |
| TCGA-77-8146-01A-11R-2247-07 | 2 | NA | 214 | Primary solid Tumor |
| TCGA-77-8148-01A-11R-2247-07 | 2 | NA | 214 | Primary solid Tumor |
| TCGA-77-8150-01A-11R-2247-07 | 2 | NA | 214 | Primary solid Tumor |
| TCGA-77-8153-01A-11R-2403-07 | 2 | NA | 243 | Primary solid Tumor |
| TCGA-77-8154-01A-11R-2247-07 | 2 | NA | 214 | Primary solid Tumor |
| TCGA-77-8156-01A-11R-2247-07 | 2 | NA | 214 | Primary solid Tumor |
| TCGA-77-A5FZ-01A-31R-A27Q-07 | 15 | NA | 293 | Primary solid Tumor |
| TCGA-77-A5G1-01A-11R-A27Q-07 | 2 | NA | 293 | Primary solid Tumor |
| TCGA-77-A5G3-01A-31R-A27Q-07 | 2 | NA | 293 | Primary solid Tumor |
| TCGA-77-A5G6-01A-11R-A27Q-07 | 2 | NA | 293 | Primary solid Tumor |
| TCGA-77-A5G7-01B-11R-A27Q-07 | 2 | NA | 293 | Primary solid Tumor |
| TCGA-77-A5G8-01B-11R-A27Q-07 | 2 | NA | 293 | Primary solid Tumor |
| TCGA-77-A5GA-01A-11R-A27Q-07 | 2 | NA | 293 | Primary solid Tumor |
| TCGA-77-A5GB-01B-11R-A27Q-07 | 2 | NA | 293 | Primary solid Tumor |
| TCGA-77-A5GF-01A-21R-A27Q-07 | 2 | NA | 293 | Primary solid Tumor |
| TCGA-77-A5GH-01A-11R-A27Q-07 | 2 | NA | 293 | Primary solid Tumor |
| TCGA-78-7143-01A-11R-2039-07 | 15 | 10 | 166 | Primary solid Tumor |
| TCGA-78-7145-01A-11R-2039-07 | 15 | 10 | 166 | Primary solid Tumor |
| TCGA-78-7146-01A-11R-2039-07 | 15 | 10 | 166 | Primary solid Tumor |
| TCGA-78-7147-01A-11R-2039-07 | 15 | 10 | 166 | Primary solid Tumor |
| TCGA-78-7148-01A-11R-2039-07 | 15 | 10 | 166 | Primary solid Tumor |
| TCGA-78-7149-01A-11R-2039-07 | 15 | 10 | 166 | Primary solid Tumor |
| TCGA-78-7150-01A-21R-2039-07 | 15 | 10 | 166 | Primary solid Tumor |
| TCGA-78-7152-01A-11R-2039-07 | 15 | 10 | 166 | Primary solid Tumor |
| TCGA-78-7153-01A-11R-2039-07 | 15 | 10 | 166 | Primary solid Tumor |
| TCGA-78-7154-01A-11R-2039-07 | 15 | 10 | 166 | Primary solid Tumor |
| TCGA-78-7155-01A-11R-2039-07 | 18 | 14 | 166 | Primary solid Tumor |
| TCGA-78-7156-01A-11R-2039-07 | 15 | 10 | 166 | Primary solid Tumor |
| TCGA-78-7158-01A-11R-2039-07 | 15 | 10 | 166 | Primary solid Tumor |
| TCGA-78-7159-01A-11R-2039-07 | 15 | 10 | 166 | Primary solid Tumor |
| TCGA-78-7160-01A-11R-2039-07 | 15 | 10 | 166 | Primary solid Tumor |
| TCGA-78-7161-01A-11R-2039-07 | 15 | 10 | 166 | Primary solid Tumor |
| TCGA-78-7162-01A-21R-2066-07 | 15 | 10 | 183 | Primary solid Tumor |
| TCGA-78-7163-01A-12R-2066-07 | 15 | 10 | 183 | Primary solid Tumor |
| TCGA-78-7166-01A-12R-2066-07 | 15 | 10 | 183 | Primary solid Tumor |
| TCGA-78-7167-01A-11R-2066-07 | 15 | 10 | 183 | Primary solid Tumor |
| TCGA-78-7220-01A-11R-2039-07 | 15 | 10 | 166 | Primary solid Tumor |
| TCGA-78-7535-01A-11R-2066-07 | 15 | 10 | 183 | Primary solid Tumor |
| TCGA-78-7536-01A-11R-2066-07 | 15 | 10 | 183 | Primary solid Tumor |
| TCGA-78-7537-01A-11R-2066-07 | 15 | 10 | 183 | Primary solid Tumor |
| TCGA-78-7539-01A-11R-2066-07 | 15 | 10 | 183 | Primary solid Tumor |
| TCGA-78-7540-01A-11R-2066-07 | 15 | 10 | 183 | Primary solid Tumor |
| TCGA-78-7542-01A-21R-2066-07 | 2 | 4 | 183 | Primary solid Tumor |
| TCGA-78-7633-01A-11R-2066-07 | 15 | 10 | 183 | Primary solid Tumor |
| TCGA-78-8640-01A-11R-2403-07 | 15 | NA | 238 | Primary solid Tumor |
| TCGA-78-8648-01A-11R-2403-07 | 15 | NA | 238 | Primary solid Tumor |
| TCGA-78-8655-01A-11R-2403-07 | 15 | NA | 238 | Primary solid Tumor |
| TCGA-78-8660-01A-11R-2403-07 | 15 | NA | 238 | Primary solid Tumor |
| TCGA-78-8662-01A-11R-2403-07 | 15 | NA | 238 | Primary solid Tumor |
| TCGA-79-5596-01A-31R-1949-07 | 2 | 4 | 159 | Primary solid Tumor |
| TCGA-80-5607-01A-31R-1949-07 | 15 | 10 | 160 | Primary solid Tumor |
| TCGA-80-5608-01A-31R-1949-07 | 15 | 10 | 160 | Primary solid Tumor |
| TCGA-80-5611-01A-01R-1628-07 | 15 | 10 | 84 | Primary solid Tumor |
| TCGA-83-5908-01A-21R-2287-07 | 15 | 10 | 84 | Primary solid Tumor |
| TCGA-85-6175-01A-11R-1820-07 | 2 | 4 | 140 | Primary solid Tumor |
| TCGA-85-6560-01A-11R-1820-07 | 3 | 2 | 140 | Primary solid Tumor |
| TCGA-85-6561-01A-11R-1820-07 | 2 | 4 | 140 | Primary solid Tumor |
| TCGA-85-6798-01A-11R-1949-07 | 2 | 4 | 159 | Primary solid Tumor |
| TCGA-85-7696-01A-11R-2125-07 | 2 | NA | 193 | Primary solid Tumor |
| TCGA-85-7697-01A-11R-2125-07 | 2 | NA | 193 | Primary solid Tumor |
| TCGA-85-7698-01A-11R-2125-07 | 2 | NA | 193 | Primary solid Tumor |
| TCGA-85-7699-01A-11R-2125-07 | 2 | NA | 193 | Primary solid Tumor |
| TCGA-85-7710-01A-11R-2125-07 | 2 | 4 | 193 | Primary solid Tumor |
| TCGA-85-7843-01A-11R-2125-07 | 2 | NA | 193 | Primary solid Tumor |
| TCGA-85-7844-01A-11R-2125-07 | 2 | 4 | 193 | Primary solid Tumor |
| TCGA-85-7950-01A-11R-2187-07 | 2 | 4 | 208 | Primary solid Tumor |
| TCGA-85-8048-01A-11R-2247-07 | 2 | NA | 214 | Primary solid Tumor |
| TCGA-85-8049-01A-11R-2247-07 | 2 | NA | 214 | Primary solid Tumor |
| TCGA-85-8052-01A-11R-2247-07 | 2 | NA | 214 | Primary solid Tumor |
| TCGA-85-8070-01A-11R-2247-07 | 2 | NA | 214 | Primary solid Tumor |
| TCGA-85-8071-01A-11R-2247-07 | 2 | NA | 214 | Primary solid Tumor |
| TCGA-85-8072-01A-31R-2247-07 | 2 | NA | 214 | Primary solid Tumor |
| TCGA-85-8276-01A-11R-2296-07 | 2 | NA | 225 | Primary solid Tumor |
| TCGA-85-8277-01A-11R-2296-07 | 2 | NA | 225 | Primary solid Tumor |
| TCGA-85-8287-01A-11R-2296-07 | 2 | NA | 225 | Primary solid Tumor |
| TCGA-85-8288-01A-11R-2296-07 | 2 | NA | 225 | Primary solid Tumor |
| TCGA-85-8350-01A-11R-2296-07 | 2 | NA | 225 | Primary solid Tumor |
| TCGA-85-8351-01A-11R-2296-07 | 2 | NA | 225 | Primary solid Tumor |
| TCGA-85-8352-01A-31R-2326-07 | 3 | NA | 233 | Primary solid Tumor |
| TCGA-85-8353-01A-21R-2296-07 | 2 | NA | 225 | Primary solid Tumor |
| TCGA-85-8354-01A-31R-2326-07 | 2 | NA | 233 | Primary solid Tumor |
| TCGA-85-8355-01A-11R-2296-07 | 2 | NA | 225 | Primary solid Tumor |
| TCGA-85-8479-01A-11R-2326-07 | 2 | NA | 233 | Primary solid Tumor |
| TCGA-85-8481-01A-11R-2326-07 | 2 | NA | 233 | Primary solid Tumor |
| TCGA-85-8580-01A-31R-2403-07 | 2 | NA | 243 | Primary solid Tumor |
| TCGA-85-8582-01A-21R-2403-07 | 2 | NA | 243 | Primary solid Tumor |
| TCGA-85-8584-01A-11R-2403-07 | 15 | NA | 243 | Primary solid Tumor |
| TCGA-85-8664-01A-11R-2403-07 | 2 | NA | 243 | Primary solid Tumor |
| TCGA-85-8666-01A-11R-2403-07 | 2 | NA | 243 | Primary solid Tumor |
| TCGA-85-A4CL-01A-41R-A26W-07 | 2 | NA | 283 | Primary solid Tumor |
| TCGA-85-A4CN-01A-11R-A24H-07 | 2 | NA | 259 | Primary solid Tumor |
| TCGA-85-A4JB-01A-51R-A262-07 | 2 | NA | 276 | Primary solid Tumor |
| TCGA-85-A4JC-01A-11R-A24Z-07 | 2 | NA | 267 | Primary solid Tumor |
| TCGA-85-A4PA-01A-11R-A24Z-07 | 6 | NA | 267 | Primary solid Tumor |
| TCGA-85-A4QQ-01A-41R-A262-07 | 2 | NA | 276 | Primary solid Tumor |
| TCGA-85-A4QR-01A-11R-A24Z-07 | 2 | NA | 267 | Primary solid Tumor |
| TCGA-85-A50M-01A-21R-A262-07 | 2 | NA | 276 | Primary solid Tumor |
| TCGA-85-A50Z-01A-21R-A262-07 | 2 | NA | 276 | Primary solid Tumor |
| TCGA-85-A510-01A-11R-A26W-07 | 15 | NA | 283 | Primary solid Tumor |
| TCGA-85-A511-01A-21R-A262-07 | 2 | NA | 276 | Primary solid Tumor |
| TCGA-85-A512-01A-11R-A26W-07 | 2 | NA | 283 | Primary solid Tumor |
| TCGA-85-A513-01A-12R-A26W-07 | 15 | NA | 283 | Primary solid Tumor |
| TCGA-85-A53L-01A-21R-A26W-07 | 2 | NA | 283 | Primary solid Tumor |
| TCGA-85-A5B5-01A-21R-A26W-07 | 2 | NA | 283 | Primary solid Tumor |
| TCGA-86-6562-01A-11R-1755-07 | 15 | 10 | 119 | Primary solid Tumor |
| TCGA-86-6851-01A-11R-1949-07 | 15 | 10 | 160 | Primary solid Tumor |
| TCGA-86-7701-01A-11R-2170-07 | 15 | 10 | 196 | Primary solid Tumor |
| TCGA-86-7711-01A-11R-2066-07 | 15 | 10 | 183 | Primary solid Tumor |
| TCGA-86-7713-01A-11R-2066-07 | 15 | 10 | 183 | Primary solid Tumor |
| TCGA-86-7714-01A-12R-2170-07 | 15 | 10 | 196 | Primary solid Tumor |
| TCGA-86-7953-01A-11R-2187-07 | 15 | 10 | 204 | Primary solid Tumor |
| TCGA-86-7954-01A-11R-2187-07 | 15 | 10 | 204 | Primary solid Tumor |
| TCGA-86-7955-01A-11R-2187-07 | 15 | 10 | 204 | Primary solid Tumor |
| TCGA-86-8054-01A-11R-2241-07 | 15 | 10 | 213 | Primary solid Tumor |
| TCGA-86-8055-01A-11R-2241-07 | 15 | 10 | 213 | Primary solid Tumor |
| TCGA-86-8056-01A-11R-2241-07 | 15 | 10 | 213 | Primary solid Tumor |
| TCGA-86-8073-01A-11R-2241-07 | 15 | 10 | 213 | Primary solid Tumor |
| TCGA-86-8074-01A-11R-2241-07 | 15 | 10 | 213 | Primary solid Tumor |
| TCGA-86-8075-01A-11R-2241-07 | 15 | 10 | 213 | Primary solid Tumor |
| TCGA-86-8076-01A-31R-2241-07 | 15 | 10 | 213 | Primary solid Tumor |
| TCGA-86-8278-01A-11R-2287-07 | 15 | NA | 222 | Primary solid Tumor |
| TCGA-86-8279-01A-11R-2287-07 | 15 | 10 | 222 | Primary solid Tumor |
| TCGA-86-8280-01A-11R-2287-07 | 15 | 10 | 222 | Primary solid Tumor |
| TCGA-86-8281-01A-11R-2287-07 | 15 | 10 | 222 | Primary solid Tumor |
| TCGA-86-8358-01A-11R-2326-07 | 15 | NA | 232 | Primary solid Tumor |
| TCGA-86-8359-01A-11R-2326-07 | 15 | NA | 232 | Primary solid Tumor |
| TCGA-86-8585-01A-11R-2403-07 | 15 | NA | 238 | Primary solid Tumor |
| TCGA-86-8668-01A-11R-2403-07 | 15 | NA | 238 | Primary solid Tumor |
| TCGA-86-8669-01A-11R-2403-07 | 15 | NA | 238 | Primary solid Tumor |
| TCGA-86-8671-01A-11R-2403-07 | 15 | NA | 238 | Primary solid Tumor |
| TCGA-86-8672-01A-21R-2403-07 | 15 | NA | 238 | Primary solid Tumor |
| TCGA-86-8673-01A-11R-2403-07 | 15 | NA | 238 | Primary solid Tumor |
| TCGA-86-8674-01A-21R-2403-07 | 15 | NA | 238 | Primary solid Tumor |
| TCGA-86-A456-01A-11R-A24H-07 | 15 | NA | 258 | Primary solid Tumor |
| TCGA-86-A4D0-01A-11R-A24H-07 | 15 | NA | 258 | Primary solid Tumor |
| TCGA-86-A4JF-01A-11R-A24X-07 | 15 | NA | 264 | Primary solid Tumor |
| TCGA-86-A4P7-01A-11R-A24X-07 | 15 | NA | 264 | Primary solid Tumor |
| TCGA-86-A4P8-01A-11R-A24X-07 | 15 | NA | 264 | Primary solid Tumor |
| TCGA-90-6837-01A-11R-1949-07 | 2 | 4 | 159 | Primary solid Tumor |
| TCGA-90-7766-01A-21R-2125-07 | 2 | NA | 193 | Primary solid Tumor |
| TCGA-90-7767-01A-11R-2125-07 | 2 | NA | 193 | Primary solid Tumor |
| TCGA-90-7769-01A-11R-2125-07 | 2 | NA | 193 | Primary solid Tumor |
| TCGA-90-7964-01A-21R-2187-07 | 2 | 4 | 208 | Primary solid Tumor |
| TCGA-90-A4ED-01A-31R-A24Z-07 | 2 | NA | 267 | Primary solid Tumor |
| TCGA-90-A4EE-01A-11R-A24Z-07 | 2 | NA | 267 | Primary solid Tumor |
| TCGA-90-A59Q-01A-11R-A26W-07 | 15 | NA | 283 | Primary solid Tumor |
| TCGA-91-6828-01A-11R-1858-07 | 15 | 10 | 144 | Primary solid Tumor |
| TCGA-91-6829-01A-21R-1858-07 | 15 | 10 | 144 | Primary solid Tumor |
| TCGA-91-6830-01A-11R-1949-07 | 15 | 10 | 160 | Primary solid Tumor |
| TCGA-91-6831-01A-11R-1858-07 | 15 | 10 | 144 | Primary solid Tumor |
| TCGA-91-6835-01A-11R-1858-07 | 15 | 10 | 144 | Primary solid Tumor |
| TCGA-91-6836-01A-21R-1858-07 | 15 | 10 | 144 | Primary solid Tumor |
| TCGA-91-6840-01A-11R-1949-07 | 18 | 10 | 160 | Primary solid Tumor |
| TCGA-91-6847-01A-11R-1949-07 | 19 | 14 | 160 | Primary solid Tumor |
| TCGA-91-6848-01A-11R-1949-07 | 3 | 2 | 160 | Primary solid Tumor |
| TCGA-91-6849-01A-11R-1949-07 | 15 | 10 | 160 | Primary solid Tumor |
| TCGA-91-7771-01A-11R-2170-07 | 15 | 10 | 196 | Primary solid Tumor |
| TCGA-91-8496-01A-11R-2403-07 | 15 | NA | 238 | Primary solid Tumor |
| TCGA-91-8497-01A-11R-2403-07 | 15 | NA | 238 | Primary solid Tumor |
| TCGA-91-8499-01A-11R-2403-07 | 8 | NA | 238 | Primary solid Tumor |
| TCGA-91-A4BC-01A-11R-A24H-07 | 15 | NA | 258 | Primary solid Tumor |
| TCGA-91-A4BD-01A-11R-A24H-07 | 15 | NA | 258 | Primary solid Tumor |
| TCGA-92-7340-01A-21R-2045-07 | 2 | 4 | 181 | Primary solid Tumor |
| TCGA-92-7341-01A-31R-2045-07 | 2 | 4 | 181 | Primary solid Tumor |
| TCGA-92-8063-01A-11R-2247-07 | 2 | NA | 214 | Primary solid Tumor |
| TCGA-92-8064-01A-11R-2247-07 | 2 | NA | 214 | Primary solid Tumor |
| TCGA-92-8065-01A-11R-2247-07 | 2 | NA | 214 | Primary solid Tumor |
| TCGA-93-7347-01A-11R-2187-07 | 15 | 10 | 204 | Primary solid Tumor |
| TCGA-93-7348-01A-21R-2039-07 | 15 | 10 | 166 | Primary solid Tumor |
| TCGA-93-8067-01A-11R-2287-07 | 15 | 10 | 222 | Primary solid Tumor |
| TCGA-93-A4JN-01A-11R-A24X-07 | 15 | NA | 264 | Primary solid Tumor |
| TCGA-93-A4JO-01A-21R-A24X-07 | 15 | NA | 264 | Primary solid Tumor |
| TCGA-93-A4JP-01A-11R-A24X-07 | 15 | NA | 264 | Primary solid Tumor |
| TCGA-93-A4JQ-01A-11R-A24X-07 | 15 | NA | 264 | Primary solid Tumor |
| TCGA-94-7033-01A-11R-1949-07 | 2 | 4 | 159 | Primary solid Tumor |
| TCGA-94-7557-01A-11R-2125-07 | 8 | 4 | 193 | Primary solid Tumor |
| TCGA-94-7943-01A-11R-2187-07 | 8 | 4 | 208 | Primary solid Tumor |
| TCGA-94-8035-01A-11R-2247-07 | 2 | NA | 214 | Primary solid Tumor |
| TCGA-94-8490-01A-11R-2326-07 | 2 | NA | 233 | Primary solid Tumor |
| TCGA-94-8491-01A-11R-2326-07 | 2 | NA | 233 | Primary solid Tumor |
| TCGA-94-A4VJ-01A-11R-A24Z-07 | 2 | NA | 267 | Primary solid Tumor |
| TCGA-94-A5I4-01A-11R-A26W-07 | 2 | NA | 283 | Primary solid Tumor |
| TCGA-94-A5I6-01A-21R-A27Q-07 | 2 | NA | 293 | Primary solid Tumor |
| TCGA-95-7039-01A-11R-1949-07 | 15 | 10 | 160 | Primary solid Tumor |
| TCGA-95-7043-01A-11R-1949-07 | 15 | 10 | 160 | Primary solid Tumor |
| TCGA-95-7562-01A-11R-2241-07 | 15 | 10 | 213 | Primary solid Tumor |
| TCGA-95-7567-01A-11R-2066-07 | 15 | 10 | 183 | Primary solid Tumor |
| TCGA-95-7944-01A-11R-2187-07 | 15 | 10 | 204 | Primary solid Tumor |
| TCGA-95-7947-01A-11R-2187-07 | 15 | 10 | 204 | Primary solid Tumor |
| TCGA-95-7948-01A-11R-2187-07 | 15 | 10 | 204 | Primary solid Tumor |
| TCGA-95-8039-01A-11R-2241-07 | 15 | 10 | 213 | Primary solid Tumor |
| TCGA-95-8494-01A-11R-2326-07 | 15 | NA | 232 | Primary solid Tumor |
| TCGA-95-A4VK-01A-11R-A262-07 | 15 | NA | 278 | Primary solid Tumor |
| TCGA-95-A4VN-01A-11R-A262-07 | 15 | NA | 278 | Primary solid Tumor |
| TCGA-95-A4VP-01A-21R-A262-07 | 15 | NA | 278 | Primary solid Tumor |
| TCGA-96-7544-01A-11R-2045-07 | 2 | 4 | 181 | Primary solid Tumor |
| TCGA-96-7545-01A-21R-2045-07 | 2 | 4 | 181 | Primary solid Tumor |
| TCGA-96-8169-01A-11R-2296-07 | 2 | NA | 225 | Primary solid Tumor |
| TCGA-96-8170-01A-11R-2296-07 | 2 | NA | 225 | Primary solid Tumor |
| TCGA-96-A4JK-01A-11R-A24Z-07 | 2 | NA | 267 | Primary solid Tumor |
| TCGA-96-A4JL-01A-11R-A24Z-07 | 2 | NA | 267 | Primary solid Tumor |
| TCGA-97-7546-01A-11R-2039-07 | 15 | 10 | 166 | Primary solid Tumor |
| TCGA-97-7547-01A-11R-2039-07 | 15 | 10 | 166 | Primary solid Tumor |
| TCGA-97-7552-01A-11R-2039-07 | 15 | 10 | 166 | Primary solid Tumor |
| TCGA-97-7553-01A-21R-2039-07 | 15 | 10 | 166 | Primary solid Tumor |
| TCGA-97-7554-01A-11R-2039-07 | 15 | 10 | 166 | Primary solid Tumor |
| TCGA-97-7937-01A-11R-2170-07 | 15 | 10 | 196 | Primary solid Tumor |
| TCGA-97-7938-01A-11R-2170-07 | 15 | 10 | 196 | Primary solid Tumor |
| TCGA-97-7941-01A-11R-2187-07 | 15 | 10 | 204 | Primary solid Tumor |
| TCGA-97-8171-01A-11R-2287-07 | 15 | 10 | 222 | Primary solid Tumor |
| TCGA-97-8172-01A-11R-2287-07 | 15 | 10 | 222 | Primary solid Tumor |
| TCGA-97-8174-01A-11R-2287-07 | 15 | 10 | 222 | Primary solid Tumor |
| TCGA-97-8175-01A-11R-2287-07 | 15 | 10 | 222 | Primary solid Tumor |
| TCGA-97-8176-01A-11R-2403-07 | 15 | NA | 238 | Primary solid Tumor |
| TCGA-97-8177-01A-11R-2287-07 | 15 | 10 | 222 | Primary solid Tumor |
| TCGA-97-8179-01A-11R-2287-07 | 15 | 10 | 222 | Primary solid Tumor |
| TCGA-97-8547-01A-11R-2403-07 | 15 | NA | 238 | Primary solid Tumor |
| TCGA-97-8552-01A-11R-2403-07 | 15 | NA | 238 | Primary solid Tumor |
| TCGA-97-A4LX-01A-11R-A24X-07 | 15 | NA | 264 | Primary solid Tumor |
| TCGA-97-A4M0-01A-11R-A24X-07 | 15 | NA | 264 | Primary solid Tumor |
| TCGA-97-A4M1-01A-11R-A24X-07 | 15 | NA | 264 | Primary solid Tumor |
| TCGA-97-A4M2-01A-12R-A24X-07 | 15 | NA | 264 | Primary solid Tumor |
| TCGA-97-A4M3-01A-11R-A24X-07 | 15 | NA | 264 | Primary solid Tumor |
| TCGA-97-A4M5-01A-11R-A24X-07 | 15 | NA | 264 | Primary solid Tumor |
| TCGA-97-A4M6-01A-11R-A24X-07 | 15 | NA | 264 | Primary solid Tumor |
| TCGA-97-A4M7-01A-11R-A24X-07 | 15 | NA | 264 | Primary solid Tumor |
| TCGA-98-7454-01A-11R-2045-07 | 15 | 10 | 181 | Primary solid Tumor |
| TCGA-98-8020-01A-11R-2247-07 | 2 | NA | 214 | Primary solid Tumor |
| TCGA-98-8021-01A-11R-2247-07 | 2 | NA | 214 | Primary solid Tumor |
| TCGA-98-8022-01A-11R-2247-07 | 2 | NA | 214 | Primary solid Tumor |
| TCGA-98-8023-01A-11R-2247-07 | 2 | NA | 214 | Primary solid Tumor |
| TCGA-98-A538-01A-11R-A262-07 | 2 | NA | 276 | Primary solid Tumor |
| TCGA-98-A539-01A-31R-A262-07 | 2 | NA | 276 | Primary solid Tumor |
| TCGA-98-A53A-01A-11R-A262-07 | 2 | NA | 276 | Primary solid Tumor |
| TCGA-98-A53B-01A-11R-A262-07 | 2 | NA | 276 | Primary solid Tumor |
| TCGA-98-A53C-01A-11R-A262-07 | 15 | NA | 276 | Primary solid Tumor |
| TCGA-98-A53D-01A-32R-A262-07 | 15 | NA | 276 | Primary solid Tumor |
| TCGA-98-A53H-01A-12R-A262-07 | 15 | NA | 276 | Primary solid Tumor |
| TCGA-98-A53I-01A-31R-A262-07 | 2 | NA | 276 | Primary solid Tumor |
| TCGA-98-A53J-01A-11R-A26W-07 | 2 | NA | 283 | Primary solid Tumor |
| TCGA-99-7458-01A-11R-2039-07 | 15 | 10 | 166 | Primary solid Tumor |
| TCGA-99-8025-01A-11R-2241-07 | 15 | 10 | 213 | Primary solid Tumor |
| TCGA-99-8028-01A-11R-2241-07 | 15 | 10 | 213 | Primary solid Tumor |
| TCGA-99-8032-01A-11R-2241-07 | 15 | 10 | 213 | Primary solid Tumor |
| TCGA-99-8033-01A-11R-2241-07 | 15 | 10 | 213 | Primary solid Tumor |
| TCGA-99-AA5R-01A-11R-A39D-07 | 15 | NA | 406 | Primary solid Tumor |
| TCGA-A1-A0SB-01A-11R-A144-07 | 10 | 8 | 117 | Primary solid Tumor |
| TCGA-A1-A0SD-01A-11R-A115-07 | 9 | 7 | 85 | Primary solid Tumor |
| TCGA-A1-A0SE-01A-11R-A084-07 | 9 | 7 | 72 | Primary solid Tumor |
| TCGA-A1-A0SF-01A-11R-A144-07 | 9 | 7 | 117 | Primary solid Tumor |
| TCGA-A1-A0SG-01A-11R-A144-07 | 9 | 7 | 117 | Primary solid Tumor |
| TCGA-A1-A0SH-01A-11R-A084-07 | 9 | 7 | 72 | Primary solid Tumor |
| TCGA-A1-A0SI-01A-11R-A144-07 | 9 | 7 | 117 | Primary solid Tumor |
| TCGA-A1-A0SJ-01A-11R-A084-07 | 9 | 7 | 72 | Primary solid Tumor |
| TCGA-A1-A0SK-01A-12R-A084-07 | 3 | 6 | 72 | Primary solid Tumor |
| TCGA-A1-A0SM-01A-11R-A084-07 | 9 | 7 | 72 | Primary solid Tumor |
| TCGA-A1-A0SN-01A-11R-A144-07 | 9 | 7 | 117 | Primary solid Tumor |
| TCGA-A1-A0SO-01A-22R-A084-07 | 10 | 8 | 72 | Primary solid Tumor |
| TCGA-A1-A0SP-01A-11R-A084-07 | 10 | 8 | 72 | Primary solid Tumor |
| TCGA-A1-A0SQ-01A-21R-A144-07 | 9 | 7 | 117 | Primary solid Tumor |
| TCGA-A2-A04N-01A-11R-A115-07 | 9 | 7 | 85 | Primary solid Tumor |
| TCGA-A2-A04P-01A-31R-A034-07 | 10 | 8 | 56 | Primary solid Tumor |
| TCGA-A2-A04Q-01A-21R-A034-07 | 10 | 8 | 56 | Primary solid Tumor |
| TCGA-A2-A04R-01A-41R-A109-07 | 9 | 7 | 74 | Primary solid Tumor |
| TCGA-A2-A04T-01A-21R-A034-07 | 10 | 8 | 56 | Primary solid Tumor |
| TCGA-A2-A04U-01A-11R-A115-07 | 10 | 8 | 85 | Primary solid Tumor |
| TCGA-A2-A04V-01A-21R-A034-07 | 9 | 7 | 56 | Primary solid Tumor |
| TCGA-A2-A04W-01A-31R-A115-07 | 9 | 7 | 85 | Primary solid Tumor |
| TCGA-A2-A04X-01A-21R-A034-07 | 9 | 7 | 56 | Primary solid Tumor |
| TCGA-A2-A04Y-01A-21R-A034-07 | 9 | 7 | 56 | Primary solid Tumor |
| TCGA-A2-A0CK-01A-11R-A22K-07 | 9 | NA | 227 | Primary solid Tumor |
| TCGA-A2-A0CL-01A-11R-A115-07 | 9 | 7 | 85 | Primary solid Tumor |
| TCGA-A2-A0CM-01A-31R-A034-07 | 10 | 8 | 56 | Primary solid Tumor |
| TCGA-A2-A0CO-01A-13R-A22K-07 | 9 | NA | 227 | Primary solid Tumor |
| TCGA-A2-A0CP-01A-11R-A034-07 | 9 | 7 | 56 | Primary solid Tumor |
| TCGA-A2-A0CQ-01A-21R-A034-07 | 9 | 7 | 56 | Primary solid Tumor |
| TCGA-A2-A0CR-01A-11R-A22K-07 | 9 | NA | 227 | Primary solid Tumor |
| TCGA-A2-A0CS-01A-11R-A115-07 | 9 | 7 | 85 | Primary solid Tumor |
| TCGA-A2-A0CT-01A-31R-A056-07 | 9 | 7 | 61 | Primary solid Tumor |
| TCGA-A2-A0CU-01A-12R-A034-07 | 9 | 7 | 56 | Primary solid Tumor |
| TCGA-A2-A0CV-01A-31R-A115-07 | 9 | 7 | 85 | Primary solid Tumor |
| TCGA-A2-A0CW-01A-21R-A115-07 | 9 | 7 | 85 | Primary solid Tumor |
| TCGA-A2-A0CX-01A-21R-A00Z-07 | 9 | 7 | 47 | Primary solid Tumor |
| TCGA-A2-A0CY-01A-12R-A034-07 | 9 | NA | 56 | Primary solid Tumor |
| TCGA-A2-A0CZ-01A-11R-A034-07 | 9 | 7 | 56 | Primary solid Tumor |
| TCGA-A2-A0D0-01A-11R-A00Z-07 | 10 | 8 | 47 | Primary solid Tumor |
| TCGA-A2-A0D1-01A-11R-A034-07 | 9 | 7 | 56 | Primary solid Tumor |
| TCGA-A2-A0D2-01A-21R-A034-07 | 10 | 8 | 56 | Primary solid Tumor |
| TCGA-A2-A0D3-01A-11R-A115-07 | 9 | 7 | 85 | Primary solid Tumor |
| TCGA-A2-A0D4-01A-11R-A00Z-07 | 9 | 7 | 47 | Primary solid Tumor |
| TCGA-A2-A0EM-01A-11R-A034-07 | 9 | 7 | 56 | Primary solid Tumor |
| TCGA-A2-A0EN-01A-13R-A084-07 | 9 | 7 | 72 | Primary solid Tumor |
| TCGA-A2-A0EO-01A-11R-A034-07 | 9 | 7 | 56 | Primary solid Tumor |
| TCGA-A2-A0EP-01A-52R-A22U-07 | 9 | NA | 234 | Primary solid Tumor |
| TCGA-A2-A0EQ-01A-11R-A034-07 | 9 | 7 | 56 | Primary solid Tumor |
| TCGA-A2-A0ER-01A-21R-A034-07 | 9 | 7 | 56 | Primary solid Tumor |
| TCGA-A2-A0ES-01A-11R-A115-07 | 9 | 7 | 85 | Primary solid Tumor |
| TCGA-A2-A0ET-01A-31R-A034-07 | 9 | 7 | 56 | Primary solid Tumor |
| TCGA-A2-A0EU-01A-22R-A056-07 | 9 | 7 | 61 | Primary solid Tumor |
| TCGA-A2-A0EV-01A-11R-A034-07 | 9 | 7 | 56 | Primary solid Tumor |
| TCGA-A2-A0EW-01A-21R-A115-07 | 9 | 7 | 85 | Primary solid Tumor |
| TCGA-A2-A0EX-01A-21R-A034-07 | 9 | 7 | 56 | Primary solid Tumor |
| TCGA-A2-A0EY-01A-11R-A034-07 | 9 | 7 | 56 | Primary solid Tumor |
| TCGA-A2-A0ST-01A-12R-A084-07 | 10 | 8 | 72 | Primary solid Tumor |
| TCGA-A2-A0SU-01A-11R-A084-07 | 9 | 7 | 72 | Primary solid Tumor |
| TCGA-A2-A0SV-01A-11R-A084-07 | 9 | 7 | 72 | Primary solid Tumor |
| TCGA-A2-A0SW-01A-11R-A084-07 | 9 | 7 | 72 | Primary solid Tumor |
| TCGA-A2-A0SX-01A-12R-A084-07 | 10 | 8 | 72 | Primary solid Tumor |
| TCGA-A2-A0SY-01A-31R-A084-07 | 9 | 7 | 72 | Primary solid Tumor |
| TCGA-A2-A0T0-01A-22R-A084-07 | 10 | 8 | 72 | Primary solid Tumor |
| TCGA-A2-A0T1-01A-21R-A084-07 | 9 | 7 | 72 | Primary solid Tumor |
| TCGA-A2-A0T2-01A-11R-A084-07 | 10 | 8 | 72 | Primary solid Tumor |
| TCGA-A2-A0T3-01A-21R-A115-07 | 9 | 7 | 85 | Primary solid Tumor |
| TCGA-A2-A0T4-01A-31R-A084-07 | 9 | 7 | 72 | Primary solid Tumor |
| TCGA-A2-A0T5-01A-21R-A084-07 | 9 | 7 | 72 | Primary solid Tumor |
| TCGA-A2-A0T6-01A-11R-A084-07 | 9 | 7 | 72 | Primary solid Tumor |
| TCGA-A2-A0T7-01A-21R-A084-07 | 9 | 7 | 72 | Primary solid Tumor |
| TCGA-A2-A0YC-01A-11R-A109-07 | 9 | 7 | 74 | Primary solid Tumor |
| TCGA-A2-A0YD-01A-11R-A109-07 | 9 | 7 | 74 | Primary solid Tumor |
| TCGA-A2-A0YE-01A-11R-A109-07 | 10 | 8 | 74 | Primary solid Tumor |
| TCGA-A2-A0YF-01A-21R-A109-07 | 9 | 7 | 74 | Primary solid Tumor |
| TCGA-A2-A0YG-01A-21R-A109-07 | 9 | 7 | 74 | Primary solid Tumor |
| TCGA-A2-A0YH-01A-11R-A109-07 | 9 | 7 | 74 | Primary solid Tumor |
| TCGA-A2-A0YI-01A-31R-A10J-07 | 9 | 7 | 80 | Primary solid Tumor |
| TCGA-A2-A0YJ-01A-11R-A109-07 | 10 | 8 | 74 | Primary solid Tumor |
| TCGA-A2-A0YK-01A-22R-A109-07 | 9 | 7 | 74 | Primary solid Tumor |
| TCGA-A2-A0YL-01A-21R-A109-07 | 9 | 7 | 74 | Primary solid Tumor |
| TCGA-A2-A0YM-01A-11R-A109-07 | 10 | 8 | 74 | Primary solid Tumor |
| TCGA-A2-A0YT-01A-11R-A109-07 | 9 | 7 | 74 | Primary solid Tumor |
| TCGA-A2-A1FV-01A-11R-A13Q-07 | 9 | 7 | 109 | Primary solid Tumor |
| TCGA-A2-A1FW-01A-11R-A13Q-07 | 9 | 7 | 109 | Primary solid Tumor |
| TCGA-A2-A1FX-01A-11R-A13Q-07 | 9 | 7 | 109 | Primary solid Tumor |
| TCGA-A2-A1FZ-01A-51R-A14D-07 | 9 | 7 | 120 | Primary solid Tumor |
| TCGA-A2-A1G0-01A-11R-A13Q-07 | 9 | 7 | 109 | Primary solid Tumor |
| TCGA-A2-A1G1-01A-21R-A13Q-07 | 10 | 8 | 109 | Primary solid Tumor |
| TCGA-A2-A1G4-01A-11R-A13Q-07 | 9 | 7 | 109 | Primary solid Tumor |
| TCGA-A2-A1G6-01A-11R-A13Q-07 | 9 | 7 | 109 | Primary solid Tumor |
| TCGA-A2-A259-01A-11R-A16F-07 | 9 | 7 | 147 | Primary solid Tumor |
| TCGA-A2-A25A-01A-12R-A16F-07 | 9 | 7 | 147 | Primary solid Tumor |
| TCGA-A2-A25B-01A-11R-A169-07 | 9 | 7 | 142 | Primary solid Tumor |
| TCGA-A2-A25C-01A-11R-A169-07 | 9 | 7 | 142 | Primary solid Tumor |
| TCGA-A2-A25D-01A-12R-A16F-07 | 9 | 7 | 147 | Primary solid Tumor |
| TCGA-A2-A25E-01A-11R-A169-07 | 9 | 7 | 142 | Primary solid Tumor |
| TCGA-A2-A25F-01A-11R-A169-07 | 10 | 8 | 142 | Primary solid Tumor |
| TCGA-A2-A3KC-01A-11R-A213-07 | 9 | 7 | 202 | Primary solid Tumor |
| TCGA-A2-A3KD-01A-12R-A213-07 | 9 | 7 | 202 | Primary solid Tumor |
| TCGA-A2-A3XS-01A-11R-A22U-07 | 10 | NA | 234 | Primary solid Tumor |
| TCGA-A2-A3XT-01A-11R-A22U-07 | 10 | NA | 234 | Primary solid Tumor |
| TCGA-A2-A3XU-01A-12R-A22U-07 | 10 | NA | 234 | Primary solid Tumor |
| TCGA-A2-A3XV-01A-21R-A239-07 | 9 | NA | 239 | Primary solid Tumor |
| TCGA-A2-A3XW-01A-11R-A239-07 | 11 | NA | 239 | Primary solid Tumor |
| TCGA-A2-A3XX-01A-21R-A239-07 | 10 | NA | 239 | Primary solid Tumor |
| TCGA-A2-A3XY-01A-11R-A239-07 | 10 | NA | 239 | Primary solid Tumor |
| TCGA-A2-A3XZ-01A-42R-A239-07 | 9 | NA | 239 | Primary solid Tumor |
| TCGA-A2-A3Y0-01A-11R-A239-07 | 10 | NA | 239 | Primary solid Tumor |
| TCGA-A2-A4RW-01A-21R-A266-07 | 9 | NA | 271 | Primary solid Tumor |
| TCGA-A2-A4RX-01A-11R-A266-07 | 10 | NA | 271 | Primary solid Tumor |
| TCGA-A2-A4RY-01A-31R-A266-07 | 9 | NA | 271 | Primary solid Tumor |
| TCGA-A2-A4S0-01A-21R-A266-07 | 9 | NA | 271 | Primary solid Tumor |
| TCGA-A2-A4S1-01A-21R-A266-07 | 3 | NA | 271 | Primary solid Tumor |
| TCGA-A2-A4S2-01A-12R-A266-07 | 9 | NA | 271 | Primary solid Tumor |
| TCGA-A2-A4S3-01A-21R-A266-07 | 9 | NA | 271 | Primary solid Tumor |
| TCGA-A3-3306-01A-01R-0864-07 | 14 | 9 | 32 | Primary solid Tumor |
| TCGA-A3-3307-01A-01R-0864-07 | 14 | 9 | 32 | Primary solid Tumor |
| TCGA-A3-3308-01A-02R-1325-07 | 14 | 9 | 32 | Primary solid Tumor |
| TCGA-A3-3311-01A-02R-1325-07 | 14 | 9 | 32 | Primary solid Tumor |
| TCGA-A3-3313-01A-02R-1325-07 | 14 | 9 | 32 | Primary solid Tumor |
| TCGA-A3-3316-01A-01R-0864-07 | 14 | 9 | 32 | Primary solid Tumor |
| TCGA-A3-3317-01A-02R-1325-07 | 14 | 9 | 32 | Primary solid Tumor |
| TCGA-A3-3319-01A-02R-1325-07 | 14 | 9 | 32 | Primary solid Tumor |
| TCGA-A3-3320-01A-02R-1325-07 | 14 | 9 | 32 | Primary solid Tumor |
| TCGA-A3-3322-01A-02R-1325-07 | 14 | 9 | 32 | Primary solid Tumor |
| TCGA-A3-3323-01A-02R-1325-07 | 14 | 9 | 32 | Primary solid Tumor |
| TCGA-A3-3324-01A-02R-1325-07 | 14 | 9 | 32 | Primary solid Tumor |
| TCGA-A3-3325-01A-01R-0864-07 | 14 | 9 | 32 | Primary solid Tumor |
| TCGA-A3-3326-01A-01R-0864-07 | 14 | 9 | 32 | Primary solid Tumor |
| TCGA-A3-3328-01A-01R-0864-07 | 14 | 9 | 32 | Primary solid Tumor |
| TCGA-A3-3329-01A-01R-0864-07 | 14 | 9 | 32 | Primary solid Tumor |
| TCGA-A3-3331-01A-02R-1325-07 | 14 | 9 | 32 | Primary solid Tumor |
| TCGA-A3-3335-01A-01R-0864-07 | 14 | 9 | 32 | Primary solid Tumor |
| TCGA-A3-3343-01A-01R-0864-07 | 14 | 9 | 32 | Primary solid Tumor |
| TCGA-A3-3346-01A-01R-1766-07 | 14 | NA | 32 | Primary solid Tumor |
| TCGA-A3-3347-01A-02R-1325-07 | 14 | 9 | 50 | Primary solid Tumor |
| TCGA-A3-3349-01A-01R-1188-07 | 14 | 9 | 50 | Primary solid Tumor |
| TCGA-A3-3351-01A-02R-1325-07 | 14 | 9 | 50 | Primary solid Tumor |
| TCGA-A3-3352-01A-01R-0864-07 | 14 | 9 | 32 | Primary solid Tumor |
| TCGA-A3-3357-01A-02R-1420-07 | 14 | 9 | 68 | Primary solid Tumor |
| TCGA-A3-3358-01A-01R-1541-07 | 14 | 9 | 90 | Primary solid Tumor |
| TCGA-A3-3359-01A-01R-0864-07 | 14 | 9 | 32 | Primary solid Tumor |
| TCGA-A3-3362-01A-02R-1325-07 | 14 | 9 | 50 | Primary solid Tumor |
| TCGA-A3-3363-01A-01R-0864-07 | 14 | 9 | 32 | Primary solid Tumor |
| TCGA-A3-3365-01A-01R-0864-07 | 14 | 9 | 32 | Primary solid Tumor |
| TCGA-A3-3367-01A-02R-1420-07 | 14 | 9 | 68 | Primary solid Tumor |
| TCGA-A3-3370-01A-02R-1420-07 | 14 | 9 | 68 | Primary solid Tumor |
| TCGA-A3-3372-01A-02R-1325-07 | 14 | 9 | 32 | Primary solid Tumor |
| TCGA-A3-3373-01A-02R-1420-07 | 14 | 9 | 68 | Primary solid Tumor |
| TCGA-A3-3374-01A-02R-1325-07 | 14 | 9 | 32 | Primary solid Tumor |
| TCGA-A3-3376-01A-02R-1420-07 | 14 | 9 | 68 | Primary solid Tumor |
| TCGA-A3-3378-01A-02R-1325-07 | 14 | 9 | 32 | Primary solid Tumor |
| TCGA-A3-3380-01A-01R-0864-07 | 14 | 9 | 32 | Primary solid Tumor |
| TCGA-A3-3382-01A-02R-1325-07 | 14 | 9 | 32 | Primary solid Tumor |
| TCGA-A3-3383-01A-02R-1325-07 | 14 | NA | 32 | Primary solid Tumor |
| TCGA-A3-3385-01A-02R-1420-07 | 14 | 9 | 68 | Primary solid Tumor |
| TCGA-A3-3387-01A-01R-1541-07 | 14 | 9 | 90 | Primary solid Tumor |
| TCGA-A3-A6NI-01A-11R-A33J-07 | 14 | NA | 340 | Primary solid Tumor |
| TCGA-A3-A6NJ-01A-12R-A33J-07 | 14 | NA | 340 | Primary solid Tumor |
| TCGA-A3-A6NL-01A-11R-A33J-07 | 14 | NA | 340 | Primary solid Tumor |
| TCGA-A3-A6NN-01A-12R-A33J-07 | 14 | NA | 340 | Primary solid Tumor |
| TCGA-A3-A8CQ-01A-11R-A37O-07 | 14 | NA | 387 | Primary solid Tumor |
| TCGA-A3-A8OU-01A-11R-A37O-07 | 14 | NA | 387 | Primary solid Tumor |
| TCGA-A3-A8OV-01A-11R-A37O-07 | 14 | NA | 387 | Primary solid Tumor |
| TCGA-A3-A8OW-01A-11R-A37O-07 | 14 | NA | 387 | Primary solid Tumor |
| TCGA-A3-A8OX-01A-11R-A37O-07 | 14 | NA | 387 | Primary solid Tumor |
| TCGA-A5-A0G1-01A-11R-A118-07 | 16 | 11 | 92 | Primary solid Tumor |
| TCGA-A5-A0G2-01A-11R-A16W-07 | 16 | 11 | 59 | Primary solid Tumor |
| TCGA-A5-A0G3-01A-11R-A040-07 | 16 | 11 | 59 | Primary solid Tumor |
| TCGA-A5-A0G5-01A-11R-A040-07 | 16 | 11 | 59 | Primary solid Tumor |
| TCGA-A5-A0G9-01A-11R-A040-07 | 16 | 11 | 59 | Primary solid Tumor |
| TCGA-A5-A0GA-01A-11R-A040-07 | 16 | 11 | 59 | Primary solid Tumor |
| TCGA-A5-A0GB-01A-11R-A040-07 | 16 | 11 | 59 | Primary solid Tumor |
| TCGA-A5-A0GD-01A-11R-A16W-07 | 16 | 11 | 59 | Primary solid Tumor |
| TCGA-A5-A0GE-01A-11R-A16W-07 | 16 | 11 | 59 | Primary solid Tumor |
| TCGA-A5-A0GG-01A-11R-A118-07 | 16 | 11 | 92 | Primary solid Tumor |
| TCGA-A5-A0GH-01A-21R-A16W-07 | 7 | 15 | 59 | Primary solid Tumor |
| TCGA-A5-A0GI-01A-11R-A040-07 | 16 | 11 | 59 | Primary solid Tumor |
| TCGA-A5-A0GJ-01A-11R-A040-07 | 16 | 11 | 59 | Primary solid Tumor |
| TCGA-A5-A0GM-01A-11R-A040-07 | 16 | 11 | 59 | Primary solid Tumor |
| TCGA-A5-A0GN-01A-11R-A040-07 | 16 | 11 | 59 | Primary solid Tumor |
| TCGA-A5-A0GP-01A-11R-A040-07 | 16 | 11 | 59 | Primary solid Tumor |
| TCGA-A5-A0GQ-01A-11R-A118-07 | 16 | 11 | 92 | Primary solid Tumor |
| TCGA-A5-A0GR-01A-11R-A118-07 | 16 | 11 | 92 | Primary solid Tumor |
| TCGA-A5-A0GU-01A-11R-A16W-07 | 16 | 11 | 59 | Primary solid Tumor |
| TCGA-A5-A0GV-01A-31R-A16W-07 | 16 | 11 | 59 | Primary solid Tumor |
| TCGA-A5-A0GW-01A-11R-A16W-07 | 16 | 11 | 59 | Primary solid Tumor |
| TCGA-A5-A0GX-01A-11R-A040-07 | 16 | 11 | 59 | Primary solid Tumor |
| TCGA-A5-A0R6-01A-11R-A104-07 | 16 | 11 | 73 | Primary solid Tumor |
| TCGA-A5-A0R7-01A-31R-A16W-07 | 16 | 11 | 73 | Primary solid Tumor |
| TCGA-A5-A0R8-01A-11R-A104-07 | 16 | 11 | 73 | Primary solid Tumor |
| TCGA-A5-A0R9-01A-11R-A104-07 | 16 | 11 | 73 | Primary solid Tumor |
| TCGA-A5-A0RA-01A-21R-A104-07 | 16 | 11 | 73 | Primary solid Tumor |
| TCGA-A5-A0VO-01A-21R-A109-07 | 16 | 11 | 75 | Primary solid Tumor |
| TCGA-A5-A0VP-01A-21R-A104-07 | 16 | 11 | 73 | Primary solid Tumor |
| TCGA-A5-A0VQ-01A-11R-A104-07 | 16 | 11 | 73 | Primary solid Tumor |
| TCGA-A5-A1OF-01A-11R-A14D-07 | 16 | 11 | 121 | Primary solid Tumor |
| TCGA-A5-A1OG-01A-11R-A14D-07 | 16 | 11 | 121 | Primary solid Tumor |
| TCGA-A5-A1OH-01A-21R-A22K-07 | 3 | NA | 228 | Primary solid Tumor |
| TCGA-A5-A1OJ-01A-11R-A14D-07 | 16 | 11 | 121 | Primary solid Tumor |
| TCGA-A5-A1OK-01A-11R-A14M-07 | 16 | 11 | 125 | Primary solid Tumor |
| TCGA-A5-A2K2-01A-11R-A18M-07 | 16 | NA | 178 | Primary solid Tumor |
| TCGA-A5-A2K3-01A-11R-A32Y-07 | 16 | NA | 168 | Primary solid Tumor |
| TCGA-A5-A2K4-01A-11R-A18M-07 | 16 | NA | 178 | Primary solid Tumor |
| TCGA-A5-A2K5-01A-11R-A180-07 | 16 | NA | 168 | Primary solid Tumor |
| TCGA-A5-A2K7-01A-11R-A180-07 | 16 | NA | 168 | Primary solid Tumor |
| TCGA-A5-A3LO-01A-11R-A22K-07 | 16 | NA | 228 | Primary solid Tumor |
| TCGA-A5-A3LP-01A-11R-A22K-07 | 20 | NA | 228 | Primary solid Tumor |
| TCGA-A5-A7WJ-01A-12R-A34R-07 | 16 | NA | 354 | Primary solid Tumor |
| TCGA-A5-A7WK-01A-11R-A34R-07 | 16 | NA | 354 | Primary solid Tumor |
| TCGA-A5-AB3J-01A-11R-A40A-07 | 16 | NA | 421 | Primary solid Tumor |
| TCGA-A6-2670-01A-02R-0821-07 | 7 | NA | 28 | Primary solid Tumor |
| TCGA-A6-2671-01A-01R-1410-07 | 7 | 15 | 76 | Primary solid Tumor |
| TCGA-A6-2672-01A-01R-0826-07 | 7 | 15 | 29 | Primary solid Tumor |
| TCGA-A6-2674-01A-02R-0821-07 | 7 | 15 | 28 | Primary solid Tumor |
| TCGA-A6-2675-01A-02R-1723-07 | 7 | NA | 116 | Primary solid Tumor |
| TCGA-A6-2676-01A-01R-0826-07 | 7 | 15 | 29 | Primary solid Tumor |
| TCGA-A6-2677-01A-01R-0821-07 | 7 | 15 | 28 | Primary solid Tumor |
| TCGA-A6-2678-01A-01R-0821-07 | 7 | 15 | 28 | Primary solid Tumor |
| TCGA-A6-2679-01A-02R-1410-07 | 7 | 15 | 76 | Primary solid Tumor |
| TCGA-A6-2680-01A-01R-1410-07 | 7 | 15 | 76 | Primary solid Tumor |
| TCGA-A6-2681-01A-01R-1410-07 | 7 | 15 | 76 | Primary solid Tumor |
| TCGA-A6-2682-01A-01R-1410-07 | 7 | 15 | 76 | Primary solid Tumor |
| TCGA-A6-2683-01A-01R-0821-07 | 7 | 15 | 28 | Primary solid Tumor |
| TCGA-A6-2684-01A-01R-1410-07 | 7 | 15 | 76 | Primary solid Tumor |
| TCGA-A6-2685-01A-01R-1410-07 | 7 | 15 | 76 | Primary solid Tumor |
| TCGA-A6-2686-01A-01R-A32Z-07 | 7 | NA | 76 | Primary solid Tumor |
| TCGA-A6-3807-01A-01R-1022-07 | 7 | 15 | 41 | Primary solid Tumor |
| TCGA-A6-3808-01A-01R-1022-07 | 7 | 15 | 41 | Primary solid Tumor |
| TCGA-A6-3809-01A-01R-1022-07 | 7 | 15 | 41 | Primary solid Tumor |
| TCGA-A6-3810-01A-01R-1022-07 | 7 | 15 | 41 | Primary solid Tumor |
| TCGA-A6-4105-01A-02R-1774-07 | 7 | NA | 123 | Primary solid Tumor |
| TCGA-A6-4107-01A-02R-1410-07 | 7 | 15 | 76 | Primary solid Tumor |
| TCGA-A6-5656-01A-21R-1839-07 | 7 | NA | 138 | Primary solid Tumor |
| TCGA-A6-5657-01A-01R-A32Z-07 | 7 | NA | 89 | Primary solid Tumor |
| TCGA-A6-5659-01A-01R-1653-07 | 7 | NA | 89 | Primary solid Tumor |
| TCGA-A6-5660-01A-01R-1653-07 | 7 | NA | 89 | Primary solid Tumor |
| TCGA-A6-5661-01A-01R-1653-07 | 7 | NA | 89 | Primary solid Tumor |
| TCGA-A6-5662-01A-01R-1653-07 | 7 | NA | 89 | Primary solid Tumor |
| TCGA-A6-5664-01A-21R-1839-07 | 7 | NA | 138 | Primary solid Tumor |
| TCGA-A6-5665-01A-01R-1653-07 | 7 | NA | 89 | Primary solid Tumor |
| TCGA-A6-5666-01A-01R-1653-07 | 7 | NA | 89 | Primary solid Tumor |
| TCGA-A6-5667-01A-21R-1723-07 | 7 | NA | 116 | Primary solid Tumor |
| TCGA-A6-6137-01A-11R-1774-07 | 7 | NA | 123 | Primary solid Tumor |
| TCGA-A6-6138-01A-11R-1774-07 | 7 | NA | 123 | Primary solid Tumor |
| TCGA-A6-6140-01A-11R-1774-07 | 7 | NA | 123 | Primary solid Tumor |
| TCGA-A6-6141-01A-11R-1774-07 | 7 | NA | 123 | Primary solid Tumor |
| TCGA-A6-6142-01A-11R-1774-07 | 7 | NA | 123 | Primary solid Tumor |
| TCGA-A6-6648-01A-11R-1774-07 | 7 | NA | 123 | Primary solid Tumor |
| TCGA-A6-6649-01A-11R-1774-07 | 7 | NA | 123 | Primary solid Tumor |
| TCGA-A6-6650-01A-11R-1774-07 | 7 | NA | 123 | Primary solid Tumor |
| TCGA-A6-6651-01A-21R-1839-07 | 7 | NA | 138 | Primary solid Tumor |
| TCGA-A6-6652-01A-11R-1774-07 | 7 | NA | 123 | Primary solid Tumor |
| TCGA-A6-6653-01A-11R-1774-07 | 7 | NA | 123 | Primary solid Tumor |
| TCGA-A6-6654-01A-21R-1839-07 | 7 | NA | 138 | Primary solid Tumor |
| TCGA-A6-6780-01A-11R-1839-07 | 7 | NA | 138 | Primary solid Tumor |
| TCGA-A6-6781-01A-22R-1928-07 | 7 | NA | 157 | Primary solid Tumor |
| TCGA-A6-6782-01A-11R-1839-07 | 7 | NA | 138 | Primary solid Tumor |
| TCGA-A6-A565-01A-31R-A28H-07 | 7 | NA | 300 | Primary solid Tumor |
| TCGA-A6-A566-01A-11R-A28H-07 | 7 | NA | 300 | Primary solid Tumor |
| TCGA-A6-A567-01A-31R-A28H-07 | 7 | NA | 300 | Primary solid Tumor |
| TCGA-A6-A56B-01A-31R-A28H-07 | 7 | NA | 300 | Primary solid Tumor |
| TCGA-A6-A5ZU-01A-11R-A28H-07 | 7 | NA | 300 | Primary solid Tumor |
| TCGA-A7-A0CD-01A-11R-A00Z-07 | 9 | 7 | 47 | Primary solid Tumor |
| TCGA-A7-A0CE-01A-11R-A00Z-07 | 10 | 8 | 47 | Primary solid Tumor |
| TCGA-A7-A0CG-01A-12R-A056-07 | 9 | 7 | 47 | Primary solid Tumor |
| TCGA-A7-A0CH-01A-21R-A00Z-07 | 9 | 7 | 47 | Primary solid Tumor |
| TCGA-A7-A0CJ-01A-21R-A00Z-07 | 9 | 7 | 47 | Primary solid Tumor |
| TCGA-A7-A0D9-01A-31R-A056-07 | 9 | 7 | 61 | Primary solid Tumor |
| TCGA-A7-A0DA-01A-31R-A115-07 | 10 | 8 | 85 | Primary solid Tumor |
| TCGA-A7-A0DB-01A-11R-A00Z-07 | 9 | 7 | 47 | Primary solid Tumor |
| TCGA-A7-A0DC-01A-11R-A00Z-07 | 9 | NA | 47 | Primary solid Tumor |
| TCGA-A7-A13D-01A-13R-A12P-07 | 10 | 8 | 96 | Primary solid Tumor |
| TCGA-A7-A13E-01A-11R-A12P-07 | 10 | 8 | 96 | Primary solid Tumor |
| TCGA-A7-A13F-01A-11R-A12P-07 | 9 | 7 | 96 | Primary solid Tumor |
| TCGA-A7-A13G-01A-11R-A13Q-07 | 9 | 7 | 109 | Primary solid Tumor |
| TCGA-A7-A13H-01A-11R-A22K-07 | 9 | NA | 227 | Primary solid Tumor |
| TCGA-A7-A26E-01A-11R-A169-07 | 9 | 7 | 142 | Primary solid Tumor |
| TCGA-A7-A26F-01A-21R-A169-07 | 10 | 8 | 142 | Primary solid Tumor |
| TCGA-A7-A26G-01A-21R-A169-07 | 10 | 8 | 142 | Primary solid Tumor |
| TCGA-A7-A26H-01A-11R-A169-07 | 9 | 7 | 142 | Primary solid Tumor |
| TCGA-A7-A26I-01A-11R-A169-07 | 10 | 8 | 142 | Primary solid Tumor |
| TCGA-A7-A26J-01A-11R-A169-07 | 9 | 7 | 142 | Primary solid Tumor |
| TCGA-A7-A2KD-01A-31R-A21T-07 | 9 | 7 | 216 | Primary solid Tumor |
| TCGA-A7-A3IY-01A-21R-A21T-07 | 9 | 7 | 216 | Primary solid Tumor |
| TCGA-A7-A3IZ-01A-11R-A213-07 | 9 | 7 | 202 | Primary solid Tumor |
| TCGA-A7-A3J0-01A-11R-A213-07 | 9 | 7 | 202 | Primary solid Tumor |
| TCGA-A7-A3J1-01A-11R-A213-07 | 9 | 7 | 202 | Primary solid Tumor |
| TCGA-A7-A3RF-01A-11R-A22K-07 | 9 | NA | 227 | Primary solid Tumor |
| TCGA-A7-A425-01A-11R-A24H-07 | 9 | NA | 255 | Primary solid Tumor |
| TCGA-A7-A426-01A-22R-A24H-07 | 9 | NA | 255 | Primary solid Tumor |
| TCGA-A7-A4SA-01A-11R-A266-07 | 9 | NA | 271 | Primary solid Tumor |
| TCGA-A7-A4SB-01A-21R-A266-07 | 9 | NA | 271 | Primary solid Tumor |
| TCGA-A7-A4SC-01A-12R-A266-07 | 9 | NA | 271 | Primary solid Tumor |
| TCGA-A7-A4SD-01A-11R-A266-07 | 10 | NA | 271 | Primary solid Tumor |
| TCGA-A7-A4SE-01A-11R-A266-07 | 10 | NA | 271 | Primary solid Tumor |
| TCGA-A7-A4SF-01A-11R-A266-07 | 9 | NA | 271 | Primary solid Tumor |
| TCGA-A7-A56D-01A-11R-A27Q-07 | 9 | NA | 288 | Primary solid Tumor |
| TCGA-A7-A5ZV-01A-11R-A28M-07 | 10 | NA | 296 | Primary solid Tumor |
| TCGA-A7-A5ZW-01A-12R-A29R-07 | 9 | NA | 305 | Primary solid Tumor |
| TCGA-A7-A5ZX-01A-12R-A29R-07 | 9 | NA | 305 | Primary solid Tumor |
| TCGA-A7-A6VV-01A-22R-A33J-07 | 10 | NA | 338 | Primary solid Tumor |
| TCGA-A7-A6VW-01A-21R-A33J-07 | 10 | NA | 338 | Primary solid Tumor |
| TCGA-A7-A6VX-01A-12R-A33J-07 | 9 | NA | 338 | Primary solid Tumor |
| TCGA-A7-A6VY-01A-12R-A33J-07 | 10 | NA | 338 | Primary solid Tumor |
| TCGA-A8-A06N-01A-11R-A00Z-07 | 9 | 7 | 47 | Primary solid Tumor |
| TCGA-A8-A06O-01A-11R-A00Z-07 | 9 | 7 | 47 | Primary solid Tumor |
| TCGA-A8-A06P-01A-11R-A00Z-07 | 9 | 7 | 47 | Primary solid Tumor |
| TCGA-A8-A06Q-01A-11R-A034-07 | 9 | 7 | 56 | Primary solid Tumor |
| TCGA-A8-A06R-01A-11R-A00Z-07 | 9 | 7 | 47 | Primary solid Tumor |
| TCGA-A8-A06T-01A-11R-A00Z-07 | 9 | 7 | 47 | Primary solid Tumor |
| TCGA-A8-A06U-01A-11R-A00Z-07 | 9 | 7 | 47 | Primary solid Tumor |
| TCGA-A8-A06X-01A-21R-A00Z-07 | 9 | 7 | 47 | Primary solid Tumor |
| TCGA-A8-A06Y-01A-21R-A00Z-07 | 9 | 7 | 47 | Primary solid Tumor |
| TCGA-A8-A06Z-01A-11R-A00Z-07 | 9 | 7 | 47 | Primary solid Tumor |
| TCGA-A8-A075-01A-11R-A084-07 | 9 | 7 | 72 | Primary solid Tumor |
| TCGA-A8-A076-01A-21R-A00Z-07 | 9 | 7 | 47 | Primary solid Tumor |
| TCGA-A8-A079-01A-21R-A00Z-07 | 9 | 7 | 47 | Primary solid Tumor |
| TCGA-A8-A07B-01A-11R-A00Z-07 | 9 | 7 | 47 | Primary solid Tumor |
| TCGA-A8-A07C-01A-11R-A034-07 | 10 | 8 | 56 | Primary solid Tumor |
| TCGA-A8-A07E-01A-11R-A034-07 | 9 | 7 | 56 | Primary solid Tumor |
| TCGA-A8-A07F-01A-11R-A00Z-07 | 9 | 7 | 47 | Primary solid Tumor |
| TCGA-A8-A07G-01A-11R-A034-07 | 9 | 7 | 56 | Primary solid Tumor |
| TCGA-A8-A07I-01A-11R-A00Z-07 | 9 | 7 | 47 | Primary solid Tumor |
| TCGA-A8-A07J-01A-11R-A00Z-07 | 9 | 7 | 47 | Primary solid Tumor |
| TCGA-A8-A07L-01A-11R-A00Z-07 | 9 | 7 | 47 | Primary solid Tumor |
| TCGA-A8-A07O-01A-11R-A00Z-07 | 10 | 8 | 47 | Primary solid Tumor |
| TCGA-A8-A07P-01A-11R-A00Z-07 | 9 | 7 | 47 | Primary solid Tumor |
| TCGA-A8-A07R-01A-21R-A034-07 | 10 | 8 | 56 | Primary solid Tumor |
| TCGA-A8-A07S-01A-11R-A034-07 | 9 | 7 | 56 | Primary solid Tumor |
| TCGA-A8-A07U-01A-11R-A034-07 | 10 | 8 | 56 | Primary solid Tumor |
| TCGA-A8-A07W-01A-11R-A00Z-07 | 9 | 7 | 47 | Primary solid Tumor |
| TCGA-A8-A07Z-01A-11R-A00Z-07 | 9 | NA | 47 | Primary solid Tumor |
| TCGA-A8-A081-01A-11R-A00Z-07 | 9 | 7 | 47 | Primary solid Tumor |
| TCGA-A8-A082-01A-11R-A00Z-07 | 9 | 7 | 47 | Primary solid Tumor |
| TCGA-A8-A083-01A-21R-A00Z-07 | 9 | 7 | 47 | Primary solid Tumor |
| TCGA-A8-A084-01A-21R-A00Z-07 | 9 | 7 | 47 | Primary solid Tumor |
| TCGA-A8-A085-01A-11R-A00Z-07 | 9 | 7 | 47 | Primary solid Tumor |
| TCGA-A8-A086-01A-11R-A00Z-07 | 9 | 7 | 47 | Primary solid Tumor |
| TCGA-A8-A08A-01A-11R-A32Y-07 | 9 | NA | 47 | Primary solid Tumor |
| TCGA-A8-A08B-01A-11R-A00Z-07 | 9 | 7 | 47 | Primary solid Tumor |
| TCGA-A8-A08C-01A-11R-A00Z-07 | 9 | 7 | 47 | Primary solid Tumor |
| TCGA-A8-A08F-01A-11R-A00Z-07 | 9 | 7 | 47 | Primary solid Tumor |
| TCGA-A8-A08G-01A-11R-A00Z-07 | 9 | 7 | 47 | Primary solid Tumor |
| TCGA-A8-A08H-01A-21R-A00Z-07 | 9 | 7 | 47 | Primary solid Tumor |
| TCGA-A8-A08I-01A-11R-A00Z-07 | 9 | 7 | 47 | Primary solid Tumor |
| TCGA-A8-A08J-01A-11R-A00Z-07 | 9 | 7 | 47 | Primary solid Tumor |
| TCGA-A8-A08L-01A-11R-A00Z-07 | 9 | 7 | 47 | Primary solid Tumor |
| TCGA-A8-A08O-01A-21R-A056-07 | 9 | 7 | 61 | Primary solid Tumor |
| TCGA-A8-A08P-01A-11R-A00Z-07 | 9 | 7 | 47 | Primary solid Tumor |
| TCGA-A8-A08R-01A-11R-A034-07 | 10 | 8 | 56 | Primary solid Tumor |
| TCGA-A8-A08S-01A-11R-A034-07 | 9 | 7 | 56 | Primary solid Tumor |
| TCGA-A8-A08T-01A-21R-A00Z-07 | 9 | 7 | 47 | Primary solid Tumor |
| TCGA-A8-A08X-01A-21R-A00Z-07 | 9 | 7 | 47 | Primary solid Tumor |
| TCGA-A8-A08Z-01A-21R-A00Z-07 | 9 | 7 | 47 | Primary solid Tumor |
| TCGA-A8-A090-01A-11R-A00Z-07 | 9 | 7 | 47 | Primary solid Tumor |
| TCGA-A8-A091-01A-11R-A00Z-07 | 9 | 7 | 47 | Primary solid Tumor |
| TCGA-A8-A092-01A-11R-A00Z-07 | 9 | 7 | 47 | Primary solid Tumor |
| TCGA-A8-A093-01A-11R-A00Z-07 | 9 | 7 | 47 | Primary solid Tumor |
| TCGA-A8-A094-01A-11R-A00Z-07 | 9 | 7 | 47 | Primary solid Tumor |
| TCGA-A8-A095-01A-11R-A00Z-07 | 9 | 7 | 47 | Primary solid Tumor |
| TCGA-A8-A096-01A-11R-A00Z-07 | 9 | 7 | 47 | Primary solid Tumor |
| TCGA-A8-A097-01A-11R-A034-07 | 9 | 7 | 56 | Primary solid Tumor |
| TCGA-A8-A099-01A-11R-A00Z-07 | 9 | 7 | 47 | Primary solid Tumor |
| TCGA-A8-A09A-01A-11R-A00Z-07 | 9 | 7 | 47 | Primary solid Tumor |
| TCGA-A8-A09B-01A-11R-A00Z-07 | 9 | 7 | 47 | Primary solid Tumor |
| TCGA-A8-A09C-01A-11R-A00Z-07 | 9 | 7 | 47 | Primary solid Tumor |
| TCGA-A8-A09D-01A-11R-A00Z-07 | 9 | 7 | 47 | Primary solid Tumor |
| TCGA-A8-A09E-01A-11R-A00Z-07 | 9 | NA | 47 | Primary solid Tumor |
| TCGA-A8-A09G-01A-21R-A00Z-07 | 9 | 7 | 47 | Primary solid Tumor |
| TCGA-A8-A09I-01A-22R-A034-07 | 9 | 7 | 56 | Primary solid Tumor |
| TCGA-A8-A09K-01A-11R-A00Z-07 | 9 | 7 | 47 | Primary solid Tumor |
| TCGA-A8-A09M-01A-11R-A00Z-07 | 9 | 7 | 47 | Primary solid Tumor |
| TCGA-A8-A09N-01A-11R-A00Z-07 | 9 | 7 | 47 | Primary solid Tumor |
| TCGA-A8-A09Q-01A-11R-A00Z-07 | 9 | 7 | 47 | Primary solid Tumor |
| TCGA-A8-A09R-01A-11R-A00Z-07 | 9 | 7 | 47 | Primary solid Tumor |
| TCGA-A8-A09T-01A-11R-A00Z-07 | 9 | 7 | 47 | Primary solid Tumor |
| TCGA-A8-A09V-01A-11R-A034-07 | 9 | 7 | 56 | Primary solid Tumor |
| TCGA-A8-A09W-01A-11R-A00Z-07 | 9 | 7 | 47 | Primary solid Tumor |
| TCGA-A8-A09X-01A-11R-A00Z-07 | 9 | 7 | 47 | Primary solid Tumor |
| TCGA-A8-A09Z-01A-11R-A00Z-07 | 9 | NA | 47 | Primary solid Tumor |
| TCGA-A8-A0A1-01A-11R-A00Z-07 | 9 | 7 | 47 | Primary solid Tumor |
| TCGA-A8-A0A2-01A-11R-A034-07 | 9 | 7 | 56 | Primary solid Tumor |
| TCGA-A8-A0A4-01A-11R-A00Z-07 | 9 | 7 | 47 | Primary solid Tumor |
| TCGA-A8-A0A6-01A-12R-A056-07 | 9 | 7 | 61 | Primary solid Tumor |
| TCGA-A8-A0A7-01A-11R-A00Z-07 | 9 | 7 | 47 | Primary solid Tumor |
| TCGA-A8-A0A9-01A-11R-A00Z-07 | 9 | 7 | 47 | Primary solid Tumor |
| TCGA-A8-A0AB-01A-11R-A034-07 | 9 | 7 | 56 | Primary solid Tumor |
| TCGA-A8-A0AD-01A-11R-A056-07 | 9 | 7 | 61 | Primary solid Tumor |
| TCGA-AA-3488-01A-01R-1410-07 | 7 | 15 | 76 | Primary solid Tumor |
| TCGA-AA-3489-01A-21R-1839-07 | 7 | NA | 138 | Primary solid Tumor |
| TCGA-AA-3492-01A-01R-1410-07 | 7 | 15 | 76 | Primary solid Tumor |
| TCGA-AA-3494-01A-01R-1410-07 | 7 | 15 | 76 | Primary solid Tumor |
| TCGA-AA-3495-01A-01R-1410-07 | 7 | 15 | 76 | Primary solid Tumor |
| TCGA-AA-3496-01A-21R-1839-07 | 7 | NA | 138 | Primary solid Tumor |
| TCGA-AA-3502-01A-01R-1410-07 | 7 | 15 | 76 | Primary solid Tumor |
| TCGA-AA-3506-01A-01R-1410-07 | 7 | 15 | 76 | Primary solid Tumor |
| TCGA-AA-3509-01A-01R-1410-07 | 7 | 15 | 76 | Primary solid Tumor |
| TCGA-AA-3510-01A-01R-1410-07 | 7 | NA | 76 | Primary solid Tumor |
| TCGA-AA-3511-01A-21R-1839-07 | 7 | NA | 138 | Primary solid Tumor |
| TCGA-AA-3514-01A-02R-0821-07 | 7 | 15 | 28 | Primary solid Tumor |
| TCGA-AA-3516-01A-02R-0826-07 | 7 | 15 | 29 | Primary solid Tumor |
| TCGA-AA-3517-01A-01R-0821-07 | 7 | 15 | 28 | Primary solid Tumor |
| TCGA-AA-3518-01A-02R-0826-07 | 7 | 15 | 29 | Primary solid Tumor |
| TCGA-AA-3519-01A-02R-0821-07 | 7 | 15 | 28 | Primary solid Tumor |
| TCGA-AA-3520-01A-01R-0821-07 | 7 | 15 | 28 | Primary solid Tumor |
| TCGA-AA-3521-01A-01R-0821-07 | 7 | 15 | 28 | Primary solid Tumor |
| TCGA-AA-3522-01A-01R-0821-07 | 7 | 15 | 28 | Primary solid Tumor |
| TCGA-AA-3524-01A-02R-0821-07 | 7 | 15 | 28 | Primary solid Tumor |
| TCGA-AA-3525-01A-02R-0826-07 | 7 | 15 | 29 | Primary solid Tumor |
| TCGA-AA-3526-01A-02R-A32Z-07 | 7 | NA | 28 | Primary solid Tumor |
| TCGA-AA-3527-01A-01R-0821-07 | 7 | 15 | 28 | Primary solid Tumor |
| TCGA-AA-3529-01A-02R-0821-07 | 7 | 15 | 28 | Primary solid Tumor |
| TCGA-AA-3530-01A-01R-1022-07 | 7 | 15 | 41 | Primary solid Tumor |
| TCGA-AA-3531-01A-01R-0821-07 | 7 | 15 | 28 | Primary solid Tumor |
| TCGA-AA-3532-01A-01R-0821-07 | 7 | 15 | 28 | Primary solid Tumor |
| TCGA-AA-3534-01A-01R-0821-07 | 7 | 15 | 28 | Primary solid Tumor |
| TCGA-AA-3538-01A-01R-0821-07 | 7 | 15 | 28 | Primary solid Tumor |
| TCGA-AA-3542-01A-02R-1873-07 | 7 | 15 | 28 | Primary solid Tumor |
| TCGA-AA-3543-01A-01R-0826-07 | 7 | 15 | 29 | Primary solid Tumor |
| TCGA-AA-3544-01A-01R-1873-07 | 7 | 15 | 28 | Primary solid Tumor |
| TCGA-AA-3548-01A-01R-1873-07 | 7 | 15 | 28 | Primary solid Tumor |
| TCGA-AA-3549-01A-02R-0821-07 | 7 | 15 | 28 | Primary solid Tumor |
| TCGA-AA-3552-01A-01R-0821-07 | 7 | 15 | 28 | Primary solid Tumor |
| TCGA-AA-3553-01A-01R-0821-07 | 7 | 15 | 28 | Primary solid Tumor |
| TCGA-AA-3554-01A-01R-0826-07 | 7 | 15 | 29 | Primary solid Tumor |
| TCGA-AA-3555-01A-01R-0821-07 | 7 | 15 | 28 | Primary solid Tumor |
| TCGA-AA-3556-01A-01R-0821-07 | 7 | 15 | 28 | Primary solid Tumor |
| TCGA-AA-3560-01A-01R-0821-07 | 7 | 15 | 28 | Primary solid Tumor |
| TCGA-AA-3561-01A-01R-0821-07 | 7 | 15 | 28 | Primary solid Tumor |
| TCGA-AA-3562-01A-02R-0821-07 | 7 | 15 | 28 | Primary solid Tumor |
| TCGA-AA-3655-01A-02R-1723-07 | 7 | NA | 116 | Primary solid Tumor |
| TCGA-AA-3660-01A-01R-1723-07 | 7 | NA | 116 | Primary solid Tumor |
| TCGA-AA-3662-01A-01R-1723-07 | 7 | NA | 116 | Primary solid Tumor |
| TCGA-AA-3663-01A-01R-1723-07 | 7 | NA | 116 | Primary solid Tumor |
| TCGA-AA-3664-01A-01R-0905-07 | 7 | 15 | 36 | Primary solid Tumor |
| TCGA-AA-3666-01A-02R-0905-07 | 7 | 15 | 36 | Primary solid Tumor |
| TCGA-AA-3667-01A-01R-0905-07 | 7 | 15 | 36 | Primary solid Tumor |
| TCGA-AA-3672-01A-01R-0905-07 | 7 | 15 | 36 | Primary solid Tumor |
| TCGA-AA-3673-01A-01R-0905-07 | 7 | 15 | 36 | Primary solid Tumor |
| TCGA-AA-3675-01A-02R-0905-07 | 7 | NA | 36 | Primary solid Tumor |
| TCGA-AA-3678-01A-01R-0905-07 | 7 | 15 | 36 | Primary solid Tumor |
| TCGA-AA-3679-01A-02R-0905-07 | 7 | 15 | 36 | Primary solid Tumor |
| TCGA-AA-3680-01A-01R-0905-07 | 7 | 15 | 36 | Primary solid Tumor |
| TCGA-AA-3681-01A-01R-0905-07 | 7 | 15 | 36 | Primary solid Tumor |
| TCGA-AA-3684-01A-02R-0905-07 | 7 | 15 | 36 | Primary solid Tumor |
| TCGA-AA-3685-01A-02R-A32Z-07 | 7 | NA | 36 | Primary solid Tumor |
| TCGA-AA-3688-01A-01R-0905-07 | 7 | 15 | 36 | Primary solid Tumor |
| TCGA-AA-3692-01A-01R-0905-07 | 7 | 15 | 36 | Primary solid Tumor |
| TCGA-AA-3693-01A-01R-0905-07 | 7 | 15 | 36 | Primary solid Tumor |
| TCGA-AA-3695-01A-01R-0905-07 | 3 | NA | 36 | Primary solid Tumor |
| TCGA-AA-3696-01A-01R-0905-07 | 7 | 15 | 36 | Primary solid Tumor |
| TCGA-AA-3697-01A-01R-1723-07 | 7 | NA | 116 | Primary solid Tumor |
| TCGA-AA-3710-01A-01R-1022-07 | 7 | 15 | 41 | Primary solid Tumor |
| TCGA-AA-3712-01A-21R-1723-07 | 7 | NA | 116 | Primary solid Tumor |
| TCGA-AA-3713-01A-21R-1723-07 | 7 | NA | 116 | Primary solid Tumor |
| TCGA-AA-3715-01A-01R-0905-07 | 7 | 15 | 36 | Primary solid Tumor |
| TCGA-AA-3811-01A-01R-1022-07 | 7 | 15 | 41 | Primary solid Tumor |
| TCGA-AA-3812-01A-01R-0905-07 | 7 | 15 | 36 | Primary solid Tumor |
| TCGA-AA-3814-01A-01R-0905-07 | 7 | 15 | 36 | Primary solid Tumor |
| TCGA-AA-3815-01A-01R-1022-07 | 7 | 15 | 41 | Primary solid Tumor |
| TCGA-AA-3818-01A-01R-0905-07 | 7 | 15 | 36 | Primary solid Tumor |
| TCGA-AA-3819-01A-01R-0905-07 | 7 | 15 | 36 | Primary solid Tumor |
| TCGA-AA-3821-01A-01R-1022-07 | 7 | 15 | 41 | Primary solid Tumor |
| TCGA-AA-3831-01A-01R-0905-07 | 7 | 15 | 36 | Primary solid Tumor |
| TCGA-AA-3833-01A-01R-0905-07 | 7 | 15 | 36 | Primary solid Tumor |
| TCGA-AA-3837-01A-01R-0905-07 | 7 | 15 | 36 | Primary solid Tumor |
| TCGA-AA-3841-01A-01R-0905-07 | 7 | 15 | 36 | Primary solid Tumor |
| TCGA-AA-3842-01A-01R-1022-07 | 7 | 15 | 41 | Primary solid Tumor |
| TCGA-AA-3844-01A-01R-1022-07 | 7 | 15 | 41 | Primary solid Tumor |
| TCGA-AA-3845-01A-01R-1022-07 | 7 | 15 | 41 | Primary solid Tumor |
| TCGA-AA-3846-01A-01R-1022-07 | 7 | 15 | 41 | Primary solid Tumor |
| TCGA-AA-3848-01A-01R-0905-07 | 7 | 15 | 36 | Primary solid Tumor |
| TCGA-AA-3850-01A-01R-1022-07 | 7 | 15 | 41 | Primary solid Tumor |
| TCGA-AA-3851-01A-01R-1022-07 | 7 | 15 | 41 | Primary solid Tumor |
| TCGA-AA-3852-01A-01R-0905-07 | 7 | 15 | 36 | Primary solid Tumor |
| TCGA-AA-3854-01A-01R-0905-07 | 7 | 15 | 36 | Primary solid Tumor |
| TCGA-AA-3855-01A-01R-1022-07 | 7 | 15 | 41 | Primary solid Tumor |
| TCGA-AA-3856-01A-01R-0905-07 | 7 | 15 | 36 | Primary solid Tumor |
| TCGA-AA-3858-01A-01R-0905-07 | 7 | 15 | 36 | Primary solid Tumor |
| TCGA-AA-3860-01A-02R-0905-07 | 7 | 15 | 36 | Primary solid Tumor |
| TCGA-AA-3861-01A-01R-1022-07 | 7 | 15 | 41 | Primary solid Tumor |
| TCGA-AA-3862-01A-01R-1022-07 | 7 | 15 | 41 | Primary solid Tumor |
| TCGA-AA-3864-01A-01R-1022-07 | 7 | 15 | 41 | Primary solid Tumor |
| TCGA-AA-3866-01A-01R-1022-07 | 7 | 15 | 41 | Primary solid Tumor |
| TCGA-AA-3867-01A-01R-1022-07 | 7 | 15 | 41 | Primary solid Tumor |
| TCGA-AA-3869-01A-01R-1022-07 | 7 | 15 | 41 | Primary solid Tumor |
| TCGA-AA-3870-01A-01R-1022-07 | 7 | 15 | 41 | Primary solid Tumor |
| TCGA-AA-3872-01A-01R-1022-07 | 7 | 15 | 41 | Primary solid Tumor |
| TCGA-AA-3875-01A-01R-0905-07 | 7 | 15 | 36 | Primary solid Tumor |
| TCGA-AA-3877-01A-01R-1022-07 | 7 | 15 | 41 | Primary solid Tumor |
| TCGA-AA-3930-01A-01R-1022-07 | 7 | 15 | 41 | Primary solid Tumor |
| TCGA-AA-3939-01A-01R-1022-07 | 7 | 15 | 41 | Primary solid Tumor |
| TCGA-AA-3941-01A-01R-1022-07 | 7 | 15 | 41 | Primary solid Tumor |
| TCGA-AA-3947-01A-01R-1022-07 | 7 | 15 | 41 | Primary solid Tumor |
| TCGA-AA-3949-01A-01R-1022-07 | 7 | 15 | 41 | Primary solid Tumor |
| TCGA-AA-3950-01A-02R-1022-07 | 7 | 15 | 41 | Primary solid Tumor |
| TCGA-AA-3952-01A-01R-1022-07 | 7 | 15 | 41 | Primary solid Tumor |
| TCGA-AA-3955-01A-02R-1022-07 | 7 | 15 | 41 | Primary solid Tumor |
| TCGA-AA-3956-01A-02R-1022-07 | 7 | 15 | 41 | Primary solid Tumor |
| TCGA-AA-3966-01A-01R-1113-07 | 7 | 15 | 45 | Primary solid Tumor |
| TCGA-AA-3968-01A-01R-1022-07 | 7 | 15 | 41 | Primary solid Tumor |
| TCGA-AA-3970-01A-01R-1022-07 | 7 | 15 | 41 | Primary solid Tumor |
| TCGA-AA-3971-01A-01R-1022-07 | 7 | 15 | 41 | Primary solid Tumor |
| TCGA-AA-3972-01A-01R-1022-07 | 7 | 15 | 41 | Primary solid Tumor |
| TCGA-AA-3973-01A-01R-1022-07 | 7 | 15 | 41 | Primary solid Tumor |
| TCGA-AA-3975-01A-01R-1022-07 | 7 | 15 | 41 | Primary solid Tumor |
| TCGA-AA-3976-01A-01R-1022-07 | 7 | 15 | 41 | Primary solid Tumor |
| TCGA-AA-3977-01A-01R-1022-07 | 7 | 15 | 41 | Primary solid Tumor |
| TCGA-AA-3979-01A-01R-1022-07 | 7 | 15 | 41 | Primary solid Tumor |
| TCGA-AA-3980-01A-02R-1022-07 | 7 | 15 | 41 | Primary solid Tumor |
| TCGA-AA-3982-01A-02R-1022-07 | 7 | 15 | 41 | Primary solid Tumor |
| TCGA-AA-3984-01A-02R-1022-07 | 7 | 15 | 41 | Primary solid Tumor |
| TCGA-AA-3986-01A-02R-1022-07 | 7 | 15 | 41 | Primary solid Tumor |
| TCGA-AA-3989-01A-01R-1022-07 | 7 | 15 | 41 | Primary solid Tumor |
| TCGA-AA-3994-01A-01R-1113-07 | 7 | 15 | 45 | Primary solid Tumor |
| TCGA-AA-A004-01A-01R-A00A-07 | 7 | 15 | 33 | Primary solid Tumor |
| TCGA-AA-A00A-01A-01R-A002-07 | 7 | 15 | 30 | Primary solid Tumor |
| TCGA-AA-A00D-01A-01R-A002-07 | 7 | 15 | 30 | Primary solid Tumor |
| TCGA-AA-A00E-01A-01R-A002-07 | 7 | 15 | 30 | Primary solid Tumor |
| TCGA-AA-A00F-01A-01R-A002-07 | 7 | 15 | 30 | Primary solid Tumor |
| TCGA-AA-A00J-01A-02R-A002-07 | 7 | 15 | 30 | Primary solid Tumor |
| TCGA-AA-A00K-01A-02R-A002-07 | 7 | 15 | 30 | Primary solid Tumor |
| TCGA-AA-A00L-01A-01R-A002-07 | 7 | 15 | 30 | Primary solid Tumor |
| TCGA-AA-A00N-01A-02R-A00A-07 | 7 | 15 | 33 | Primary solid Tumor |
| TCGA-AA-A00O-01A-02R-A089-07 | 7 | 15 | 33 | Primary solid Tumor |
| TCGA-AA-A00Q-01A-01R-A002-07 | 7 | 15 | 30 | Primary solid Tumor |
| TCGA-AA-A00R-01A-01R-A002-07 | 7 | 15 | 30 | Primary solid Tumor |
| TCGA-AA-A00U-01A-01R-A002-07 | 7 | 15 | 30 | Primary solid Tumor |
| TCGA-AA-A00W-01A-01R-A002-07 | 7 | 15 | 30 | Primary solid Tumor |
| TCGA-AA-A00Z-01A-01R-A002-07 | 7 | 15 | 30 | Primary solid Tumor |
| TCGA-AA-A010-01A-01R-A089-07 | 7 | 15 | 33 | Primary solid Tumor |
| TCGA-AA-A017-01A-01R-A00A-07 | 7 | 15 | 33 | Primary solid Tumor |
| TCGA-AA-A01C-01A-01R-A00A-07 | 7 | 15 | 33 | Primary solid Tumor |
| TCGA-AA-A01D-01A-01R-A00A-07 | 7 | 15 | 33 | Primary solid Tumor |
| TCGA-AA-A01F-01A-01R-A002-07 | 7 | 15 | 30 | Primary solid Tumor |
| TCGA-AA-A01G-01A-01R-A002-07 | 7 | 15 | 30 | Primary solid Tumor |
| TCGA-AA-A01I-01A-02R-A089-07 | 7 | 15 | 33 | Primary solid Tumor |
| TCGA-AA-A01K-01A-01R-A00A-07 | 7 | 15 | 33 | Primary solid Tumor |
| TCGA-AA-A01P-01A-21R-A083-07 | 7 | 15 | 66 | Primary solid Tumor |
| TCGA-AA-A01Q-01A-01R-A002-07 | 7 | 15 | 30 | Primary solid Tumor |
| TCGA-AA-A01R-01A-21R-A083-07 | 7 | 15 | 66 | Primary solid Tumor |
| TCGA-AA-A01S-01A-21R-A083-07 | 7 | 15 | 66 | Primary solid Tumor |
| TCGA-AA-A01T-01A-21R-A16W-07 | 7 | 15 | 66 | Primary solid Tumor |
| TCGA-AA-A01V-01A-23R-A083-07 | 7 | 15 | 66 | Primary solid Tumor |
| TCGA-AA-A01X-01A-21R-A083-07 | 7 | 15 | 66 | Primary solid Tumor |
| TCGA-AA-A01Z-01A-11R-A083-07 | 7 | 15 | 66 | Primary solid Tumor |
| TCGA-AA-A022-01A-21R-A16W-07 | 7 | 15 | 66 | Primary solid Tumor |
| TCGA-AA-A024-01A-02R-A00A-07 | 7 | 15 | 33 | Primary solid Tumor |
| TCGA-AA-A029-01A-01R-A00A-07 | 7 | 15 | 33 | Primary solid Tumor |
| TCGA-AA-A02E-01A-01R-A00A-07 | 7 | 15 | 33 | Primary solid Tumor |
| TCGA-AA-A02F-01A-01R-A089-07 | 7 | 15 | 33 | Primary solid Tumor |
| TCGA-AA-A02H-01A-01R-A089-07 | 7 | 15 | 33 | Primary solid Tumor |
| TCGA-AA-A02J-01A-01R-A00A-07 | 7 | 15 | 33 | Primary solid Tumor |
| TCGA-AA-A02K-01A-03R-A32Y-07 | 7 | NA | 66 | Primary solid Tumor |
| TCGA-AA-A02O-01A-21R-A16W-07 | 7 | 15 | 66 | Primary solid Tumor |
| TCGA-AA-A02R-01A-01R-A00A-07 | 7 | 15 | 33 | Primary solid Tumor |
| TCGA-AA-A02W-01A-01R-A00A-07 | 7 | 15 | 33 | Primary solid Tumor |
| TCGA-AA-A02Y-01A-43R-A32Y-07 | 7 | NA | 66 | Primary solid Tumor |
| TCGA-AA-A03F-01A-11R-A16W-07 | 7 | 15 | 66 | Primary solid Tumor |
| TCGA-AA-A03J-01A-21R-A16W-07 | 7 | 15 | 66 | Primary solid Tumor |
| TCGA-AB-2803-03A-01T-0734-13 | 6 | 13 | 25 | Primary Blood Derived Cancer - Peripheral Blood |
| TCGA-AB-2805-03A-01T-0734-13 | 6 | 13 | 25 | Primary Blood Derived Cancer - Peripheral Blood |
| TCGA-AB-2806-03A-01T-0734-13 | 6 | 13 | 25 | Primary Blood Derived Cancer - Peripheral Blood |
| TCGA-AB-2807-03A-01T-0734-13 | 6 | 13 | 25 | Primary Blood Derived Cancer - Peripheral Blood |
| TCGA-AB-2808-03A-01T-0734-13 | 6 | 13 | 25 | Primary Blood Derived Cancer - Peripheral Blood |
| TCGA-AB-2810-03A-01T-0736-13 | 6 | 13 | 25 | Primary Blood Derived Cancer - Peripheral Blood |
| TCGA-AB-2811-03B-01T-0760-13 | 6 | 13 | 25 | Primary Blood Derived Cancer - Peripheral Blood |
| TCGA-AB-2812-03A-01T-0734-13 | 6 | 13 | 25 | Primary Blood Derived Cancer - Peripheral Blood |
| TCGA-AB-2813-03A-01T-0736-13 | 6 | 13 | 25 | Primary Blood Derived Cancer - Peripheral Blood |
| TCGA-AB-2814-03A-01T-0734-13 | 6 | 13 | 25 | Primary Blood Derived Cancer - Peripheral Blood |
| TCGA-AB-2815-03A-01T-0734-13 | 6 | 13 | 25 | Primary Blood Derived Cancer - Peripheral Blood |
| TCGA-AB-2816-03A-01T-0734-13 | 6 | 13 | 25 | Primary Blood Derived Cancer - Peripheral Blood |
| TCGA-AB-2817-03A-01T-0736-13 | 6 | 13 | 25 | Primary Blood Derived Cancer - Peripheral Blood |
| TCGA-AB-2818-03A-01T-0734-13 | 6 | 13 | 25 | Primary Blood Derived Cancer - Peripheral Blood |
| TCGA-AB-2819-03A-01T-0734-13 | 6 | 13 | 25 | Primary Blood Derived Cancer - Peripheral Blood |
| TCGA-AB-2820-03A-01T-0735-13 | 6 | 13 | 25 | Primary Blood Derived Cancer - Peripheral Blood |
| TCGA-AB-2821-03A-01T-0735-13 | 6 | 13 | 25 | Primary Blood Derived Cancer - Peripheral Blood |
| TCGA-AB-2822-03A-01T-0734-13 | 6 | 13 | 25 | Primary Blood Derived Cancer - Peripheral Blood |
| TCGA-AB-2823-03A-01T-0736-13 | 6 | 13 | 25 | Primary Blood Derived Cancer - Peripheral Blood |
| TCGA-AB-2824-03A-01T-0736-13 | 6 | 13 | 25 | Primary Blood Derived Cancer - Peripheral Blood |
| TCGA-AB-2825-03A-01T-0736-13 | 6 | 13 | 25 | Primary Blood Derived Cancer - Peripheral Blood |
| TCGA-AB-2826-03A-01T-0734-13 | 6 | 13 | 25 | Primary Blood Derived Cancer - Peripheral Blood |
| TCGA-AB-2828-03A-01T-0734-13 | 6 | 13 | 25 | Primary Blood Derived Cancer - Peripheral Blood |
| TCGA-AB-2830-03A-01T-0736-13 | 6 | 13 | 25 | Primary Blood Derived Cancer - Peripheral Blood |
| TCGA-AB-2832-03A-01T-0736-13 | 6 | 13 | 25 | Primary Blood Derived Cancer - Peripheral Blood |
| TCGA-AB-2833-03A-01T-0734-13 | 6 | 13 | 25 | Primary Blood Derived Cancer - Peripheral Blood |
| TCGA-AB-2834-03A-01T-0734-13 | 6 | 13 | 25 | Primary Blood Derived Cancer - Peripheral Blood |
| TCGA-AB-2835-03A-01T-0736-13 | 6 | 13 | 25 | Primary Blood Derived Cancer - Peripheral Blood |
| TCGA-AB-2836-03A-01T-0736-13 | 6 | 13 | 25 | Primary Blood Derived Cancer - Peripheral Blood |
| TCGA-AB-2837-03A-01T-0736-13 | 6 | 13 | 25 | Primary Blood Derived Cancer - Peripheral Blood |
| TCGA-AB-2838-03A-01T-0736-13 | 6 | 13 | 25 | Primary Blood Derived Cancer - Peripheral Blood |
| TCGA-AB-2839-03A-01T-0734-13 | 6 | 13 | 25 | Primary Blood Derived Cancer - Peripheral Blood |
| TCGA-AB-2840-03A-01T-0734-13 | 6 | 13 | 25 | Primary Blood Derived Cancer - Peripheral Blood |
| TCGA-AB-2841-03B-01T-0760-13 | 6 | 13 | 25 | Primary Blood Derived Cancer - Peripheral Blood |
| TCGA-AB-2842-03A-01T-0734-13 | 6 | 13 | 25 | Primary Blood Derived Cancer - Peripheral Blood |
| TCGA-AB-2843-03A-01T-0736-13 | 6 | 13 | 25 | Primary Blood Derived Cancer - Peripheral Blood |
| TCGA-AB-2844-03A-01T-0736-13 | 6 | 13 | 25 | Primary Blood Derived Cancer - Peripheral Blood |
| TCGA-AB-2845-03B-01T-0748-13 | 6 | 13 | 25 | Primary Blood Derived Cancer - Peripheral Blood |
| TCGA-AB-2846-03A-01T-0736-13 | 6 | 13 | 25 | Primary Blood Derived Cancer - Peripheral Blood |
| TCGA-AB-2847-03A-01T-0736-13 | 6 | 13 | 25 | Primary Blood Derived Cancer - Peripheral Blood |
| TCGA-AB-2848-03A-01T-0736-13 | 6 | 13 | 25 | Primary Blood Derived Cancer - Peripheral Blood |
| TCGA-AB-2849-03A-01T-0734-13 | 6 | 13 | 25 | Primary Blood Derived Cancer - Peripheral Blood |
| TCGA-AB-2851-03A-01T-0736-13 | 6 | 13 | 25 | Primary Blood Derived Cancer - Peripheral Blood |
| TCGA-AB-2853-03A-01T-0734-13 | 6 | 13 | 25 | Primary Blood Derived Cancer - Peripheral Blood |
| TCGA-AB-2854-03A-01T-0734-13 | 6 | 13 | 25 | Primary Blood Derived Cancer - Peripheral Blood |
| TCGA-AB-2855-03A-01T-0736-13 | 6 | 13 | 25 | Primary Blood Derived Cancer - Peripheral Blood |
| TCGA-AB-2856-03A-01T-0736-13 | 6 | 13 | 25 | Primary Blood Derived Cancer - Peripheral Blood |
| TCGA-AB-2857-03A-01T-0736-13 | 6 | 13 | 25 | Primary Blood Derived Cancer - Peripheral Blood |
| TCGA-AB-2858-03A-01T-0736-13 | 6 | 13 | 25 | Primary Blood Derived Cancer - Peripheral Blood |
| TCGA-AB-2859-03A-01T-0736-13 | 6 | 13 | 25 | Primary Blood Derived Cancer - Peripheral Blood |
| TCGA-AB-2860-03A-01T-0736-13 | 6 | 13 | 25 | Primary Blood Derived Cancer - Peripheral Blood |
| TCGA-AB-2861-03A-01T-0736-13 | 6 | 13 | 25 | Primary Blood Derived Cancer - Peripheral Blood |
| TCGA-AB-2862-03A-01T-0736-13 | 6 | 13 | 25 | Primary Blood Derived Cancer - Peripheral Blood |
| TCGA-AB-2863-03A-01T-0734-13 | 6 | 13 | 25 | Primary Blood Derived Cancer - Peripheral Blood |
| TCGA-AB-2865-03A-01T-0736-13 | 6 | 13 | 25 | Primary Blood Derived Cancer - Peripheral Blood |
| TCGA-AB-2866-03A-01T-0736-13 | 6 | 13 | 25 | Primary Blood Derived Cancer - Peripheral Blood |
| TCGA-AB-2867-03A-01T-0734-13 | 6 | 13 | 25 | Primary Blood Derived Cancer - Peripheral Blood |
| TCGA-AB-2868-03A-01T-0736-13 | 6 | 13 | 25 | Primary Blood Derived Cancer - Peripheral Blood |
| TCGA-AB-2869-03A-01T-0735-13 | 6 | 13 | 25 | Primary Blood Derived Cancer - Peripheral Blood |
| TCGA-AB-2870-03A-01T-0735-13 | 6 | 13 | 25 | Primary Blood Derived Cancer - Peripheral Blood |
| TCGA-AB-2871-03A-01T-0735-13 | 6 | 13 | 25 | Primary Blood Derived Cancer - Peripheral Blood |
| TCGA-AB-2872-03A-01T-0735-13 | 6 | 13 | 25 | Primary Blood Derived Cancer - Peripheral Blood |
| TCGA-AB-2873-03A-01T-0735-13 | 6 | 13 | 25 | Primary Blood Derived Cancer - Peripheral Blood |
| TCGA-AB-2874-03A-01T-0735-13 | 6 | 13 | 25 | Primary Blood Derived Cancer - Peripheral Blood |
| TCGA-AB-2875-03A-01T-0735-13 | 6 | 13 | 25 | Primary Blood Derived Cancer - Peripheral Blood |
| TCGA-AB-2877-03A-01T-0735-13 | 6 | 13 | 25 | Primary Blood Derived Cancer - Peripheral Blood |
| TCGA-AB-2879-03A-01T-0735-13 | 6 | 13 | 25 | Primary Blood Derived Cancer - Peripheral Blood |
| TCGA-AB-2880-03A-01T-0735-13 | 6 | 13 | 25 | Primary Blood Derived Cancer - Peripheral Blood |
| TCGA-AB-2881-03A-01T-0735-13 | 6 | 13 | 25 | Primary Blood Derived Cancer - Peripheral Blood |
| TCGA-AB-2882-03A-01T-0740-13 | 6 | 13 | 25 | Primary Blood Derived Cancer - Peripheral Blood |
| TCGA-AB-2884-03A-01T-0735-13 | 6 | 13 | 25 | Primary Blood Derived Cancer - Peripheral Blood |
| TCGA-AB-2885-03A-01T-0735-13 | 6 | 13 | 25 | Primary Blood Derived Cancer - Peripheral Blood |
| TCGA-AB-2886-03A-01T-0735-13 | 6 | 13 | 25 | Primary Blood Derived Cancer - Peripheral Blood |
| TCGA-AB-2887-03A-01T-0735-13 | 6 | 13 | 25 | Primary Blood Derived Cancer - Peripheral Blood |
| TCGA-AB-2888-03B-01T-0748-13 | 6 | 13 | 25 | Primary Blood Derived Cancer - Peripheral Blood |
| TCGA-AB-2889-03A-01T-0735-13 | 6 | 13 | 25 | Primary Blood Derived Cancer - Peripheral Blood |
| TCGA-AB-2890-03A-01T-0735-13 | 6 | 13 | 25 | Primary Blood Derived Cancer - Peripheral Blood |
| TCGA-AB-2891-03A-01T-0735-13 | 6 | 13 | 25 | Primary Blood Derived Cancer - Peripheral Blood |
| TCGA-AB-2895-03A-01T-0735-13 | 6 | 13 | 25 | Primary Blood Derived Cancer - Peripheral Blood |
| TCGA-AB-2896-03B-01T-0751-13 | 6 | 13 | 25 | Primary Blood Derived Cancer - Peripheral Blood |
| TCGA-AB-2897-03A-01T-0735-13 | 6 | 13 | 25 | Primary Blood Derived Cancer - Peripheral Blood |
| TCGA-AB-2898-03A-01T-0735-13 | 6 | 13 | 25 | Primary Blood Derived Cancer - Peripheral Blood |
| TCGA-AB-2899-03A-01T-0736-13 | 6 | 13 | 25 | Primary Blood Derived Cancer - Peripheral Blood |
| TCGA-AB-2900-03A-01T-0735-13 | 6 | 13 | 25 | Primary Blood Derived Cancer - Peripheral Blood |
| TCGA-AB-2901-03A-01T-0735-13 | 6 | 13 | 25 | Primary Blood Derived Cancer - Peripheral Blood |
| TCGA-AB-2903-03A-01T-0734-13 | 6 | 13 | 25 | Primary Blood Derived Cancer - Peripheral Blood |
| TCGA-AB-2904-03A-01T-0734-13 | 6 | 13 | 25 | Primary Blood Derived Cancer - Peripheral Blood |
| TCGA-AB-2908-03A-01T-0740-13 | 6 | 13 | 25 | Primary Blood Derived Cancer - Peripheral Blood |
| TCGA-AB-2909-03A-01T-0744-13 | 6 | 13 | 25 | Primary Blood Derived Cancer - Peripheral Blood |
| TCGA-AB-2910-03A-01T-0740-13 | 6 | 13 | 25 | Primary Blood Derived Cancer - Peripheral Blood |
| TCGA-AB-2911-03A-01T-0734-13 | 6 | 13 | 25 | Primary Blood Derived Cancer - Peripheral Blood |
| TCGA-AB-2912-03A-01T-0734-13 | 6 | 13 | 25 | Primary Blood Derived Cancer - Peripheral Blood |
| TCGA-AB-2913-03A-01T-0734-13 | 6 | 13 | 25 | Primary Blood Derived Cancer - Peripheral Blood |
| TCGA-AB-2914-03A-01T-0734-13 | 6 | 13 | 25 | Primary Blood Derived Cancer - Peripheral Blood |
| TCGA-AB-2915-03A-01T-0740-13 | 6 | 13 | 25 | Primary Blood Derived Cancer - Peripheral Blood |
| TCGA-AB-2916-03A-01T-0734-13 | 6 | 13 | 25 | Primary Blood Derived Cancer - Peripheral Blood |
| TCGA-AB-2917-03A-01T-0734-13 | 6 | 13 | 25 | Primary Blood Derived Cancer - Peripheral Blood |
| TCGA-AB-2918-03A-01T-0740-13 | 6 | 13 | 25 | Primary Blood Derived Cancer - Peripheral Blood |
| TCGA-AB-2919-03A-01T-0740-13 | 6 | 13 | 25 | Primary Blood Derived Cancer - Peripheral Blood |
| TCGA-AB-2920-03B-01T-0760-13 | 6 | 13 | 25 | Primary Blood Derived Cancer - Peripheral Blood |
| TCGA-AB-2921-03A-01T-0740-13 | 6 | 13 | 25 | Primary Blood Derived Cancer - Peripheral Blood |
| TCGA-AB-2924-03A-01T-0740-13 | 6 | 13 | 25 | Primary Blood Derived Cancer - Peripheral Blood |
| TCGA-AB-2925-03A-01T-0735-13 | 6 | 13 | 25 | Primary Blood Derived Cancer - Peripheral Blood |
| TCGA-AB-2927-03A-01T-0740-13 | 6 | 13 | 25 | Primary Blood Derived Cancer - Peripheral Blood |
| TCGA-AB-2928-03A-01T-0740-13 | 6 | 13 | 25 | Primary Blood Derived Cancer - Peripheral Blood |
| TCGA-AB-2929-03A-01T-0735-13 | 6 | 13 | 25 | Primary Blood Derived Cancer - Peripheral Blood |
| TCGA-AB-2930-03A-01T-0740-13 | 6 | 13 | 25 | Primary Blood Derived Cancer - Peripheral Blood |
| TCGA-AB-2931-03A-01T-0740-13 | 6 | 13 | 25 | Primary Blood Derived Cancer - Peripheral Blood |
| TCGA-AB-2932-03A-01T-0740-13 | 6 | 13 | 25 | Primary Blood Derived Cancer - Peripheral Blood |
| TCGA-AB-2933-03A-01T-0734-13 | 6 | 13 | 25 | Primary Blood Derived Cancer - Peripheral Blood |
| TCGA-AB-2934-03A-01T-0740-13 | 6 | 13 | 25 | Primary Blood Derived Cancer - Peripheral Blood |
| TCGA-AB-2935-03A-01T-0740-13 | 6 | 13 | 25 | Primary Blood Derived Cancer - Peripheral Blood |
| TCGA-AB-2936-03A-01T-0740-13 | 6 | 13 | 25 | Primary Blood Derived Cancer - Peripheral Blood |
| TCGA-AB-2937-03A-01T-0734-13 | 6 | 13 | 25 | Primary Blood Derived Cancer - Peripheral Blood |
| TCGA-AB-2938-03A-01T-0736-13 | 6 | 13 | 25 | Primary Blood Derived Cancer - Peripheral Blood |
| TCGA-AB-2939-03A-01T-0740-13 | 6 | 13 | 25 | Primary Blood Derived Cancer - Peripheral Blood |
| TCGA-AB-2940-03A-01T-0736-13 | 6 | 13 | 25 | Primary Blood Derived Cancer - Peripheral Blood |
| TCGA-AB-2941-03A-01T-0740-13 | 6 | 13 | 25 | Primary Blood Derived Cancer - Peripheral Blood |
| TCGA-AB-2942-03A-01T-0734-13 | 6 | 13 | 25 | Primary Blood Derived Cancer - Peripheral Blood |
| TCGA-AB-2943-03A-01T-0740-13 | 6 | 13 | 25 | Primary Blood Derived Cancer - Peripheral Blood |
| TCGA-AB-2944-03A-01T-0740-13 | 6 | 13 | 25 | Primary Blood Derived Cancer - Peripheral Blood |
| TCGA-AB-2946-03A-01T-0740-13 | 6 | 13 | 25 | Primary Blood Derived Cancer - Peripheral Blood |
| TCGA-AB-2948-03A-01T-0740-13 | 6 | 13 | 25 | Primary Blood Derived Cancer - Peripheral Blood |
| TCGA-AB-2949-03B-01T-0748-13 | 6 | 13 | 25 | Primary Blood Derived Cancer - Peripheral Blood |
| TCGA-AB-2950-03A-01T-0735-13 | 6 | 13 | 25 | Primary Blood Derived Cancer - Peripheral Blood |
| TCGA-AB-2952-03B-01T-0760-13 | 6 | 13 | 25 | Primary Blood Derived Cancer - Peripheral Blood |
| TCGA-AB-2954-03A-01T-0736-13 | 6 | 13 | 25 | Primary Blood Derived Cancer - Peripheral Blood |
| TCGA-AB-2955-03A-01T-0734-13 | 6 | 13 | 25 | Primary Blood Derived Cancer - Peripheral Blood |
| TCGA-AB-2956-03A-01T-0740-13 | 6 | 13 | 25 | Primary Blood Derived Cancer - Peripheral Blood |
| TCGA-AB-2959-03A-01T-0734-13 | 6 | 13 | 25 | Primary Blood Derived Cancer - Peripheral Blood |
| TCGA-AB-2963-03A-01T-0734-13 | 6 | 13 | 25 | Primary Blood Derived Cancer - Peripheral Blood |
| TCGA-AB-2964-03A-01T-0734-13 | 6 | 13 | 25 | Primary Blood Derived Cancer - Peripheral Blood |
| TCGA-AB-2965-03A-01T-0734-13 | 6 | 13 | 25 | Primary Blood Derived Cancer - Peripheral Blood |
| TCGA-AB-2966-03A-01T-0734-13 | 6 | 13 | 25 | Primary Blood Derived Cancer - Peripheral Blood |
| TCGA-AB-2967-03A-01T-0734-13 | 6 | 13 | 25 | Primary Blood Derived Cancer - Peripheral Blood |
| TCGA-AB-2969-03A-01T-0734-13 | 6 | 13 | 25 | Primary Blood Derived Cancer - Peripheral Blood |
| TCGA-AB-2970-03A-01T-0734-13 | 6 | 13 | 25 | Primary Blood Derived Cancer - Peripheral Blood |
| TCGA-AB-2971-03A-01T-0734-13 | 6 | 13 | 25 | Primary Blood Derived Cancer - Peripheral Blood |
| TCGA-AB-2972-03A-01T-0734-13 | 6 | 13 | 25 | Primary Blood Derived Cancer - Peripheral Blood |
| TCGA-AB-2973-03A-01T-0734-13 | 6 | 13 | 25 | Primary Blood Derived Cancer - Peripheral Blood |
| TCGA-AB-2975-03A-01T-0734-13 | 6 | 13 | 25 | Primary Blood Derived Cancer - Peripheral Blood |
| TCGA-AB-2976-03A-01T-0734-13 | 6 | 13 | 25 | Primary Blood Derived Cancer - Peripheral Blood |
| TCGA-AB-2977-03B-01T-0760-13 | 6 | 13 | 25 | Primary Blood Derived Cancer - Peripheral Blood |
| TCGA-AB-2978-03A-01T-0734-13 | 6 | 13 | 25 | Primary Blood Derived Cancer - Peripheral Blood |
| TCGA-AB-2979-03B-01T-0760-13 | 6 | 13 | 25 | Primary Blood Derived Cancer - Peripheral Blood |
| TCGA-AB-2980-03A-01T-0734-13 | 6 | 13 | 25 | Primary Blood Derived Cancer - Peripheral Blood |
| TCGA-AB-2981-03B-01T-0748-13 | 6 | 13 | 25 | Primary Blood Derived Cancer - Peripheral Blood |
| TCGA-AB-2982-03B-01T-0748-13 | 6 | 13 | 25 | Primary Blood Derived Cancer - Peripheral Blood |
| TCGA-AB-2983-03A-01T-0734-13 | 6 | 13 | 25 | Primary Blood Derived Cancer - Peripheral Blood |
| TCGA-AB-2984-03A-01T-0734-13 | 6 | 13 | 25 | Primary Blood Derived Cancer - Peripheral Blood |
| TCGA-AB-2985-03A-01T-0734-13 | 6 | 13 | 25 | Primary Blood Derived Cancer - Peripheral Blood |
| TCGA-AB-2986-03A-01T-0734-13 | 6 | 13 | 25 | Primary Blood Derived Cancer - Peripheral Blood |
| TCGA-AB-2987-03A-01T-0734-13 | 6 | 13 | 25 | Primary Blood Derived Cancer - Peripheral Blood |
| TCGA-AB-2988-03B-01T-0748-13 | 6 | 13 | 25 | Primary Blood Derived Cancer - Peripheral Blood |
| TCGA-AB-2990-03B-01T-0748-13 | 6 | 13 | 25 | Primary Blood Derived Cancer - Peripheral Blood |
| TCGA-AB-2991-03A-01T-0735-13 | 6 | 13 | 25 | Primary Blood Derived Cancer - Peripheral Blood |
| TCGA-AB-2992-03A-01T-0735-13 | 6 | 13 | 25 | Primary Blood Derived Cancer - Peripheral Blood |
| TCGA-AB-2993-03A-01T-0735-13 | 6 | 13 | 25 | Primary Blood Derived Cancer - Peripheral Blood |
| TCGA-AB-2994-03A-01T-0735-13 | 6 | 13 | 25 | Primary Blood Derived Cancer - Peripheral Blood |
| TCGA-AB-2995-03A-01T-0735-13 | 6 | 13 | 25 | Primary Blood Derived Cancer - Peripheral Blood |
| TCGA-AB-2996-03A-01T-0735-13 | 6 | 13 | 25 | Primary Blood Derived Cancer - Peripheral Blood |
| TCGA-AB-2998-03A-01T-0735-13 | 6 | 13 | 25 | Primary Blood Derived Cancer - Peripheral Blood |
| TCGA-AB-2999-03B-01T-0748-13 | 6 | 13 | 25 | Primary Blood Derived Cancer - Peripheral Blood |
| TCGA-AB-3000-03A-01T-0736-13 | 6 | 13 | 25 | Primary Blood Derived Cancer - Peripheral Blood |
| TCGA-AB-3001-03A-01T-0736-13 | 6 | 13 | 25 | Primary Blood Derived Cancer - Peripheral Blood |
| TCGA-AB-3002-03A-01T-0736-13 | 6 | 13 | 25 | Primary Blood Derived Cancer - Peripheral Blood |
| TCGA-AB-3005-03A-01T-0736-13 | 6 | 13 | 25 | Primary Blood Derived Cancer - Peripheral Blood |
| TCGA-AB-3006-03A-01T-0736-13 | 6 | 13 | 25 | Primary Blood Derived Cancer - Peripheral Blood |
| TCGA-AB-3007-03A-01T-0736-13 | 6 | 13 | 25 | Primary Blood Derived Cancer - Peripheral Blood |
| TCGA-AB-3008-03A-01T-0736-13 | 6 | 13 | 25 | Primary Blood Derived Cancer - Peripheral Blood |
| TCGA-AB-3009-03A-01T-0736-13 | 6 | 13 | 25 | Primary Blood Derived Cancer - Peripheral Blood |
| TCGA-AB-3011-03A-01T-0736-13 | 6 | 13 | 25 | Primary Blood Derived Cancer - Peripheral Blood |
| TCGA-AB-3012-03A-01T-0736-13 | 6 | 13 | 25 | Primary Blood Derived Cancer - Peripheral Blood |
| TCGA-AC-A23C-01A-12R-A169-07 | 9 | 7 | 142 | Primary solid Tumor |
| TCGA-AC-A23E-01A-11R-A157-07 | 9 | 7 | 136 | Primary solid Tumor |
| TCGA-AC-A23G-01A-11R-A213-07 | 9 | 7 | 202 | Primary solid Tumor |
| TCGA-AC-A23H-01A-11R-A157-07 | 9 | 7 | 136 | Primary solid Tumor |
| TCGA-AC-A2B8-01A-11R-A17B-07 | 9 | 7 | 155 | Primary solid Tumor |
| TCGA-AC-A2BK-01A-11R-A21T-07 | 10 | 8 | 216 | Primary solid Tumor |
| TCGA-AC-A2BM-01A-11R-A21T-07 | 9 | 7 | 216 | Primary solid Tumor |
| TCGA-AC-A2FB-01A-11R-A17B-07 | 9 | 7 | 155 | Primary solid Tumor |
| TCGA-AC-A2FE-01A-11R-A19W-07 | 9 | NA | 185 | Primary solid Tumor |
| TCGA-AC-A2FF-01A-11R-A17B-07 | 9 | 7 | 155 | Primary solid Tumor |
| TCGA-AC-A2FG-01A-11R-A17B-07 | 9 | 7 | 155 | Primary solid Tumor |
| TCGA-AC-A2FK-01A-12R-A180-07 | 9 | 7 | 167 | Primary solid Tumor |
| TCGA-AC-A2FM-01A-11R-A19W-07 | 9 | NA | 185 | Primary solid Tumor |
| TCGA-AC-A2FO-01A-11R-A180-07 | 9 | 7 | 167 | Primary solid Tumor |
| TCGA-AC-A2QH-01A-11R-A18M-07 | 3 | 6 | 177 | Primary solid Tumor |
| TCGA-AC-A2QI-01A-12R-A19W-07 | 9 | NA | 185 | Primary solid Tumor |
| TCGA-AC-A2QJ-01A-12R-A19W-07 | 3 | NA | 185 | Primary solid Tumor |
| TCGA-AC-A3BB-01A-21R-A19W-07 | 9 | NA | 185 | Primary solid Tumor |
| TCGA-AC-A3EH-01A-22R-A22K-07 | 9 | NA | 227 | Primary solid Tumor |
| TCGA-AC-A3HN-01A-11R-A213-07 | 9 | 7 | 202 | Primary solid Tumor |
| TCGA-AC-A3OD-01A-11R-A21T-07 | 9 | 7 | 216 | Primary solid Tumor |
| TCGA-AC-A3QP-01A-11R-A22U-07 | 9 | NA | 234 | Primary solid Tumor |
| TCGA-AC-A3QQ-01A-11R-A22K-07 | 9 | NA | 227 | Primary solid Tumor |
| TCGA-AC-A3TM-01A-11R-A22K-07 | 9 | NA | 227 | Primary solid Tumor |
| TCGA-AC-A3TN-01A-11R-A22K-07 | 9 | NA | 227 | Primary solid Tumor |
| TCGA-AC-A3W5-01A-11R-A22K-07 | 9 | NA | 227 | Primary solid Tumor |
| TCGA-AC-A3W6-01A-12R-A22K-07 | 9 | NA | 227 | Primary solid Tumor |
| TCGA-AC-A3W7-01A-11R-A22K-07 | 9 | NA | 227 | Primary solid Tumor |
| TCGA-AC-A3YI-01A-21R-A239-07 | 9 | NA | 239 | Primary solid Tumor |
| TCGA-AC-A3YJ-01A-11R-A22U-07 | 9 | NA | 234 | Primary solid Tumor |
| TCGA-AC-A4ZE-01A-11R-A41B-07 | 9 | NA | 379 | Primary solid Tumor |
| TCGA-AC-A5EH-01A-11R-A28M-07 | 9 | NA | 296 | Primary solid Tumor |
| TCGA-AC-A5XS-01A-11R-A29R-07 | 9 | NA | 305 | Primary solid Tumor |
| TCGA-AC-A5XU-01A-11R-A28M-07 | 9 | NA | 296 | Primary solid Tumor |
| TCGA-AC-A62V-01A-11R-A31O-07 | 9 | NA | 322 | Primary solid Tumor |
| TCGA-AC-A62X-01A-11R-A29R-07 | 10 | NA | 305 | Primary solid Tumor |
| TCGA-AC-A62Y-01A-11R-A29R-07 | 9 | NA | 305 | Primary solid Tumor |
| TCGA-AC-A6IV-01A-12R-A33J-07 | 9 | NA | 338 | Primary solid Tumor |
| TCGA-AC-A6IW-01A-12R-A33J-07 | 10 | NA | 338 | Primary solid Tumor |
| TCGA-AC-A6IX-01A-12R-A32P-07 | 9 | NA | 334 | Primary solid Tumor |
| TCGA-AC-A6NO-01A-12R-A33J-07 | 9 | NA | 338 | Primary solid Tumor |
| TCGA-AC-A7VB-01A-11R-A352-07 | 9 | NA | 360 | Primary solid Tumor |
| TCGA-AC-A7VC-01A-11R-A352-07 | 3 | NA | 360 | Primary solid Tumor |
| TCGA-AC-A8OP-01A-11R-A36F-07 | 9 | NA | 372 | Primary solid Tumor |
| TCGA-AC-A8OQ-01A-11R-A41B-07 | 10 | NA | 379 | Primary solid Tumor |
| TCGA-AC-A8OR-01A-21R-A41B-07 | 9 | NA | 379 | Primary solid Tumor |
| TCGA-AC-A8OS-01A-12R-A41B-07 | 9 | NA | 379 | Primary solid Tumor |
| TCGA-AD-5900-01A-11R-1653-07 | 7 | NA | 89 | Primary solid Tumor |
| TCGA-AD-6548-01A-11R-1839-07 | 7 | NA | 138 | Primary solid Tumor |
| TCGA-AD-6888-01A-11R-1928-07 | 7 | NA | 157 | Primary solid Tumor |
| TCGA-AD-6889-01A-11R-1928-07 | 7 | NA | 157 | Primary solid Tumor |
| TCGA-AD-6890-01A-11R-1928-07 | 7 | NA | 157 | Primary solid Tumor |
| TCGA-AD-6895-01A-11R-1928-07 | 7 | NA | 157 | Primary solid Tumor |
| TCGA-AD-6899-01A-11R-1928-07 | 7 | NA | 157 | Primary solid Tumor |
| TCGA-AD-6901-01A-11R-1928-07 | 7 | NA | 157 | Primary solid Tumor |
| TCGA-AD-6963-01A-11R-1928-07 | 7 | NA | 157 | Primary solid Tumor |
| TCGA-AD-6964-01A-11R-1928-07 | 7 | NA | 157 | Primary solid Tumor |
| TCGA-AD-6965-01A-11R-1928-07 | 7 | NA | 157 | Primary solid Tumor |
| TCGA-AD-A5EJ-01A-11R-A28H-07 | 7 | NA | 300 | Primary solid Tumor |
| TCGA-AD-A5EK-01A-11R-A28H-07 | 7 | NA | 300 | Primary solid Tumor |
| TCGA-AF-2687-01A-02R-1736-07 | 7 | NA | 122 | Primary solid Tumor |
| TCGA-AF-2690-01A-02R-1736-07 | 7 | NA | 122 | Primary solid Tumor |
| TCGA-AF-2691-01A-01R-0821-07 | 7 | 15 | 42 | Primary solid Tumor |
| TCGA-AF-2692-01A-01R-0821-07 | 7 | 15 | 42 | Primary solid Tumor |
| TCGA-AF-2693-01A-02R-1736-07 | 7 | NA | 122 | Primary solid Tumor |
| TCGA-AF-3400-01A-01R-0821-07 | 7 | 15 | 42 | Primary solid Tumor |
| TCGA-AF-3911-01A-01R-1736-07 | 7 | NA | 122 | Primary solid Tumor |
| TCGA-AF-3913-01A-02R-1119-07 | 7 | 15 | 46 | Primary solid Tumor |
| TCGA-AF-4110-01A-02R-1736-07 | 7 | NA | 122 | Primary solid Tumor |
| TCGA-AF-5654-01A-01R-1660-07 | 7 | NA | 102 | Primary solid Tumor |
| TCGA-AF-6136-01A-11R-1830-07 | 7 | NA | 139 | Primary solid Tumor |
| TCGA-AF-6655-01A-11R-1830-07 | 7 | NA | 139 | Primary solid Tumor |
| TCGA-AF-6672-01A-11R-1830-07 | 7 | NA | 139 | Primary solid Tumor |
| TCGA-AF-A56K-01A-32R-A39D-07 | 7 | NA | 411 | Primary solid Tumor |
| TCGA-AF-A56L-01A-31R-A39D-07 | 7 | NA | 411 | Primary solid Tumor |
| TCGA-AF-A56N-01A-12R-A39D-07 | 7 | NA | 411 | Primary solid Tumor |
| TCGA-AG-3574-01A-01R-0821-07 | 7 | 15 | 42 | Primary solid Tumor |
| TCGA-AG-3575-01A-01R-0821-07 | 7 | 15 | 42 | Primary solid Tumor |
| TCGA-AG-3578-01A-01R-0821-07 | 7 | 15 | 42 | Primary solid Tumor |
| TCGA-AG-3580-01A-01R-0821-07 | 7 | 15 | 42 | Primary solid Tumor |
| TCGA-AG-3581-01A-01R-0821-07 | 7 | 15 | 42 | Primary solid Tumor |
| TCGA-AG-3582-01A-01R-0821-07 | 7 | 15 | 42 | Primary solid Tumor |
| TCGA-AG-3583-01A-01R-0821-07 | 7 | 15 | 42 | Primary solid Tumor |
| TCGA-AG-3584-01A-01R-0821-07 | 7 | 15 | 42 | Primary solid Tumor |
| TCGA-AG-3586-01A-02R-0821-07 | 7 | 15 | 42 | Primary solid Tumor |
| TCGA-AG-3587-01A-01R-0821-07 | 7 | 15 | 42 | Primary solid Tumor |
| TCGA-AG-3591-01A-01R-1736-07 | 7 | NA | 122 | Primary solid Tumor |
| TCGA-AG-3592-01A-02R-1736-07 | 7 | NA | 122 | Primary solid Tumor |
| TCGA-AG-3593-01A-01R-0821-07 | 7 | 15 | 42 | Primary solid Tumor |
| TCGA-AG-3594-01A-02R-0821-07 | 7 | 15 | 42 | Primary solid Tumor |
| TCGA-AG-3598-01A-01R-0826-07 | 7 | 15 | 42 | Primary solid Tumor |
| TCGA-AG-3599-01A-02R-0826-07 | 7 | 15 | 42 | Primary solid Tumor |
| TCGA-AG-3600-01A-01R-0826-07 | 7 | 15 | 42 | Primary solid Tumor |
| TCGA-AG-3601-01A-01R-0826-07 | 7 | 15 | 42 | Primary solid Tumor |
| TCGA-AG-3602-01A-02R-0826-07 | 7 | 15 | 42 | Primary solid Tumor |
| TCGA-AG-3605-01A-01R-0826-07 | 7 | 15 | 42 | Primary solid Tumor |
| TCGA-AG-3608-01A-01R-0826-07 | 7 | 15 | 42 | Primary solid Tumor |
| TCGA-AG-3609-01A-02R-0826-07 | 7 | 15 | 42 | Primary solid Tumor |
| TCGA-AG-3611-01A-01R-0826-07 | 7 | 15 | 42 | Primary solid Tumor |
| TCGA-AG-3612-01A-01R-0826-07 | 7 | 15 | 42 | Primary solid Tumor |
| TCGA-AG-3725-01A-11R-1736-07 | 7 | NA | 122 | Primary solid Tumor |
| TCGA-AG-3726-01A-02R-0905-07 | 7 | 15 | 42 | Primary solid Tumor |
| TCGA-AG-3727-01A-01R-0905-07 | 7 | 15 | 42 | Primary solid Tumor |
| TCGA-AG-3728-01A-01R-0905-07 | 7 | 15 | 42 | Primary solid Tumor |
| TCGA-AG-3731-01A-11R-1736-07 | 7 | NA | 122 | Primary solid Tumor |
| TCGA-AG-3732-01A-11R-1660-07 | 7 | NA | 102 | Primary solid Tumor |
| TCGA-AG-3742-01A-11R-1660-07 | 7 | NA | 102 | Primary solid Tumor |
| TCGA-AG-3878-01A-02R-0905-07 | 7 | 15 | 42 | Primary solid Tumor |
| TCGA-AG-3881-01A-01R-0905-07 | 7 | 15 | 42 | Primary solid Tumor |
| TCGA-AG-3882-01A-01R-0905-07 | 7 | 15 | 42 | Primary solid Tumor |
| TCGA-AG-3883-01A-02R-0905-07 | 7 | 15 | 42 | Primary solid Tumor |
| TCGA-AG-3885-01A-01R-0905-07 | 7 | 15 | 42 | Primary solid Tumor |
| TCGA-AG-3887-01A-01R-1119-07 | 7 | 15 | 46 | Primary solid Tumor |
| TCGA-AG-3890-01A-01R-1119-07 | 7 | 15 | 46 | Primary solid Tumor |
| TCGA-AG-3892-01A-01R-1119-07 | 7 | 15 | 46 | Primary solid Tumor |
| TCGA-AG-3893-01A-01R-1119-07 | 7 | 15 | 46 | Primary solid Tumor |
| TCGA-AG-3894-01A-01R-1119-07 | 7 | 15 | 46 | Primary solid Tumor |
| TCGA-AG-3896-01A-01R-1119-07 | 7 | 15 | 46 | Primary solid Tumor |
| TCGA-AG-3898-01A-01R-1119-07 | 7 | 15 | 46 | Primary solid Tumor |
| TCGA-AG-3901-01A-01R-1119-07 | 7 | 15 | 46 | Primary solid Tumor |
| TCGA-AG-3902-01A-01R-A32Z-07 | 7 | NA | 46 | Primary solid Tumor |
| TCGA-AG-3909-01A-01R-1119-07 | 7 | 15 | 46 | Primary solid Tumor |
| TCGA-AG-3999-01A-01R-1119-07 | 7 | 15 | 46 | Primary solid Tumor |
| TCGA-AG-4001-01A-02R-1119-07 | 7 | 15 | 46 | Primary solid Tumor |
| TCGA-AG-4005-01A-01R-1119-07 | 7 | 15 | 46 | Primary solid Tumor |
| TCGA-AG-4007-01A-01R-1119-07 | 7 | 15 | 46 | Primary solid Tumor |
| TCGA-AG-4008-01A-01R-1119-07 | 7 | 15 | 46 | Primary solid Tumor |
| TCGA-AG-4015-01A-01R-1119-07 | 7 | 15 | 46 | Primary solid Tumor |
| TCGA-AG-4021-01A-01R-1736-07 | 7 | NA | 122 | Primary solid Tumor |
| TCGA-AG-4022-01A-01R-1736-07 | 7 | NA | 122 | Primary solid Tumor |
| TCGA-AG-A002-01A-01R-A002-07 | 7 | 15 | 43 | Primary solid Tumor |
| TCGA-AG-A008-01A-01R-A002-07 | 7 | 15 | 43 | Primary solid Tumor |
| TCGA-AG-A00C-01A-01R-A002-07 | 7 | 15 | 43 | Primary solid Tumor |
| TCGA-AG-A00H-01A-01R-A00A-07 | 7 | 15 | 43 | Primary solid Tumor |
| TCGA-AG-A00Y-01A-02R-A002-07 | 7 | 15 | 43 | Primary solid Tumor |
| TCGA-AG-A011-01A-01R-A002-07 | 7 | 15 | 43 | Primary solid Tumor |
| TCGA-AG-A014-01A-02R-A002-07 | 7 | 15 | 43 | Primary solid Tumor |
| TCGA-AG-A015-01A-01R-A002-07 | 7 | 15 | 43 | Primary solid Tumor |
| TCGA-AG-A016-01A-01R-A002-07 | 7 | 15 | 43 | Primary solid Tumor |
| TCGA-AG-A01J-01A-01R-A00A-07 | 7 | 15 | 43 | Primary solid Tumor |
| TCGA-AG-A01L-01A-01R-A002-07 | 7 | 15 | 43 | Primary solid Tumor |
| TCGA-AG-A01N-01A-01R-A00A-07 | 7 | 15 | 43 | Primary solid Tumor |
| TCGA-AG-A01W-01A-21R-A083-07 | 7 | 15 | 67 | Primary solid Tumor |
| TCGA-AG-A01Y-01A-41R-A083-07 | 7 | 15 | 67 | Primary solid Tumor |
| TCGA-AG-A020-01A-21R-A083-07 | 7 | 15 | 67 | Primary solid Tumor |
| TCGA-AG-A023-01A-01R-A00A-07 | 7 | 15 | 43 | Primary solid Tumor |
| TCGA-AG-A025-01A-01R-A00A-07 | 7 | 15 | 43 | Primary solid Tumor |
| TCGA-AG-A026-01A-01R-A00A-07 | 7 | 15 | 67 | Primary solid Tumor |
| TCGA-AG-A02G-01A-01R-A00A-07 | 7 | 15 | 43 | Primary solid Tumor |
| TCGA-AG-A02N-01A-11R-A083-07 | 7 | 15 | 67 | Primary solid Tumor |
| TCGA-AG-A02X-01A-01R-A00A-07 | 7 | 15 | 43 | Primary solid Tumor |
| TCGA-AG-A032-01A-01R-A00A-07 | 7 | 15 | 43 | Primary solid Tumor |
| TCGA-AG-A036-01A-12R-A083-07 | 7 | 15 | 67 | Primary solid Tumor |
| TCGA-AH-6544-01A-11R-1830-07 | 7 | NA | 139 | Primary solid Tumor |
| TCGA-AH-6547-01A-11R-1830-07 | 7 | NA | 139 | Primary solid Tumor |
| TCGA-AH-6549-01A-11R-1830-07 | 7 | NA | 139 | Primary solid Tumor |
| TCGA-AH-6643-01A-11R-1830-07 | 7 | NA | 139 | Primary solid Tumor |
| TCGA-AH-6644-01A-21R-1830-07 | 7 | NA | 139 | Primary solid Tumor |
| TCGA-AH-6897-01A-11R-1928-07 | 7 | NA | 158 | Primary solid Tumor |
| TCGA-AH-6903-01A-11R-1928-07 | 7 | NA | 158 | Primary solid Tumor |
| TCGA-AJ-A23M-01A-11R-A157-07 | 16 | 11 | 137 | Primary solid Tumor |
| TCGA-AJ-A23N-01A-11R-A22K-07 | 16 | NA | 228 | Primary solid Tumor |
| TCGA-AJ-A23O-01A-11R-A157-07 | 16 | 11 | 137 | Primary solid Tumor |
| TCGA-AJ-A2QK-01A-11R-A18M-07 | 16 | 11 | 178 | Primary solid Tumor |
| TCGA-AJ-A2QL-01A-11R-A18M-07 | 16 | 11 | 178 | Primary solid Tumor |
| TCGA-AJ-A2QM-01A-11R-A18M-07 | 16 | NA | 178 | Primary solid Tumor |
| TCGA-AJ-A2QN-01A-11R-A18M-07 | 16 | 11 | 178 | Primary solid Tumor |
| TCGA-AJ-A2QO-01A-11R-A32Y-07 | 16 | NA | 178 | Primary solid Tumor |
| TCGA-AJ-A3BD-01A-11R-A19W-07 | 16 | NA | 186 | Primary solid Tumor |
| TCGA-AJ-A3BF-01A-11R-A213-07 | 16 | NA | 201 | Primary solid Tumor |
| TCGA-AJ-A3BG-01A-11R-A19W-07 | 16 | NA | 186 | Primary solid Tumor |
| TCGA-AJ-A3BH-01A-11R-A19W-07 | 16 | NA | 186 | Primary solid Tumor |
| TCGA-AJ-A3BI-01A-11R-A213-07 | 16 | NA | 201 | Primary solid Tumor |
| TCGA-AJ-A3BK-01A-11R-A19W-07 | 16 | NA | 186 | Primary solid Tumor |
| TCGA-AJ-A3EJ-01A-11R-A19W-07 | 16 | NA | 186 | Primary solid Tumor |
| TCGA-AJ-A3EK-01A-11R-A19W-07 | 16 | NA | 186 | Primary solid Tumor |
| TCGA-AJ-A3EL-01A-11R-A213-07 | 16 | NA | 201 | Primary solid Tumor |
| TCGA-AJ-A3EM-01A-11R-A213-07 | 16 | NA | 201 | Primary solid Tumor |
| TCGA-AJ-A3I9-01A-11R-A22K-07 | 16 | NA | 228 | Primary solid Tumor |
| TCGA-AJ-A3IA-01A-11R-A213-07 | 16 | NA | 201 | Primary solid Tumor |
| TCGA-AJ-A3NC-01A-11R-A22K-07 | 16 | NA | 228 | Primary solid Tumor |
| TCGA-AJ-A3NE-01A-11R-A22K-07 | 16 | NA | 228 | Primary solid Tumor |
| TCGA-AJ-A3NF-01A-11R-A22K-07 | 16 | NA | 228 | Primary solid Tumor |
| TCGA-AJ-A3NG-01A-11R-A22K-07 | 16 | NA | 228 | Primary solid Tumor |
| TCGA-AJ-A3NH-01A-11R-A22K-07 | 16 | NA | 228 | Primary solid Tumor |
| TCGA-AJ-A3OJ-01A-11R-A22K-07 | 16 | NA | 228 | Primary solid Tumor |
| TCGA-AJ-A3OK-01A-12R-A22K-07 | 16 | NA | 228 | Primary solid Tumor |
| TCGA-AJ-A3OL-01A-11R-A22K-07 | 16 | NA | 228 | Primary solid Tumor |
| TCGA-AJ-A3QS-01A-11R-A22K-07 | 16 | NA | 228 | Primary solid Tumor |
| TCGA-AJ-A3TW-01A-11R-A22K-07 | 20 | NA | 228 | Primary solid Tumor |
| TCGA-AJ-A5DV-01A-11R-A27V-07 | 16 | NA | 289 | Primary solid Tumor |
| TCGA-AJ-A5DW-01A-11R-A27V-07 | 16 | NA | 289 | Primary solid Tumor |
| TCGA-AJ-A6NU-01A-11R-A34R-07 | 16 | NA | 354 | Primary solid Tumor |
| TCGA-AJ-A8CT-01A-11R-A37O-07 | 16 | NA | 381 | Primary solid Tumor |
| TCGA-AJ-A8CV-01A-11R-A37O-07 | 16 | NA | 381 | Primary solid Tumor |
| TCGA-AJ-A8CW-01A-11R-A37O-07 | 16 | NA | 381 | Primary solid Tumor |
| TCGA-AK-3425-01A-02R-1277-07 | 14 | NA | 63 | Primary solid Tumor |
| TCGA-AK-3426-01A-02R-1325-07 | 14 | 9 | 50 | Primary solid Tumor |
| TCGA-AK-3427-01A-01R-0864-07 | 14 | 9 | 32 | Primary solid Tumor |
| TCGA-AK-3428-01A-02R-1277-07 | 14 | 9 | 63 | Primary solid Tumor |
| TCGA-AK-3429-01A-02R-1325-07 | 14 | 9 | 50 | Primary solid Tumor |
| TCGA-AK-3431-01A-02R-1277-07 | 14 | 9 | 63 | Primary solid Tumor |
| TCGA-AK-3433-01A-02R-1277-07 | 14 | 9 | 63 | Primary solid Tumor |
| TCGA-AK-3434-01A-02R-1277-07 | 14 | 9 | 63 | Primary solid Tumor |
| TCGA-AK-3436-01A-02R-1325-07 | 14 | 9 | 50 | Primary solid Tumor |
| TCGA-AK-3440-01A-02R-1277-07 | 14 | 9 | 32 | Primary solid Tumor |
| TCGA-AK-3443-01A-02R-1325-07 | 14 | 9 | 32 | Primary solid Tumor |
| TCGA-AK-3444-01A-02R-1325-07 | 14 | 9 | 32 | Primary solid Tumor |
| TCGA-AK-3445-01A-02R-1277-07 | 14 | 9 | 63 | Primary solid Tumor |
| TCGA-AK-3447-01A-01R-1766-07 | 14 | NA | 32 | Primary solid Tumor |
| TCGA-AK-3450-01A-02R-1277-07 | 14 | 9 | 63 | Primary solid Tumor |
| TCGA-AK-3451-01A-02R-1188-07 | 14 | 9 | 50 | Primary solid Tumor |
| TCGA-AK-3453-01A-02R-1277-07 | 14 | 9 | 32 | Primary solid Tumor |
| TCGA-AK-3454-01A-02R-1277-07 | 14 | 9 | 63 | Primary solid Tumor |
| TCGA-AK-3455-01A-01R-0864-07 | 14 | 9 | 32 | Primary solid Tumor |
| TCGA-AK-3456-01A-02R-1325-07 | 14 | 9 | 50 | Primary solid Tumor |
| TCGA-AK-3458-01A-01R-1503-07 | 14 | 9 | 82 | Primary solid Tumor |
| TCGA-AK-3460-01A-02R-1277-07 | 14 | 9 | 63 | Primary solid Tumor |
| TCGA-AK-3461-01A-02R-1277-07 | 14 | 9 | 63 | Primary solid Tumor |
| TCGA-AK-3465-01A-02R-1325-07 | 14 | 9 | 32 | Primary solid Tumor |
| TCGA-AM-5820-01A-01R-1653-07 | 7 | NA | 89 | Primary solid Tumor |
| TCGA-AM-5821-01A-01R-1653-07 | 7 | NA | 89 | Primary solid Tumor |
| TCGA-AN-A03X-01A-21R-A00Z-07 | 9 | 7 | 47 | Primary solid Tumor |
| TCGA-AN-A03Y-01A-21R-A00Z-07 | 9 | 7 | 47 | Primary solid Tumor |
| TCGA-AN-A041-01A-11R-A034-07 | 9 | 7 | 56 | Primary solid Tumor |
| TCGA-AN-A046-01A-21R-A034-07 | 9 | 7 | 56 | Primary solid Tumor |
| TCGA-AN-A049-01A-21R-A00Z-07 | 9 | 7 | 47 | Primary solid Tumor |
| TCGA-AN-A04A-01A-21R-A034-07 | 9 | 7 | 56 | Primary solid Tumor |
| TCGA-AN-A04C-01A-21R-A034-07 | 9 | 7 | 56 | Primary solid Tumor |
| TCGA-AN-A04D-01A-21R-A034-07 | 10 | 8 | 56 | Primary solid Tumor |
| TCGA-AN-A0AJ-01A-11R-A00Z-07 | 9 | 7 | 47 | Primary solid Tumor |
| TCGA-AN-A0AK-01A-21R-A00Z-07 | 9 | 7 | 47 | Primary solid Tumor |
| TCGA-AN-A0AL-01A-11R-A00Z-07 | 10 | 8 | 47 | Primary solid Tumor |
| TCGA-AN-A0AM-01A-11R-A034-07 | 9 | 7 | 56 | Primary solid Tumor |
| TCGA-AN-A0AR-01A-11R-A00Z-07 | 10 | NA | 47 | Primary solid Tumor |
| TCGA-AN-A0AS-01A-11R-A00Z-07 | 9 | 7 | 47 | Primary solid Tumor |
| TCGA-AN-A0AT-01A-11R-A034-07 | 10 | 8 | 56 | Primary solid Tumor |
| TCGA-AN-A0FD-01A-11R-A034-07 | 9 | 7 | 56 | Primary solid Tumor |
| TCGA-AN-A0FF-01A-11R-A034-07 | 9 | 7 | 56 | Primary solid Tumor |
| TCGA-AN-A0FJ-01A-11R-A00Z-07 | 10 | 8 | 47 | Primary solid Tumor |
| TCGA-AN-A0FK-01A-11R-A034-07 | 9 | 7 | 56 | Primary solid Tumor |
| TCGA-AN-A0FL-01A-11R-A034-07 | 10 | 8 | 56 | Primary solid Tumor |
| TCGA-AN-A0FN-01A-11R-A034-07 | 9 | 7 | 56 | Primary solid Tumor |
| TCGA-AN-A0FS-01A-11R-A034-07 | 9 | 7 | 56 | Primary solid Tumor |
| TCGA-AN-A0FT-01A-11R-A034-07 | 9 | 7 | 56 | Primary solid Tumor |
| TCGA-AN-A0FV-01A-11R-A00Z-07 | 9 | 7 | 47 | Primary solid Tumor |
| TCGA-AN-A0FW-01A-11R-A034-07 | 9 | 7 | 56 | Primary solid Tumor |
| TCGA-AN-A0FX-01A-11R-A034-07 | 10 | 8 | 56 | Primary solid Tumor |
| TCGA-AN-A0FY-01A-11R-A034-07 | 9 | 7 | 56 | Primary solid Tumor |
| TCGA-AN-A0FZ-01A-11R-A034-07 | 9 | 7 | 56 | Primary solid Tumor |
| TCGA-AN-A0G0-01A-11R-A034-07 | 10 | 8 | 56 | Primary solid Tumor |
| TCGA-AN-A0XL-01A-11R-A10J-07 | 9 | 7 | 80 | Primary solid Tumor |
| TCGA-AN-A0XN-01A-21R-A109-07 | 9 | 7 | 74 | Primary solid Tumor |
| TCGA-AN-A0XO-01A-11R-A109-07 | 9 | 7 | 74 | Primary solid Tumor |
| TCGA-AN-A0XP-01A-11R-A109-07 | 9 | 7 | 74 | Primary solid Tumor |
| TCGA-AN-A0XR-01A-11R-A109-07 | 9 | 7 | 74 | Primary solid Tumor |
| TCGA-AN-A0XS-01A-22R-A109-07 | 9 | 7 | 74 | Primary solid Tumor |
| TCGA-AN-A0XT-01A-11R-A109-07 | 9 | 7 | 74 | Primary solid Tumor |
| TCGA-AN-A0XU-01A-11R-A109-07 | 10 | 8 | 74 | Primary solid Tumor |
| TCGA-AN-A0XV-01A-11R-A109-07 | 9 | 7 | 74 | Primary solid Tumor |
| TCGA-AN-A0XW-01A-11R-A109-07 | 9 | 7 | 74 | Primary solid Tumor |
| TCGA-AO-A03L-01A-41R-A056-07 | 9 | 7 | 61 | Primary solid Tumor |
| TCGA-AO-A03M-01B-11R-A10J-07 | 9 | 7 | 80 | Primary solid Tumor |
| TCGA-AO-A03N-01B-11R-A10J-07 | 9 | 7 | 80 | Primary solid Tumor |
| TCGA-AO-A03O-01A-11R-A00Z-07 | 9 | 7 | 47 | Primary solid Tumor |
| TCGA-AO-A03P-01A-11R-A00Z-07 | 9 | 7 | 47 | Primary solid Tumor |
| TCGA-AO-A03R-01A-21R-A034-07 | 9 | 7 | 56 | Primary solid Tumor |
| TCGA-AO-A03T-01A-21R-A034-07 | 9 | 7 | 56 | Primary solid Tumor |
| TCGA-AO-A03U-01B-21R-A10J-07 | 10 | 8 | 80 | Primary solid Tumor |
| TCGA-AO-A03V-01A-11R-A115-07 | 9 | 7 | 85 | Primary solid Tumor |
| TCGA-AO-A0J2-01A-11R-A034-07 | 9 | 7 | 56 | Primary solid Tumor |
| TCGA-AO-A0J3-01A-11R-A034-07 | 9 | 7 | 56 | Primary solid Tumor |
| TCGA-AO-A0J4-01A-11R-A034-07 | 10 | 8 | 56 | Primary solid Tumor |
| TCGA-AO-A0J5-01A-11R-A034-07 | 9 | 7 | 56 | Primary solid Tumor |
| TCGA-AO-A0J6-01A-11R-A034-07 | 10 | 8 | 56 | Primary solid Tumor |
| TCGA-AO-A0J7-01A-11R-A034-07 | 9 | 7 | 56 | Primary solid Tumor |
| TCGA-AO-A0J8-01A-21R-A034-07 | 9 | 7 | 56 | Primary solid Tumor |
| TCGA-AO-A0J9-01A-11R-A034-07 | 9 | 7 | 56 | Primary solid Tumor |
| TCGA-AO-A0JA-01A-11R-A056-07 | 9 | 7 | 61 | Primary solid Tumor |
| TCGA-AO-A0JB-01A-11R-A32Y-07 | 9 | NA | 61 | Primary solid Tumor |
| TCGA-AO-A0JC-01A-11R-A056-07 | 6 | 7 | 61 | Primary solid Tumor |
| TCGA-AO-A0JD-01A-11R-A056-07 | 9 | 7 | 61 | Primary solid Tumor |
| TCGA-AO-A0JE-01A-11R-A056-07 | 9 | 7 | 61 | Primary solid Tumor |
| TCGA-AO-A0JF-01A-11R-A056-07 | 9 | 7 | 61 | Primary solid Tumor |
| TCGA-AO-A0JG-01A-31R-A084-07 | 9 | 7 | 72 | Primary solid Tumor |
| TCGA-AO-A0JI-01A-21R-A056-07 | 9 | 7 | 61 | Primary solid Tumor |
| TCGA-AO-A0JJ-01A-11R-A056-07 | 9 | 7 | 61 | Primary solid Tumor |
| TCGA-AO-A0JL-01A-11R-A056-07 | 10 | 8 | 61 | Primary solid Tumor |
| TCGA-AO-A0JM-01A-21R-A056-07 | 9 | 7 | 61 | Primary solid Tumor |
| TCGA-AO-A124-01A-11R-A10J-07 | 10 | 8 | 80 | Primary solid Tumor |
| TCGA-AO-A125-01A-11R-A10J-07 | 9 | 7 | 80 | Primary solid Tumor |
| TCGA-AO-A126-01A-11R-A10J-07 | 9 | 7 | 80 | Primary solid Tumor |
| TCGA-AO-A128-01A-11R-A10J-07 | 10 | 8 | 80 | Primary solid Tumor |
| TCGA-AO-A129-01A-21R-A10J-07 | 10 | 8 | 80 | Primary solid Tumor |
| TCGA-AO-A12A-01A-21R-A115-07 | 9 | 7 | 85 | Primary solid Tumor |
| TCGA-AO-A12B-01A-11R-A10J-07 | 9 | 7 | 80 | Primary solid Tumor |
| TCGA-AO-A12C-01A-11R-A10J-07 | 9 | 7 | 80 | Primary solid Tumor |
| TCGA-AO-A12D-01A-11R-A115-07 | 9 | 7 | 85 | Primary solid Tumor |
| TCGA-AO-A12E-01A-11R-A10J-07 | 9 | 7 | 80 | Primary solid Tumor |
| TCGA-AO-A12F-01A-11R-A115-07 | 10 | 8 | 85 | Primary solid Tumor |
| TCGA-AO-A12G-01A-11R-A10J-07 | 9 | 7 | 80 | Primary solid Tumor |
| TCGA-AO-A12H-01A-11R-A115-07 | 9 | 7 | 85 | Primary solid Tumor |
| TCGA-AO-A1KO-01A-31R-A13Q-07 | 9 | 7 | 109 | Primary solid Tumor |
| TCGA-AO-A1KP-01A-11R-A13Q-07 | 9 | 7 | 109 | Primary solid Tumor |
| TCGA-AO-A1KQ-01A-11R-A13Q-07 | 9 | 7 | 109 | Primary solid Tumor |
| TCGA-AO-A1KR-01A-12R-A144-07 | 10 | 8 | 117 | Primary solid Tumor |
| TCGA-AO-A1KS-01A-11R-A13Q-07 | 9 | 7 | 109 | Primary solid Tumor |
| TCGA-AO-A1KT-01A-11R-A13Q-07 | 9 | 7 | 109 | Primary solid Tumor |
| TCGA-AP-A051-01A-21R-A00V-07 | 16 | 11 | 49 | Primary solid Tumor |
| TCGA-AP-A052-01A-11R-A00V-07 | 16 | 11 | 49 | Primary solid Tumor |
| TCGA-AP-A053-01A-21R-A00V-07 | 16 | 11 | 49 | Primary solid Tumor |
| TCGA-AP-A054-01A-11R-A16W-07 | 3 | 6 | 59 | Primary solid Tumor |
| TCGA-AP-A056-01A-11R-A00V-07 | 16 | 11 | 49 | Primary solid Tumor |
| TCGA-AP-A059-01A-21R-A118-07 | 16 | 11 | 92 | Primary solid Tumor |
| TCGA-AP-A05A-01A-11R-A00V-07 | 16 | 11 | 49 | Primary solid Tumor |
| TCGA-AP-A05D-01A-11R-A00V-07 | 16 | 11 | 49 | Primary solid Tumor |
| TCGA-AP-A05H-01A-11R-A00V-07 | 16 | 11 | 49 | Primary solid Tumor |
| TCGA-AP-A05J-01A-11R-A00V-07 | 16 | 11 | 49 | Primary solid Tumor |
| TCGA-AP-A05N-01A-11R-A00V-07 | 16 | 11 | 49 | Primary solid Tumor |
| TCGA-AP-A05O-01A-12R-A118-07 | 16 | 11 | 92 | Primary solid Tumor |
| TCGA-AP-A05P-01A-11R-A16W-07 | 16 | 11 | 59 | Primary solid Tumor |
| TCGA-AP-A0L8-01A-11R-A040-07 | 16 | 11 | 59 | Primary solid Tumor |
| TCGA-AP-A0L9-01A-11R-A040-07 | 16 | 11 | 59 | Primary solid Tumor |
| TCGA-AP-A0LD-01A-11R-A040-07 | 16 | 11 | 59 | Primary solid Tumor |
| TCGA-AP-A0LE-01A-11R-A104-07 | 16 | 11 | 73 | Primary solid Tumor |
| TCGA-AP-A0LF-01A-11R-A118-07 | 16 | 11 | 92 | Primary solid Tumor |
| TCGA-AP-A0LG-01A-11R-A16W-07 | 16 | 11 | 59 | Primary solid Tumor |
| TCGA-AP-A0LH-01A-11R-A16W-07 | 16 | 15 | 59 | Primary solid Tumor |
| TCGA-AP-A0LI-01A-11R-A040-07 | 16 | 11 | 59 | Primary solid Tumor |
| TCGA-AP-A0LJ-01A-11R-A040-07 | 16 | 11 | 59 | Primary solid Tumor |
| TCGA-AP-A0LL-01A-12R-A104-07 | 16 | 11 | 73 | Primary solid Tumor |
| TCGA-AP-A0LM-01A-11R-A118-07 | 16 | 11 | 92 | Primary solid Tumor |
| TCGA-AP-A0LN-01A-11R-A040-07 | 16 | 11 | 59 | Primary solid Tumor |
| TCGA-AP-A0LO-01A-11R-A040-07 | 16 | 11 | 59 | Primary solid Tumor |
| TCGA-AP-A0LP-01A-12R-A104-07 | 16 | 11 | 73 | Primary solid Tumor |
| TCGA-AP-A0LS-01A-11R-A14D-07 | 16 | 11 | 121 | Primary solid Tumor |
| TCGA-AP-A0LT-01A-11R-A040-07 | 16 | 11 | 59 | Primary solid Tumor |
| TCGA-AP-A0LV-01A-11R-A040-07 | 16 | 11 | 59 | Primary solid Tumor |
| TCGA-AP-A1DH-01A-31R-A137-07 | 16 | 11 | 104 | Primary solid Tumor |
| TCGA-AP-A1DK-01A-11R-A137-07 | 16 | 11 | 104 | Primary solid Tumor |
| TCGA-AP-A1DM-01A-21R-A137-07 | 16 | 11 | 104 | Primary solid Tumor |
| TCGA-AP-A1DO-01A-11R-A137-07 | 16 | 11 | 104 | Primary solid Tumor |
| TCGA-AP-A1DP-01A-11R-A137-07 | 16 | 11 | 104 | Primary solid Tumor |
| TCGA-AP-A1DQ-01A-11R-A137-07 | 16 | 11 | 104 | Primary solid Tumor |
| TCGA-AP-A1DR-01A-11R-A137-07 | 16 | 11 | 104 | Primary solid Tumor |
| TCGA-AP-A1DV-01A-21R-A137-07 | 16 | 11 | 104 | Primary solid Tumor |
| TCGA-AP-A1E0-01A-11R-A137-07 | 16 | 11 | 104 | Primary solid Tumor |
| TCGA-AP-A1E1-01A-11R-A137-07 | 16 | 11 | 104 | Primary solid Tumor |
| TCGA-AP-A1E3-01A-11R-A137-07 | 16 | 11 | 104 | Primary solid Tumor |
| TCGA-AP-A1E4-01A-12R-A137-07 | 16 | 11 | 104 | Primary solid Tumor |
| TCGA-AP-A3K1-01A-11R-A213-07 | 16 | NA | 201 | Primary solid Tumor |
| TCGA-AP-A5FX-01A-11R-A27V-07 | 16 | NA | 289 | Primary solid Tumor |
| TCGA-AQ-A04H-01B-11R-A10J-07 | 9 | 7 | 80 | Primary solid Tumor |
| TCGA-AQ-A04J-01A-02R-A034-07 | 10 | 8 | 56 | Primary solid Tumor |
| TCGA-AQ-A04L-01B-21R-A10J-07 | 9 | 7 | 80 | Primary solid Tumor |
| TCGA-AQ-A0Y5-01A-11R-A14M-07 | 9 | 7 | 124 | Primary solid Tumor |
| TCGA-AQ-A1H2-01A-11R-A13Q-07 | 9 | 7 | 109 | Primary solid Tumor |
| TCGA-AQ-A1H3-01A-31R-A13Q-07 | 9 | 7 | 109 | Primary solid Tumor |
| TCGA-AQ-A54N-01A-11R-A266-07 | 10 | NA | 271 | Primary solid Tumor |
| TCGA-AQ-A54O-01A-11R-A266-07 | 9 | NA | 271 | Primary solid Tumor |
| TCGA-AQ-A7U7-01A-22R-A352-07 | 9 | NA | 360 | Primary solid Tumor |
| TCGA-AR-A0TP-01A-11R-A084-07 | 10 | 8 | 72 | Primary solid Tumor |
| TCGA-AR-A0TQ-01A-11R-A084-07 | 9 | 7 | 72 | Primary solid Tumor |
| TCGA-AR-A0TR-01A-11R-A084-07 | 9 | 7 | 72 | Primary solid Tumor |
| TCGA-AR-A0TS-01A-11R-A115-07 | 10 | 8 | 85 | Primary solid Tumor |
| TCGA-AR-A0TT-01A-31R-A084-07 | 9 | 7 | 72 | Primary solid Tumor |
| TCGA-AR-A0TU-01A-31R-A109-07 | 10 | 8 | 74 | Primary solid Tumor |
| TCGA-AR-A0TV-01A-21R-A084-07 | 9 | 7 | 72 | Primary solid Tumor |
| TCGA-AR-A0TW-01A-11R-A084-07 | 9 | 7 | 72 | Primary solid Tumor |
| TCGA-AR-A0TX-01A-11R-A084-07 | 9 | 7 | 72 | Primary solid Tumor |
| TCGA-AR-A0TY-01A-12R-A115-07 | 9 | 7 | 85 | Primary solid Tumor |
| TCGA-AR-A0TZ-01A-12R-A084-07 | 9 | 7 | 72 | Primary solid Tumor |
| TCGA-AR-A0U0-01A-11R-A109-07 | 10 | 8 | 74 | Primary solid Tumor |
| TCGA-AR-A0U2-01A-11R-A109-07 | 9 | 7 | 74 | Primary solid Tumor |
| TCGA-AR-A0U3-01A-11R-A109-07 | 9 | 7 | 74 | Primary solid Tumor |
| TCGA-AR-A0U4-01A-11R-A109-07 | 10 | 8 | 74 | Primary solid Tumor |
| TCGA-AR-A1AH-01A-11R-A12D-07 | 10 | 8 | 93 | Primary solid Tumor |
| TCGA-AR-A1AI-01A-11R-A12P-07 | 10 | 8 | 96 | Primary solid Tumor |
| TCGA-AR-A1AJ-01A-21R-A12P-07 | 10 | 8 | 96 | Primary solid Tumor |
| TCGA-AR-A1AK-01A-21R-A12P-07 | 9 | 7 | 96 | Primary solid Tumor |
| TCGA-AR-A1AL-01A-21R-A12P-07 | 9 | 7 | 96 | Primary solid Tumor |
| TCGA-AR-A1AM-01A-41R-A22K-07 | 9 | NA | 227 | Primary solid Tumor |
| TCGA-AR-A1AN-01A-11R-A12P-07 | 9 | 7 | 96 | Primary solid Tumor |
| TCGA-AR-A1AO-01A-11R-A12P-07 | 9 | 7 | 96 | Primary solid Tumor |
| TCGA-AR-A1AP-01A-11R-A12P-07 | 9 | 7 | 96 | Primary solid Tumor |
| TCGA-AR-A1AQ-01A-11R-A12P-07 | 10 | 8 | 96 | Primary solid Tumor |
| TCGA-AR-A1AR-01A-31R-A137-07 | 10 | 8 | 103 | Primary solid Tumor |
| TCGA-AR-A1AS-01A-11R-A12P-07 | 9 | 7 | 96 | Primary solid Tumor |
| TCGA-AR-A1AT-01A-11R-A12P-07 | 9 | NA | 96 | Primary solid Tumor |
| TCGA-AR-A1AU-01A-11R-A12P-07 | 9 | 7 | 96 | Primary solid Tumor |
| TCGA-AR-A1AV-01A-21R-A12P-07 | 9 | 7 | 96 | Primary solid Tumor |
| TCGA-AR-A1AW-01A-21R-A12P-07 | 9 | 7 | 96 | Primary solid Tumor |
| TCGA-AR-A1AX-01A-11R-A12P-07 | 9 | 7 | 96 | Primary solid Tumor |
| TCGA-AR-A1AY-01A-21R-A12P-07 | 10 | 8 | 96 | Primary solid Tumor |
| TCGA-AR-A24H-01A-11R-A169-07 | 9 | 7 | 142 | Primary solid Tumor |
| TCGA-AR-A24K-01A-11R-A169-07 | 9 | 7 | 142 | Primary solid Tumor |
| TCGA-AR-A24L-01A-11R-A169-07 | 9 | 7 | 142 | Primary solid Tumor |
| TCGA-AR-A24M-01A-11R-A169-07 | 9 | 7 | 142 | Primary solid Tumor |
| TCGA-AR-A24N-01A-11R-A169-07 | 9 | 7 | 142 | Primary solid Tumor |
| TCGA-AR-A24O-01A-11R-A169-07 | 9 | 7 | 142 | Primary solid Tumor |
| TCGA-AR-A24P-01A-11R-A169-07 | 9 | 7 | 142 | Primary solid Tumor |
| TCGA-AR-A24Q-01A-12R-A169-07 | 10 | 8 | 142 | Primary solid Tumor |
| TCGA-AR-A24R-01A-11R-A169-07 | 9 | 7 | 142 | Primary solid Tumor |
| TCGA-AR-A24S-01A-11R-A169-07 | 9 | 7 | 142 | Primary solid Tumor |
| TCGA-AR-A24T-01A-11R-A169-07 | 9 | 7 | 142 | Primary solid Tumor |
| TCGA-AR-A24U-01A-11R-A169-07 | 9 | 7 | 142 | Primary solid Tumor |
| TCGA-AR-A24V-01A-21R-A169-07 | 9 | 7 | 142 | Primary solid Tumor |
| TCGA-AR-A24W-01A-11R-A169-07 | 9 | 7 | 142 | Primary solid Tumor |
| TCGA-AR-A24X-01A-11R-A169-07 | 9 | 7 | 142 | Primary solid Tumor |
| TCGA-AR-A24Z-01A-11R-A169-07 | 9 | 7 | 142 | Primary solid Tumor |
| TCGA-AR-A250-01A-31R-A169-07 | 9 | 7 | 142 | Primary solid Tumor |
| TCGA-AR-A251-01A-12R-A169-07 | 10 | 8 | 142 | Primary solid Tumor |
| TCGA-AR-A252-01A-11R-A169-07 | 9 | 7 | 142 | Primary solid Tumor |
| TCGA-AR-A254-01A-21R-A169-07 | 9 | 7 | 142 | Primary solid Tumor |
| TCGA-AR-A255-01A-11R-A169-07 | 9 | 7 | 142 | Primary solid Tumor |
| TCGA-AR-A256-01A-11R-A169-07 | 10 | 8 | 142 | Primary solid Tumor |
| TCGA-AR-A2LE-01A-11R-A180-07 | 9 | 7 | 167 | Primary solid Tumor |
| TCGA-AR-A2LH-01A-31R-A18M-07 | 9 | 7 | 177 | Primary solid Tumor |
| TCGA-AR-A2LJ-01A-12R-A19W-07 | 9 | NA | 185 | Primary solid Tumor |
| TCGA-AR-A2LK-01A-11R-A180-07 | 9 | 7 | 167 | Primary solid Tumor |
| TCGA-AR-A2LL-01A-11R-A180-07 | 9 | 7 | 167 | Primary solid Tumor |
| TCGA-AR-A2LM-01A-11R-A180-07 | 9 | 7 | 167 | Primary solid Tumor |
| TCGA-AR-A2LN-01A-21R-A18M-07 | 9 | 7 | 177 | Primary solid Tumor |
| TCGA-AR-A2LO-01A-31R-A18M-07 | 9 | 7 | 177 | Primary solid Tumor |
| TCGA-AR-A2LQ-01A-22R-A18M-07 | 9 | 7 | 177 | Primary solid Tumor |
| TCGA-AR-A2LR-01A-12R-A18M-07 | 10 | 8 | 177 | Primary solid Tumor |
| TCGA-AR-A5QM-01A-11R-A27Q-07 | 9 | NA | 288 | Primary solid Tumor |
| TCGA-AR-A5QN-01A-12R-A28M-07 | 9 | NA | 296 | Primary solid Tumor |
| TCGA-AR-A5QP-01A-11R-A28M-07 | 9 | NA | 296 | Primary solid Tumor |
| TCGA-AR-A5QQ-01A-11R-A28M-07 | 2 | NA | 296 | Primary solid Tumor |
| TCGA-AS-3777-01A-01R-0864-07 | 14 | 9 | 32 | Primary solid Tumor |
| TCGA-AS-3778-01A-01R-A32Z-07 | 14 | NA | 32 | Primary solid Tumor |
| TCGA-AU-3779-01A-01R-1723-07 | 7 | NA | 116 | Primary solid Tumor |
| TCGA-AU-6004-01A-11R-1723-07 | 7 | NA | 116 | Primary solid Tumor |
| TCGA-AW-A1PO-01A-12R-A157-07 | 16 | 11 | 137 | Primary solid Tumor |
| TCGA-AX-A05S-01A-11R-A00V-07 | 16 | 11 | 49 | Primary solid Tumor |
| TCGA-AX-A05T-01A-11R-A00V-07 | 16 | 11 | 49 | Primary solid Tumor |
| TCGA-AX-A05U-01A-11R-A00V-07 | 16 | 11 | 49 | Primary solid Tumor |
| TCGA-AX-A05W-01A-21R-A466-07 | 16 | NA | 49 | Primary solid Tumor |
| TCGA-AX-A05Y-01A-11R-A00V-07 | 16 | 11 | 49 | Primary solid Tumor |
| TCGA-AX-A05Z-01A-11R-A00V-07 | 16 | 11 | 49 | Primary solid Tumor |
| TCGA-AX-A060-01A-11R-A00V-07 | 16 | 11 | 49 | Primary solid Tumor |
| TCGA-AX-A062-01A-11R-A00V-07 | 16 | 11 | 49 | Primary solid Tumor |
| TCGA-AX-A063-01A-11R-A00V-07 | 16 | 11 | 49 | Primary solid Tumor |
| TCGA-AX-A064-01A-11R-A00V-07 | 16 | 11 | 49 | Primary solid Tumor |
| TCGA-AX-A06B-01A-11R-A00V-07 | 16 | 11 | 49 | Primary solid Tumor |
| TCGA-AX-A06D-01A-11R-A118-07 | 16 | 11 | 92 | Primary solid Tumor |
| TCGA-AX-A06F-01A-11R-A00V-07 | 16 | 11 | 49 | Primary solid Tumor |
| TCGA-AX-A06H-01A-11R-A118-07 | 16 | 11 | 92 | Primary solid Tumor |
| TCGA-AX-A06J-01A-11R-A00V-07 | 16 | 11 | 49 | Primary solid Tumor |
| TCGA-AX-A06L-01A-11R-A118-07 | 16 | 11 | 92 | Primary solid Tumor |
| TCGA-AX-A0IS-01A-12R-A10J-07 | 16 | 11 | 81 | Primary solid Tumor |
| TCGA-AX-A0IU-01A-11R-A16W-07 | 16 | 11 | 73 | Primary solid Tumor |
| TCGA-AX-A0IW-01A-11R-A040-07 | 16 | 11 | 59 | Primary solid Tumor |
| TCGA-AX-A0IZ-01A-11R-A118-07 | 16 | 11 | 92 | Primary solid Tumor |
| TCGA-AX-A0J0-01A-11R-A109-07 | 16 | 11 | 75 | Primary solid Tumor |
| TCGA-AX-A0J1-01A-11R-A040-07 | 16 | 11 | 59 | Primary solid Tumor |
| TCGA-AX-A1C4-01A-11R-A137-07 | 16 | 11 | 104 | Primary solid Tumor |
| TCGA-AX-A1C5-01A-11R-A137-07 | 16 | 11 | 104 | Primary solid Tumor |
| TCGA-AX-A1C7-01A-11R-A137-07 | 16 | 11 | 104 | Primary solid Tumor |
| TCGA-AX-A1C8-01A-11R-A137-07 | 16 | 11 | 104 | Primary solid Tumor |
| TCGA-AX-A1C9-01A-11R-A137-07 | 16 | 11 | 104 | Primary solid Tumor |
| TCGA-AX-A1CA-01A-12R-A137-07 | 16 | 11 | 104 | Primary solid Tumor |
| TCGA-AX-A1CC-01A-11R-A137-07 | 16 | 11 | 104 | Primary solid Tumor |
| TCGA-AX-A1CE-01A-11R-A137-07 | 16 | 11 | 104 | Primary solid Tumor |
| TCGA-AX-A1CF-01A-11R-A137-07 | 16 | 11 | 104 | Primary solid Tumor |
| TCGA-AX-A1CI-01A-11R-A137-07 | 16 | 11 | 104 | Primary solid Tumor |
| TCGA-AX-A1CJ-01A-11R-A137-07 | 16 | 11 | 104 | Primary solid Tumor |
| TCGA-AX-A1CK-01A-11R-A137-07 | 16 | 11 | 104 | Primary solid Tumor |
| TCGA-AX-A1CN-01A-11R-A137-07 | 4 | 3 | 104 | Primary solid Tumor |
| TCGA-AX-A1CP-01A-11R-A137-07 | 16 | 11 | 104 | Primary solid Tumor |
| TCGA-AX-A1CR-01A-12R-A137-07 | 16 | 11 | 104 | Primary solid Tumor |
| TCGA-AX-A2H2-01A-11R-A180-07 | 16 | 11 | 168 | Primary solid Tumor |
| TCGA-AX-A2H4-01A-21R-A18M-07 | 16 | NA | 178 | Primary solid Tumor |
| TCGA-AX-A2H5-01A-11R-A17B-07 | 16 | 11 | 156 | Primary solid Tumor |
| TCGA-AX-A2H7-01A-12R-A18M-07 | 16 | 11 | 178 | Primary solid Tumor |
| TCGA-AX-A2H8-01A-11R-A17B-07 | 16 | 11 | 156 | Primary solid Tumor |
| TCGA-AX-A2HA-01A-12R-A18M-07 | 16 | 11 | 178 | Primary solid Tumor |
| TCGA-AX-A2HC-01A-11R-A17B-07 | 3 | 6 | 156 | Primary solid Tumor |
| TCGA-AX-A2HD-01A-21R-A17B-07 | 16 | 11 | 156 | Primary solid Tumor |
| TCGA-AX-A2HF-01A-11R-A17B-07 | 16 | 11 | 156 | Primary solid Tumor |
| TCGA-AX-A2HG-01A-11R-A17B-07 | 16 | 11 | 156 | Primary solid Tumor |
| TCGA-AX-A2HH-01A-11R-A32Y-07 | 16 | NA | 156 | Primary solid Tumor |
| TCGA-AX-A2HJ-01A-11R-A17B-07 | 16 | 11 | 156 | Primary solid Tumor |
| TCGA-AX-A2HK-01A-11R-A17B-07 | 16 | 11 | 156 | Primary solid Tumor |
| TCGA-AX-A2IN-01A-12R-A180-07 | 3 | NA | 168 | Primary solid Tumor |
| TCGA-AX-A2IO-01A-11R-A180-07 | 16 | 11 | 168 | Primary solid Tumor |
| TCGA-AX-A3FS-01A-11R-A22K-07 | 3 | NA | 228 | Primary solid Tumor |
| TCGA-AX-A3FT-01A-11R-A22K-07 | 16 | NA | 228 | Primary solid Tumor |
| TCGA-AX-A3FV-01A-11R-A22K-07 | 8 | NA | 228 | Primary solid Tumor |
| TCGA-AX-A3FW-01A-11R-A22K-07 | 16 | NA | 228 | Primary solid Tumor |
| TCGA-AX-A3FX-01A-11R-A22K-07 | 16 | NA | 228 | Primary solid Tumor |
| TCGA-AX-A3FZ-01A-11R-A22K-07 | 3 | NA | 228 | Primary solid Tumor |
| TCGA-AX-A3G1-01A-11R-A22K-07 | 16 | NA | 228 | Primary solid Tumor |
| TCGA-AX-A3G3-01A-11R-A213-07 | 20 | NA | 201 | Primary solid Tumor |
| TCGA-AX-A3G4-01A-11R-A213-07 | 16 | NA | 201 | Primary solid Tumor |
| TCGA-AX-A3G6-01A-11R-A213-07 | 16 | NA | 201 | Primary solid Tumor |
| TCGA-AX-A3G7-01A-12R-A213-07 | 20 | NA | 201 | Primary solid Tumor |
| TCGA-AX-A3G8-01A-11R-A22K-07 | 16 | NA | 228 | Primary solid Tumor |
| TCGA-AX-A3G9-01A-11R-A22K-07 | 16 | NA | 228 | Primary solid Tumor |
| TCGA-AX-A3GB-01A-11R-A22K-07 | 16 | NA | 228 | Primary solid Tumor |
| TCGA-AX-A3GI-01A-11R-A213-07 | 16 | NA | 201 | Primary solid Tumor |
| TCGA-AY-4070-01A-01R-1113-07 | 7 | 15 | 45 | Primary solid Tumor |
| TCGA-AY-4071-01A-01R-1113-07 | 7 | 15 | 45 | Primary solid Tumor |
| TCGA-AY-5543-01A-01R-1653-07 | 7 | NA | 89 | Primary solid Tumor |
| TCGA-AY-6196-01A-11R-1723-07 | 7 | NA | 116 | Primary solid Tumor |
| TCGA-AY-6197-01A-11R-1723-07 | 7 | NA | 116 | Primary solid Tumor |
| TCGA-AY-6386-01A-21R-1723-07 | 7 | NA | 116 | Primary solid Tumor |
| TCGA-AY-A54L-01A-11R-A28H-07 | 7 | NA | 300 | Primary solid Tumor |
| TCGA-AY-A69D-01A-11R-A37K-07 | 7 | NA | 385 | Primary solid Tumor |
| TCGA-AY-A71X-01A-12R-A37K-07 | 7 | NA | 385 | Primary solid Tumor |
| TCGA-AY-A8YK-01A-11R-A41B-07 | 7 | NA | 422 | Primary solid Tumor |
| TCGA-AZ-4308-01A-01R-1410-07 | 7 | 15 | 76 | Primary solid Tumor |
| TCGA-AZ-4313-01A-01R-1410-07 | 7 | 15 | 76 | Primary solid Tumor |
| TCGA-AZ-4315-01A-01R-1410-07 | 7 | 15 | 76 | Primary solid Tumor |
| TCGA-AZ-4323-01A-21R-1839-07 | 3 | NA | 138 | Primary solid Tumor |
| TCGA-AZ-4614-01A-01R-1410-07 | 7 | 15 | 76 | Primary solid Tumor |
| TCGA-AZ-4615-01A-01R-1410-07 | 7 | 15 | 76 | Primary solid Tumor |
| TCGA-AZ-4616-01A-21R-1839-07 | 7 | NA | 138 | Primary solid Tumor |
| TCGA-AZ-4681-01A-01R-1410-07 | 7 | 15 | 76 | Primary solid Tumor |
| TCGA-AZ-4682-01B-01R-A32Z-07 | 7 | NA | 76 | Primary solid Tumor |
| TCGA-AZ-4684-01A-01R-1410-07 | 7 | 15 | 76 | Primary solid Tumor |
| TCGA-AZ-5403-01A-01R-1653-07 | 7 | NA | 89 | Primary solid Tumor |
| TCGA-AZ-5407-01A-01R-1723-07 | 7 | NA | 116 | Primary solid Tumor |
| TCGA-AZ-6598-01A-11R-1774-07 | 7 | NA | 123 | Primary solid Tumor |
| TCGA-AZ-6599-01A-11R-1774-07 | 7 | NA | 123 | Primary solid Tumor |
| TCGA-AZ-6600-01A-11R-1774-07 | 7 | NA | 123 | Primary solid Tumor |
| TCGA-AZ-6601-01A-11R-1774-07 | 7 | NA | 123 | Primary solid Tumor |
| TCGA-AZ-6603-01A-11R-1839-07 | 7 | NA | 138 | Primary solid Tumor |
| TCGA-AZ-6605-01A-11R-1839-07 | 7 | NA | 138 | Primary solid Tumor |
| TCGA-AZ-6606-01A-11R-1839-07 | 7 | NA | 138 | Primary solid Tumor |
| TCGA-AZ-6607-01A-11R-1839-07 | 7 | NA | 138 | Primary solid Tumor |
| TCGA-AZ-6608-01A-11R-1839-07 | 7 | NA | 138 | Primary solid Tumor |
| TCGA-B0-4688-01A-01R-1277-07 | 3 | 6 | 63 | Primary solid Tumor |
| TCGA-B0-4690-01A-01R-1277-07 | 14 | 9 | 63 | Primary solid Tumor |
| TCGA-B0-4691-01A-01R-1277-07 | 14 | 9 | 63 | Primary solid Tumor |
| TCGA-B0-4693-01A-01R-1277-07 | 14 | 9 | 63 | Primary solid Tumor |
| TCGA-B0-4694-01A-01R-1277-07 | 14 | 9 | 63 | Primary solid Tumor |
| TCGA-B0-4696-01A-01R-1277-07 | 3 | 6 | 63 | Primary solid Tumor |
| TCGA-B0-4697-01A-01R-1277-07 | 14 | 9 | 63 | Primary solid Tumor |
| TCGA-B0-4698-01A-01R-1503-07 | 3 | 2 | 82 | Primary solid Tumor |
| TCGA-B0-4699-01A-01R-1277-07 | 14 | 9 | 63 | Primary solid Tumor |
| TCGA-B0-4700-01A-02R-1541-07 | 14 | 9 | 90 | Primary solid Tumor |
| TCGA-B0-4701-01A-01R-1277-07 | 14 | 9 | 63 | Primary solid Tumor |
| TCGA-B0-4703-01A-01R-1277-07 | 14 | 9 | 63 | Primary solid Tumor |
| TCGA-B0-4706-01A-01R-1503-07 | 14 | 9 | 82 | Primary solid Tumor |
| TCGA-B0-4707-01A-01R-1277-07 | 14 | 9 | 63 | Primary solid Tumor |
| TCGA-B0-4710-01A-01R-1503-07 | 14 | NA | 82 | Primary solid Tumor |
| TCGA-B0-4712-01A-01R-1503-07 | 14 | 9 | 82 | Primary solid Tumor |
| TCGA-B0-4713-01A-01R-1277-07 | 14 | 9 | 63 | Primary solid Tumor |
| TCGA-B0-4714-01A-01R-1277-07 | 14 | 9 | 63 | Primary solid Tumor |
| TCGA-B0-4718-01A-01R-1277-07 | 14 | 9 | 63 | Primary solid Tumor |
| TCGA-B0-4810-01A-01R-1503-07 | 14 | 9 | 82 | Primary solid Tumor |
| TCGA-B0-4811-01A-01R-1503-07 | 14 | 9 | 82 | Primary solid Tumor |
| TCGA-B0-4813-01A-01R-1277-07 | 14 | 9 | 63 | Primary solid Tumor |
| TCGA-B0-4814-01A-01R-1277-07 | 14 | 9 | 63 | Primary solid Tumor |
| TCGA-B0-4815-01A-01R-1503-07 | 14 | 9 | 82 | Primary solid Tumor |
| TCGA-B0-4816-01A-01R-1503-07 | 14 | 9 | 82 | Primary solid Tumor |
| TCGA-B0-4817-01A-01R-1277-07 | 14 | 9 | 63 | Primary solid Tumor |
| TCGA-B0-4818-01A-01R-1503-07 | 14 | 9 | 82 | Primary solid Tumor |
| TCGA-B0-4819-01A-01R-1277-07 | 14 | 9 | 63 | Primary solid Tumor |
| TCGA-B0-4821-01A-01R-1503-07 | 14 | 9 | 82 | Primary solid Tumor |
| TCGA-B0-4822-01A-01R-1277-07 | 14 | NA | 63 | Primary solid Tumor |
| TCGA-B0-4823-01A-02R-1420-07 | 14 | 9 | 68 | Primary solid Tumor |
| TCGA-B0-4824-01A-01R-1277-07 | 14 | 9 | 63 | Primary solid Tumor |
| TCGA-B0-4827-01A-02R-1420-07 | 14 | 9 | 68 | Primary solid Tumor |
| TCGA-B0-4828-01A-01R-1277-07 | 14 | 9 | 63 | Primary solid Tumor |
| TCGA-B0-4833-01A-01R-1305-07 | 14 | 9 | 65 | Primary solid Tumor |
| TCGA-B0-4834-01A-01R-1305-07 | 14 | 9 | 65 | Primary solid Tumor |
| TCGA-B0-4836-01A-01R-1305-07 | 14 | 9 | 65 | Primary solid Tumor |
| TCGA-B0-4837-01A-01R-1305-07 | 14 | 9 | 65 | Primary solid Tumor |
| TCGA-B0-4838-01A-01R-1305-07 | 14 | 9 | 65 | Primary solid Tumor |
| TCGA-B0-4839-01A-01R-1305-07 | 14 | 9 | 65 | Primary solid Tumor |
| TCGA-B0-4841-01A-01R-1277-07 | 14 | 9 | 63 | Primary solid Tumor |
| TCGA-B0-4842-01A-02R-1420-07 | 14 | 9 | 68 | Primary solid Tumor |
| TCGA-B0-4843-01A-01R-1277-07 | 14 | 9 | 63 | Primary solid Tumor |
| TCGA-B0-4844-01A-01R-1277-07 | 14 | 9 | 63 | Primary solid Tumor |
| TCGA-B0-4845-01A-01R-1277-07 | 14 | 9 | 63 | Primary solid Tumor |
| TCGA-B0-4846-01A-01R-1277-07 | 14 | 9 | 63 | Primary solid Tumor |
| TCGA-B0-4847-01A-01R-1277-07 | 14 | 9 | 63 | Primary solid Tumor |
| TCGA-B0-4848-01A-01R-1277-07 | 14 | 9 | 63 | Primary solid Tumor |
| TCGA-B0-4849-01A-01R-1277-07 | 14 | 9 | 63 | Primary solid Tumor |
| TCGA-B0-4852-01A-01R-1503-07 | 14 | 9 | 82 | Primary solid Tumor |
| TCGA-B0-4945-01A-01R-1420-07 | 14 | 9 | 68 | Primary solid Tumor |
| TCGA-B0-5075-01A-01R-1334-07 | 14 | 9 | 69 | Primary solid Tumor |
| TCGA-B0-5077-01A-01R-1334-07 | 14 | 9 | 69 | Primary solid Tumor |
| TCGA-B0-5080-01A-01R-1503-07 | 14 | 9 | 82 | Primary solid Tumor |
| TCGA-B0-5081-01A-01R-1334-07 | 14 | 9 | 69 | Primary solid Tumor |
| TCGA-B0-5083-01A-02R-1420-07 | 14 | 9 | 68 | Primary solid Tumor |
| TCGA-B0-5084-01A-01R-1334-07 | 14 | 9 | 69 | Primary solid Tumor |
| TCGA-B0-5085-01A-01R-1334-07 | 14 | 9 | 69 | Primary solid Tumor |
| TCGA-B0-5088-01A-01R-1334-07 | 14 | 9 | 69 | Primary solid Tumor |
| TCGA-B0-5092-01A-01R-1420-07 | 14 | 9 | 68 | Primary solid Tumor |
| TCGA-B0-5094-01A-01R-1420-07 | 14 | 9 | 68 | Primary solid Tumor |
| TCGA-B0-5095-01A-01R-1420-07 | 14 | 9 | 68 | Primary solid Tumor |
| TCGA-B0-5096-01A-01R-1420-07 | 14 | 9 | 68 | Primary solid Tumor |
| TCGA-B0-5097-01A-01R-1420-07 | 14 | 9 | 68 | Primary solid Tumor |
| TCGA-B0-5098-01A-01R-1420-07 | 14 | 9 | 68 | Primary solid Tumor |
| TCGA-B0-5099-01A-01R-1420-07 | 14 | 9 | 68 | Primary solid Tumor |
| TCGA-B0-5100-01A-01R-1420-07 | 14 | 9 | 68 | Primary solid Tumor |
| TCGA-B0-5102-01A-01R-1420-07 | 14 | 9 | 68 | Primary solid Tumor |
| TCGA-B0-5104-01A-01R-1420-07 | 14 | 9 | 68 | Primary solid Tumor |
| TCGA-B0-5106-01A-01R-1420-07 | 14 | 9 | 68 | Primary solid Tumor |
| TCGA-B0-5107-01A-01R-1420-07 | 14 | 9 | 68 | Primary solid Tumor |
| TCGA-B0-5108-01A-01R-1420-07 | 14 | 9 | 68 | Primary solid Tumor |
| TCGA-B0-5109-01A-02R-1420-07 | 14 | 9 | 68 | Primary solid Tumor |
| TCGA-B0-5110-01A-01R-1420-07 | 14 | 9 | 68 | Primary solid Tumor |
| TCGA-B0-5113-01A-01R-1420-07 | 14 | 9 | 68 | Primary solid Tumor |
| TCGA-B0-5115-01A-01R-1420-07 | 14 | 9 | 68 | Primary solid Tumor |
| TCGA-B0-5116-01A-02R-1420-07 | 14 | 9 | 68 | Primary solid Tumor |
| TCGA-B0-5117-01A-01R-1420-07 | 14 | 9 | 68 | Primary solid Tumor |
| TCGA-B0-5119-01A-02R-1420-07 | 14 | 9 | 68 | Primary solid Tumor |
| TCGA-B0-5120-01A-01R-1420-07 | 14 | 9 | 68 | Primary solid Tumor |
| TCGA-B0-5121-01A-02R-1420-07 | 14 | 9 | 68 | Primary solid Tumor |
| TCGA-B0-5399-01A-01R-1503-07 | 14 | 9 | 82 | Primary solid Tumor |
| TCGA-B0-5400-01A-01R-1503-07 | 14 | 9 | 82 | Primary solid Tumor |
| TCGA-B0-5402-01A-01R-1503-07 | 14 | 9 | 82 | Primary solid Tumor |
| TCGA-B0-5690-01A-11R-1541-07 | 14 | 9 | 90 | Primary solid Tumor |
| TCGA-B0-5691-01A-11R-1541-07 | 14 | 9 | 90 | Primary solid Tumor |
| TCGA-B0-5692-01A-11R-1541-07 | 14 | 9 | 90 | Primary solid Tumor |
| TCGA-B0-5693-01A-11R-1541-07 | 14 | 9 | 90 | Primary solid Tumor |
| TCGA-B0-5694-01A-11R-1541-07 | 14 | 9 | 90 | Primary solid Tumor |
| TCGA-B0-5695-01A-11R-1541-07 | 14 | 9 | 90 | Primary solid Tumor |
| TCGA-B0-5696-01A-11R-1541-07 | 14 | 9 | 90 | Primary solid Tumor |
| TCGA-B0-5697-01A-11R-1541-07 | 14 | 9 | 90 | Primary solid Tumor |
| TCGA-B0-5698-01A-11R-1672-07 | 14 | 9 | 105 | Primary solid Tumor |
| TCGA-B0-5699-01A-11R-1541-07 | 14 | 9 | 90 | Primary solid Tumor |
| TCGA-B0-5700-01A-11R-1541-07 | 14 | 9 | 90 | Primary solid Tumor |
| TCGA-B0-5701-01A-11R-1541-07 | 14 | 9 | 90 | Primary solid Tumor |
| TCGA-B0-5702-01A-11R-1541-07 | 14 | NA | 90 | Primary solid Tumor |
| TCGA-B0-5703-01A-11R-1541-07 | 14 | 9 | 90 | Primary solid Tumor |
| TCGA-B0-5705-01A-11R-1541-07 | 14 | 9 | 90 | Primary solid Tumor |
| TCGA-B0-5706-01A-11R-1541-07 | 14 | 9 | 90 | Primary solid Tumor |
| TCGA-B0-5707-01A-11R-1541-07 | 14 | NA | 90 | Primary solid Tumor |
| TCGA-B0-5709-01A-11R-1541-07 | 14 | 9 | 90 | Primary solid Tumor |
| TCGA-B0-5710-01A-11R-1672-07 | 14 | 9 | 105 | Primary solid Tumor |
| TCGA-B0-5711-01A-11R-1672-07 | 14 | 9 | 105 | Primary solid Tumor |
| TCGA-B0-5712-01A-11R-1672-07 | 14 | 9 | 105 | Primary solid Tumor |
| TCGA-B0-5713-01A-11R-1672-07 | 14 | 9 | 105 | Primary solid Tumor |
| TCGA-B0-5812-01A-11R-1672-07 | 14 | 9 | 105 | Primary solid Tumor |
| TCGA-B2-3923-01A-02R-1325-07 | 14 | 9 | 50 | Primary solid Tumor |
| TCGA-B2-3924-01A-02R-1325-07 | 14 | 9 | 50 | Primary solid Tumor |
| TCGA-B2-4098-01A-02R-1325-07 | 14 | 9 | 50 | Primary solid Tumor |
| TCGA-B2-4099-01A-02R-1188-07 | 14 | 9 | 50 | Primary solid Tumor |
| TCGA-B2-4101-01A-02R-1277-07 | 14 | 9 | 63 | Primary solid Tumor |
| TCGA-B2-4102-01A-02R-1325-07 | 14 | 9 | 50 | Primary solid Tumor |
| TCGA-B2-5633-01A-01R-1541-07 | 14 | 9 | 90 | Primary solid Tumor |
| TCGA-B2-5635-01A-01R-1541-07 | 14 | 9 | 90 | Primary solid Tumor |
| TCGA-B2-5636-01A-02R-1541-07 | 14 | 9 | 90 | Primary solid Tumor |
| TCGA-B2-5639-01A-01R-1541-07 | 14 | 9 | 90 | Primary solid Tumor |
| TCGA-B2-5641-01A-01R-1541-07 | 14 | 9 | 90 | Primary solid Tumor |
| TCGA-B2-A4SR-01A-11R-A266-07 | 14 | NA | 274 | Primary solid Tumor |
| TCGA-B4-5377-01A-01R-1503-07 | 14 | 9 | 82 | Primary solid Tumor |
| TCGA-B4-5378-01A-01R-1503-07 | 14 | 9 | 82 | Primary solid Tumor |
| TCGA-B4-5832-01A-11R-1672-07 | 14 | 9 | 105 | Primary solid Tumor |
| TCGA-B4-5834-01A-11R-1672-07 | 14 | 9 | 105 | Primary solid Tumor |
| TCGA-B4-5835-01A-11R-1672-07 | 14 | 9 | 105 | Primary solid Tumor |
| TCGA-B4-5836-01A-11R-1672-07 | 14 | 9 | 105 | Primary solid Tumor |
| TCGA-B4-5838-01A-11R-1672-07 | 14 | 9 | 105 | Primary solid Tumor |
| TCGA-B4-5843-01A-11R-1672-07 | 14 | 9 | 105 | Primary solid Tumor |
| TCGA-B4-5844-01A-11R-1672-07 | 14 | 9 | 105 | Primary solid Tumor |
| TCGA-B5-A0JN-01A-11R-A104-07 | 16 | NA | 73 | Primary solid Tumor |
| TCGA-B5-A0JR-01A-13R-A466-07 | 16 | NA | 59 | Primary solid Tumor |
| TCGA-B5-A0JS-01A-11R-A104-07 | 16 | 11 | 73 | Primary solid Tumor |
| TCGA-B5-A0JT-01A-21R-A118-07 | 16 | 11 | 92 | Primary solid Tumor |
| TCGA-B5-A0JU-01B-11R-A14D-07 | 16 | 11 | 121 | Primary solid Tumor |
| TCGA-B5-A0JV-01A-11R-A104-07 | 16 | 11 | 73 | Primary solid Tumor |
| TCGA-B5-A0JX-01A-21R-A14M-07 | 16 | 11 | 125 | Primary solid Tumor |
| TCGA-B5-A0JY-01A-11R-A104-07 | 16 | 11 | 73 | Primary solid Tumor |
| TCGA-B5-A0JZ-01A-11R-A040-07 | 16 | 11 | 59 | Primary solid Tumor |
| TCGA-B5-A0K0-01A-11R-A16W-07 | 16 | 11 | 59 | Primary solid Tumor |
| TCGA-B5-A0K1-01A-11R-A16W-07 | 16 | 11 | 59 | Primary solid Tumor |
| TCGA-B5-A0K2-01A-12R-A104-07 | 16 | 11 | 73 | Primary solid Tumor |
| TCGA-B5-A0K3-01A-11R-A040-07 | 16 | 11 | 59 | Primary solid Tumor |
| TCGA-B5-A0K4-01A-11R-A040-07 | 16 | 11 | 59 | Primary solid Tumor |
| TCGA-B5-A0K6-01A-11R-A040-07 | 16 | 11 | 59 | Primary solid Tumor |
| TCGA-B5-A0K7-01A-11R-A104-07 | 16 | 11 | 73 | Primary solid Tumor |
| TCGA-B5-A0K8-01A-11R-A14M-07 | 16 | 11 | 125 | Primary solid Tumor |
| TCGA-B5-A0K9-01A-21R-A104-07 | 16 | NA | 73 | Primary solid Tumor |
| TCGA-B5-A0KB-01B-11R-A14D-07 | 16 | 11 | 121 | Primary solid Tumor |
| TCGA-B5-A11E-01A-11R-A10J-07 | 16 | 11 | 81 | Primary solid Tumor |
| TCGA-B5-A11F-01A-11R-A10J-07 | 16 | 11 | 81 | Primary solid Tumor |
| TCGA-B5-A11G-01A-13R-A118-07 | 16 | 11 | 92 | Primary solid Tumor |
| TCGA-B5-A11H-01A-11R-A118-07 | 16 | 11 | 92 | Primary solid Tumor |
| TCGA-B5-A11I-01A-11R-A10J-07 | 20 | 11 | 81 | Primary solid Tumor |
| TCGA-B5-A11J-01A-11R-A118-07 | 16 | 11 | 92 | Primary solid Tumor |
| TCGA-B5-A11L-01B-21R-A13S-07 | 16 | 11 | 110 | Primary solid Tumor |
| TCGA-B5-A11M-01A-11R-A118-07 | 16 | 11 | 92 | Primary solid Tumor |
| TCGA-B5-A11N-01A-11R-A118-07 | 16 | 11 | 92 | Primary solid Tumor |
| TCGA-B5-A11O-01A-11R-A118-07 | 16 | 11 | 92 | Primary solid Tumor |
| TCGA-B5-A11P-01B-11R-A14D-07 | 16 | 11 | 121 | Primary solid Tumor |
| TCGA-B5-A11Q-01A-11R-A118-07 | 16 | 11 | 92 | Primary solid Tumor |
| TCGA-B5-A11R-01A-11R-A118-07 | 16 | NA | 92 | Primary solid Tumor |
| TCGA-B5-A11S-01A-11R-A118-07 | 16 | 11 | 92 | Primary solid Tumor |
| TCGA-B5-A11U-01A-11R-A118-07 | 16 | 11 | 92 | Primary solid Tumor |
| TCGA-B5-A11V-01A-11R-A10J-07 | 16 | 11 | 81 | Primary solid Tumor |
| TCGA-B5-A11W-01A-12R-A118-07 | 16 | 11 | 92 | Primary solid Tumor |
| TCGA-B5-A11X-01A-11R-A10J-07 | 4 | 3 | 81 | Primary solid Tumor |
| TCGA-B5-A11Y-01A-21R-A10J-07 | 16 | 11 | 81 | Primary solid Tumor |
| TCGA-B5-A11Z-01A-11R-A10J-07 | 16 | 11 | 81 | Primary solid Tumor |
| TCGA-B5-A121-01A-31R-A118-07 | 16 | 11 | 92 | Primary solid Tumor |
| TCGA-B5-A1MR-01A-31R-A14D-07 | 16 | 13 | 121 | Primary solid Tumor |
| TCGA-B5-A1MS-01B-11R-A22K-07 | 16 | NA | 228 | Primary solid Tumor |
| TCGA-B5-A1MU-01A-11R-A13S-07 | 16 | 11 | 110 | Primary solid Tumor |
| TCGA-B5-A1MV-01A-31R-A14D-07 | 16 | 11 | 121 | Primary solid Tumor |
| TCGA-B5-A1MW-01A-11R-A32Y-07 | 16 | NA | 156 | Primary solid Tumor |
| TCGA-B5-A1MX-01A-11R-A144-07 | 16 | 11 | 118 | Primary solid Tumor |
| TCGA-B5-A1MY-01A-11R-A144-07 | 16 | 11 | 118 | Primary solid Tumor |
| TCGA-B5-A1MZ-01A-11R-A144-07 | 16 | 11 | 118 | Primary solid Tumor |
| TCGA-B5-A1N2-01A-21R-A144-07 | 16 | 11 | 118 | Primary solid Tumor |
| TCGA-B5-A3F9-01A-21R-A22K-07 | 16 | NA | 228 | Primary solid Tumor |
| TCGA-B5-A3FA-01A-11R-A19W-07 | 16 | NA | 186 | Primary solid Tumor |
| TCGA-B5-A3FB-01A-11R-A19W-07 | 16 | NA | 186 | Primary solid Tumor |
| TCGA-B5-A3FC-01A-11R-A22K-07 | 16 | NA | 228 | Primary solid Tumor |
| TCGA-B5-A3FD-01A-11R-A19W-07 | 16 | NA | 186 | Primary solid Tumor |
| TCGA-B5-A3FH-01A-11R-A19W-07 | 16 | NA | 186 | Primary solid Tumor |
| TCGA-B5-A3S1-01A-11R-A22K-07 | 16 | NA | 228 | Primary solid Tumor |
| TCGA-B5-A5OC-01A-21R-A27V-07 | 16 | NA | 289 | Primary solid Tumor |
| TCGA-B5-A5OD-01A-11R-A31O-07 | 16 | NA | 324 | Primary solid Tumor |
| TCGA-B5-A5OE-01A-11R-A31O-07 | 16 | NA | 324 | Primary solid Tumor |
| TCGA-B6-A0I1-01A-11R-A21T-07 | 10 | 8 | 216 | Primary solid Tumor |
| TCGA-B6-A0I2-01A-11R-A034-07 | 10 | 8 | 56 | Primary solid Tumor |
| TCGA-B6-A0I5-01A-11R-A034-07 | 9 | 7 | 56 | Primary solid Tumor |
| TCGA-B6-A0I6-01A-11R-A034-07 | 10 | 8 | 56 | Primary solid Tumor |
| TCGA-B6-A0I8-01A-11R-A034-07 | 9 | 7 | 56 | Primary solid Tumor |
| TCGA-B6-A0I9-01A-11R-A034-07 | 9 | 7 | 56 | Primary solid Tumor |
| TCGA-B6-A0IA-01A-11R-A034-07 | 9 | 7 | 56 | Primary solid Tumor |
| TCGA-B6-A0IB-01A-11R-A034-07 | 9 | 7 | 56 | Primary solid Tumor |
| TCGA-B6-A0IC-01A-11R-A034-07 | 9 | 7 | 56 | Primary solid Tumor |
| TCGA-B6-A0IE-01A-11R-A034-07 | 9 | 7 | 56 | Primary solid Tumor |
| TCGA-B6-A0IG-01A-11R-A034-07 | 9 | 7 | 56 | Primary solid Tumor |
| TCGA-B6-A0IH-01A-11R-A115-07 | 9 | 7 | 85 | Primary solid Tumor |
| TCGA-B6-A0IJ-01A-11R-A034-07 | 10 | 8 | 56 | Primary solid Tumor |
| TCGA-B6-A0IK-01A-12R-A056-07 | 9 | 7 | 61 | Primary solid Tumor |
| TCGA-B6-A0IM-01A-11R-A034-07 | 9 | 7 | 56 | Primary solid Tumor |
| TCGA-B6-A0IN-01A-11R-A034-07 | 9 | 7 | 56 | Primary solid Tumor |
| TCGA-B6-A0IO-01A-11R-A034-07 | 9 | 7 | 56 | Primary solid Tumor |
| TCGA-B6-A0IP-01A-11R-A034-07 | 9 | 7 | 56 | Primary solid Tumor |
| TCGA-B6-A0IQ-01A-11R-A034-07 | 10 | 8 | 56 | Primary solid Tumor |
| TCGA-B6-A0RE-01A-11R-A056-07 | 10 | 8 | 61 | Primary solid Tumor |
| TCGA-B6-A0RG-01A-11R-A056-07 | 9 | 7 | 61 | Primary solid Tumor |
| TCGA-B6-A0RH-01A-21R-A115-07 | 9 | 7 | 85 | Primary solid Tumor |
| TCGA-B6-A0RI-01A-11R-A056-07 | 9 | 7 | 61 | Primary solid Tumor |
| TCGA-B6-A0RL-01A-11R-A084-07 | 9 | 7 | 72 | Primary solid Tumor |
| TCGA-B6-A0RM-01A-11R-A084-07 | 9 | 7 | 72 | Primary solid Tumor |
| TCGA-B6-A0RN-01A-12R-A084-07 | 9 | 7 | 72 | Primary solid Tumor |
| TCGA-B6-A0RO-01A-22R-A084-07 | 9 | 7 | 72 | Primary solid Tumor |
| TCGA-B6-A0RP-01A-21R-A084-07 | 9 | 7 | 72 | Primary solid Tumor |
| TCGA-B6-A0RQ-01A-11R-A115-07 | 9 | 7 | 85 | Primary solid Tumor |
| TCGA-B6-A0RS-01A-11R-A084-07 | 9 | 7 | 72 | Primary solid Tumor |
| TCGA-B6-A0RT-01A-21R-A084-07 | 10 | 8 | 72 | Primary solid Tumor |
| TCGA-B6-A0RU-01A-11R-A084-07 | 10 | 8 | 72 | Primary solid Tumor |
| TCGA-B6-A0RV-01A-11R-A084-07 | 9 | 7 | 72 | Primary solid Tumor |
| TCGA-B6-A0WS-01A-11R-A115-07 | 9 | 7 | 85 | Primary solid Tumor |
| TCGA-B6-A0WT-01A-11R-A109-07 | 9 | 7 | 74 | Primary solid Tumor |
| TCGA-B6-A0WV-01A-11R-A109-07 | 9 | 7 | 74 | Primary solid Tumor |
| TCGA-B6-A0WW-01A-11R-A109-07 | 9 | 7 | 74 | Primary solid Tumor |
| TCGA-B6-A0WX-01A-11R-A109-07 | 10 | 8 | 74 | Primary solid Tumor |
| TCGA-B6-A0WY-01A-11R-A109-07 | 9 | 7 | 74 | Primary solid Tumor |
| TCGA-B6-A0WZ-01A-11R-A109-07 | 9 | 7 | 74 | Primary solid Tumor |
| TCGA-B6-A0X0-01A-21R-A115-07 | 9 | 7 | 85 | Primary solid Tumor |
| TCGA-B6-A0X1-01A-11R-A109-07 | 10 | 8 | 74 | Primary solid Tumor |
| TCGA-B6-A0X4-01A-11R-A109-07 | 9 | 7 | 74 | Primary solid Tumor |
| TCGA-B6-A0X5-01A-21R-A109-07 | 9 | 7 | 74 | Primary solid Tumor |
| TCGA-B6-A0X7-01A-11R-A10J-07 | 9 | 7 | 80 | Primary solid Tumor |
| TCGA-B6-A1KC-01B-11R-A157-07 | 9 | 7 | 109 | Primary solid Tumor |
| TCGA-B6-A1KF-01A-11R-A13Q-07 | 10 | 8 | 109 | Primary solid Tumor |
| TCGA-B6-A1KI-01A-11R-A14M-07 | 9 | 7 | 124 | Primary solid Tumor |
| TCGA-B6-A1KN-01A-11R-A13Q-07 | 9 | 7 | 109 | Primary solid Tumor |
| TCGA-B6-A2IU-01A-32R-A18M-07 | 9 | 7 | 177 | Primary solid Tumor |
| TCGA-B6-A3ZX-01A-11R-A239-07 | 10 | NA | 239 | Primary solid Tumor |
| TCGA-B6-A400-01A-11R-A239-07 | 10 | NA | 239 | Primary solid Tumor |
| TCGA-B6-A401-01A-11R-A239-07 | 9 | NA | 239 | Primary solid Tumor |
| TCGA-B6-A402-01A-11R-A239-07 | 10 | NA | 239 | Primary solid Tumor |
| TCGA-B6-A408-01A-12R-A24H-07 | 9 | NA | 255 | Primary solid Tumor |
| TCGA-B6-A409-01A-11R-A24H-07 | 10 | NA | 255 | Primary solid Tumor |
| TCGA-B6-A40B-01A-11R-A239-07 | 9 | NA | 239 | Primary solid Tumor |
| TCGA-B6-A40C-01A-11R-A239-07 | 9 | NA | 239 | Primary solid Tumor |
| TCGA-B8-4143-01A-01R-1188-07 | 14 | 9 | 50 | Primary solid Tumor |
| TCGA-B8-4146-01B-11R-1672-07 | 14 | 9 | 105 | Primary solid Tumor |
| TCGA-B8-4148-01A-02R-1325-07 | 14 | 9 | 50 | Primary solid Tumor |
| TCGA-B8-4151-01A-01R-1188-07 | 14 | 9 | 50 | Primary solid Tumor |
| TCGA-B8-4153-01B-11R-1672-07 | 14 | 9 | 105 | Primary solid Tumor |
| TCGA-B8-4154-01A-01R-1188-07 | 14 | 9 | 50 | Primary solid Tumor |
| TCGA-B8-4619-01A-02R-1325-07 | 14 | 9 | 50 | Primary solid Tumor |
| TCGA-B8-4620-01A-02R-1325-07 | 14 | 9 | 50 | Primary solid Tumor |
| TCGA-B8-4621-01A-01R-1503-07 | 14 | 9 | 82 | Primary solid Tumor |
| TCGA-B8-4622-01A-02R-1277-07 | 14 | 9 | 63 | Primary solid Tumor |
| TCGA-B8-5158-01A-01R-1420-07 | 14 | 9 | 68 | Primary solid Tumor |
| TCGA-B8-5159-01A-01R-1420-07 | 14 | 9 | 68 | Primary solid Tumor |
| TCGA-B8-5162-01A-01R-1420-07 | 14 | 9 | 68 | Primary solid Tumor |
| TCGA-B8-5163-01A-01R-1420-07 | 14 | 9 | 68 | Primary solid Tumor |
| TCGA-B8-5164-01A-01R-1420-07 | 14 | 9 | 68 | Primary solid Tumor |
| TCGA-B8-5165-01A-01R-1420-07 | 14 | 9 | 68 | Primary solid Tumor |
| TCGA-B8-5545-01A-01R-1672-07 | 14 | 9 | 105 | Primary solid Tumor |
| TCGA-B8-5546-01A-01R-1541-07 | 14 | 9 | 90 | Primary solid Tumor |
| TCGA-B8-5549-01A-01R-1541-07 | 14 | 9 | 90 | Primary solid Tumor |
| TCGA-B8-5550-01A-01R-1541-07 | 14 | 9 | 90 | Primary solid Tumor |
| TCGA-B8-5551-01A-01R-1541-07 | 14 | 9 | 90 | Primary solid Tumor |
| TCGA-B8-5552-01B-11R-1672-07 | 14 | 9 | 105 | Primary solid Tumor |
| TCGA-B8-5553-01A-01R-1541-07 | 14 | 9 | 90 | Primary solid Tumor |
| TCGA-B8-A54D-01A-21R-A266-07 | 14 | NA | 274 | Primary solid Tumor |
| TCGA-B8-A54E-01A-11R-A266-07 | 14 | NA | 274 | Primary solid Tumor |
| TCGA-B8-A54F-01A-11R-A266-07 | 14 | NA | 274 | Primary solid Tumor |
| TCGA-B8-A54G-01A-11R-A266-07 | 14 | NA | 274 | Primary solid Tumor |
| TCGA-B8-A54H-01A-11R-A33J-07 | 14 | NA | 340 | Primary solid Tumor |
| TCGA-B8-A54I-01A-21R-A33J-07 | 14 | NA | 340 | Primary solid Tumor |
| TCGA-B8-A54J-01A-11R-A33J-07 | 14 | NA | 340 | Primary solid Tumor |
| TCGA-B8-A54K-01A-11R-A33J-07 | 14 | NA | 340 | Primary solid Tumor |
| TCGA-B8-A7U6-01A-12R-A37O-07 | 14 | NA | 387 | Primary solid Tumor |
| TCGA-B8-A8YJ-01A-13R-A39I-07 | 14 | NA | 404 | Primary solid Tumor |
| TCGA-BA-4074-01A-01R-1436-07 | 2 | 4 | 54 | Primary solid Tumor |
| TCGA-BA-4075-01A-01R-1436-07 | 2 | 4 | 54 | Primary solid Tumor |
| TCGA-BA-4076-01A-01R-1436-07 | 2 | 4 | 54 | Primary solid Tumor |
| TCGA-BA-4077-01B-01R-1436-07 | 2 | 4 | 54 | Primary solid Tumor |
| TCGA-BA-4078-01A-01R-1436-07 | 2 | 4 | 54 | Primary solid Tumor |
| TCGA-BA-5149-01A-01R-1514-07 | 2 | 4 | 83 | Primary solid Tumor |
| TCGA-BA-5151-01A-01R-1436-07 | 2 | 4 | 54 | Primary solid Tumor |
| TCGA-BA-5152-01A-02R-1873-07 | 2 | 4 | 145 | Primary solid Tumor |
| TCGA-BA-5153-01A-01R-1436-07 | 2 | 4 | 54 | Primary solid Tumor |
| TCGA-BA-5555-01A-01R-1514-07 | 2 | 4 | 83 | Primary solid Tumor |
| TCGA-BA-5556-01A-01R-1514-07 | 2 | 4 | 83 | Primary solid Tumor |
| TCGA-BA-5557-01A-01R-1514-07 | 2 | 4 | 83 | Primary solid Tumor |
| TCGA-BA-5558-01A-01R-1514-07 | 2 | 4 | 83 | Primary solid Tumor |
| TCGA-BA-5559-01A-01R-1514-07 | 2 | 4 | 83 | Primary solid Tumor |
| TCGA-BA-6868-01B-12R-1915-07 | 2 | 4 | 151 | Primary solid Tumor |
| TCGA-BA-6869-01A-11R-1873-07 | 2 | 4 | 145 | Primary solid Tumor |
| TCGA-BA-6870-01A-11R-1873-07 | 2 | 4 | 145 | Primary solid Tumor |
| TCGA-BA-6871-01A-11R-1873-07 | 2 | 4 | 145 | Primary solid Tumor |
| TCGA-BA-6872-01A-11R-1873-07 | 2 | 4 | 145 | Primary solid Tumor |
| TCGA-BA-6873-01A-11R-1873-07 | 2 | 4 | 145 | Primary solid Tumor |
| TCGA-BA-7269-01A-11R-2016-07 | 2 | 4 | 164 | Primary solid Tumor |
| TCGA-BA-A4IF-01A-11R-A266-07 | 2 | NA | 273 | Primary solid Tumor |
| TCGA-BA-A4IG-01A-11R-A266-07 | 2 | NA | 273 | Primary solid Tumor |
| TCGA-BA-A4IH-01A-11R-A266-07 | 2 | NA | 273 | Primary solid Tumor |
| TCGA-BA-A4II-01A-11R-A266-07 | 2 | NA | 273 | Primary solid Tumor |
| TCGA-BA-A6D8-01A-31R-A31N-07 | 2 | NA | 318 | Primary solid Tumor |
| TCGA-BA-A6DA-01A-31R-A31N-07 | 2 | NA | 318 | Primary solid Tumor |
| TCGA-BA-A6DB-01A-11R-A30B-07 | 2 | NA | 311 | Primary solid Tumor |
| TCGA-BA-A6DD-01A-12R-A31N-07 | 2 | NA | 318 | Primary solid Tumor |
| TCGA-BA-A6DE-01A-22R-A31N-07 | 2 | NA | 318 | Primary solid Tumor |
| TCGA-BA-A6DG-01A-21R-A30B-07 | 2 | NA | 311 | Primary solid Tumor |
| TCGA-BA-A6DI-01A-11R-A30B-07 | 2 | NA | 311 | Primary solid Tumor |
| TCGA-BA-A6DJ-01A-11R-A30B-07 | 2 | NA | 311 | Primary solid Tumor |
| TCGA-BA-A6DL-01A-21R-A30B-07 | 2 | NA | 311 | Primary solid Tumor |
| TCGA-BA-A8YP-01A-11R-A39I-07 | 2 | NA | 403 | Primary solid Tumor |
| TCGA-BB-4217-01A-11R-2081-07 | 2 | 4 | 188 | Primary solid Tumor |
| TCGA-BB-4223-01A-01R-1436-07 | 2 | 4 | 54 | Primary solid Tumor |
| TCGA-BB-4224-01A-01R-1436-07 | 2 | 4 | 54 | Primary solid Tumor |
| TCGA-BB-4225-01A-01R-1436-07 | 2 | 4 | 54 | Primary solid Tumor |
| TCGA-BB-4227-01A-01R-1873-07 | 2 | NA | 145 | Primary solid Tumor |
| TCGA-BB-4228-01A-01R-1436-07 | 2 | 4 | 54 | Primary solid Tumor |
| TCGA-BB-7861-01A-11R-2232-07 | 2 | 4 | 215 | Primary solid Tumor |
| TCGA-BB-7862-01A-21R-2232-07 | 2 | 4 | 215 | Primary solid Tumor |
| TCGA-BB-7863-01A-11R-2232-07 | 2 | 4 | 215 | Primary solid Tumor |
| TCGA-BB-7864-01A-11R-2232-07 | 2 | 4 | 215 | Primary solid Tumor |
| TCGA-BB-7866-01A-11R-2232-07 | 2 | 4 | 215 | Primary solid Tumor |
| TCGA-BB-7870-01A-11R-2232-07 | 2 | 4 | 215 | Primary solid Tumor |
| TCGA-BB-7871-01A-11R-2232-07 | 2 | 4 | 215 | Primary solid Tumor |
| TCGA-BB-7872-01A-11R-2232-07 | 2 | 4 | 215 | Primary solid Tumor |
| TCGA-BB-8596-01A-11R-2403-07 | 2 | NA | 241 | Primary solid Tumor |
| TCGA-BB-8601-01A-11R-2403-07 | 2 | NA | 241 | Primary solid Tumor |
| TCGA-BB-A5HU-01A-11R-A28V-07 | 2 | NA | 301 | Primary solid Tumor |
| TCGA-BB-A5HY-01A-11R-A28V-07 | 2 | NA | 301 | Primary solid Tumor |
| TCGA-BB-A5HZ-01A-21R-A28V-07 | 2 | NA | 301 | Primary solid Tumor |
| TCGA-BB-A6UM-01A-12R-A34R-07 | 2 | NA | 355 | Primary solid Tumor |
| TCGA-BB-A6UO-01A-12R-A34R-07 | 2 | NA | 355 | Primary solid Tumor |
| TCGA-BG-A0LW-01A-11R-A16W-07 | 16 | 11 | 59 | Primary solid Tumor |
| TCGA-BG-A0LX-01A-11R-A16W-07 | 16 | 11 | 59 | Primary solid Tumor |
| TCGA-BG-A0M0-01A-11R-A104-07 | 16 | 11 | 73 | Primary solid Tumor |
| TCGA-BG-A0M2-01A-11R-A104-07 | 16 | 11 | 73 | Primary solid Tumor |
| TCGA-BG-A0M3-01A-11R-A104-07 | 16 | 11 | 73 | Primary solid Tumor |
| TCGA-BG-A0M4-01A-11R-A104-07 | 16 | 11 | 73 | Primary solid Tumor |
| TCGA-BG-A0M6-01A-31R-A104-07 | 16 | 11 | 73 | Primary solid Tumor |
| TCGA-BG-A0M7-01A-11R-A040-07 | 16 | 11 | 59 | Primary solid Tumor |
| TCGA-BG-A0M8-01A-12R-A104-07 | 16 | 11 | 73 | Primary solid Tumor |
| TCGA-BG-A0M9-01A-21R-A104-07 | 16 | 11 | 73 | Primary solid Tumor |
| TCGA-BG-A0MA-01A-11R-A17B-07 | 16 | 11 | 156 | Primary solid Tumor |
| TCGA-BG-A0MC-01A-21R-A040-07 | 16 | 11 | 59 | Primary solid Tumor |
| TCGA-BG-A0MG-01A-21R-A104-07 | 16 | 11 | 73 | Primary solid Tumor |
| TCGA-BG-A0MH-01A-11R-A118-07 | 16 | 11 | 92 | Primary solid Tumor |
| TCGA-BG-A0MI-01A-11R-A040-07 | 16 | 11 | 59 | Primary solid Tumor |
| TCGA-BG-A0MK-01A-51R-A19W-07 | 16 | NA | 186 | Primary solid Tumor |
| TCGA-BG-A0MO-01A-11R-A040-07 | 16 | 11 | 59 | Primary solid Tumor |
| TCGA-BG-A0MQ-01A-11R-A104-07 | 16 | 11 | 73 | Primary solid Tumor |
| TCGA-BG-A0MS-01A-11R-A104-07 | 16 | 11 | 73 | Primary solid Tumor |
| TCGA-BG-A0MT-01A-11R-A104-07 | 16 | 11 | 73 | Primary solid Tumor |
| TCGA-BG-A0MU-01A-11R-A104-07 | 16 | 11 | 73 | Primary solid Tumor |
| TCGA-BG-A0RY-01A-11R-A104-07 | 16 | 11 | 73 | Primary solid Tumor |
| TCGA-BG-A0VT-01A-11R-A10J-07 | 16 | 11 | 81 | Primary solid Tumor |
| TCGA-BG-A0VV-01A-21R-A118-07 | 16 | 11 | 92 | Primary solid Tumor |
| TCGA-BG-A0VW-01A-11R-A118-07 | 16 | 11 | 92 | Primary solid Tumor |
| TCGA-BG-A0VX-01A-11R-A118-07 | 16 | 11 | 92 | Primary solid Tumor |
| TCGA-BG-A0VZ-01A-11R-A109-07 | 16 | 11 | 75 | Primary solid Tumor |
| TCGA-BG-A0W1-01A-12R-A109-07 | 16 | 11 | 75 | Primary solid Tumor |
| TCGA-BG-A0W2-01A-11R-A109-07 | 16 | 11 | 75 | Primary solid Tumor |
| TCGA-BG-A0YU-01A-21R-A10J-07 | 16 | 11 | 81 | Primary solid Tumor |
| TCGA-BG-A0YV-01A-11R-A10J-07 | 16 | 11 | 81 | Primary solid Tumor |
| TCGA-BG-A186-01A-11R-A12I-07 | 16 | 11 | 94 | Primary solid Tumor |
| TCGA-BG-A187-01A-11R-A12I-07 | 16 | 11 | 94 | Primary solid Tumor |
| TCGA-BG-A18A-01A-21R-A12I-07 | 16 | 11 | 94 | Primary solid Tumor |
| TCGA-BG-A18B-01A-11R-A12I-07 | 16 | 11 | 94 | Primary solid Tumor |
| TCGA-BG-A18C-01A-11R-A12I-07 | 16 | 11 | 94 | Primary solid Tumor |
| TCGA-BG-A220-01A-11R-A157-07 | 16 | 11 | 137 | Primary solid Tumor |
| TCGA-BG-A221-01A-21R-A157-07 | 16 | 11 | 137 | Primary solid Tumor |
| TCGA-BG-A222-01A-11R-A157-07 | 16 | 11 | 137 | Primary solid Tumor |
| TCGA-BG-A2AD-01A-21R-A16F-07 | 16 | 11 | 143 | Primary solid Tumor |
| TCGA-BG-A2AE-01A-11R-A16F-07 | 16 | 11 | 143 | Primary solid Tumor |
| TCGA-BG-A2L7-01A-11R-A18M-07 | 16 | 11 | 178 | Primary solid Tumor |
| TCGA-BG-A3EW-01A-11R-A22K-07 | 16 | NA | 228 | Primary solid Tumor |
| TCGA-BG-A3PP-01A-11R-A22K-07 | 16 | NA | 228 | Primary solid Tumor |
| TCGA-BH-A0AU-01A-11R-A12P-07 | 9 | 7 | 96 | Primary solid Tumor |
| TCGA-BH-A0AV-01A-31R-A115-07 | 10 | 8 | 85 | Primary solid Tumor |
| TCGA-BH-A0AW-01A-11R-A056-07 | 9 | 7 | 61 | Primary solid Tumor |
| TCGA-BH-A0AY-01A-21R-A00Z-07 | 9 | 7 | 47 | Primary solid Tumor |
| TCGA-BH-A0AZ-01A-21R-A12P-07 | 9 | 7 | 96 | Primary solid Tumor |
| TCGA-BH-A0B0-01A-21R-A115-07 | 9 | 7 | 85 | Primary solid Tumor |
| TCGA-BH-A0B1-01A-12R-A056-07 | 9 | 7 | 61 | Primary solid Tumor |
| TCGA-BH-A0B2-01A-11R-A10J-07 | 9 | NA | 80 | Primary solid Tumor |
| TCGA-BH-A0B3-01A-11R-A056-07 | 10 | 8 | 61 | Primary solid Tumor |
| TCGA-BH-A0B4-01A-11R-A00Z-07 | 9 | 7 | 47 | Primary solid Tumor |
| TCGA-BH-A0B5-01A-11R-A12P-07 | 9 | 7 | 96 | Primary solid Tumor |
| TCGA-BH-A0B6-01A-11R-A19W-07 | 9 | NA | 185 | Primary solid Tumor |
| TCGA-BH-A0B7-01A-12R-A115-07 | 9 | 7 | 85 | Primary solid Tumor |
| TCGA-BH-A0B8-01A-21R-A056-07 | 9 | 7 | 61 | Primary solid Tumor |
| TCGA-BH-A0B9-01A-11R-A056-07 | 10 | NA | 61 | Primary solid Tumor |
| TCGA-BH-A0BA-01A-11R-A056-07 | 9 | 7 | 61 | Primary solid Tumor |
| TCGA-BH-A0BC-01A-22R-A084-07 | 9 | 7 | 72 | Primary solid Tumor |
| TCGA-BH-A0BD-01A-11R-A034-07 | 9 | 7 | 56 | Primary solid Tumor |
| TCGA-BH-A0BF-01A-21R-A12P-07 | 9 | 7 | 96 | Primary solid Tumor |
| TCGA-BH-A0BG-01A-11R-A115-07 | 10 | 8 | 85 | Primary solid Tumor |
| TCGA-BH-A0BJ-01A-11R-A056-07 | 9 | 7 | 61 | Primary solid Tumor |
| TCGA-BH-A0BL-01A-11R-A115-07 | 10 | 8 | 85 | Primary solid Tumor |
| TCGA-BH-A0BM-01A-11R-A056-07 | 9 | 7 | 61 | Primary solid Tumor |
| TCGA-BH-A0BO-01A-23R-A12D-07 | 9 | 7 | 93 | Primary solid Tumor |
| TCGA-BH-A0BP-01A-11R-A115-07 | 9 | 7 | 85 | Primary solid Tumor |
| TCGA-BH-A0BQ-01A-21R-A115-07 | 9 | 7 | 85 | Primary solid Tumor |
| TCGA-BH-A0BR-01A-21R-A115-07 | 9 | 7 | 85 | Primary solid Tumor |
| TCGA-BH-A0BS-01A-11R-A12P-07 | 9 | 7 | 96 | Primary solid Tumor |
| TCGA-BH-A0BT-01A-11R-A12P-07 | 9 | 7 | 96 | Primary solid Tumor |
| TCGA-BH-A0BV-01A-11R-A00Z-07 | 9 | 7 | 47 | Primary solid Tumor |
| TCGA-BH-A0BW-01A-11R-A115-07 | 10 | 8 | 85 | Primary solid Tumor |
| TCGA-BH-A0BZ-01A-31R-A12P-07 | 9 | 7 | 96 | Primary solid Tumor |
| TCGA-BH-A0C0-01A-21R-A056-07 | 9 | 7 | 61 | Primary solid Tumor |
| TCGA-BH-A0C1-01B-11R-A12D-07 | 9 | 7 | 93 | Primary solid Tumor |
| TCGA-BH-A0C3-01A-21R-A12P-07 | 9 | 7 | 96 | Primary solid Tumor |
| TCGA-BH-A0C7-01B-11R-A115-07 | 9 | 7 | 85 | Primary solid Tumor |
| TCGA-BH-A0DD-01A-31R-A12P-07 | 9 | 7 | 96 | Primary solid Tumor |
| TCGA-BH-A0DE-01A-11R-A115-07 | 9 | 7 | 85 | Primary solid Tumor |
| TCGA-BH-A0DG-01A-21R-A12P-07 | 9 | 7 | 96 | Primary solid Tumor |
| TCGA-BH-A0DH-01A-11R-A084-07 | 9 | 7 | 72 | Primary solid Tumor |
| TCGA-BH-A0DI-01A-21R-A12P-07 | 9 | 7 | 96 | Primary solid Tumor |
| TCGA-BH-A0DK-01A-21R-A056-07 | 9 | 7 | 61 | Primary solid Tumor |
| TCGA-BH-A0DL-01A-11R-A115-07 | 10 | 8 | 85 | Primary solid Tumor |
| TCGA-BH-A0DO-01B-11R-A12D-07 | 9 | 7 | 93 | Primary solid Tumor |
| TCGA-BH-A0DP-01A-21R-A056-07 | 9 | 7 | 61 | Primary solid Tumor |
| TCGA-BH-A0DQ-01A-11R-A084-07 | 9 | 7 | 72 | Primary solid Tumor |
| TCGA-BH-A0DS-01A-11R-A056-07 | 9 | 7 | 61 | Primary solid Tumor |
| TCGA-BH-A0DT-01A-21R-A12D-07 | 9 | 7 | 93 | Primary solid Tumor |
| TCGA-BH-A0DV-01A-21R-A12P-07 | 9 | 7 | 96 | Primary solid Tumor |
| TCGA-BH-A0DX-01A-11R-A115-07 | 9 | 7 | 85 | Primary solid Tumor |
| TCGA-BH-A0DZ-01A-11R-A00Z-07 | 9 | 7 | 47 | Primary solid Tumor |
| TCGA-BH-A0E0-01A-11R-A056-07 | 10 | 8 | 61 | Primary solid Tumor |
| TCGA-BH-A0E1-01A-11R-A056-07 | 9 | 7 | 61 | Primary solid Tumor |
| TCGA-BH-A0E2-01A-11R-A056-07 | 9 | 7 | 61 | Primary solid Tumor |
| TCGA-BH-A0E6-01A-11R-A034-07 | 10 | 8 | 56 | Primary solid Tumor |
| TCGA-BH-A0E7-01A-11R-A034-07 | 9 | 7 | 56 | Primary solid Tumor |
| TCGA-BH-A0E9-01B-11R-A115-07 | 9 | 7 | 85 | Primary solid Tumor |
| TCGA-BH-A0EA-01A-11R-A115-07 | 9 | 7 | 85 | Primary solid Tumor |
| TCGA-BH-A0EB-01A-11R-A034-07 | 9 | 7 | 56 | Primary solid Tumor |
| TCGA-BH-A0EE-01A-11R-A034-07 | 12 | 7 | 56 | Primary solid Tumor |
| TCGA-BH-A0EI-01A-11R-A115-07 | 9 | 7 | 85 | Primary solid Tumor |
| TCGA-BH-A0GY-01A-11R-A056-07 | 9 | 7 | 61 | Primary solid Tumor |
| TCGA-BH-A0GZ-01A-11R-A056-07 | 9 | 7 | 61 | Primary solid Tumor |
| TCGA-BH-A0H0-01A-11R-A056-07 | 9 | 7 | 61 | Primary solid Tumor |
| TCGA-BH-A0H3-01A-11R-A12P-07 | 9 | 7 | 96 | Primary solid Tumor |
| TCGA-BH-A0H5-01A-21R-A115-07 | 9 | 7 | 85 | Primary solid Tumor |
| TCGA-BH-A0H6-01A-21R-A056-07 | 9 | 7 | 61 | Primary solid Tumor |
| TCGA-BH-A0H7-01A-13R-A056-07 | 9 | 7 | 61 | Primary solid Tumor |
| TCGA-BH-A0H9-01A-11R-A056-07 | 9 | 7 | 61 | Primary solid Tumor |
| TCGA-BH-A0HA-01A-11R-A12P-07 | 9 | 7 | 96 | Primary solid Tumor |
| TCGA-BH-A0HB-01A-11R-A056-07 | 9 | 7 | 61 | Primary solid Tumor |
| TCGA-BH-A0HF-01A-11R-A056-07 | 9 | 7 | 61 | Primary solid Tumor |
| TCGA-BH-A0HI-01A-11R-A084-07 | 9 | 7 | 72 | Primary solid Tumor |
| TCGA-BH-A0HK-01A-11R-A056-07 | 9 | 7 | 61 | Primary solid Tumor |
| TCGA-BH-A0HL-01A-11R-A10U-07 | 9 | NA | 56 | Primary solid Tumor |
| TCGA-BH-A0HN-01A-11R-A10U-07 | 9 | NA | 72 | Primary solid Tumor |
| TCGA-BH-A0HO-01A-11R-A034-07 | 9 | 7 | 56 | Primary solid Tumor |
| TCGA-BH-A0HP-01A-12R-A084-07 | 9 | 7 | 72 | Primary solid Tumor |
| TCGA-BH-A0HQ-01A-11R-A034-07 | 9 | 7 | 56 | Primary solid Tumor |
| TCGA-BH-A0HU-01A-11R-A034-07 | 9 | 7 | 56 | Primary solid Tumor |
| TCGA-BH-A0HW-01A-11R-A034-07 | 9 | 7 | 56 | Primary solid Tumor |
| TCGA-BH-A0HX-01A-21R-A056-07 | 9 | 7 | 61 | Primary solid Tumor |
| TCGA-BH-A0HY-01A-11R-A056-07 | 9 | 7 | 61 | Primary solid Tumor |
| TCGA-BH-A0RX-01A-21R-A084-07 | 10 | 8 | 72 | Primary solid Tumor |
| TCGA-BH-A0W3-01A-11R-A109-07 | 9 | 7 | 74 | Primary solid Tumor |
| TCGA-BH-A0W4-01A-11R-A109-07 | 9 | 7 | 74 | Primary solid Tumor |
| TCGA-BH-A0W5-01A-11R-A109-07 | 9 | 7 | 74 | Primary solid Tumor |
| TCGA-BH-A0W7-01A-11R-A115-07 | 9 | 7 | 85 | Primary solid Tumor |
| TCGA-BH-A0WA-01A-11R-A109-07 | 10 | 8 | 74 | Primary solid Tumor |
| TCGA-BH-A18F-01A-11R-A12D-07 | 9 | 7 | 93 | Primary solid Tumor |
| TCGA-BH-A18G-01A-11R-A12D-07 | 10 | 8 | 93 | Primary solid Tumor |
| TCGA-BH-A18H-01A-11R-A12D-07 | 9 | 7 | 93 | Primary solid Tumor |
| TCGA-BH-A18I-01A-11R-A12D-07 | 9 | 7 | 93 | Primary solid Tumor |
| TCGA-BH-A18J-01A-11R-A12D-07 | 9 | 7 | 93 | Primary solid Tumor |
| TCGA-BH-A18K-01A-11R-A12D-07 | 9 | 7 | 93 | Primary solid Tumor |
| TCGA-BH-A18L-01A-32R-A12D-07 | 9 | 7 | 93 | Primary solid Tumor |
| TCGA-BH-A18M-01A-11R-A12D-07 | 9 | 7 | 93 | Primary solid Tumor |
| TCGA-BH-A18N-01A-11R-A12D-07 | 9 | 7 | 93 | Primary solid Tumor |
| TCGA-BH-A18P-01A-11R-A12D-07 | 9 | 7 | 93 | Primary solid Tumor |
| TCGA-BH-A18Q-01A-12R-A12D-07 | 10 | 8 | 93 | Primary solid Tumor |
| TCGA-BH-A18R-01A-11R-A12D-07 | 9 | 7 | 93 | Primary solid Tumor |
| TCGA-BH-A18S-01A-11R-A12D-07 | 9 | 7 | 93 | Primary solid Tumor |
| TCGA-BH-A18T-01A-11R-A12D-07 | 10 | 8 | 93 | Primary solid Tumor |
| TCGA-BH-A18U-01A-21R-A12D-07 | 9 | 7 | 93 | Primary solid Tumor |
| TCGA-BH-A18V-01A-11R-A12D-07 | 10 | 8 | 93 | Primary solid Tumor |
| TCGA-BH-A1EN-01A-11R-A13Q-07 | 9 | 7 | 109 | Primary solid Tumor |
| TCGA-BH-A1EO-01A-11R-A137-07 | 9 | 7 | 103 | Primary solid Tumor |
| TCGA-BH-A1ES-01A-11R-A137-07 | 9 | 7 | 103 | Primary solid Tumor |
| TCGA-BH-A1ET-01A-11R-A137-07 | 9 | 7 | 103 | Primary solid Tumor |
| TCGA-BH-A1EU-01A-11R-A137-07 | 9 | 7 | 103 | Primary solid Tumor |
| TCGA-BH-A1EV-01A-11R-A137-07 | 9 | 7 | 103 | Primary solid Tumor |
| TCGA-BH-A1EW-01A-11R-A137-07 | 9 | 7 | 103 | Primary solid Tumor |
| TCGA-BH-A1EX-01A-11R-A13Q-07 | 9 | 7 | 109 | Primary solid Tumor |
| TCGA-BH-A1EY-01A-11R-A13Q-07 | 9 | 7 | 109 | Primary solid Tumor |
| TCGA-BH-A1F0-01A-11R-A137-07 | 10 | 8 | 103 | Primary solid Tumor |
| TCGA-BH-A1F2-01A-31R-A13Q-07 | 9 | 7 | 109 | Primary solid Tumor |
| TCGA-BH-A1F5-01A-12R-A13Q-07 | 9 | 7 | 109 | Primary solid Tumor |
| TCGA-BH-A1F6-01A-11R-A13Q-07 | 10 | 8 | 109 | Primary solid Tumor |
| TCGA-BH-A1F8-01A-11R-A13Q-07 | 9 | 7 | 109 | Primary solid Tumor |
| TCGA-BH-A1FB-01A-11R-A13Q-07 | 9 | 7 | 109 | Primary solid Tumor |
| TCGA-BH-A1FC-01A-11R-A13Q-07 | 10 | 8 | 109 | Primary solid Tumor |
| TCGA-BH-A1FD-01A-11R-A13Q-07 | 9 | 7 | 109 | Primary solid Tumor |
| TCGA-BH-A1FE-01A-11R-A13Q-07 | 9 | 7 | 109 | Primary solid Tumor |
| TCGA-BH-A1FG-01A-11R-A13Q-07 | 9 | 7 | 109 | Primary solid Tumor |
| TCGA-BH-A1FH-01A-12R-A13Q-07 | 9 | 7 | 109 | Primary solid Tumor |
| TCGA-BH-A1FJ-01A-11R-A13Q-07 | 9 | 7 | 109 | Primary solid Tumor |
| TCGA-BH-A1FL-01A-11R-A13Q-07 | 9 | 7 | 109 | Primary solid Tumor |
| TCGA-BH-A1FM-01A-11R-A13Q-07 | 9 | 7 | 109 | Primary solid Tumor |
| TCGA-BH-A1FN-01A-11R-A13Q-07 | 9 | 7 | 109 | Primary solid Tumor |
| TCGA-BH-A1FR-01A-11R-A13Q-07 | 9 | 7 | 109 | Primary solid Tumor |
| TCGA-BH-A1FU-01A-11R-A14D-07 | 9 | 7 | 120 | Primary solid Tumor |
| TCGA-BH-A201-01A-11R-A14M-07 | 9 | 7 | 124 | Primary solid Tumor |
| TCGA-BH-A202-01A-11R-A14M-07 | 9 | 7 | 124 | Primary solid Tumor |
| TCGA-BH-A203-01A-12R-A169-07 | 9 | 7 | 142 | Primary solid Tumor |
| TCGA-BH-A204-01A-11R-A157-07 | 9 | 7 | 136 | Primary solid Tumor |
| TCGA-BH-A208-01A-11R-A157-07 | 9 | 7 | 136 | Primary solid Tumor |
| TCGA-BH-A209-01A-11R-A157-07 | 9 | 7 | 136 | Primary solid Tumor |
| TCGA-BH-A28O-01A-11R-A22K-07 | 9 | NA | 227 | Primary solid Tumor |
| TCGA-BH-A28Q-01A-11R-A16F-07 | 9 | 7 | 147 | Primary solid Tumor |
| TCGA-BH-A2L8-01A-11R-A18M-07 | 9 | 7 | 177 | Primary solid Tumor |
| TCGA-BH-A42T-01A-11R-A24H-07 | 9 | NA | 255 | Primary solid Tumor |
| TCGA-BH-A42U-01A-12R-A24H-07 | 9 | NA | 255 | Primary solid Tumor |
| TCGA-BH-A42V-01A-11R-A24H-07 | 9 | NA | 255 | Primary solid Tumor |
| TCGA-BH-A5IZ-01A-11R-A27Q-07 | 10 | NA | 288 | Primary solid Tumor |
| TCGA-BH-A5J0-01A-11R-A27Q-07 | 9 | NA | 288 | Primary solid Tumor |
| TCGA-BH-A6R8-01A-21R-A33J-07 | 9 | NA | 338 | Primary solid Tumor |
| TCGA-BH-A6R9-01A-21R-A32P-07 | 3 | NA | 334 | Primary solid Tumor |
| TCGA-BH-A8FY-01A-11R-A36F-07 | 9 | NA | 372 | Primary solid Tumor |
| TCGA-BH-A8FZ-01A-11R-A352-07 | 9 | NA | 360 | Primary solid Tumor |
| TCGA-BH-A8G0-01A-11R-A352-07 | 9 | NA | 360 | Primary solid Tumor |
| TCGA-BH-AB28-01A-31R-A41B-07 | 9 | NA | 379 | Primary solid Tumor |
| TCGA-BK-A0C9-01A-11R-A00V-07 | 16 | 11 | 49 | Primary solid Tumor |
| TCGA-BK-A0CA-01A-21R-A118-07 | 16 | 11 | 92 | Primary solid Tumor |
| TCGA-BK-A0CB-01A-32R-A104-07 | 16 | 11 | 73 | Primary solid Tumor |
| TCGA-BK-A0CC-01A-21R-A16W-07 | 16 | 11 | 49 | Primary solid Tumor |
| TCGA-BK-A139-01A-11R-A118-07 | 16 | 11 | 92 | Primary solid Tumor |
| TCGA-BK-A13B-01A-51R-A22K-07 | 16 | NA | 228 | Primary solid Tumor |
| TCGA-BK-A13C-01A-11R-A118-07 | 16 | 11 | 92 | Primary solid Tumor |
| TCGA-BK-A26L-01A-11R-A16F-07 | 16 | 11 | 143 | Primary solid Tumor |
| TCGA-BK-A4ZD-01A-11R-A27V-07 | 16 | NA | 289 | Primary solid Tumor |
| TCGA-BK-A56F-01A-32R-A27V-07 | 16 | NA | 289 | Primary solid Tumor |
| TCGA-BK-A6W3-01A-12R-A34R-07 | 16 | NA | 354 | Primary solid Tumor |
| TCGA-BK-A6W4-01A-12R-A34R-07 | 16 | NA | 354 | Primary solid Tumor |
| TCGA-BL-A0C8-01A-11R-A10U-07 | 1 | 1 | 86 | Primary solid Tumor |
| TCGA-BL-A13I-01A-11R-A13Y-07 | 3 | 2 | 113 | Primary solid Tumor |
| TCGA-BL-A13J-01A-11R-A10U-07 | 1 | 1 | 86 | Primary solid Tumor |
| TCGA-BL-A3JM-01A-12R-A21D-07 | 4 | 3 | 207 | Primary solid Tumor |
| TCGA-BL-A5ZZ-01A-31R-A30C-07 | 3 | NA | 309 | Primary solid Tumor |
| TCGA-BM-6198-01A-11R-1736-07 | 7 | NA | 122 | Primary solid Tumor |
| TCGA-BP-4158-01A-02R-1289-07 | 14 | 9 | 64 | Primary solid Tumor |
| TCGA-BP-4159-01A-02R-1289-07 | 14 | 9 | 64 | Primary solid Tumor |
| TCGA-BP-4160-01A-02R-1289-07 | 14 | 9 | 64 | Primary solid Tumor |
| TCGA-BP-4161-01A-02R-1325-07 | 14 | 9 | 50 | Primary solid Tumor |
| TCGA-BP-4162-01A-02R-1325-07 | 14 | 9 | 50 | Primary solid Tumor |
| TCGA-BP-4163-01A-02R-1325-07 | 14 | 9 | 50 | Primary solid Tumor |
| TCGA-BP-4164-01A-02R-1325-07 | 14 | 9 | 50 | Primary solid Tumor |
| TCGA-BP-4165-01A-02R-1289-07 | 14 | 9 | 64 | Primary solid Tumor |
| TCGA-BP-4166-01A-02R-1289-07 | 14 | 9 | 64 | Primary solid Tumor |
| TCGA-BP-4167-01A-02R-1325-07 | 14 | 9 | 50 | Primary solid Tumor |
| TCGA-BP-4169-01A-02R-1289-07 | 14 | 9 | 64 | Primary solid Tumor |
| TCGA-BP-4170-01A-02R-1289-07 | 14 | 9 | 64 | Primary solid Tumor |
| TCGA-BP-4173-01A-02R-1289-07 | 14 | 9 | 64 | Primary solid Tumor |
| TCGA-BP-4174-01A-02R-1289-07 | 14 | 9 | 64 | Primary solid Tumor |
| TCGA-BP-4176-01A-02R-1289-07 | 14 | 9 | 64 | Primary solid Tumor |
| TCGA-BP-4177-01A-02R-1420-07 | 14 | 9 | 68 | Primary solid Tumor |
| TCGA-BP-4325-01A-02R-1289-07 | 14 | 9 | 64 | Primary solid Tumor |
| TCGA-BP-4326-01A-01R-1289-07 | 14 | 9 | 64 | Primary solid Tumor |
| TCGA-BP-4327-01A-01R-1289-07 | 14 | 9 | 64 | Primary solid Tumor |
| TCGA-BP-4329-01A-02R-1289-07 | 14 | 9 | 64 | Primary solid Tumor |
| TCGA-BP-4330-01A-01R-1289-07 | 14 | 9 | 64 | Primary solid Tumor |
| TCGA-BP-4331-01A-01R-1289-07 | 14 | NA | 64 | Primary solid Tumor |
| TCGA-BP-4332-01A-01R-1289-07 | 14 | 9 | 64 | Primary solid Tumor |
| TCGA-BP-4334-01A-01R-1289-07 | 14 | 9 | 64 | Primary solid Tumor |
| TCGA-BP-4335-01A-01R-1289-07 | 14 | 9 | 64 | Primary solid Tumor |
| TCGA-BP-4337-01A-01R-1289-07 | 14 | 9 | 64 | Primary solid Tumor |
| TCGA-BP-4338-01A-01R-1289-07 | 14 | 9 | 64 | Primary solid Tumor |
| TCGA-BP-4340-01A-01R-1289-07 | 14 | 9 | 64 | Primary solid Tumor |
| TCGA-BP-4341-01A-01R-1289-07 | 14 | 9 | 64 | Primary solid Tumor |
| TCGA-BP-4342-01A-01R-1289-07 | 14 | 9 | 64 | Primary solid Tumor |
| TCGA-BP-4343-01A-02R-1289-07 | 14 | 9 | 64 | Primary solid Tumor |
| TCGA-BP-4344-01A-01R-1289-07 | 14 | 9 | 64 | Primary solid Tumor |
| TCGA-BP-4345-01A-01R-1289-07 | 14 | 9 | 64 | Primary solid Tumor |
| TCGA-BP-4346-01A-01R-1289-07 | 14 | 9 | 64 | Primary solid Tumor |
| TCGA-BP-4347-01A-01R-1289-07 | 14 | 9 | 64 | Primary solid Tumor |
| TCGA-BP-4349-01A-01R-1289-07 | 14 | 9 | 64 | Primary solid Tumor |
| TCGA-BP-4351-01A-01R-1289-07 | 14 | 9 | 64 | Primary solid Tumor |
| TCGA-BP-4352-01A-01R-1289-07 | 14 | 9 | 64 | Primary solid Tumor |
| TCGA-BP-4353-01A-02R-1289-07 | 14 | 9 | 64 | Primary solid Tumor |
| TCGA-BP-4354-01A-02R-1289-07 | 14 | 9 | 64 | Primary solid Tumor |
| TCGA-BP-4355-01A-01R-1289-07 | 14 | 9 | 64 | Primary solid Tumor |
| TCGA-BP-4756-01A-01R-1289-07 | 14 | 9 | 64 | Primary solid Tumor |
| TCGA-BP-4758-01A-01R-1289-07 | 14 | 9 | 64 | Primary solid Tumor |
| TCGA-BP-4759-01A-01R-1289-07 | 14 | 9 | 64 | Primary solid Tumor |
| TCGA-BP-4760-01A-02R-1420-07 | 14 | 9 | 68 | Primary solid Tumor |
| TCGA-BP-4761-01A-01R-1289-07 | 14 | 9 | 64 | Primary solid Tumor |
| TCGA-BP-4762-01A-02R-1289-07 | 14 | 9 | 64 | Primary solid Tumor |
| TCGA-BP-4763-01A-01R-1289-07 | 14 | 9 | 64 | Primary solid Tumor |
| TCGA-BP-4765-01A-01R-1289-07 | 14 | 9 | 64 | Primary solid Tumor |
| TCGA-BP-4766-01A-01R-1289-07 | 14 | 9 | 64 | Primary solid Tumor |
| TCGA-BP-4768-01A-01R-1289-07 | 14 | 9 | 64 | Primary solid Tumor |
| TCGA-BP-4769-01A-01R-1289-07 | 14 | 9 | 64 | Primary solid Tumor |
| TCGA-BP-4770-01A-01R-1503-07 | 3 | 6 | 82 | Primary solid Tumor |
| TCGA-BP-4771-01A-01R-1289-07 | 14 | 9 | 64 | Primary solid Tumor |
| TCGA-BP-4774-01A-01R-1289-07 | 14 | 9 | 64 | Primary solid Tumor |
| TCGA-BP-4775-01A-01R-1289-07 | 14 | 9 | 64 | Primary solid Tumor |
| TCGA-BP-4776-01A-01R-1289-07 | 14 | NA | 64 | Primary solid Tumor |
| TCGA-BP-4777-01A-01R-1289-07 | 14 | 9 | 64 | Primary solid Tumor |
| TCGA-BP-4781-01A-01R-1305-07 | 14 | 9 | 65 | Primary solid Tumor |
| TCGA-BP-4782-01A-02R-1420-07 | 14 | 9 | 68 | Primary solid Tumor |
| TCGA-BP-4784-01A-01R-1305-07 | 14 | 9 | 65 | Primary solid Tumor |
| TCGA-BP-4787-01A-01R-1305-07 | 14 | 9 | 65 | Primary solid Tumor |
| TCGA-BP-4789-01A-01R-1305-07 | 14 | 9 | 65 | Primary solid Tumor |
| TCGA-BP-4790-01A-01R-1305-07 | 14 | 9 | 65 | Primary solid Tumor |
| TCGA-BP-4795-01A-02R-1420-07 | 14 | 9 | 68 | Primary solid Tumor |
| TCGA-BP-4797-01A-01R-1305-07 | 14 | 9 | 65 | Primary solid Tumor |
| TCGA-BP-4798-01A-01R-1305-07 | 14 | 9 | 65 | Primary solid Tumor |
| TCGA-BP-4799-01A-01R-1305-07 | 14 | 9 | 65 | Primary solid Tumor |
| TCGA-BP-4801-01A-02R-1420-07 | 14 | 9 | 68 | Primary solid Tumor |
| TCGA-BP-4803-01A-01R-1305-07 | 14 | 9 | 65 | Primary solid Tumor |
| TCGA-BP-4804-01A-02R-1305-07 | 14 | 9 | 65 | Primary solid Tumor |
| TCGA-BP-4807-01A-01R-1305-07 | 14 | 9 | 65 | Primary solid Tumor |
| TCGA-BP-4959-01A-01R-1334-07 | 14 | 9 | 69 | Primary solid Tumor |
| TCGA-BP-4960-01A-01R-1334-07 | 14 | 9 | 69 | Primary solid Tumor |
| TCGA-BP-4961-01A-01R-1334-07 | 14 | 9 | 69 | Primary solid Tumor |
| TCGA-BP-4962-01A-01R-1334-07 | 14 | 9 | 69 | Primary solid Tumor |
| TCGA-BP-4963-01A-01R-1334-07 | 14 | 9 | 69 | Primary solid Tumor |
| TCGA-BP-4964-01A-01R-1334-07 | 14 | 9 | 69 | Primary solid Tumor |
| TCGA-BP-4965-01A-01R-1334-07 | 14 | 9 | 69 | Primary solid Tumor |
| TCGA-BP-4967-01A-01R-1334-07 | 14 | 9 | 69 | Primary solid Tumor |
| TCGA-BP-4968-01A-01R-1334-07 | 14 | 9 | 69 | Primary solid Tumor |
| TCGA-BP-4969-01A-01R-1334-07 | 14 | 9 | 69 | Primary solid Tumor |
| TCGA-BP-4970-01A-01R-1334-07 | 14 | 9 | 69 | Primary solid Tumor |
| TCGA-BP-4971-01A-01R-1334-07 | 14 | 9 | 69 | Primary solid Tumor |
| TCGA-BP-4972-01A-01R-1334-07 | 14 | 9 | 69 | Primary solid Tumor |
| TCGA-BP-4973-01A-01R-1334-07 | 14 | 9 | 69 | Primary solid Tumor |
| TCGA-BP-4974-01A-01R-1334-07 | 14 | 9 | 69 | Primary solid Tumor |
| TCGA-BP-4975-01A-01R-1334-07 | 14 | 9 | 69 | Primary solid Tumor |
| TCGA-BP-4976-01A-01R-1334-07 | 14 | 9 | 69 | Primary solid Tumor |
| TCGA-BP-4977-01A-01R-1334-07 | 14 | 9 | 69 | Primary solid Tumor |
| TCGA-BP-4981-01A-01R-1334-07 | 14 | 9 | 69 | Primary solid Tumor |
| TCGA-BP-4982-01A-01R-1334-07 | 14 | 9 | 69 | Primary solid Tumor |
| TCGA-BP-4983-01A-01R-1334-07 | 14 | 9 | 69 | Primary solid Tumor |
| TCGA-BP-4985-01A-01R-1334-07 | 14 | 9 | 69 | Primary solid Tumor |
| TCGA-BP-4986-01A-01R-1334-07 | 14 | 9 | 69 | Primary solid Tumor |
| TCGA-BP-4987-01A-01R-1334-07 | 14 | 9 | 69 | Primary solid Tumor |
| TCGA-BP-4988-01A-01R-1334-07 | 14 | 9 | 69 | Primary solid Tumor |
| TCGA-BP-4989-01A-01R-1334-07 | 14 | 9 | 69 | Primary solid Tumor |
| TCGA-BP-4991-01A-01R-1334-07 | 14 | 9 | 69 | Primary solid Tumor |
| TCGA-BP-4992-01A-01R-1334-07 | 14 | 9 | 69 | Primary solid Tumor |
| TCGA-BP-4993-01A-02R-1420-07 | 14 | 9 | 68 | Primary solid Tumor |
| TCGA-BP-4994-01A-01R-1334-07 | 14 | 9 | 69 | Primary solid Tumor |
| TCGA-BP-4995-01A-01R-1334-07 | 14 | 9 | 69 | Primary solid Tumor |
| TCGA-BP-4998-01A-01R-1334-07 | 14 | 9 | 69 | Primary solid Tumor |
| TCGA-BP-4999-01A-01R-1334-07 | 14 | 9 | 69 | Primary solid Tumor |
| TCGA-BP-5000-01A-01R-1334-07 | 14 | 9 | 69 | Primary solid Tumor |
| TCGA-BP-5001-01A-01R-1334-07 | 14 | 9 | 69 | Primary solid Tumor |
| TCGA-BP-5004-01A-01R-1334-07 | 14 | 9 | 69 | Primary solid Tumor |
| TCGA-BP-5006-01A-01R-1334-07 | 14 | 9 | 69 | Primary solid Tumor |
| TCGA-BP-5007-01A-01R-1334-07 | 14 | 9 | 69 | Primary solid Tumor |
| TCGA-BP-5008-01A-01R-1334-07 | 14 | 9 | 69 | Primary solid Tumor |
| TCGA-BP-5009-01A-01R-1334-07 | 14 | 9 | 69 | Primary solid Tumor |
| TCGA-BP-5010-01A-02R-1420-07 | 14 | 9 | 68 | Primary solid Tumor |
| TCGA-BP-5168-01A-01R-1420-07 | 14 | 9 | 68 | Primary solid Tumor |
| TCGA-BP-5169-01A-01R-1426-07 | 14 | 9 | 70 | Primary solid Tumor |
| TCGA-BP-5170-01A-01R-1426-07 | 14 | 9 | 70 | Primary solid Tumor |
| TCGA-BP-5173-01A-01R-1426-07 | 14 | 9 | 70 | Primary solid Tumor |
| TCGA-BP-5174-01A-01R-1426-07 | 14 | 9 | 70 | Primary solid Tumor |
| TCGA-BP-5175-01A-01R-1426-07 | 14 | 9 | 70 | Primary solid Tumor |
| TCGA-BP-5176-01A-01R-1426-07 | 14 | 9 | 70 | Primary solid Tumor |
| TCGA-BP-5177-01A-01R-1426-07 | 14 | 9 | 70 | Primary solid Tumor |
| TCGA-BP-5178-01A-01R-1426-07 | 14 | 9 | 70 | Primary solid Tumor |
| TCGA-BP-5180-01A-01R-1426-07 | 14 | 9 | 70 | Primary solid Tumor |
| TCGA-BP-5181-01A-01R-1426-07 | 14 | 9 | 70 | Primary solid Tumor |
| TCGA-BP-5182-01A-01R-1426-07 | 14 | 9 | 70 | Primary solid Tumor |
| TCGA-BP-5183-01A-01R-1426-07 | 14 | 9 | 70 | Primary solid Tumor |
| TCGA-BP-5184-01A-01R-1426-07 | 14 | 9 | 70 | Primary solid Tumor |
| TCGA-BP-5185-01A-01R-1426-07 | 14 | NA | 70 | Primary solid Tumor |
| TCGA-BP-5186-01A-01R-1426-07 | 14 | 9 | 70 | Primary solid Tumor |
| TCGA-BP-5187-01A-01R-1426-07 | 14 | 9 | 70 | Primary solid Tumor |
| TCGA-BP-5189-01A-02R-1426-07 | 14 | 9 | 70 | Primary solid Tumor |
| TCGA-BP-5190-01A-01R-1426-07 | 14 | 9 | 70 | Primary solid Tumor |
| TCGA-BP-5191-01A-01R-1426-07 | 14 | 9 | 70 | Primary solid Tumor |
| TCGA-BP-5192-01A-01R-1426-07 | 14 | 9 | 70 | Primary solid Tumor |
| TCGA-BP-5194-01A-02R-1426-07 | 14 | 9 | 70 | Primary solid Tumor |
| TCGA-BP-5195-01A-02R-1426-07 | 14 | 9 | 70 | Primary solid Tumor |
| TCGA-BP-5196-01A-01R-1426-07 | 14 | 9 | 70 | Primary solid Tumor |
| TCGA-BP-5198-01A-01R-1426-07 | 14 | 9 | 70 | Primary solid Tumor |
| TCGA-BP-5199-01A-01R-1426-07 | 14 | 9 | 70 | Primary solid Tumor |
| TCGA-BP-5200-01A-01R-1426-07 | 14 | 9 | 70 | Primary solid Tumor |
| TCGA-BP-5201-01A-01R-1426-07 | 14 | 9 | 70 | Primary solid Tumor |
| TCGA-BP-5202-01A-02R-1426-07 | 14 | 9 | 70 | Primary solid Tumor |
| TCGA-BS-A0T9-01A-11R-A12I-07 | 16 | 11 | 94 | Primary solid Tumor |
| TCGA-BS-A0TA-01A-11R-A104-07 | 16 | 11 | 73 | Primary solid Tumor |
| TCGA-BS-A0TC-01A-11R-A104-07 | 16 | 11 | 73 | Primary solid Tumor |
| TCGA-BS-A0TD-01A-11R-A104-07 | 16 | 11 | 73 | Primary solid Tumor |
| TCGA-BS-A0TE-01A-11R-A104-07 | 7 | 15 | 73 | Primary solid Tumor |
| TCGA-BS-A0TG-01A-32R-A16W-07 | 16 | 11 | 73 | Primary solid Tumor |
| TCGA-BS-A0TI-01A-11R-A104-07 | 16 | 11 | 73 | Primary solid Tumor |
| TCGA-BS-A0TJ-01A-11R-A104-07 | 16 | 11 | 73 | Primary solid Tumor |
| TCGA-BS-A0U5-01A-11R-A109-07 | 16 | 11 | 75 | Primary solid Tumor |
| TCGA-BS-A0U7-01A-21R-A104-07 | 16 | 11 | 73 | Primary solid Tumor |
| TCGA-BS-A0U8-01A-11R-A104-07 | 16 | 11 | 73 | Primary solid Tumor |
| TCGA-BS-A0U9-01B-21R-A10J-07 | 16 | 11 | 81 | Primary solid Tumor |
| TCGA-BS-A0UA-01A-11R-A118-07 | 16 | 11 | 92 | Primary solid Tumor |
| TCGA-BS-A0UF-01A-11R-A104-07 | 16 | 11 | 73 | Primary solid Tumor |
| TCGA-BS-A0UJ-01A-12R-A104-07 | 16 | 11 | 73 | Primary solid Tumor |
| TCGA-BS-A0UL-01A-11R-A109-07 | 16 | 11 | 75 | Primary solid Tumor |
| TCGA-BS-A0UM-01A-11R-A104-07 | 16 | 11 | 73 | Primary solid Tumor |
| TCGA-BS-A0UT-01A-11R-A104-07 | 16 | 11 | 73 | Primary solid Tumor |
| TCGA-BS-A0UV-01A-11R-A16W-07 | 16 | 11 | 73 | Primary solid Tumor |
| TCGA-BS-A0V4-01A-11R-A14D-07 | 16 | NA | 121 | Primary solid Tumor |
| TCGA-BS-A0V6-01A-11R-A118-07 | 16 | 11 | 92 | Primary solid Tumor |
| TCGA-BS-A0V7-01A-21R-A118-07 | 16 | NA | 92 | Primary solid Tumor |
| TCGA-BS-A0V8-01A-11R-A118-07 | 16 | 11 | 92 | Primary solid Tumor |
| TCGA-BS-A0VI-01A-11R-A14D-07 | 16 | 11 | 121 | Primary solid Tumor |
| TCGA-BS-A0WQ-01A-21R-A109-07 | 16 | 11 | 75 | Primary solid Tumor |
| TCGA-BT-A0S7-01A-11R-A10U-07 | 2 | 1 | 86 | Primary solid Tumor |
| TCGA-BT-A0YX-01A-11R-A10U-07 | 2 | 4 | 86 | Primary solid Tumor |
| TCGA-BT-A20J-01A-11R-A14Y-07 | 2 | 5 | 128 | Primary solid Tumor |
| TCGA-BT-A20N-01A-11R-A14Y-07 | 1 | 1 | 128 | Primary solid Tumor |
| TCGA-BT-A20O-01A-21R-A14Y-07 | 3 | 2 | 128 | Primary solid Tumor |
| TCGA-BT-A20P-01A-11R-A14Y-07 | 1 | 1 | 128 | Primary solid Tumor |
| TCGA-BT-A20Q-01A-11R-A14Y-07 | 1 | 1 | 128 | Primary solid Tumor |
| TCGA-BT-A20R-01A-12R-A16R-07 | 1 | 1 | 150 | Primary solid Tumor |
| TCGA-BT-A20T-01A-11R-A14Y-07 | 1 | 1 | 128 | Primary solid Tumor |
| TCGA-BT-A20U-01A-11R-A14Y-07 | 1 | 4 | 128 | Primary solid Tumor |
| TCGA-BT-A20V-01A-11R-A14Y-07 | 2 | 4 | 128 | Primary solid Tumor |
| TCGA-BT-A20W-01A-21R-A14Y-07 | 1 | 1 | 128 | Primary solid Tumor |
| TCGA-BT-A20X-01A-11R-A16R-07 | 2 | 4 | 150 | Primary solid Tumor |
| TCGA-BT-A2LA-01A-11R-A18C-07 | 3 | 6 | 175 | Primary solid Tumor |
| TCGA-BT-A2LB-01A-11R-A18C-07 | 1 | 1 | 175 | Primary solid Tumor |
| TCGA-BT-A2LD-01A-12R-A20F-07 | 1 | 1 | 199 | Primary solid Tumor |
| TCGA-BT-A3PH-01A-11R-A220-07 | 1 | 1 | 223 | Primary solid Tumor |
| TCGA-BT-A3PJ-01A-21R-A220-07 | 2 | 4 | 223 | Primary solid Tumor |
| TCGA-BT-A3PK-01A-21R-A220-07 | 2 | 4 | 223 | Primary solid Tumor |
| TCGA-BT-A42C-01A-11R-A23N-07 | 1 | NA | 249 | Primary solid Tumor |
| TCGA-BT-A42E-01A-11R-A23W-07 | 2 | NA | 252 | Primary solid Tumor |
| TCGA-BT-A42F-01A-11R-A23W-07 | 2 | NA | 252 | Primary solid Tumor |
| TCGA-C4-A0EZ-01A-21R-A24X-07 | 3 | NA | 86 | Primary solid Tumor |
| TCGA-C4-A0F0-01A-12R-A10U-07 | 2 | 4 | 86 | Primary solid Tumor |
| TCGA-C4-A0F1-01A-11R-A034-07 | 2 | NA | 86 | Primary solid Tumor |
| TCGA-C4-A0F6-01A-11R-A10U-07 | 1 | 1 | 86 | Primary solid Tumor |
| TCGA-C4-A0F7-01A-11R-A084-07 | 2 | NA | 86 | Primary solid Tumor |
| TCGA-C8-A12K-01A-21R-A115-07 | 10 | 8 | 85 | Primary solid Tumor |
| TCGA-C8-A12L-01A-11R-A115-07 | 9 | 7 | 85 | Primary solid Tumor |
| TCGA-C8-A12M-01A-11R-A115-07 | 9 | 7 | 85 | Primary solid Tumor |
| TCGA-C8-A12N-01A-11R-A115-07 | 9 | 7 | 85 | Primary solid Tumor |
| TCGA-C8-A12O-01A-11R-A115-07 | 9 | 7 | 85 | Primary solid Tumor |
| TCGA-C8-A12P-01A-11R-A115-07 | 9 | 7 | 85 | Primary solid Tumor |
| TCGA-C8-A12Q-01A-11R-A115-07 | 9 | 7 | 85 | Primary solid Tumor |
| TCGA-C8-A12T-01A-11R-A115-07 | 9 | 7 | 85 | Primary solid Tumor |
| TCGA-C8-A12U-01A-11R-A115-07 | 9 | 7 | 85 | Primary solid Tumor |
| TCGA-C8-A12V-01A-11R-A115-07 | 10 | 8 | 85 | Primary solid Tumor |
| TCGA-C8-A12W-01A-11R-A115-07 | 9 | 7 | 85 | Primary solid Tumor |
| TCGA-C8-A12X-01A-11R-A115-07 | 9 | 7 | 85 | Primary solid Tumor |
| TCGA-C8-A12Y-01A-11R-A12D-07 | 9 | 7 | 93 | Primary solid Tumor |
| TCGA-C8-A12Z-01A-11R-A115-07 | 9 | 7 | 85 | Primary solid Tumor |
| TCGA-C8-A130-01A-31R-A115-07 | 9 | 7 | 85 | Primary solid Tumor |
| TCGA-C8-A131-01A-11R-A115-07 | 9 | 7 | 85 | Primary solid Tumor |
| TCGA-C8-A132-01A-31R-A115-07 | 9 | 7 | 85 | Primary solid Tumor |
| TCGA-C8-A133-01A-32R-A12D-07 | 9 | 7 | 93 | Primary solid Tumor |
| TCGA-C8-A134-01A-11R-A115-07 | 10 | 8 | 85 | Primary solid Tumor |
| TCGA-C8-A135-01A-11R-A115-07 | 9 | 7 | 85 | Primary solid Tumor |
| TCGA-C8-A137-01A-11R-A115-07 | 9 | 7 | 85 | Primary solid Tumor |
| TCGA-C8-A138-01A-11R-A115-07 | 9 | 7 | 85 | Primary solid Tumor |
| TCGA-C8-A1HE-01A-11R-A13Q-07 | 9 | 7 | 109 | Primary solid Tumor |
| TCGA-C8-A1HF-01A-11R-A137-07 | 9 | 7 | 103 | Primary solid Tumor |
| TCGA-C8-A1HG-01A-11R-A137-07 | 9 | 7 | 103 | Primary solid Tumor |
| TCGA-C8-A1HI-01A-11R-A137-07 | 9 | 7 | 103 | Primary solid Tumor |
| TCGA-C8-A1HJ-01A-11R-A13Q-07 | 10 | 8 | 109 | Primary solid Tumor |
| TCGA-C8-A1HK-01A-21R-A13Q-07 | 9 | 7 | 109 | Primary solid Tumor |
| TCGA-C8-A1HL-01A-11R-A137-07 | 9 | 7 | 103 | Primary solid Tumor |
| TCGA-C8-A1HM-01A-12R-A137-07 | 9 | 7 | 103 | Primary solid Tumor |
| TCGA-C8-A1HN-01A-11R-A137-07 | 9 | 7 | 103 | Primary solid Tumor |
| TCGA-C8-A1HO-01A-11R-A13Q-07 | 9 | 7 | 109 | Primary solid Tumor |
| TCGA-C8-A26V-01A-11R-A16F-07 | 9 | 7 | 147 | Primary solid Tumor |
| TCGA-C8-A26W-01A-11R-A16F-07 | 9 | 7 | 147 | Primary solid Tumor |
| TCGA-C8-A26X-01A-31R-A16F-07 | 9 | 7 | 147 | Primary solid Tumor |
| TCGA-C8-A26Y-01A-11R-A16F-07 | 12 | 7 | 147 | Primary solid Tumor |
| TCGA-C8-A26Z-01A-11R-A16F-07 | 9 | 7 | 147 | Primary solid Tumor |
| TCGA-C8-A273-01A-11R-A16F-07 | 9 | 7 | 147 | Primary solid Tumor |
| TCGA-C8-A274-01A-11R-A16F-07 | 9 | 7 | 147 | Primary solid Tumor |
| TCGA-C8-A275-01A-21R-A16F-07 | 9 | 7 | 147 | Primary solid Tumor |
| TCGA-C8-A278-01A-11R-A169-07 | 9 | 7 | 142 | Primary solid Tumor |
| TCGA-C8-A27A-01A-11R-A169-07 | 9 | 7 | 142 | Primary solid Tumor |
| TCGA-C8-A27B-01A-11R-A169-07 | 10 | 8 | 142 | Primary solid Tumor |
| TCGA-C8-A3M7-01A-12R-A21T-07 | 9 | 7 | 216 | Primary solid Tumor |
| TCGA-C8-A3M8-01A-11R-A213-07 | 9 | 7 | 202 | Primary solid Tumor |
| TCGA-C8-A8HP-01A-11R-A36F-07 | 9 | NA | 372 | Primary solid Tumor |
| TCGA-C8-A8HQ-01A-11R-A36F-07 | 9 | NA | 372 | Primary solid Tumor |
| TCGA-C8-A8HR-01A-11R-A36F-07 | 9 | NA | 372 | Primary solid Tumor |
| TCGA-C9-A47Z-01A-11R-A24H-07 | 2 | NA | 260 | Primary solid Tumor |
| TCGA-C9-A480-01A-12R-A24H-07 | 2 | NA | 260 | Primary solid Tumor |
| TCGA-CA-5254-01A-21R-1839-07 | 7 | NA | 138 | Primary solid Tumor |
| TCGA-CA-5255-01A-11R-1839-07 | 7 | NA | 138 | Primary solid Tumor |
| TCGA-CA-5256-01A-01R-1410-07 | 7 | 15 | 76 | Primary solid Tumor |
| TCGA-CA-5796-01A-01R-1653-07 | 7 | NA | 89 | Primary solid Tumor |
| TCGA-CA-5797-01A-01R-1653-07 | 7 | NA | 89 | Primary solid Tumor |
| TCGA-CA-6715-01A-21R-1839-07 | 7 | NA | 138 | Primary solid Tumor |
| TCGA-CA-6716-01A-11R-1839-07 | 7 | NA | 138 | Primary solid Tumor |
| TCGA-CA-6717-01A-11R-1839-07 | 7 | NA | 138 | Primary solid Tumor |
| TCGA-CA-6718-01A-11R-1839-07 | 7 | NA | 138 | Primary solid Tumor |
| TCGA-CA-6719-01A-11R-1839-07 | 7 | NA | 138 | Primary solid Tumor |
| TCGA-CF-A1HR-01A-11R-A13Y-07 | 1 | 1 | 113 | Primary solid Tumor |
| TCGA-CF-A1HS-01A-11R-A13Y-07 | 2 | 4 | 113 | Primary solid Tumor |
| TCGA-CF-A27C-01A-11R-A16R-07 | 1 | 1 | 150 | Primary solid Tumor |
| TCGA-CF-A3MF-01A-12R-A21D-07 | 1 | 1 | 207 | Primary solid Tumor |
| TCGA-CF-A3MG-01A-11R-A20F-07 | 1 | 1 | 199 | Primary solid Tumor |
| TCGA-CF-A3MH-01A-11R-A20F-07 | 1 | 1 | 199 | Primary solid Tumor |
| TCGA-CF-A3MI-01A-11R-A20F-07 | 1 | 1 | 199 | Primary solid Tumor |
| TCGA-CF-A47S-01A-11R-A23W-07 | 1 | NA | 252 | Primary solid Tumor |
| TCGA-CF-A47T-01A-11R-A23W-07 | 1 | NA | 252 | Primary solid Tumor |
| TCGA-CF-A47V-01A-11R-A23W-07 | 1 | NA | 252 | Primary solid Tumor |
| TCGA-CF-A47W-01A-11R-A23W-07 | 1 | NA | 252 | Primary solid Tumor |
| TCGA-CF-A47X-01A-31R-A23W-07 | 1 | NA | 252 | Primary solid Tumor |
| TCGA-CF-A47Y-01A-11R-A23W-07 | 1 | NA | 252 | Primary solid Tumor |
| TCGA-CF-A5U8-01A-11R-A28M-07 | 1 | NA | 294 | Primary solid Tumor |
| TCGA-CF-A5UA-01A-11R-A28M-07 | 1 | NA | 294 | Primary solid Tumor |
| TCGA-CF-A7I0-01A-22R-A352-07 | 1 | NA | 356 | Primary solid Tumor |
| TCGA-CF-A8HX-01A-11R-A36F-07 | 1 | NA | 367 | Primary solid Tumor |
| TCGA-CF-A8HY-01A-11R-A36F-07 | 1 | NA | 367 | Primary solid Tumor |
| TCGA-CF-A9FF-01A-11R-A38B-07 | 1 | NA | 391 | Primary solid Tumor |
| TCGA-CF-A9FH-01A-11R-A38B-07 | 1 | NA | 391 | Primary solid Tumor |
| TCGA-CF-A9FL-01A-11R-A38B-07 | 1 | NA | 391 | Primary solid Tumor |
| TCGA-CF-A9FM-01A-11R-A38B-07 | 1 | NA | 391 | Primary solid Tumor |
| TCGA-CI-6619-01B-11R-1830-07 | 7 | NA | 139 | Primary solid Tumor |
| TCGA-CI-6620-01A-11R-1830-07 | 7 | NA | 139 | Primary solid Tumor |
| TCGA-CI-6621-01A-11R-1830-07 | 7 | NA | 139 | Primary solid Tumor |
| TCGA-CI-6622-01A-11R-1830-07 | 7 | NA | 139 | Primary solid Tumor |
| TCGA-CI-6623-01B-11R-1830-07 | 7 | NA | 139 | Primary solid Tumor |
| TCGA-CI-6624-01C-11R-1830-07 | 7 | NA | 139 | Primary solid Tumor |
| TCGA-CJ-4634-01A-02R-1325-07 | 14 | 9 | 50 | Primary solid Tumor |
| TCGA-CJ-4635-01A-02R-1305-07 | 14 | 9 | 65 | Primary solid Tumor |
| TCGA-CJ-4636-01A-02R-1325-07 | 14 | 9 | 50 | Primary solid Tumor |
| TCGA-CJ-4637-01A-02R-1325-07 | 14 | 9 | 50 | Primary solid Tumor |
| TCGA-CJ-4638-01A-02R-1325-07 | 14 | 9 | 50 | Primary solid Tumor |
| TCGA-CJ-4639-01A-02R-1325-07 | 14 | 9 | 50 | Primary solid Tumor |
| TCGA-CJ-4640-01A-02R-1325-07 | 14 | 9 | 50 | Primary solid Tumor |
| TCGA-CJ-4641-01A-02R-1325-07 | 14 | 9 | 50 | Primary solid Tumor |
| TCGA-CJ-4642-01B-01R-1305-07 | 14 | NA | 65 | Primary solid Tumor |
| TCGA-CJ-4643-01A-02R-1325-07 | 14 | 9 | 50 | Primary solid Tumor |
| TCGA-CJ-4644-01A-02R-1325-07 | 14 | 9 | 50 | Primary solid Tumor |
| TCGA-CJ-4868-01A-01R-1305-07 | 14 | 9 | 65 | Primary solid Tumor |
| TCGA-CJ-4869-01A-02R-1426-07 | 14 | 9 | 70 | Primary solid Tumor |
| TCGA-CJ-4870-01A-01R-1305-07 | 14 | NA | 65 | Primary solid Tumor |
| TCGA-CJ-4871-01A-01R-1305-07 | 14 | 9 | 65 | Primary solid Tumor |
| TCGA-CJ-4872-01A-01R-1305-07 | 14 | 9 | 65 | Primary solid Tumor |
| TCGA-CJ-4873-01A-01R-1305-07 | 14 | 9 | 65 | Primary solid Tumor |
| TCGA-CJ-4874-01A-01R-1305-07 | 14 | 9 | 65 | Primary solid Tumor |
| TCGA-CJ-4875-01A-01R-1305-07 | 14 | 9 | 65 | Primary solid Tumor |
| TCGA-CJ-4876-01A-01R-1305-07 | 14 | 9 | 65 | Primary solid Tumor |
| TCGA-CJ-4878-01A-01R-1305-07 | 14 | 9 | 65 | Primary solid Tumor |
| TCGA-CJ-4881-01A-01R-1305-07 | 14 | 9 | 65 | Primary solid Tumor |
| TCGA-CJ-4882-01A-02R-1426-07 | 14 | 9 | 70 | Primary solid Tumor |
| TCGA-CJ-4884-01A-01R-1305-07 | 14 | 9 | 65 | Primary solid Tumor |
| TCGA-CJ-4885-01A-01R-1305-07 | 14 | 9 | 65 | Primary solid Tumor |
| TCGA-CJ-4886-01A-01R-1305-07 | 14 | 9 | 65 | Primary solid Tumor |
| TCGA-CJ-4887-01A-01R-1305-07 | 14 | 9 | 65 | Primary solid Tumor |
| TCGA-CJ-4888-01A-01R-1305-07 | 14 | 9 | 65 | Primary solid Tumor |
| TCGA-CJ-4889-01A-01R-1305-07 | 14 | 9 | 65 | Primary solid Tumor |
| TCGA-CJ-4890-01A-01R-1305-07 | 14 | 9 | 65 | Primary solid Tumor |
| TCGA-CJ-4891-01A-01R-1305-07 | 14 | 9 | 65 | Primary solid Tumor |
| TCGA-CJ-4892-01A-01R-1305-07 | 14 | 9 | 65 | Primary solid Tumor |
| TCGA-CJ-4893-01A-01R-1305-07 | 14 | 9 | 65 | Primary solid Tumor |
| TCGA-CJ-4894-01A-01R-1305-07 | 14 | 9 | 65 | Primary solid Tumor |
| TCGA-CJ-4895-01A-01R-1305-07 | 14 | 9 | 65 | Primary solid Tumor |
| TCGA-CJ-4897-01A-03R-1426-07 | 14 | 9 | 70 | Primary solid Tumor |
| TCGA-CJ-4899-01A-01R-1334-07 | 14 | 9 | 69 | Primary solid Tumor |
| TCGA-CJ-4900-01A-01R-1334-07 | 14 | 9 | 69 | Primary solid Tumor |
| TCGA-CJ-4901-01A-01R-1426-07 | 14 | 9 | 70 | Primary solid Tumor |
| TCGA-CJ-4902-01A-01R-1426-07 | 14 | 9 | 70 | Primary solid Tumor |
| TCGA-CJ-4903-01A-01R-1426-07 | 14 | 9 | 70 | Primary solid Tumor |
| TCGA-CJ-4904-01A-02R-1426-07 | 14 | 9 | 70 | Primary solid Tumor |
| TCGA-CJ-4905-01A-02R-1426-07 | 14 | 9 | 70 | Primary solid Tumor |
| TCGA-CJ-4907-01A-01R-1426-07 | 14 | 9 | 70 | Primary solid Tumor |
| TCGA-CJ-4908-01A-01R-1426-07 | 14 | 9 | 70 | Primary solid Tumor |
| TCGA-CJ-4912-01A-01R-1426-07 | 14 | 9 | 70 | Primary solid Tumor |
| TCGA-CJ-4916-01A-01R-1426-07 | 14 | 9 | 70 | Primary solid Tumor |
| TCGA-CJ-4918-01A-01R-1426-07 | 14 | 9 | 70 | Primary solid Tumor |
| TCGA-CJ-4920-01A-01R-1426-07 | 14 | 9 | 70 | Primary solid Tumor |
| TCGA-CJ-4923-01A-01R-1426-07 | 14 | 9 | 70 | Primary solid Tumor |
| TCGA-CJ-5671-01A-11R-1541-07 | 14 | 9 | 90 | Primary solid Tumor |
| TCGA-CJ-5672-01A-11R-1541-07 | 14 | 9 | 90 | Primary solid Tumor |
| TCGA-CJ-5675-01A-11R-1541-07 | 14 | 9 | 90 | Primary solid Tumor |
| TCGA-CJ-5676-01A-11R-1541-07 | 14 | 9 | 90 | Primary solid Tumor |
| TCGA-CJ-5677-01A-11R-1541-07 | 14 | 9 | 90 | Primary solid Tumor |
| TCGA-CJ-5678-01A-11R-1541-07 | 14 | 9 | 90 | Primary solid Tumor |
| TCGA-CJ-5679-01A-11R-1541-07 | 14 | 9 | 90 | Primary solid Tumor |
| TCGA-CJ-5680-01A-11R-1541-07 | 14 | 9 | 90 | Primary solid Tumor |
| TCGA-CJ-5681-01A-11R-1541-07 | 14 | 9 | 90 | Primary solid Tumor |
| TCGA-CJ-5682-01A-11R-1541-07 | 14 | 9 | 90 | Primary solid Tumor |
| TCGA-CJ-5683-01A-11R-1541-07 | 14 | 9 | 90 | Primary solid Tumor |
| TCGA-CJ-5684-01A-11R-1541-07 | 14 | 9 | 90 | Primary solid Tumor |
| TCGA-CJ-5686-01A-11R-1672-07 | 14 | 9 | 105 | Primary solid Tumor |
| TCGA-CJ-5689-01A-11R-1541-07 | 14 | 9 | 90 | Primary solid Tumor |
| TCGA-CJ-6027-01A-11R-1672-07 | 14 | 9 | 105 | Primary solid Tumor |
| TCGA-CJ-6028-01A-11R-1672-07 | 14 | 9 | 105 | Primary solid Tumor |
| TCGA-CJ-6030-01A-11R-1672-07 | 14 | 9 | 105 | Primary solid Tumor |
| TCGA-CJ-6031-01A-11R-1672-07 | 14 | 9 | 105 | Primary solid Tumor |
| TCGA-CJ-6032-01A-11R-1672-07 | 14 | 9 | 105 | Primary solid Tumor |
| TCGA-CJ-6033-01A-11R-1672-07 | 14 | 9 | 105 | Primary solid Tumor |
| TCGA-CK-4947-01B-11R-1653-07 | 7 | NA | 89 | Primary solid Tumor |
| TCGA-CK-4948-01B-11R-1653-07 | 7 | NA | 89 | Primary solid Tumor |
| TCGA-CK-4950-01A-01R-1723-07 | 7 | NA | 116 | Primary solid Tumor |
| TCGA-CK-4951-01A-01R-1410-07 | 7 | 15 | 76 | Primary solid Tumor |
| TCGA-CK-4952-01A-01R-1723-07 | 7 | NA | 116 | Primary solid Tumor |
| TCGA-CK-5912-01A-11R-1653-07 | 7 | NA | 89 | Primary solid Tumor |
| TCGA-CK-5913-01A-11R-1653-07 | 7 | NA | 89 | Primary solid Tumor |
| TCGA-CK-5914-01A-11R-1653-07 | 7 | NA | 89 | Primary solid Tumor |
| TCGA-CK-5915-01A-11R-1653-07 | 7 | NA | 89 | Primary solid Tumor |
| TCGA-CK-5916-01A-11R-1653-07 | 7 | NA | 89 | Primary solid Tumor |
| TCGA-CK-6746-01A-11R-1839-07 | 7 | NA | 138 | Primary solid Tumor |
| TCGA-CK-6747-01A-11R-1839-07 | 7 | NA | 138 | Primary solid Tumor |
| TCGA-CK-6748-01A-11R-1839-07 | 7 | NA | 138 | Primary solid Tumor |
| TCGA-CK-6751-01A-11R-1839-07 | 7 | NA | 138 | Primary solid Tumor |
| TCGA-CL-4957-01A-01R-1736-07 | 7 | NA | 122 | Primary solid Tumor |
| TCGA-CL-5917-01A-11R-1660-07 | 7 | NA | 102 | Primary solid Tumor |
| TCGA-CL-5918-01A-11R-1660-07 | 7 | NA | 102 | Primary solid Tumor |
| TCGA-CM-4743-01A-01R-1723-07 | 7 | NA | 116 | Primary solid Tumor |
| TCGA-CM-4744-01A-01R-A32Z-07 | 7 | NA | 76 | Primary solid Tumor |
| TCGA-CM-4746-01A-01R-1410-07 | 7 | 15 | 76 | Primary solid Tumor |
| TCGA-CM-4747-01A-01R-1410-07 | 7 | 15 | 76 | Primary solid Tumor |
| TCGA-CM-4748-01A-01R-1410-07 | 7 | 15 | 76 | Primary solid Tumor |
| TCGA-CM-4750-01A-01R-1410-07 | 7 | 15 | 76 | Primary solid Tumor |
| TCGA-CM-4751-01A-02R-1839-07 | 7 | NA | 138 | Primary solid Tumor |
| TCGA-CM-4752-01A-01R-1410-07 | 7 | 15 | 76 | Primary solid Tumor |
| TCGA-CM-5341-01A-01R-1410-07 | 7 | 15 | 76 | Primary solid Tumor |
| TCGA-CM-5344-01A-21R-1723-07 | 7 | NA | 116 | Primary solid Tumor |
| TCGA-CM-5348-01A-21R-1723-07 | 7 | NA | 116 | Primary solid Tumor |
| TCGA-CM-5349-01A-21R-1723-07 | 7 | NA | 116 | Primary solid Tumor |
| TCGA-CM-5860-01A-01R-1653-07 | 7 | NA | 89 | Primary solid Tumor |
| TCGA-CM-5861-01A-01R-1653-07 | 7 | NA | 89 | Primary solid Tumor |
| TCGA-CM-5862-01A-01R-1653-07 | 7 | NA | 89 | Primary solid Tumor |
| TCGA-CM-5863-01A-21R-1839-07 | 7 | NA | 138 | Primary solid Tumor |
| TCGA-CM-5864-01A-01R-1653-07 | 7 | NA | 89 | Primary solid Tumor |
| TCGA-CM-5868-01A-01R-1653-07 | 7 | NA | 89 | Primary solid Tumor |
| TCGA-CM-6161-01A-11R-1653-07 | 7 | NA | 89 | Primary solid Tumor |
| TCGA-CM-6162-01A-11R-1653-07 | 7 | NA | 89 | Primary solid Tumor |
| TCGA-CM-6163-01A-11R-1653-07 | 7 | NA | 89 | Primary solid Tumor |
| TCGA-CM-6164-01A-11R-1653-07 | 7 | NA | 89 | Primary solid Tumor |
| TCGA-CM-6165-01A-11R-1653-07 | 7 | NA | 89 | Primary solid Tumor |
| TCGA-CM-6166-01A-11R-1653-07 | 7 | NA | 89 | Primary solid Tumor |
| TCGA-CM-6167-01A-11R-1653-07 | 7 | NA | 89 | Primary solid Tumor |
| TCGA-CM-6168-01A-11R-1653-07 | 7 | NA | 89 | Primary solid Tumor |
| TCGA-CM-6169-01A-11R-1653-07 | 7 | NA | 89 | Primary solid Tumor |
| TCGA-CM-6170-01A-11R-1653-07 | 7 | NA | 89 | Primary solid Tumor |
| TCGA-CM-6171-01A-11R-1653-07 | 7 | NA | 89 | Primary solid Tumor |
| TCGA-CM-6172-01A-11R-1653-07 | 7 | NA | 89 | Primary solid Tumor |
| TCGA-CM-6674-01A-11R-1839-07 | 7 | NA | 138 | Primary solid Tumor |
| TCGA-CM-6675-01A-11R-1839-07 | 7 | NA | 138 | Primary solid Tumor |
| TCGA-CM-6676-01A-11R-1839-07 | 7 | NA | 138 | Primary solid Tumor |
| TCGA-CM-6677-01A-11R-1839-07 | 7 | NA | 138 | Primary solid Tumor |
| TCGA-CM-6678-01A-11R-1839-07 | 7 | NA | 138 | Primary solid Tumor |
| TCGA-CM-6679-01A-11R-1839-07 | 7 | NA | 138 | Primary solid Tumor |
| TCGA-CM-6680-01A-11R-1839-07 | 7 | NA | 138 | Primary solid Tumor |
| TCGA-CN-4722-01A-01R-1436-07 | 2 | 4 | 54 | Primary solid Tumor |
| TCGA-CN-4723-01A-01R-1436-07 | 2 | 4 | 54 | Primary solid Tumor |
| TCGA-CN-4725-01A-01R-1436-07 | 2 | 4 | 54 | Primary solid Tumor |
| TCGA-CN-4726-01A-01R-1436-07 | 2 | 4 | 54 | Primary solid Tumor |
| TCGA-CN-4727-01A-01R-1436-07 | 2 | 4 | 54 | Primary solid Tumor |
| TCGA-CN-4728-01A-01R-1436-07 | 2 | 4 | 54 | Primary solid Tumor |
| TCGA-CN-4729-01A-01R-1436-07 | 2 | 4 | 54 | Primary solid Tumor |
| TCGA-CN-4730-01A-01R-1436-07 | 2 | 4 | 54 | Primary solid Tumor |
| TCGA-CN-4731-01A-01R-1436-07 | 2 | 4 | 54 | Primary solid Tumor |
| TCGA-CN-4733-01A-02R-1873-07 | 2 | 4 | 145 | Primary solid Tumor |
| TCGA-CN-4734-01A-01R-1436-07 | 2 | 4 | 54 | Primary solid Tumor |
| TCGA-CN-4735-01A-01R-1436-07 | 2 | 4 | 54 | Primary solid Tumor |
| TCGA-CN-4736-01A-01R-1436-07 | 2 | 4 | 54 | Primary solid Tumor |
| TCGA-CN-4737-01A-01R-1436-07 | 2 | 4 | 54 | Primary solid Tumor |
| TCGA-CN-4738-01A-02R-1514-07 | 2 | 4 | 83 | Primary solid Tumor |
| TCGA-CN-4739-01A-02R-1514-07 | 2 | 4 | 83 | Primary solid Tumor |
| TCGA-CN-4740-01A-01R-1436-07 | 2 | 4 | 54 | Primary solid Tumor |
| TCGA-CN-4741-01A-01R-1436-07 | 2 | 4 | 54 | Primary solid Tumor |
| TCGA-CN-4742-01A-02R-1514-07 | 2 | 4 | 83 | Primary solid Tumor |
| TCGA-CN-5355-01A-01R-1436-07 | 2 | 4 | 54 | Primary solid Tumor |
| TCGA-CN-5356-01A-01R-1436-07 | 2 | 4 | 54 | Primary solid Tumor |
| TCGA-CN-5358-01A-01R-1514-07 | 2 | 4 | 83 | Primary solid Tumor |
| TCGA-CN-5359-01A-01R-1436-07 | 2 | 4 | 54 | Primary solid Tumor |
| TCGA-CN-5360-01A-01R-1436-07 | 2 | 4 | 54 | Primary solid Tumor |
| TCGA-CN-5361-01A-01R-1436-07 | 2 | 4 | 54 | Primary solid Tumor |
| TCGA-CN-5363-01A-01R-1436-07 | 2 | 4 | 54 | Primary solid Tumor |
| TCGA-CN-5364-01A-01R-1436-07 | 2 | 4 | 54 | Primary solid Tumor |
| TCGA-CN-5365-01A-01R-1436-07 | 2 | 4 | 54 | Primary solid Tumor |
| TCGA-CN-5366-01A-01R-1436-07 | 2 | 4 | 54 | Primary solid Tumor |
| TCGA-CN-5367-01A-01R-1436-07 | 2 | 4 | 54 | Primary solid Tumor |
| TCGA-CN-5369-01A-01R-1436-07 | 2 | 4 | 54 | Primary solid Tumor |
| TCGA-CN-5370-01A-01R-2016-07 | 2 | 4 | 164 | Primary solid Tumor |
| TCGA-CN-5373-01A-01R-1436-07 | 2 | 4 | 54 | Primary solid Tumor |
| TCGA-CN-5374-01A-01R-1436-07 | 2 | 4 | 54 | Primary solid Tumor |
| TCGA-CN-6010-01A-11R-1686-07 | 2 | 4 | 107 | Primary solid Tumor |
| TCGA-CN-6011-01A-11R-1686-07 | 2 | 4 | 107 | Primary solid Tumor |
| TCGA-CN-6012-01A-11R-1686-07 | 2 | 4 | 107 | Primary solid Tumor |
| TCGA-CN-6013-01A-11R-1686-07 | 2 | 4 | 107 | Primary solid Tumor |
| TCGA-CN-6016-01A-11R-1686-07 | 2 | 4 | 107 | Primary solid Tumor |
| TCGA-CN-6017-01A-11R-1686-07 | 2 | 4 | 107 | Primary solid Tumor |
| TCGA-CN-6018-01A-11R-1686-07 | 2 | 4 | 107 | Primary solid Tumor |
| TCGA-CN-6019-01A-11R-1686-07 | 2 | 4 | 107 | Primary solid Tumor |
| TCGA-CN-6020-01A-11R-1686-07 | 2 | 4 | 107 | Primary solid Tumor |
| TCGA-CN-6021-01A-11R-1686-07 | 2 | 4 | 107 | Primary solid Tumor |
| TCGA-CN-6022-01A-21R-1686-07 | 2 | 4 | 107 | Primary solid Tumor |
| TCGA-CN-6023-01A-11R-1686-07 | 2 | 4 | 107 | Primary solid Tumor |
| TCGA-CN-6024-01A-11R-1686-07 | 2 | 4 | 107 | Primary solid Tumor |
| TCGA-CN-6988-01A-11R-1915-07 | 2 | 4 | 151 | Primary solid Tumor |
| TCGA-CN-6989-01A-11R-1915-07 | 2 | 4 | 151 | Primary solid Tumor |
| TCGA-CN-6992-01A-11R-1915-07 | 2 | 4 | 151 | Primary solid Tumor |
| TCGA-CN-6994-01A-11R-1915-07 | 2 | 4 | 151 | Primary solid Tumor |
| TCGA-CN-6995-01A-31R-2016-07 | 2 | 4 | 164 | Primary solid Tumor |
| TCGA-CN-6996-01A-11R-1915-07 | 2 | NA | 151 | Primary solid Tumor |
| TCGA-CN-6997-01A-11R-2016-07 | 2 | 4 | 164 | Primary solid Tumor |
| TCGA-CN-6998-01A-23R-2016-07 | 2 | 4 | 164 | Primary solid Tumor |
| TCGA-CN-A497-01A-11R-A24H-07 | 2 | NA | 260 | Primary solid Tumor |
| TCGA-CN-A498-01A-11R-A24H-07 | 2 | NA | 260 | Primary solid Tumor |
| TCGA-CN-A499-01A-11R-A24H-07 | 2 | NA | 260 | Primary solid Tumor |
| TCGA-CN-A49A-01A-11R-A24H-07 | 2 | NA | 260 | Primary solid Tumor |
| TCGA-CN-A49B-01A-31R-A24H-07 | 2 | NA | 260 | Primary solid Tumor |
| TCGA-CN-A49C-01A-11R-A24H-07 | 2 | NA | 260 | Primary solid Tumor |
| TCGA-CN-A63T-01A-11R-A28V-07 | 2 | NA | 301 | Primary solid Tumor |
| TCGA-CN-A63U-01A-11R-A30B-07 | 2 | NA | 311 | Primary solid Tumor |
| TCGA-CN-A63V-01A-11R-A466-07 | 2 | NA | 301 | Primary solid Tumor |
| TCGA-CN-A63W-01A-11R-A30B-07 | 2 | NA | 311 | Primary solid Tumor |
| TCGA-CN-A641-01A-11R-A30B-07 | 2 | NA | 311 | Primary solid Tumor |
| TCGA-CN-A642-01A-12R-A30B-07 | 2 | NA | 311 | Primary solid Tumor |
| TCGA-CN-A6UY-01A-12R-A34R-07 | 2 | NA | 355 | Primary solid Tumor |
| TCGA-CN-A6V1-01A-12R-A34R-07 | 2 | NA | 355 | Primary solid Tumor |
| TCGA-CN-A6V3-01A-12R-A34R-07 | 2 | NA | 355 | Primary solid Tumor |
| TCGA-CN-A6V6-01A-12R-A34R-07 | 2 | NA | 355 | Primary solid Tumor |
| TCGA-CN-A6V7-01A-12R-A34R-07 | 2 | NA | 355 | Primary solid Tumor |
| TCGA-CQ-5323-01A-01R-1686-07 | 2 | 4 | 107 | Primary solid Tumor |
| TCGA-CQ-5324-01A-01R-1686-07 | 2 | 4 | 107 | Primary solid Tumor |
| TCGA-CQ-5325-01A-01R-1686-07 | 2 | 4 | 107 | Primary solid Tumor |
| TCGA-CQ-5326-01A-01R-1873-07 | 2 | 4 | 145 | Primary solid Tumor |
| TCGA-CQ-5327-01A-01R-1686-07 | 2 | NA | 107 | Primary solid Tumor |
| TCGA-CQ-5329-01A-01R-1686-07 | 2 | 4 | 107 | Primary solid Tumor |
| TCGA-CQ-5330-01A-01R-1686-07 | 2 | 4 | 107 | Primary solid Tumor |
| TCGA-CQ-5331-01A-02R-1873-07 | 2 | 4 | 145 | Primary solid Tumor |
| TCGA-CQ-5332-01A-01R-1686-07 | 2 | 4 | 107 | Primary solid Tumor |
| TCGA-CQ-5333-01A-01R-2403-07 | 2 | NA | 241 | Primary solid Tumor |
| TCGA-CQ-5334-01A-01R-1686-07 | 2 | 4 | 107 | Primary solid Tumor |
| TCGA-CQ-6218-01A-11R-1915-07 | 2 | 4 | 151 | Primary solid Tumor |
| TCGA-CQ-6219-01A-11R-1915-07 | 2 | 4 | 151 | Primary solid Tumor |
| TCGA-CQ-6220-01A-11R-1915-07 | 2 | 4 | 151 | Primary solid Tumor |
| TCGA-CQ-6221-01A-11R-2081-07 | 3 | 6 | 188 | Primary solid Tumor |
| TCGA-CQ-6222-01A-11R-1915-07 | 2 | 4 | 151 | Primary solid Tumor |
| TCGA-CQ-6223-01A-11R-1915-07 | 2 | 4 | 151 | Primary solid Tumor |
| TCGA-CQ-6224-01A-11R-1915-07 | 2 | 4 | 151 | Primary solid Tumor |
| TCGA-CQ-6225-01A-11R-1915-07 | 2 | 4 | 151 | Primary solid Tumor |
| TCGA-CQ-6227-01A-11R-1915-07 | 2 | 4 | 151 | Primary solid Tumor |
| TCGA-CQ-6228-01A-11R-1915-07 | 2 | 4 | 151 | Primary solid Tumor |
| TCGA-CQ-6229-01A-11R-1915-07 | 2 | 4 | 151 | Primary solid Tumor |
| TCGA-CQ-7063-01A-11R-2403-07 | 2 | NA | 241 | Primary solid Tumor |
| TCGA-CQ-7065-01A-11R-2081-07 | 2 | 4 | 188 | Primary solid Tumor |
| TCGA-CQ-7067-01A-11R-2232-07 | 2 | 4 | 215 | Primary solid Tumor |
| TCGA-CQ-7068-01A-11R-2081-07 | 2 | 4 | 188 | Primary solid Tumor |
| TCGA-CQ-7069-01A-11R-2403-07 | 2 | NA | 241 | Primary solid Tumor |
| TCGA-CQ-7071-01A-12R-A30B-07 | 2 | NA | 311 | Primary solid Tumor |
| TCGA-CQ-7072-01A-21R-A30B-07 | 2 | NA | 311 | Primary solid Tumor |
| TCGA-CQ-A4C6-01A-11R-A24Z-07 | 2 | NA | 265 | Primary solid Tumor |
| TCGA-CQ-A4C7-01A-11R-A24Z-07 | 2 | NA | 265 | Primary solid Tumor |
| TCGA-CQ-A4C9-01A-11R-A24Z-07 | 2 | NA | 265 | Primary solid Tumor |
| TCGA-CQ-A4CA-01A-11R-A24Z-07 | 2 | NA | 265 | Primary solid Tumor |
| TCGA-CQ-A4CB-01A-11R-A24Z-07 | 2 | NA | 265 | Primary solid Tumor |
| TCGA-CQ-A4CD-01A-21R-A24Z-07 | 2 | NA | 265 | Primary solid Tumor |
| TCGA-CQ-A4CE-01A-11R-A266-07 | 2 | NA | 273 | Primary solid Tumor |
| TCGA-CQ-A4CG-01A-11R-A266-07 | 2 | NA | 273 | Primary solid Tumor |
| TCGA-CQ-A4CH-01A-11R-A266-07 | 2 | NA | 273 | Primary solid Tumor |
| TCGA-CQ-A4CI-01A-11R-A266-07 | 2 | NA | 273 | Primary solid Tumor |
| TCGA-CR-5243-01A-01R-1514-07 | 2 | 4 | 83 | Primary solid Tumor |
| TCGA-CR-5247-01A-01R-2016-07 | 2 | 4 | 164 | Primary solid Tumor |
| TCGA-CR-5248-01A-01R-2016-07 | 2 | 4 | 164 | Primary solid Tumor |
| TCGA-CR-5249-01A-01R-1514-07 | 2 | 4 | 83 | Primary solid Tumor |
| TCGA-CR-5250-01A-01R-1514-07 | 2 | 4 | 83 | Primary solid Tumor |
| TCGA-CR-6467-01A-11R-1873-07 | 2 | 4 | 145 | Primary solid Tumor |
| TCGA-CR-6470-01A-11R-1873-07 | 2 | 4 | 145 | Primary solid Tumor |
| TCGA-CR-6471-01A-11R-1873-07 | 2 | 4 | 145 | Primary solid Tumor |
| TCGA-CR-6472-01A-11R-1873-07 | 2 | 4 | 145 | Primary solid Tumor |
| TCGA-CR-6473-01A-11R-1873-07 | 2 | 4 | 145 | Primary solid Tumor |
| TCGA-CR-6474-01A-11R-1873-07 | 2 | 4 | 145 | Primary solid Tumor |
| TCGA-CR-6477-01A-11R-1873-07 | 2 | 4 | 145 | Primary solid Tumor |
| TCGA-CR-6478-01A-11R-1873-07 | 2 | 4 | 145 | Primary solid Tumor |
| TCGA-CR-6480-01A-11R-1873-07 | 2 | NA | 145 | Primary solid Tumor |
| TCGA-CR-6481-01A-11R-1873-07 | 2 | 4 | 145 | Primary solid Tumor |
| TCGA-CR-6482-01A-11R-1873-07 | 2 | 4 | 145 | Primary solid Tumor |
| TCGA-CR-6484-01A-11R-1873-07 | 2 | 4 | 145 | Primary solid Tumor |
| TCGA-CR-6487-01A-11R-1873-07 | 2 | 4 | 145 | Primary solid Tumor |
| TCGA-CR-6488-01A-12R-2081-07 | 2 | 4 | 188 | Primary solid Tumor |
| TCGA-CR-6491-01A-11R-1873-07 | 2 | 4 | 145 | Primary solid Tumor |
| TCGA-CR-6492-01A-12R-2081-07 | 2 | 4 | 188 | Primary solid Tumor |
| TCGA-CR-6493-01A-11R-1873-07 | 2 | 4 | 145 | Primary solid Tumor |
| TCGA-CR-7364-01A-11R-2016-07 | 2 | 4 | 164 | Primary solid Tumor |
| TCGA-CR-7365-01A-11R-2016-07 | 2 | 4 | 164 | Primary solid Tumor |
| TCGA-CR-7367-01A-11R-2016-07 | 2 | 4 | 164 | Primary solid Tumor |
| TCGA-CR-7368-01A-11R-2132-07 | 2 | 4 | 190 | Primary solid Tumor |
| TCGA-CR-7369-01A-11R-2132-07 | 2 | 4 | 190 | Primary solid Tumor |
| TCGA-CR-7370-01A-11R-2132-07 | 2 | 4 | 190 | Primary solid Tumor |
| TCGA-CR-7371-01A-11R-2016-07 | 2 | 4 | 164 | Primary solid Tumor |
| TCGA-CR-7372-01A-11R-2016-07 | 2 | 4 | 164 | Primary solid Tumor |
| TCGA-CR-7373-01A-11R-2016-07 | 2 | 4 | 164 | Primary solid Tumor |
| TCGA-CR-7374-01A-11R-2016-07 | 2 | 4 | 164 | Primary solid Tumor |
| TCGA-CR-7376-01A-11R-2132-07 | 2 | 4 | 190 | Primary solid Tumor |
| TCGA-CR-7377-01A-11R-2016-07 | 2 | 4 | 164 | Primary solid Tumor |
| TCGA-CR-7379-01A-11R-2016-07 | 2 | 4 | 164 | Primary solid Tumor |
| TCGA-CR-7380-01A-11R-2016-07 | 2 | 4 | 164 | Primary solid Tumor |
| TCGA-CR-7382-01A-11R-2132-07 | 2 | 4 | 190 | Primary solid Tumor |
| TCGA-CR-7383-01A-11R-2132-07 | 2 | 4 | 190 | Primary solid Tumor |
| TCGA-CR-7385-01A-11R-2016-07 | 2 | 4 | 164 | Primary solid Tumor |
| TCGA-CR-7386-01A-11R-2016-07 | 2 | 4 | 164 | Primary solid Tumor |
| TCGA-CR-7388-01A-11R-2016-07 | 2 | 4 | 164 | Primary solid Tumor |
| TCGA-CR-7389-01A-11R-2016-07 | 2 | 4 | 164 | Primary solid Tumor |
| TCGA-CR-7390-01A-11R-2016-07 | 2 | 4 | 164 | Primary solid Tumor |
| TCGA-CR-7391-01A-11R-2016-07 | 2 | 4 | 164 | Primary solid Tumor |
| TCGA-CR-7392-01A-11R-2016-07 | 2 | 4 | 164 | Primary solid Tumor |
| TCGA-CR-7393-01A-11R-2016-07 | 2 | 4 | 164 | Primary solid Tumor |
| TCGA-CR-7394-01A-11R-2016-07 | 2 | 4 | 164 | Primary solid Tumor |
| TCGA-CR-7395-01A-11R-2016-07 | 2 | 4 | 164 | Primary solid Tumor |
| TCGA-CR-7397-01A-11R-2016-07 | 2 | 4 | 164 | Primary solid Tumor |
| TCGA-CR-7398-01A-11R-2016-07 | 2 | 4 | 164 | Primary solid Tumor |
| TCGA-CR-7399-01A-11R-2016-07 | 2 | 4 | 164 | Primary solid Tumor |
| TCGA-CR-7401-01A-11R-2016-07 | 2 | 4 | 164 | Primary solid Tumor |
| TCGA-CR-7402-01A-11R-2016-07 | 2 | 4 | 164 | Primary solid Tumor |
| TCGA-CR-7404-01A-11R-2132-07 | 2 | 4 | 190 | Primary solid Tumor |
| TCGA-CU-A0YN-01A-21R-A10U-07 | 2 | 4 | 86 | Primary solid Tumor |
| TCGA-CU-A0YO-01A-11R-A10U-07 | 1 | 1 | 86 | Primary solid Tumor |
| TCGA-CU-A0YR-01A-12R-A10U-07 | 1 | 1 | 86 | Primary solid Tumor |
| TCGA-CU-A3KJ-01A-11R-A21D-07 | 2 | 4 | 207 | Primary solid Tumor |
| TCGA-CU-A3QU-01A-11R-A22U-07 | 1 | 1 | 235 | Primary solid Tumor |
| TCGA-CU-A3YL-01A-11R-A22U-07 | 1 | 1 | 235 | Primary solid Tumor |
| TCGA-CU-A5W6-01A-11R-A28M-07 | 1 | NA | 294 | Primary solid Tumor |
| TCGA-CU-A72E-01A-12R-A33J-07 | 1 | NA | 337 | Primary solid Tumor |
| TCGA-CV-5430-01A-02R-1686-07 | 2 | 4 | 107 | Primary solid Tumor |
| TCGA-CV-5431-01A-01R-1514-07 | 2 | 4 | 83 | Primary solid Tumor |
| TCGA-CV-5432-01A-02R-1686-07 | 2 | 4 | 107 | Primary solid Tumor |
| TCGA-CV-5434-01A-01R-1686-07 | 2 | 4 | 107 | Primary solid Tumor |
| TCGA-CV-5435-01A-01R-1686-07 | 2 | 4 | 107 | Primary solid Tumor |
| TCGA-CV-5436-01A-01R-1514-07 | 2 | 4 | 83 | Primary solid Tumor |
| TCGA-CV-5439-01A-01R-1686-07 | 2 | 4 | 107 | Primary solid Tumor |
| TCGA-CV-5440-01A-01R-1514-07 | 2 | 4 | 83 | Primary solid Tumor |
| TCGA-CV-5441-01A-01R-1514-07 | 2 | 4 | 83 | Primary solid Tumor |
| TCGA-CV-5442-01A-01R-1514-07 | 2 | 4 | 83 | Primary solid Tumor |
| TCGA-CV-5443-01A-01R-1514-07 | 2 | 4 | 83 | Primary solid Tumor |
| TCGA-CV-5444-01A-02R-1514-07 | 2 | 4 | 83 | Primary solid Tumor |
| TCGA-CV-5966-01A-11R-1686-07 | 2 | 4 | 107 | Primary solid Tumor |
| TCGA-CV-5970-01A-11R-1686-07 | 2 | 4 | 107 | Primary solid Tumor |
| TCGA-CV-5971-01A-11R-1686-07 | 2 | 4 | 107 | Primary solid Tumor |
| TCGA-CV-5973-01A-11R-1686-07 | 2 | 4 | 107 | Primary solid Tumor |
| TCGA-CV-5976-01A-11R-1686-07 | 2 | 4 | 107 | Primary solid Tumor |
| TCGA-CV-5977-01A-11R-1686-07 | 2 | 4 | 107 | Primary solid Tumor |
| TCGA-CV-5978-01A-11R-1686-07 | 2 | 4 | 107 | Primary solid Tumor |
| TCGA-CV-5979-01A-11R-1686-07 | 2 | 4 | 107 | Primary solid Tumor |
| TCGA-CV-6003-01A-11R-1686-07 | 2 | 4 | 107 | Primary solid Tumor |
| TCGA-CV-6433-01A-11R-1686-07 | 2 | 4 | 107 | Primary solid Tumor |
| TCGA-CV-6436-01A-11R-1686-07 | 2 | 4 | 107 | Primary solid Tumor |
| TCGA-CV-6441-01A-11R-1686-07 | 2 | 4 | 107 | Primary solid Tumor |
| TCGA-CV-6933-01A-11R-1915-07 | 2 | 4 | 151 | Primary solid Tumor |
| TCGA-CV-6934-01A-11R-1915-07 | 2 | 4 | 151 | Primary solid Tumor |
| TCGA-CV-6935-01A-11R-1915-07 | 2 | 4 | 151 | Primary solid Tumor |
| TCGA-CV-6936-01A-11R-1915-07 | 2 | 4 | 151 | Primary solid Tumor |
| TCGA-CV-6937-01A-11R-2016-07 | 2 | 4 | 164 | Primary solid Tumor |
| TCGA-CV-6938-01A-11R-1915-07 | 2 | 4 | 151 | Primary solid Tumor |
| TCGA-CV-6939-01A-11R-1915-07 | 2 | 4 | 151 | Primary solid Tumor |
| TCGA-CV-6940-01A-11R-1915-07 | 2 | 4 | 151 | Primary solid Tumor |
| TCGA-CV-6941-01A-11R-1915-07 | 2 | 4 | 151 | Primary solid Tumor |
| TCGA-CV-6942-01A-21R-2016-07 | 2 | 4 | 164 | Primary solid Tumor |
| TCGA-CV-6943-01A-11R-1915-07 | 2 | 4 | 151 | Primary solid Tumor |
| TCGA-CV-6945-01A-11R-1915-07 | 2 | 4 | 151 | Primary solid Tumor |
| TCGA-CV-6948-01A-11R-1915-07 | 2 | 4 | 151 | Primary solid Tumor |
| TCGA-CV-6950-01A-11R-1915-07 | 2 | 4 | 151 | Primary solid Tumor |
| TCGA-CV-6951-01A-11R-1915-07 | 2 | 4 | 151 | Primary solid Tumor |
| TCGA-CV-6952-01A-11R-1915-07 | 2 | 4 | 151 | Primary solid Tumor |
| TCGA-CV-6953-01A-11R-1915-07 | 2 | 4 | 151 | Primary solid Tumor |
| TCGA-CV-6954-01A-11R-1915-07 | 2 | 4 | 151 | Primary solid Tumor |
| TCGA-CV-6955-01A-11R-2016-07 | 2 | 4 | 164 | Primary solid Tumor |
| TCGA-CV-6956-01A-21R-2016-07 | 2 | 4 | 164 | Primary solid Tumor |
| TCGA-CV-6959-01A-11R-1915-07 | 2 | 4 | 151 | Primary solid Tumor |
| TCGA-CV-6960-01A-41R-2016-07 | 2 | 4 | 164 | Primary solid Tumor |
| TCGA-CV-6961-01A-21R-1915-07 | 2 | 4 | 151 | Primary solid Tumor |
| TCGA-CV-6962-01A-11R-1915-07 | 2 | 4 | 151 | Primary solid Tumor |
| TCGA-CV-7089-01A-11R-2016-07 | 2 | 4 | 164 | Primary solid Tumor |
| TCGA-CV-7090-01A-11R-2016-07 | 2 | 4 | 164 | Primary solid Tumor |
| TCGA-CV-7091-01A-11R-2016-07 | 2 | 4 | 164 | Primary solid Tumor |
| TCGA-CV-7095-01A-21R-2016-07 | 2 | 4 | 164 | Primary solid Tumor |
| TCGA-CV-7097-01A-11R-2016-07 | 2 | 4 | 164 | Primary solid Tumor |
| TCGA-CV-7099-01A-41R-2016-07 | 2 | 4 | 164 | Primary solid Tumor |
| TCGA-CV-7100-01A-11R-2016-07 | 2 | 4 | 164 | Primary solid Tumor |
| TCGA-CV-7101-01A-11R-2016-07 | 2 | 4 | 164 | Primary solid Tumor |
| TCGA-CV-7102-01A-11R-2016-07 | 2 | 4 | 164 | Primary solid Tumor |
| TCGA-CV-7103-01A-21R-2016-07 | 2 | 4 | 164 | Primary solid Tumor |
| TCGA-CV-7104-01A-11R-2016-07 | 2 | 4 | 164 | Primary solid Tumor |
| TCGA-CV-7177-01A-11R-2016-07 | 2 | 4 | 164 | Primary solid Tumor |
| TCGA-CV-7178-01A-21R-2016-07 | 2 | 4 | 164 | Primary solid Tumor |
| TCGA-CV-7180-01A-11R-2016-07 | 2 | 4 | 164 | Primary solid Tumor |
| TCGA-CV-7183-01A-11R-2016-07 | 2 | 4 | 164 | Primary solid Tumor |
| TCGA-CV-7235-01A-11R-2016-07 | 2 | 4 | 164 | Primary solid Tumor |
| TCGA-CV-7236-01A-11R-2016-07 | 2 | 4 | 164 | Primary solid Tumor |
| TCGA-CV-7238-01A-11R-2016-07 | 2 | 4 | 164 | Primary solid Tumor |
| TCGA-CV-7242-01A-11R-2016-07 | 2 | 4 | 164 | Primary solid Tumor |
| TCGA-CV-7243-01A-11R-2016-07 | 2 | 4 | 164 | Primary solid Tumor |
| TCGA-CV-7245-01A-11R-2016-07 | 2 | 4 | 164 | Primary solid Tumor |
| TCGA-CV-7247-01A-11R-2016-07 | 2 | 4 | 164 | Primary solid Tumor |
| TCGA-CV-7248-01A-11R-2016-07 | 2 | 4 | 164 | Primary solid Tumor |
| TCGA-CV-7250-01A-11R-2016-07 | 2 | 4 | 164 | Primary solid Tumor |
| TCGA-CV-7252-01A-11R-2016-07 | 2 | 4 | 164 | Primary solid Tumor |
| TCGA-CV-7253-01A-11R-2016-07 | 2 | 4 | 164 | Primary solid Tumor |
| TCGA-CV-7254-01A-11R-2016-07 | 2 | 4 | 164 | Primary solid Tumor |
| TCGA-CV-7255-01A-11R-2016-07 | 2 | 4 | 164 | Primary solid Tumor |
| TCGA-CV-7261-01A-11R-2016-07 | 2 | 4 | 164 | Primary solid Tumor |
| TCGA-CV-7263-01A-11R-2016-07 | 2 | 4 | 164 | Primary solid Tumor |
| TCGA-CV-7406-01A-11R-2081-07 | 2 | 4 | 188 | Primary solid Tumor |
| TCGA-CV-7407-01A-11R-2081-07 | 2 | 4 | 188 | Primary solid Tumor |
| TCGA-CV-7409-01A-31R-2232-07 | 2 | 4 | 215 | Primary solid Tumor |
| TCGA-CV-7410-01A-21R-2081-07 | 2 | 4 | 188 | Primary solid Tumor |
| TCGA-CV-7411-01A-11R-2081-07 | 2 | 4 | 188 | Primary solid Tumor |
| TCGA-CV-7413-01A-11R-2081-07 | 2 | 4 | 188 | Primary solid Tumor |
| TCGA-CV-7414-01A-11R-2081-07 | 2 | 4 | 188 | Primary solid Tumor |
| TCGA-CV-7415-01A-11R-2081-07 | 2 | 4 | 188 | Primary solid Tumor |
| TCGA-CV-7416-01A-11R-2081-07 | 2 | 4 | 188 | Primary solid Tumor |
| TCGA-CV-7418-01A-11R-2081-07 | 2 | 4 | 188 | Primary solid Tumor |
| TCGA-CV-7421-01A-11R-2081-07 | 2 | 4 | 188 | Primary solid Tumor |
| TCGA-CV-7422-01A-21R-2081-07 | 2 | 4 | 188 | Primary solid Tumor |
| TCGA-CV-7423-01A-11R-2081-07 | 2 | 4 | 188 | Primary solid Tumor |
| TCGA-CV-7424-01A-11R-2081-07 | 2 | 4 | 188 | Primary solid Tumor |
| TCGA-CV-7425-01A-11R-2081-07 | 2 | NA | 188 | Primary solid Tumor |
| TCGA-CV-7427-01A-11R-2081-07 | 2 | 4 | 188 | Primary solid Tumor |
| TCGA-CV-7428-01A-11R-2132-07 | 2 | 4 | 190 | Primary solid Tumor |
| TCGA-CV-7429-01A-11R-2132-07 | 2 | 4 | 190 | Primary solid Tumor |
| TCGA-CV-7430-01A-11R-2132-07 | 2 | 4 | 190 | Primary solid Tumor |
| TCGA-CV-7432-01A-11R-2132-07 | 2 | 4 | 190 | Primary solid Tumor |
| TCGA-CV-7433-01A-11R-2132-07 | 2 | 4 | 190 | Primary solid Tumor |
| TCGA-CV-7434-01A-11R-2132-07 | 2 | 4 | 190 | Primary solid Tumor |
| TCGA-CV-7435-01A-11R-2132-07 | 2 | 4 | 190 | Primary solid Tumor |
| TCGA-CV-7437-01A-21R-2132-07 | 2 | 4 | 190 | Primary solid Tumor |
| TCGA-CV-7438-01A-21R-2132-07 | 2 | 4 | 190 | Primary solid Tumor |
| TCGA-CV-7440-01A-11R-2132-07 | 2 | 4 | 190 | Primary solid Tumor |
| TCGA-CV-7446-01A-11R-2232-07 | 2 | NA | 190 | Primary solid Tumor |
| TCGA-CV-7568-01A-11R-2232-07 | 2 | NA | 190 | Primary solid Tumor |
| TCGA-CV-A45O-01A-21R-A24H-07 | 2 | NA | 260 | Primary solid Tumor |
| TCGA-CV-A45P-01A-11R-A24H-07 | 2 | NA | 260 | Primary solid Tumor |
| TCGA-CV-A45Q-01A-11R-A24H-07 | 2 | NA | 260 | Primary solid Tumor |
| TCGA-CV-A45R-01A-11R-A24H-07 | 2 | NA | 260 | Primary solid Tumor |
| TCGA-CV-A45T-01A-11R-A24H-07 | 2 | NA | 260 | Primary solid Tumor |
| TCGA-CV-A45U-01A-12R-A24H-07 | 2 | NA | 260 | Primary solid Tumor |
| TCGA-CV-A45V-01A-21R-A24Z-07 | 2 | NA | 265 | Primary solid Tumor |
| TCGA-CV-A45W-01A-11R-A24Z-07 | 2 | NA | 265 | Primary solid Tumor |
| TCGA-CV-A45X-01A-21R-A24Z-07 | 2 | NA | 265 | Primary solid Tumor |
| TCGA-CV-A45Y-01A-11R-A24Z-07 | 2 | NA | 265 | Primary solid Tumor |
| TCGA-CV-A45Z-01A-21R-A24Z-07 | 2 | NA | 265 | Primary solid Tumor |
| TCGA-CV-A460-01A-21R-A24Z-07 | 2 | NA | 265 | Primary solid Tumor |
| TCGA-CV-A461-01A-41R-A266-07 | 2 | NA | 273 | Primary solid Tumor |
| TCGA-CV-A463-01A-11R-A266-07 | 2 | NA | 273 | Primary solid Tumor |
| TCGA-CV-A464-01A-11R-A266-07 | 2 | NA | 273 | Primary solid Tumor |
| TCGA-CV-A465-01A-11R-A266-07 | 2 | NA | 273 | Primary solid Tumor |
| TCGA-CV-A468-01A-11R-A266-07 | 2 | NA | 273 | Primary solid Tumor |
| TCGA-CV-A6JD-01A-11R-A31N-07 | 2 | NA | 318 | Primary solid Tumor |
| TCGA-CV-A6JE-01A-11R-A31N-07 | 2 | NA | 318 | Primary solid Tumor |
| TCGA-CV-A6JM-01A-11R-A31N-07 | 2 | NA | 318 | Primary solid Tumor |
| TCGA-CV-A6JN-01A-11R-A31N-07 | 2 | NA | 318 | Primary solid Tumor |
| TCGA-CV-A6JO-01B-11R-A34R-07 | 2 | NA | 355 | Primary solid Tumor |
| TCGA-CV-A6JT-01A-11R-A31N-07 | 2 | NA | 318 | Primary solid Tumor |
| TCGA-CV-A6JU-01A-11R-A31N-07 | 2 | NA | 318 | Primary solid Tumor |
| TCGA-CV-A6JY-01A-11R-A31N-07 | 2 | NA | 318 | Primary solid Tumor |
| TCGA-CV-A6JZ-01A-11R-A31N-07 | 2 | NA | 318 | Primary solid Tumor |
| TCGA-CV-A6K0-01B-21R-A31N-07 | 2 | NA | 318 | Primary solid Tumor |
| TCGA-CV-A6K1-01A-11R-A31N-07 | 2 | NA | 318 | Primary solid Tumor |
| TCGA-CV-A6K2-01A-11R-A31N-07 | 2 | NA | 318 | Primary solid Tumor |
| TCGA-CW-5580-01A-01R-1672-07 | 14 | 9 | 105 | Primary solid Tumor |
| TCGA-CW-5581-01A-02R-1541-07 | 14 | 9 | 90 | Primary solid Tumor |
| TCGA-CW-5583-01A-02R-1541-07 | 14 | 9 | 90 | Primary solid Tumor |
| TCGA-CW-5584-01A-01R-1541-07 | 14 | 9 | 90 | Primary solid Tumor |
| TCGA-CW-5585-01A-01R-1541-07 | 14 | 9 | 90 | Primary solid Tumor |
| TCGA-CW-5587-01A-01R-1541-07 | 14 | 9 | 90 | Primary solid Tumor |
| TCGA-CW-5588-01A-01R-1541-07 | 14 | 9 | 90 | Primary solid Tumor |
| TCGA-CW-5589-01A-01R-1541-07 | 14 | 9 | 90 | Primary solid Tumor |
| TCGA-CW-5590-01A-01R-1541-07 | 14 | 9 | 90 | Primary solid Tumor |
| TCGA-CW-5591-01A-01R-1541-07 | 14 | 9 | 90 | Primary solid Tumor |
| TCGA-CW-6087-01A-11R-1672-07 | 14 | 9 | 105 | Primary solid Tumor |
| TCGA-CW-6088-01A-11R-1672-07 | 14 | 9 | 105 | Primary solid Tumor |
| TCGA-CW-6090-01A-11R-1672-07 | 14 | 9 | 105 | Primary solid Tumor |
| TCGA-CW-6093-01A-11R-1672-07 | 14 | 9 | 105 | Primary solid Tumor |
| TCGA-CW-6097-01A-11R-1672-07 | 14 | 9 | 105 | Primary solid Tumor |
| TCGA-CX-7082-01A-11R-2016-07 | 2 | 4 | 164 | Primary solid Tumor |
| TCGA-CX-7085-01A-21R-2016-07 | 2 | 4 | 164 | Primary solid Tumor |
| TCGA-CX-7086-01A-11R-2081-07 | 2 | 4 | 188 | Primary solid Tumor |
| TCGA-CX-7219-01A-11R-2016-07 | 2 | 4 | 164 | Primary solid Tumor |
| TCGA-CX-A4AQ-01A-11R-A24Z-07 | 2 | NA | 265 | Primary solid Tumor |
| TCGA-CZ-4853-01A-01R-1426-07 | 14 | 9 | 70 | Primary solid Tumor |
| TCGA-CZ-4854-01A-01R-1305-07 | 14 | 9 | 65 | Primary solid Tumor |
| TCGA-CZ-4856-01A-02R-1426-07 | 14 | 9 | 70 | Primary solid Tumor |
| TCGA-CZ-4857-01A-01R-1305-07 | 14 | 9 | 65 | Primary solid Tumor |
| TCGA-CZ-4858-01A-01R-1305-07 | 14 | 9 | 65 | Primary solid Tumor |
| TCGA-CZ-4859-01A-02R-1426-07 | 14 | 9 | 70 | Primary solid Tumor |
| TCGA-CZ-4860-01A-01R-1305-07 | 14 | 9 | 65 | Primary solid Tumor |
| TCGA-CZ-4861-01A-01R-1305-07 | 14 | 9 | 65 | Primary solid Tumor |
| TCGA-CZ-4862-01A-01R-1305-07 | 14 | 9 | 65 | Primary solid Tumor |
| TCGA-CZ-4863-01A-01R-1503-07 | 14 | 9 | 82 | Primary solid Tumor |
| TCGA-CZ-4864-01A-01R-1503-07 | 14 | 9 | 82 | Primary solid Tumor |
| TCGA-CZ-4865-01A-02R-1503-07 | 14 | 9 | 82 | Primary solid Tumor |
| TCGA-CZ-4866-01A-01R-1503-07 | 14 | 9 | 82 | Primary solid Tumor |
| TCGA-CZ-5451-01A-01R-1503-07 | 14 | NA | 82 | Primary solid Tumor |
| TCGA-CZ-5452-01A-01R-1503-07 | 14 | 9 | 82 | Primary solid Tumor |
| TCGA-CZ-5453-01A-01R-1503-07 | 14 | 9 | 82 | Primary solid Tumor |
| TCGA-CZ-5454-01A-01R-1503-07 | 14 | 9 | 82 | Primary solid Tumor |
| TCGA-CZ-5455-01A-01R-1503-07 | 14 | 9 | 82 | Primary solid Tumor |
| TCGA-CZ-5456-01A-01R-1503-07 | 14 | 9 | 82 | Primary solid Tumor |
| TCGA-CZ-5457-01A-01R-1503-07 | 14 | 9 | 82 | Primary solid Tumor |
| TCGA-CZ-5458-01A-01R-1503-07 | 14 | 9 | 82 | Primary solid Tumor |
| TCGA-CZ-5459-01A-01R-1503-07 | 14 | NA | 82 | Primary solid Tumor |
| TCGA-CZ-5460-01A-01R-1503-07 | 14 | 9 | 82 | Primary solid Tumor |
| TCGA-CZ-5461-01A-01R-1503-07 | 14 | 9 | 82 | Primary solid Tumor |
| TCGA-CZ-5462-01A-01R-1503-07 | 14 | 9 | 82 | Primary solid Tumor |
| TCGA-CZ-5463-01A-01R-1503-07 | 14 | 9 | 82 | Primary solid Tumor |
| TCGA-CZ-5464-01A-01R-1503-07 | 14 | 9 | 82 | Primary solid Tumor |
| TCGA-CZ-5465-01A-01R-1503-07 | 14 | 9 | 82 | Primary solid Tumor |
| TCGA-CZ-5466-01A-01R-1503-07 | 14 | NA | 82 | Primary solid Tumor |
| TCGA-CZ-5467-01A-01R-1503-07 | 14 | 9 | 82 | Primary solid Tumor |
| TCGA-CZ-5468-01A-01R-1503-07 | 14 | 9 | 82 | Primary solid Tumor |
| TCGA-CZ-5469-01A-01R-1503-07 | 14 | 9 | 82 | Primary solid Tumor |
| TCGA-CZ-5470-01A-01R-1503-07 | 14 | 9 | 82 | Primary solid Tumor |
| TCGA-CZ-5982-01A-11R-1672-07 | 14 | 9 | 105 | Primary solid Tumor |
| TCGA-CZ-5984-01A-11R-1672-07 | 14 | 9 | 105 | Primary solid Tumor |
| TCGA-CZ-5985-01A-11R-1672-07 | 14 | 9 | 105 | Primary solid Tumor |
| TCGA-CZ-5986-01A-11R-1672-07 | 14 | 9 | 105 | Primary solid Tumor |
| TCGA-CZ-5987-01A-11R-1672-07 | 14 | 9 | 105 | Primary solid Tumor |
| TCGA-CZ-5988-01A-11R-1672-07 | 14 | 9 | 105 | Primary solid Tumor |
| TCGA-CZ-5989-01A-11R-1672-07 | 14 | 9 | 105 | Primary solid Tumor |
| TCGA-D1-A0ZN-01A-11R-A118-07 | 16 | 11 | 92 | Primary solid Tumor |
| TCGA-D1-A0ZO-01A-11R-A118-07 | 16 | 11 | 92 | Primary solid Tumor |
| TCGA-D1-A0ZP-01A-21R-A10J-07 | 16 | 11 | 81 | Primary solid Tumor |
| TCGA-D1-A0ZQ-01A-11R-A118-07 | 16 | 11 | 92 | Primary solid Tumor |
| TCGA-D1-A0ZR-01A-21R-A10J-07 | 16 | 11 | 81 | Primary solid Tumor |
| TCGA-D1-A0ZS-01A-11R-A118-07 | 13 | 6 | 92 | Primary solid Tumor |
| TCGA-D1-A0ZU-01A-11R-A10J-07 | 16 | 11 | 81 | Primary solid Tumor |
| TCGA-D1-A0ZV-01A-11R-A10J-07 | 16 | 11 | 81 | Primary solid Tumor |
| TCGA-D1-A0ZZ-01A-11R-A10J-07 | 16 | 11 | 81 | Primary solid Tumor |
| TCGA-D1-A101-01A-12R-A10J-07 | 16 | 11 | 81 | Primary solid Tumor |
| TCGA-D1-A102-01A-11R-A10J-07 | 16 | 11 | 81 | Primary solid Tumor |
| TCGA-D1-A103-01A-11R-A10J-07 | 16 | 11 | 81 | Primary solid Tumor |
| TCGA-D1-A15V-01A-11R-A118-07 | 16 | 11 | 92 | Primary solid Tumor |
| TCGA-D1-A15W-01A-11R-A118-07 | 16 | 11 | 92 | Primary solid Tumor |
| TCGA-D1-A15X-01A-11R-A118-07 | 16 | 11 | 92 | Primary solid Tumor |
| TCGA-D1-A15Z-01A-11R-A118-07 | 16 | 11 | 92 | Primary solid Tumor |
| TCGA-D1-A160-01A-11R-A118-07 | 16 | 11 | 92 | Primary solid Tumor |
| TCGA-D1-A161-01A-11R-A118-07 | 16 | 11 | 92 | Primary solid Tumor |
| TCGA-D1-A162-01A-11R-A118-07 | 16 | 11 | 92 | Primary solid Tumor |
| TCGA-D1-A163-01A-11R-A12I-07 | 16 | 11 | 94 | Primary solid Tumor |
| TCGA-D1-A165-01A-11R-A12I-07 | 16 | 11 | 94 | Primary solid Tumor |
| TCGA-D1-A167-01A-11R-A12I-07 | 16 | 11 | 94 | Primary solid Tumor |
| TCGA-D1-A168-01A-31R-A12I-07 | 13 | 1 | 94 | Primary solid Tumor |
| TCGA-D1-A169-01A-11R-A12I-07 | 16 | 11 | 94 | Primary solid Tumor |
| TCGA-D1-A16B-01A-11R-A12I-07 | 16 | 11 | 94 | Primary solid Tumor |
| TCGA-D1-A16D-01A-11R-A12I-07 | 16 | 11 | 94 | Primary solid Tumor |
| TCGA-D1-A16E-01A-22R-A12I-07 | 16 | 11 | 94 | Primary solid Tumor |
| TCGA-D1-A16F-01A-11R-A12I-07 | 16 | 11 | 94 | Primary solid Tumor |
| TCGA-D1-A16G-01A-31R-A12I-07 | 16 | 15 | 94 | Primary solid Tumor |
| TCGA-D1-A16I-01A-11R-A12I-07 | 16 | 11 | 94 | Primary solid Tumor |
| TCGA-D1-A16J-01A-11R-A12I-07 | 16 | 11 | 94 | Primary solid Tumor |
| TCGA-D1-A16N-01A-11R-A12I-07 | 16 | 11 | 94 | Primary solid Tumor |
| TCGA-D1-A16O-01A-11R-A12I-07 | 16 | 11 | 94 | Primary solid Tumor |
| TCGA-D1-A16Q-01A-12R-A12I-07 | 16 | 11 | 94 | Primary solid Tumor |
| TCGA-D1-A16R-01A-11R-A12I-07 | 16 | 11 | 94 | Primary solid Tumor |
| TCGA-D1-A16S-01A-11R-A12I-07 | 16 | 11 | 94 | Primary solid Tumor |
| TCGA-D1-A16V-01A-11R-A12I-07 | 16 | 11 | 94 | Primary solid Tumor |
| TCGA-D1-A16X-01A-11R-A12I-07 | 16 | 11 | 94 | Primary solid Tumor |
| TCGA-D1-A16Y-01A-31R-A12I-07 | 16 | 11 | 94 | Primary solid Tumor |
| TCGA-D1-A174-01A-11R-A12I-07 | 16 | 11 | 94 | Primary solid Tumor |
| TCGA-D1-A175-01A-11R-A12I-07 | 16 | 11 | 94 | Primary solid Tumor |
| TCGA-D1-A176-01A-11R-A12I-07 | 16 | 11 | 94 | Primary solid Tumor |
| TCGA-D1-A177-01A-21R-A12I-07 | 16 | 11 | 94 | Primary solid Tumor |
| TCGA-D1-A179-01A-11R-A12I-07 | 16 | 11 | 94 | Primary solid Tumor |
| TCGA-D1-A17A-01A-11R-A12I-07 | 16 | 11 | 94 | Primary solid Tumor |
| TCGA-D1-A17B-01A-22R-A12I-07 | 16 | 11 | 94 | Primary solid Tumor |
| TCGA-D1-A17C-01A-11R-A12I-07 | 16 | 11 | 94 | Primary solid Tumor |
| TCGA-D1-A17D-01A-12R-A12I-07 | 16 | 11 | 94 | Primary solid Tumor |
| TCGA-D1-A17F-01A-11R-A12I-07 | 16 | 11 | 94 | Primary solid Tumor |
| TCGA-D1-A17H-01A-11R-A12I-07 | 16 | 11 | 94 | Primary solid Tumor |
| TCGA-D1-A17K-01A-11R-A12I-07 | 16 | 11 | 94 | Primary solid Tumor |
| TCGA-D1-A17L-01A-11R-A12I-07 | 16 | 11 | 94 | Primary solid Tumor |
| TCGA-D1-A17M-01A-21R-A12I-07 | 16 | 11 | 94 | Primary solid Tumor |
| TCGA-D1-A17N-01A-11R-A12I-07 | 16 | 11 | 94 | Primary solid Tumor |
| TCGA-D1-A17Q-01A-11R-A12I-07 | 16 | 11 | 94 | Primary solid Tumor |
| TCGA-D1-A17R-01A-11R-A12I-07 | 16 | 11 | 94 | Primary solid Tumor |
| TCGA-D1-A17S-01A-11R-A12I-07 | 16 | 11 | 94 | Primary solid Tumor |
| TCGA-D1-A17T-01A-11R-A12I-07 | 16 | 11 | 94 | Primary solid Tumor |
| TCGA-D1-A17U-01A-21R-A12I-07 | 16 | 11 | 94 | Primary solid Tumor |
| TCGA-D1-A1NS-01A-11R-A14D-07 | 16 | 11 | 121 | Primary solid Tumor |
| TCGA-D1-A1NU-01A-11R-A14D-07 | 16 | 11 | 121 | Primary solid Tumor |
| TCGA-D1-A1NW-01A-11R-A14M-07 | 16 | 11 | 125 | Primary solid Tumor |
| TCGA-D1-A1NX-01A-11R-A16F-07 | 16 | 11 | 143 | Primary solid Tumor |
| TCGA-D1-A1NY-01A-11R-A16F-07 | 16 | 11 | 143 | Primary solid Tumor |
| TCGA-D1-A1NZ-01A-21R-A14D-07 | 16 | 11 | 121 | Primary solid Tumor |
| TCGA-D1-A1O0-01A-11R-A17B-07 | 16 | 11 | 156 | Primary solid Tumor |
| TCGA-D1-A1O5-01A-11R-A14D-07 | 16 | 11 | 121 | Primary solid Tumor |
| TCGA-D1-A1O7-01A-11R-A14D-07 | 16 | 11 | 121 | Primary solid Tumor |
| TCGA-D1-A1O8-01A-11R-A14D-07 | 4 | 3 | 121 | Primary solid Tumor |
| TCGA-D1-A2G0-01A-11R-A32Y-07 | 16 | NA | 156 | Primary solid Tumor |
| TCGA-D1-A2G5-01A-11R-A17B-07 | 16 | 11 | 156 | Primary solid Tumor |
| TCGA-D1-A2G6-01A-11R-A17B-07 | 16 | 11 | 156 | Primary solid Tumor |
| TCGA-D1-A2G7-01A-21R-A180-07 | 16 | 11 | 168 | Primary solid Tumor |
| TCGA-D1-A3DA-01A-12R-A213-07 | 16 | NA | 201 | Primary solid Tumor |
| TCGA-D1-A3DG-01A-11R-A19W-07 | 16 | NA | 186 | Primary solid Tumor |
| TCGA-D1-A3DH-01A-11R-A19W-07 | 16 | NA | 186 | Primary solid Tumor |
| TCGA-D1-A3JP-01A-31R-A22K-07 | 16 | NA | 228 | Primary solid Tumor |
| TCGA-D1-A3JQ-01A-11R-A22K-07 | 16 | NA | 228 | Primary solid Tumor |
| TCGA-D5-5537-01A-21R-1928-07 | 7 | NA | 157 | Primary solid Tumor |
| TCGA-D5-5538-01A-01R-1653-07 | 7 | NA | 89 | Primary solid Tumor |
| TCGA-D5-5539-01A-01R-1653-07 | 7 | NA | 89 | Primary solid Tumor |
| TCGA-D5-5540-01A-01R-1653-07 | 7 | NA | 89 | Primary solid Tumor |
| TCGA-D5-5541-01A-01R-1653-07 | 7 | NA | 89 | Primary solid Tumor |
| TCGA-D5-6529-01A-11R-1774-07 | 7 | NA | 123 | Primary solid Tumor |
| TCGA-D5-6530-01A-11R-1723-07 | 7 | NA | 116 | Primary solid Tumor |
| TCGA-D5-6531-01A-11R-1723-07 | 7 | NA | 116 | Primary solid Tumor |
| TCGA-D5-6532-01A-11R-1723-07 | 7 | NA | 116 | Primary solid Tumor |
| TCGA-D5-6533-01A-11R-1723-07 | 7 | NA | 116 | Primary solid Tumor |
| TCGA-D5-6534-01A-21R-1928-07 | 7 | NA | 157 | Primary solid Tumor |
| TCGA-D5-6535-01A-11R-1723-07 | 7 | NA | 116 | Primary solid Tumor |
| TCGA-D5-6536-01A-11R-1723-07 | 7 | NA | 116 | Primary solid Tumor |
| TCGA-D5-6537-01A-11R-1723-07 | 7 | NA | 116 | Primary solid Tumor |
| TCGA-D5-6538-01A-11R-1723-07 | 7 | NA | 116 | Primary solid Tumor |
| TCGA-D5-6539-01A-11R-1723-07 | 7 | NA | 116 | Primary solid Tumor |
| TCGA-D5-6540-01A-11R-1723-07 | 7 | NA | 116 | Primary solid Tumor |
| TCGA-D5-6541-01A-11R-1723-07 | 7 | NA | 116 | Primary solid Tumor |
| TCGA-D5-6898-01A-11R-1928-07 | 7 | NA | 157 | Primary solid Tumor |
| TCGA-D5-6920-01A-11R-1928-07 | 7 | NA | 157 | Primary solid Tumor |
| TCGA-D5-6922-01A-11R-1928-07 | 7 | NA | 157 | Primary solid Tumor |
| TCGA-D5-6923-01A-11R-A32Z-07 | 7 | NA | 157 | Primary solid Tumor |
| TCGA-D5-6924-01A-11R-1928-07 | 7 | NA | 157 | Primary solid Tumor |
| TCGA-D5-6926-01A-11R-1928-07 | 7 | NA | 157 | Primary solid Tumor |
| TCGA-D5-6927-01A-21R-1928-07 | 7 | NA | 157 | Primary solid Tumor |
| TCGA-D5-6928-01A-11R-1928-07 | 7 | NA | 157 | Primary solid Tumor |
| TCGA-D5-6929-01A-31R-1928-07 | 7 | NA | 157 | Primary solid Tumor |
| TCGA-D5-6930-01A-11R-1928-07 | 7 | NA | 157 | Primary solid Tumor |
| TCGA-D5-6931-01A-11R-1928-07 | 7 | NA | 157 | Primary solid Tumor |
| TCGA-D5-6932-01A-11R-1928-07 | 7 | NA | 157 | Primary solid Tumor |
| TCGA-D5-7000-01A-11R-A32Z-07 | 7 | NA | 157 | Primary solid Tumor |
| TCGA-D6-6515-01A-21R-1873-07 | 2 | 4 | 145 | Primary solid Tumor |
| TCGA-D6-6516-01A-11R-1873-07 | 2 | 4 | 145 | Primary solid Tumor |
| TCGA-D6-6517-01A-11R-1873-07 | 2 | 4 | 145 | Primary solid Tumor |
| TCGA-D6-6823-01A-11R-1915-07 | 2 | 4 | 151 | Primary solid Tumor |
| TCGA-D6-6824-01A-11R-1915-07 | 2 | 4 | 151 | Primary solid Tumor |
| TCGA-D6-6825-01A-21R-1915-07 | 2 | 4 | 151 | Primary solid Tumor |
| TCGA-D6-6826-01A-11R-1915-07 | 2 | 4 | 151 | Primary solid Tumor |
| TCGA-D6-6827-01A-11R-1915-07 | 2 | NA | 151 | Primary solid Tumor |
| TCGA-D6-8568-01A-11R-2403-07 | 2 | NA | 241 | Primary solid Tumor |
| TCGA-D6-8569-01A-11R-2403-07 | 2 | NA | 241 | Primary solid Tumor |
| TCGA-D6-A4Z9-01A-11R-A24Z-07 | 2 | NA | 265 | Primary solid Tumor |
| TCGA-D6-A4ZB-01A-11R-A24Z-07 | 2 | NA | 265 | Primary solid Tumor |
| TCGA-D6-A6EK-01A-11R-A31N-07 | 2 | NA | 318 | Primary solid Tumor |
| TCGA-D6-A6EM-01A-21R-A31N-07 | 2 | NA | 318 | Primary solid Tumor |
| TCGA-D6-A6EN-01A-11R-A31N-07 | 2 | NA | 318 | Primary solid Tumor |
| TCGA-D6-A6EO-01A-11R-A31N-07 | 2 | NA | 318 | Primary solid Tumor |
| TCGA-D6-A6EP-01A-11R-A31N-07 | 3 | NA | 318 | Primary solid Tumor |
| TCGA-D6-A6EQ-01A-11R-A31N-07 | 2 | NA | 318 | Primary solid Tumor |
| TCGA-D6-A6ES-01A-12R-A31N-07 | 2 | NA | 318 | Primary solid Tumor |
| TCGA-D6-A74Q-01A-11R-A34R-07 | 2 | NA | 355 | Primary solid Tumor |
| TCGA-D8-A13Y-01A-11R-A115-07 | 3 | 6 | 85 | Primary solid Tumor |
| TCGA-D8-A13Z-01A-11R-A115-07 | 9 | 7 | 85 | Primary solid Tumor |
| TCGA-D8-A140-01A-11R-A115-07 | 9 | 7 | 85 | Primary solid Tumor |
| TCGA-D8-A141-01A-11R-A115-07 | 9 | 7 | 85 | Primary solid Tumor |
| TCGA-D8-A142-01A-11R-A115-07 | 9 | 8 | 85 | Primary solid Tumor |
| TCGA-D8-A143-01A-11R-A115-07 | 9 | 7 | 85 | Primary solid Tumor |
| TCGA-D8-A145-01A-11R-A115-07 | 9 | 7 | 85 | Primary solid Tumor |
| TCGA-D8-A146-01A-31R-A115-07 | 9 | 7 | 85 | Primary solid Tumor |
| TCGA-D8-A147-01A-11R-A115-07 | 10 | 8 | 85 | Primary solid Tumor |
| TCGA-D8-A1J8-01A-11R-A13Q-07 | 9 | 7 | 109 | Primary solid Tumor |
| TCGA-D8-A1J9-01A-11R-A13Q-07 | 9 | 7 | 109 | Primary solid Tumor |
| TCGA-D8-A1JA-01A-11R-A13Q-07 | 9 | 7 | 109 | Primary solid Tumor |
| TCGA-D8-A1JB-01A-11R-A13Q-07 | 9 | 7 | 109 | Primary solid Tumor |
| TCGA-D8-A1JC-01A-11R-A13Q-07 | 9 | 7 | 109 | Primary solid Tumor |
| TCGA-D8-A1JD-01A-11R-A13Q-07 | 9 | 7 | 109 | Primary solid Tumor |
| TCGA-D8-A1JE-01A-11R-A13Q-07 | 9 | 7 | 109 | Primary solid Tumor |
| TCGA-D8-A1JF-01A-11R-A13Q-07 | 9 | 7 | 109 | Primary solid Tumor |
| TCGA-D8-A1JG-01B-11R-A13Q-07 | 9 | 7 | 109 | Primary solid Tumor |
| TCGA-D8-A1JH-01A-11R-A13Q-07 | 9 | 7 | 109 | Primary solid Tumor |
| TCGA-D8-A1JI-01A-11R-A13Q-07 | 9 | 7 | 109 | Primary solid Tumor |
| TCGA-D8-A1JJ-01A-31R-A14M-07 | 9 | 7 | 124 | Primary solid Tumor |
| TCGA-D8-A1JK-01A-11R-A13Q-07 | 9 | 7 | 109 | Primary solid Tumor |
| TCGA-D8-A1JL-01A-11R-A13Q-07 | 10 | 8 | 109 | Primary solid Tumor |
| TCGA-D8-A1JM-01A-11R-A13Q-07 | 10 | 8 | 109 | Primary solid Tumor |
| TCGA-D8-A1JN-01A-11R-A13Q-07 | 9 | 7 | 109 | Primary solid Tumor |
| TCGA-D8-A1JP-01A-11R-A13Q-07 | 9 | 7 | 109 | Primary solid Tumor |
| TCGA-D8-A1JS-01A-11R-A13Q-07 | 9 | 7 | 109 | Primary solid Tumor |
| TCGA-D8-A1JT-01A-31R-A13Q-07 | 9 | 7 | 109 | Primary solid Tumor |
| TCGA-D8-A1JU-01A-11R-A13Q-07 | 9 | 7 | 109 | Primary solid Tumor |
| TCGA-D8-A1X5-01A-11R-A14D-07 | 9 | 7 | 120 | Primary solid Tumor |
| TCGA-D8-A1X6-01A-11R-A14M-07 | 9 | 7 | 124 | Primary solid Tumor |
| TCGA-D8-A1X7-01A-11R-A14M-07 | 9 | 7 | 124 | Primary solid Tumor |
| TCGA-D8-A1X8-01A-11R-A14M-07 | 9 | 7 | 124 | Primary solid Tumor |
| TCGA-D8-A1X9-01A-12R-A157-07 | 9 | 7 | 136 | Primary solid Tumor |
| TCGA-D8-A1XA-01A-11R-A14D-07 | 9 | 7 | 120 | Primary solid Tumor |
| TCGA-D8-A1XB-01A-11R-A14D-07 | 9 | 7 | 120 | Primary solid Tumor |
| TCGA-D8-A1XC-01A-11R-A14D-07 | 9 | 7 | 120 | Primary solid Tumor |
| TCGA-D8-A1XD-01A-11R-A14D-07 | 9 | 7 | 120 | Primary solid Tumor |
| TCGA-D8-A1XF-01A-11R-A14D-07 | 9 | 7 | 120 | Primary solid Tumor |
| TCGA-D8-A1XG-01A-11R-A14D-07 | 9 | 7 | 120 | Primary solid Tumor |
| TCGA-D8-A1XJ-01A-11R-A14M-07 | 9 | 7 | 124 | Primary solid Tumor |
| TCGA-D8-A1XK-01A-21R-A14M-07 | 10 | 8 | 124 | Primary solid Tumor |
| TCGA-D8-A1XL-01A-11R-A14M-07 | 9 | 7 | 124 | Primary solid Tumor |
| TCGA-D8-A1XM-01A-21R-A14M-07 | 9 | 7 | 124 | Primary solid Tumor |
| TCGA-D8-A1XO-01A-11R-A14M-07 | 9 | 7 | 124 | Primary solid Tumor |
| TCGA-D8-A1XQ-01A-11R-A14M-07 | 10 | 8 | 124 | Primary solid Tumor |
| TCGA-D8-A1XR-01A-11R-A14M-07 | 9 | 7 | 124 | Primary solid Tumor |
| TCGA-D8-A1XS-01A-11R-A14M-07 | 9 | 7 | 124 | Primary solid Tumor |
| TCGA-D8-A1XT-01A-11R-A14M-07 | 9 | 7 | 124 | Primary solid Tumor |
| TCGA-D8-A1XU-01A-11R-A14M-07 | 9 | 7 | 124 | Primary solid Tumor |
| TCGA-D8-A1XV-01A-11R-A14M-07 | 9 | 7 | 124 | Primary solid Tumor |
| TCGA-D8-A1XW-01A-11R-A14M-07 | 10 | 8 | 124 | Primary solid Tumor |
| TCGA-D8-A1XY-01A-11R-A14M-07 | 9 | 7 | 124 | Primary solid Tumor |
| TCGA-D8-A1XZ-01A-11R-A14M-07 | 9 | 7 | 124 | Primary solid Tumor |
| TCGA-D8-A1Y0-01A-11R-A14M-07 | 9 | 7 | 124 | Primary solid Tumor |
| TCGA-D8-A1Y1-01A-21R-A14M-07 | 9 | 7 | 124 | Primary solid Tumor |
| TCGA-D8-A1Y2-01A-11R-A157-07 | 9 | 7 | 136 | Primary solid Tumor |
| TCGA-D8-A1Y3-01A-11R-A157-07 | 9 | 7 | 136 | Primary solid Tumor |
| TCGA-D8-A27E-01A-11R-A16F-07 | 9 | 7 | 147 | Primary solid Tumor |
| TCGA-D8-A27F-01A-11R-A16F-07 | 10 | 8 | 147 | Primary solid Tumor |
| TCGA-D8-A27G-01A-11R-A16F-07 | 9 | 7 | 147 | Primary solid Tumor |
| TCGA-D8-A27H-01A-11R-A16F-07 | 10 | 8 | 147 | Primary solid Tumor |
| TCGA-D8-A27I-01A-11R-A16F-07 | 9 | 7 | 147 | Primary solid Tumor |
| TCGA-D8-A27K-01A-11R-A16F-07 | 9 | 7 | 147 | Primary solid Tumor |
| TCGA-D8-A27L-01A-11R-A16F-07 | 9 | 7 | 147 | Primary solid Tumor |
| TCGA-D8-A27M-01A-11R-A16F-07 | 10 | 8 | 147 | Primary solid Tumor |
| TCGA-D8-A27N-01A-11R-A16F-07 | 9 | 7 | 147 | Primary solid Tumor |
| TCGA-D8-A27P-01A-11R-A16F-07 | 9 | 7 | 147 | Primary solid Tumor |
| TCGA-D8-A27R-01A-11R-A16F-07 | 9 | 7 | 147 | Primary solid Tumor |
| TCGA-D8-A27T-01A-11R-A16F-07 | 9 | 7 | 147 | Primary solid Tumor |
| TCGA-D8-A27V-01A-12R-A17B-07 | 9 | 7 | 155 | Primary solid Tumor |
| TCGA-D8-A27W-01A-11R-A16F-07 | 9 | 7 | 147 | Primary solid Tumor |
| TCGA-D8-A3Z5-01A-41R-A24H-07 | 9 | NA | 255 | Primary solid Tumor |
| TCGA-D8-A3Z6-01A-11R-A239-07 | 9 | NA | 239 | Primary solid Tumor |
| TCGA-D8-A4Z1-01A-21R-A266-07 | 9 | NA | 271 | Primary solid Tumor |
| TCGA-D8-A73U-01A-11R-A33J-07 | 9 | NA | 338 | Primary solid Tumor |
| TCGA-D8-A73W-01A-22R-A352-07 | 9 | NA | 360 | Primary solid Tumor |
| TCGA-D8-A73X-01A-11R-A32P-07 | 9 | NA | 334 | Primary solid Tumor |
| TCGA-DC-4745-01A-01R-A32Z-07 | 7 | NA | 122 | Primary solid Tumor |
| TCGA-DC-4749-01A-01R-1736-07 | 7 | NA | 122 | Primary solid Tumor |
| TCGA-DC-5337-01A-01R-1660-07 | 7 | NA | 102 | Primary solid Tumor |
| TCGA-DC-5869-01A-01R-1660-07 | 7 | NA | 102 | Primary solid Tumor |
| TCGA-DC-6154-01A-31R-1928-07 | 7 | NA | 158 | Primary solid Tumor |
| TCGA-DC-6155-01A-11R-1660-07 | 7 | NA | 102 | Primary solid Tumor |
| TCGA-DC-6156-01A-11R-1660-07 | 7 | NA | 102 | Primary solid Tumor |
| TCGA-DC-6157-01A-11R-1660-07 | 7 | NA | 102 | Primary solid Tumor |
| TCGA-DC-6158-01A-11R-1660-07 | 7 | NA | 102 | Primary solid Tumor |
| TCGA-DC-6160-01A-11R-1660-07 | 7 | NA | 102 | Primary solid Tumor |
| TCGA-DC-6681-01A-11R-A32Z-07 | 7 | NA | 139 | Primary solid Tumor |
| TCGA-DC-6682-01A-11R-1830-07 | 7 | NA | 139 | Primary solid Tumor |
| TCGA-DC-6683-01A-11R-1830-07 | 7 | NA | 139 | Primary solid Tumor |
| TCGA-DF-A2KN-01A-11R-A180-07 | 16 | NA | 168 | Primary solid Tumor |
| TCGA-DF-A2KR-01A-11R-A180-07 | 16 | NA | 168 | Primary solid Tumor |
| TCGA-DF-A2KS-01A-11R-A18M-07 | 16 | 11 | 178 | Primary solid Tumor |
| TCGA-DF-A2KU-01A-11R-A180-07 | 16 | NA | 168 | Primary solid Tumor |
| TCGA-DF-A2KV-01A-11R-A180-07 | 16 | NA | 168 | Primary solid Tumor |
| TCGA-DF-A2KY-01A-21R-A213-07 | 3 | NA | 201 | Primary solid Tumor |
| TCGA-DF-A2KZ-01A-11R-A213-07 | 16 | NA | 201 | Primary solid Tumor |
| TCGA-DF-A2L0-01A-11R-A180-07 | 16 | NA | 168 | Primary solid Tumor |
| TCGA-DI-A0WH-01A-12R-A12I-07 | 16 | 11 | 94 | Primary solid Tumor |
| TCGA-DI-A1BU-01A-11R-A137-07 | 16 | NA | 104 | Primary solid Tumor |
| TCGA-DI-A1BY-01A-21R-A137-07 | 16 | 11 | 104 | Primary solid Tumor |
| TCGA-DI-A1C3-01A-41R-A137-07 | 16 | NA | 104 | Primary solid Tumor |
| TCGA-DI-A1NN-01A-11R-A16F-07 | 20 | 11 | 143 | Primary solid Tumor |
| TCGA-DI-A1NO-01A-31R-A157-07 | 16 | 11 | 137 | Primary solid Tumor |
| TCGA-DI-A2QT-01A-12R-A19W-07 | 16 | NA | 186 | Primary solid Tumor |
| TCGA-DI-A2QU-01A-11R-A18M-07 | 16 | 11 | 178 | Primary solid Tumor |
| TCGA-DI-A2QY-01A-12R-A19W-07 | 16 | NA | 186 | Primary solid Tumor |
| TCGA-DK-A1A3-01A-11R-A13Y-07 | 1 | 1 | 113 | Primary solid Tumor |
| TCGA-DK-A1A5-01A-11R-A13Y-07 | 1 | 1 | 113 | Primary solid Tumor |
| TCGA-DK-A1A6-01A-11R-A13Y-07 | 1 | 1 | 113 | Primary solid Tumor |
| TCGA-DK-A1A7-01A-11R-A13Y-07 | 1 | 1 | 113 | Primary solid Tumor |
| TCGA-DK-A1AA-01A-11R-A13Y-07 | 1 | 1 | 113 | Primary solid Tumor |
| TCGA-DK-A1AB-01A-11R-A13Y-07 | 2 | 4 | 113 | Primary solid Tumor |
| TCGA-DK-A1AC-01A-11R-A13Y-07 | 1 | 1 | 113 | Primary solid Tumor |
| TCGA-DK-A1AD-01A-11R-A13Y-07 | 1 | 1 | 113 | Primary solid Tumor |
| TCGA-DK-A1AE-01A-11R-A13Y-07 | 1 | 4 | 113 | Primary solid Tumor |
| TCGA-DK-A1AF-01A-11R-A13Y-07 | 1 | 1 | 113 | Primary solid Tumor |
| TCGA-DK-A1AG-01A-11R-A13Y-07 | 1 | 1 | 113 | Primary solid Tumor |
| TCGA-DK-A2HX-01A-12R-A18C-07 | 1 | 1 | 175 | Primary solid Tumor |
| TCGA-DK-A2I1-01A-11R-A180-07 | 1 | 1 | 170 | Primary solid Tumor |
| TCGA-DK-A2I2-01A-11R-A180-07 | 2 | 4 | 170 | Primary solid Tumor |
| TCGA-DK-A2I4-01A-11R-A21D-07 | 2 | 2 | 207 | Primary solid Tumor |
| TCGA-DK-A2I6-01A-12R-A18C-07 | 1 | 1 | 175 | Primary solid Tumor |
| TCGA-DK-A3IK-01A-32R-A21D-07 | 1 | 1 | 207 | Primary solid Tumor |
| TCGA-DK-A3IL-01A-11R-A20F-07 | 1 | 1 | 199 | Primary solid Tumor |
| TCGA-DK-A3IM-01A-11R-A20F-07 | 2 | 4 | 199 | Primary solid Tumor |
| TCGA-DK-A3IN-01A-11R-A20F-07 | 1 | 1 | 199 | Primary solid Tumor |
| TCGA-DK-A3IQ-01A-31R-A32Y-07 | 1 | NA | 199 | Primary solid Tumor |
| TCGA-DK-A3IS-01A-21R-A21D-07 | 1 | 1 | 207 | Primary solid Tumor |
| TCGA-DK-A3IT-01A-31R-A20F-07 | 1 | 1 | 199 | Primary solid Tumor |
| TCGA-DK-A3IU-01A-11R-A20F-07 | 5 | 2 | 199 | Primary solid Tumor |
| TCGA-DK-A3IV-01A-22R-A21D-07 | 1 | 1 | 207 | Primary solid Tumor |
| TCGA-DK-A3WW-01A-22R-A23N-07 | 2 | NA | 249 | Primary solid Tumor |
| TCGA-DK-A3WX-01A-22R-A22U-07 | 2 | 4 | 235 | Primary solid Tumor |
| TCGA-DK-A3WY-01A-11R-A22U-07 | 6 | 7 | 235 | Primary solid Tumor |
| TCGA-DK-A3X1-01A-12R-A22U-07 | 1 | 1 | 235 | Primary solid Tumor |
| TCGA-DK-A3X2-01A-11R-A22U-07 | 1 | 1 | 235 | Primary solid Tumor |
| TCGA-DK-A6AV-01A-12R-A30C-07 | 1 | NA | 309 | Primary solid Tumor |
| TCGA-DK-A6AW-01A-11R-A30C-07 | 1 | NA | 309 | Primary solid Tumor |
| TCGA-DK-A6B0-01A-11R-A31N-07 | 1 | NA | 317 | Primary solid Tumor |
| TCGA-DK-A6B1-01A-12R-A30C-07 | 1 | NA | 309 | Primary solid Tumor |
| TCGA-DK-A6B2-01A-11R-A30C-07 | 1 | NA | 309 | Primary solid Tumor |
| TCGA-DK-A6B5-01A-11R-A31N-07 | 1 | NA | 317 | Primary solid Tumor |
| TCGA-DK-A6B6-01A-11R-A30C-07 | 1 | NA | 309 | Primary solid Tumor |
| TCGA-DK-AA6L-01A-11R-A39I-07 | 1 | NA | 401 | Primary solid Tumor |
| TCGA-DK-AA6M-01A-11R-A39I-07 | 2 | NA | 401 | Primary solid Tumor |
| TCGA-DK-AA6P-01A-11R-A39I-07 | 1 | NA | 401 | Primary solid Tumor |
| TCGA-DK-AA6Q-01A-11R-A39I-07 | 2 | NA | 401 | Primary solid Tumor |
| TCGA-DK-AA6R-01A-11R-A42T-07 | 2 | NA | 433 | Primary solid Tumor |
| TCGA-DK-AA6S-01A-21R-A39I-07 | 1 | NA | 401 | Primary solid Tumor |
| TCGA-DK-AA6T-01A-11R-A39I-07 | 6 | NA | 401 | Primary solid Tumor |
| TCGA-DK-AA6U-01A-11R-A39I-07 | 1 | NA | 401 | Primary solid Tumor |
| TCGA-DK-AA6W-01A-12R-A39I-07 | 3 | NA | 401 | Primary solid Tumor |
| TCGA-DK-AA6X-01A-12R-A42T-07 | 1 | NA | 433 | Primary solid Tumor |
| TCGA-DK-AA71-01A-31R-A39I-07 | 1 | NA | 401 | Primary solid Tumor |
| TCGA-DK-AA74-01A-11R-A39I-07 | 2 | NA | 401 | Primary solid Tumor |
| TCGA-DK-AA75-01A-11R-A39I-07 | 1 | NA | 401 | Primary solid Tumor |
| TCGA-DK-AA76-01A-11R-A39I-07 | 1 | NA | 401 | Primary solid Tumor |
| TCGA-DK-AA77-01A-11R-A39I-07 | 1 | NA | 401 | Primary solid Tumor |
| TCGA-DM-A0X9-01A-11R-A155-07 | 7 | NA | 132 | Primary solid Tumor |
| TCGA-DM-A0XD-01A-12R-A155-07 | 7 | NA | 132 | Primary solid Tumor |
| TCGA-DM-A0XF-01A-11R-A155-07 | 7 | NA | 132 | Primary solid Tumor |
| TCGA-DM-A1D0-01A-11R-A155-07 | 7 | NA | 132 | Primary solid Tumor |
| TCGA-DM-A1D4-01A-21R-A155-07 | 7 | NA | 132 | Primary solid Tumor |
| TCGA-DM-A1D6-01A-21R-A155-07 | 7 | NA | 132 | Primary solid Tumor |
| TCGA-DM-A1D7-01A-11R-A155-07 | 7 | NA | 132 | Primary solid Tumor |
| TCGA-DM-A1D8-01A-11R-A155-07 | 7 | NA | 132 | Primary solid Tumor |
| TCGA-DM-A1D9-01A-11R-A155-07 | 7 | NA | 132 | Primary solid Tumor |
| TCGA-DM-A1DA-01A-11R-A155-07 | 7 | NA | 132 | Primary solid Tumor |
| TCGA-DM-A1DB-01A-11R-A155-07 | 7 | NA | 132 | Primary solid Tumor |
| TCGA-DM-A1HA-01A-11R-A155-07 | 7 | NA | 132 | Primary solid Tumor |
| TCGA-DM-A1HB-01A-21R-A180-07 | 7 | NA | 172 | Primary solid Tumor |
| TCGA-DM-A280-01A-12R-A16W-07 | 7 | NA | 154 | Primary solid Tumor |
| TCGA-DM-A282-01A-12R-A16W-07 | 7 | NA | 154 | Primary solid Tumor |
| TCGA-DM-A285-01A-11R-A16W-07 | 7 | NA | 154 | Primary solid Tumor |
| TCGA-DM-A288-01A-11R-A16W-07 | 7 | NA | 154 | Primary solid Tumor |
| TCGA-DM-A28A-01A-21R-A32Y-07 | 7 | NA | 154 | Primary solid Tumor |
| TCGA-DM-A28C-01A-11R-A32Y-07 | 7 | NA | 154 | Primary solid Tumor |
| TCGA-DM-A28E-01A-11R-A32Y-07 | 7 | NA | 154 | Primary solid Tumor |
| TCGA-DM-A28F-01A-11R-A32Y-07 | 7 | NA | 154 | Primary solid Tumor |
| TCGA-DM-A28G-01A-11R-A16W-07 | 7 | NA | 154 | Primary solid Tumor |
| TCGA-DM-A28H-01A-11R-A16W-07 | 7 | NA | 154 | Primary solid Tumor |
| TCGA-DM-A28K-01A-21R-A32Y-07 | 7 | NA | 154 | Primary solid Tumor |
| TCGA-DM-A28M-01A-12R-A16W-07 | 7 | NA | 154 | Primary solid Tumor |
| TCGA-DQ-5624-01A-01R-1873-07 | 2 | 4 | 145 | Primary solid Tumor |
| TCGA-DQ-5625-01A-01R-1873-07 | 2 | 4 | 145 | Primary solid Tumor |
| TCGA-DQ-5629-01A-01R-1873-07 | 2 | 4 | 145 | Primary solid Tumor |
| TCGA-DQ-5630-01A-01R-1873-07 | 2 | 4 | 145 | Primary solid Tumor |
| TCGA-DQ-5631-01A-01R-1873-07 | 2 | 4 | 145 | Primary solid Tumor |
| TCGA-DQ-7588-01A-11R-2081-07 | 2 | 4 | 188 | Primary solid Tumor |
| TCGA-DQ-7589-01A-11R-2232-07 | 2 | 4 | 215 | Primary solid Tumor |
| TCGA-DQ-7590-01A-11R-2232-07 | 2 | 4 | 215 | Primary solid Tumor |
| TCGA-DQ-7591-01A-11R-2081-07 | 2 | 4 | 188 | Primary solid Tumor |
| TCGA-DQ-7592-01A-11R-2081-07 | 2 | 4 | 188 | Primary solid Tumor |
| TCGA-DQ-7593-01A-11R-2232-07 | 2 | 4 | 215 | Primary solid Tumor |
| TCGA-DQ-7594-01A-11R-2232-07 | 2 | 4 | 215 | Primary solid Tumor |
| TCGA-DQ-7595-01A-11R-2232-07 | 2 | 4 | 215 | Primary solid Tumor |
| TCGA-DQ-7596-01A-11R-2232-07 | 2 | 4 | 215 | Primary solid Tumor |
| TCGA-DT-5265-01A-21R-1830-07 | 7 | NA | 139 | Primary solid Tumor |
| TCGA-DV-5565-01A-01R-1541-07 | 14 | 9 | 90 | Primary solid Tumor |
| TCGA-DV-5566-01A-01R-1541-07 | 14 | 9 | 90 | Primary solid Tumor |
| TCGA-DV-5567-01A-01R-1541-07 | 14 | 9 | 90 | Primary solid Tumor |
| TCGA-DV-5568-01A-01R-1541-07 | 14 | 9 | 90 | Primary solid Tumor |
| TCGA-DV-5569-01A-01R-1541-07 | 14 | 9 | 90 | Primary solid Tumor |
| TCGA-DV-5573-01A-01R-1541-07 | 14 | 9 | 90 | Primary solid Tumor |
| TCGA-DV-5574-01A-01R-1541-07 | 14 | 9 | 90 | Primary solid Tumor |
| TCGA-DV-5575-01A-01R-1541-07 | 14 | 9 | 90 | Primary solid Tumor |
| TCGA-DV-5576-01A-01R-1541-07 | 14 | 9 | 90 | Primary solid Tumor |
| TCGA-DV-A4VX-01A-11R-A266-07 | 14 | NA | 274 | Primary solid Tumor |
| TCGA-DV-A4VZ-01A-11R-A266-07 | 14 | NA | 274 | Primary solid Tumor |
| TCGA-DV-A4W0-01A-11R-A266-07 | 14 | NA | 274 | Primary solid Tumor |
| TCGA-DY-A0XA-01A-11R-A155-07 | 7 | NA | 133 | Primary solid Tumor |
| TCGA-DY-A1DC-01A-31R-A155-07 | 7 | NA | 133 | Primary solid Tumor |
| TCGA-DY-A1DD-01A-21R-A155-07 | 7 | NA | 133 | Primary solid Tumor |
| TCGA-DY-A1DE-01A-11R-A155-07 | 7 | NA | 133 | Primary solid Tumor |
| TCGA-DY-A1DF-01A-11R-A155-07 | 7 | NA | 133 | Primary solid Tumor |
| TCGA-DY-A1DG-01A-11R-A32Y-07 | 7 | NA | 133 | Primary solid Tumor |
| TCGA-DY-A1H8-01A-21R-A155-07 | 7 | NA | 133 | Primary solid Tumor |
| TCGA-E2-A105-01A-11R-A10J-07 | 9 | 7 | 80 | Primary solid Tumor |
| TCGA-E2-A106-01A-11R-A10J-07 | 9 | 7 | 80 | Primary solid Tumor |
| TCGA-E2-A107-01A-11R-A10J-07 | 9 | NA | 80 | Primary solid Tumor |
| TCGA-E2-A108-01A-13R-A10J-07 | 9 | 7 | 80 | Primary solid Tumor |
| TCGA-E2-A109-01A-11R-A10J-07 | 9 | 7 | 80 | Primary solid Tumor |
| TCGA-E2-A10A-01A-21R-A115-07 | 9 | 7 | 85 | Primary solid Tumor |
| TCGA-E2-A10B-01A-11R-A10J-07 | 9 | 7 | 80 | Primary solid Tumor |
| TCGA-E2-A10C-01A-21R-A10J-07 | 9 | 7 | 80 | Primary solid Tumor |
| TCGA-E2-A10E-01A-21R-A10J-07 | 9 | 7 | 80 | Primary solid Tumor |
| TCGA-E2-A10F-01A-11R-A10J-07 | 9 | 7 | 80 | Primary solid Tumor |
| TCGA-E2-A14N-01A-31R-A137-07 | 10 | 8 | 103 | Primary solid Tumor |
| TCGA-E2-A14O-01A-31R-A115-07 | 9 | 7 | 85 | Primary solid Tumor |
| TCGA-E2-A14P-01A-31R-A12D-07 | 9 | 7 | 93 | Primary solid Tumor |
| TCGA-E2-A14Q-01A-11R-A12D-07 | 9 | 7 | 93 | Primary solid Tumor |
| TCGA-E2-A14R-01A-11R-A115-07 | 10 | 8 | 85 | Primary solid Tumor |
| TCGA-E2-A14S-01A-11R-A12D-07 | 9 | 7 | 93 | Primary solid Tumor |
| TCGA-E2-A14T-01A-11R-A115-07 | 9 | 7 | 85 | Primary solid Tumor |
| TCGA-E2-A14U-01A-11R-A22K-07 | 9 | NA | 227 | Primary solid Tumor |
| TCGA-E2-A14V-01A-11R-A12D-07 | 9 | 7 | 93 | Primary solid Tumor |
| TCGA-E2-A14W-01A-11R-A12D-07 | 9 | 7 | 93 | Primary solid Tumor |
| TCGA-E2-A14X-01A-11R-A115-07 | 10 | 8 | 85 | Primary solid Tumor |
| TCGA-E2-A14Y-01A-21R-A12D-07 | 10 | 8 | 93 | Primary solid Tumor |
| TCGA-E2-A14Z-01A-11R-A115-07 | 9 | 7 | 85 | Primary solid Tumor |
| TCGA-E2-A150-01A-11R-A12D-07 | 10 | 8 | 93 | Primary solid Tumor |
| TCGA-E2-A152-01A-11R-A12D-07 | 9 | 7 | 93 | Primary solid Tumor |
| TCGA-E2-A153-01A-12R-A12D-07 | 9 | 7 | 93 | Primary solid Tumor |
| TCGA-E2-A154-01A-11R-A115-07 | 9 | 7 | 85 | Primary solid Tumor |
| TCGA-E2-A155-01A-11R-A12D-07 | 9 | 7 | 93 | Primary solid Tumor |
| TCGA-E2-A156-01A-11R-A12D-07 | 9 | 7 | 93 | Primary solid Tumor |
| TCGA-E2-A158-01A-11R-A12D-07 | 10 | 8 | 93 | Primary solid Tumor |
| TCGA-E2-A159-01A-11R-A115-07 | 10 | 8 | 85 | Primary solid Tumor |
| TCGA-E2-A15A-01A-11R-A12D-07 | 9 | 7 | 93 | Primary solid Tumor |
| TCGA-E2-A15C-01A-31R-A12D-07 | 9 | 7 | 93 | Primary solid Tumor |
| TCGA-E2-A15D-01A-11R-A115-07 | 9 | 7 | 85 | Primary solid Tumor |
| TCGA-E2-A15E-01A-11R-A12D-07 | 9 | 7 | 93 | Primary solid Tumor |
| TCGA-E2-A15F-01A-11R-A115-07 | 9 | NA | 85 | Primary solid Tumor |
| TCGA-E2-A15G-01A-11R-A12D-07 | 9 | 7 | 93 | Primary solid Tumor |
| TCGA-E2-A15H-01A-11R-A12D-07 | 9 | 7 | 93 | Primary solid Tumor |
| TCGA-E2-A15I-01A-21R-A137-07 | 9 | 7 | 103 | Primary solid Tumor |
| TCGA-E2-A15J-01A-11R-A12P-07 | 9 | 7 | 96 | Primary solid Tumor |
| TCGA-E2-A15K-01A-11R-A12P-07 | 9 | 7 | 96 | Primary solid Tumor |
| TCGA-E2-A15L-01A-11R-A12D-07 | 9 | 7 | 93 | Primary solid Tumor |
| TCGA-E2-A15M-01A-11R-A12D-07 | 9 | 7 | 93 | Primary solid Tumor |
| TCGA-E2-A15O-01A-11R-A115-07 | 9 | 7 | 85 | Primary solid Tumor |
| TCGA-E2-A15P-01A-11R-A115-07 | 9 | 7 | 85 | Primary solid Tumor |
| TCGA-E2-A15R-01A-11R-A115-07 | 9 | 7 | 85 | Primary solid Tumor |
| TCGA-E2-A15S-01A-11R-A115-07 | 9 | 7 | 85 | Primary solid Tumor |
| TCGA-E2-A15T-01A-11R-A115-07 | 9 | NA | 85 | Primary solid Tumor |
| TCGA-E2-A1AZ-01A-11R-A12P-07 | 10 | 8 | 96 | Primary solid Tumor |
| TCGA-E2-A1B0-01A-11R-A12P-07 | 9 | 7 | 96 | Primary solid Tumor |
| TCGA-E2-A1B1-01A-21R-A12P-07 | 9 | 7 | 96 | Primary solid Tumor |
| TCGA-E2-A1B4-01A-11R-A12P-07 | 9 | 7 | 96 | Primary solid Tumor |
| TCGA-E2-A1B5-01A-21R-A12P-07 | 9 | 7 | 96 | Primary solid Tumor |
| TCGA-E2-A1B6-01A-31R-A12P-07 | 10 | NA | 96 | Primary solid Tumor |
| TCGA-E2-A1BC-01A-11R-A12P-07 | 9 | 7 | 96 | Primary solid Tumor |
| TCGA-E2-A1BD-01A-11R-A12P-07 | 9 | 7 | 96 | Primary solid Tumor |
| TCGA-E2-A1IE-01A-11R-A13Q-07 | 9 | 7 | 109 | Primary solid Tumor |
| TCGA-E2-A1IF-01A-11R-A144-07 | 9 | 7 | 117 | Primary solid Tumor |
| TCGA-E2-A1IG-01A-11R-A144-07 | 9 | 7 | 117 | Primary solid Tumor |
| TCGA-E2-A1IH-01A-11R-A13Q-07 | 9 | 7 | 109 | Primary solid Tumor |
| TCGA-E2-A1II-01A-11R-A144-07 | 10 | 8 | 117 | Primary solid Tumor |
| TCGA-E2-A1IJ-01A-11R-A144-07 | 9 | 7 | 117 | Primary solid Tumor |
| TCGA-E2-A1IK-01A-11R-A144-07 | 9 | 7 | 117 | Primary solid Tumor |
| TCGA-E2-A1IL-01A-11R-A14D-07 | 9 | 7 | 120 | Primary solid Tumor |
| TCGA-E2-A1IN-01A-11R-A13Q-07 | 9 | 7 | 109 | Primary solid Tumor |
| TCGA-E2-A1IO-01A-11R-A144-07 | 9 | 7 | 117 | Primary solid Tumor |
| TCGA-E2-A1IU-01A-11R-A14D-07 | 9 | 7 | 120 | Primary solid Tumor |
| TCGA-E2-A1L6-01A-11R-A13Q-07 | 9 | 7 | 109 | Primary solid Tumor |
| TCGA-E2-A1L7-01A-11R-A144-07 | 9 | 7 | 117 | Primary solid Tumor |
| TCGA-E2-A1L8-01A-11R-A13Q-07 | 9 | 7 | 109 | Primary solid Tumor |
| TCGA-E2-A1L9-01A-11R-A13Q-07 | 9 | 7 | 109 | Primary solid Tumor |
| TCGA-E2-A1LA-01A-11R-A144-07 | 9 | 7 | 117 | Primary solid Tumor |
| TCGA-E2-A1LB-01A-11R-A144-07 | 9 | 7 | 117 | Primary solid Tumor |
| TCGA-E2-A1LE-01A-12R-A19W-07 | 9 | NA | 185 | Primary solid Tumor |
| TCGA-E2-A1LG-01A-21R-A14M-07 | 10 | 8 | 124 | Primary solid Tumor |
| TCGA-E2-A1LH-01A-11R-A14D-07 | 10 | 8 | 120 | Primary solid Tumor |
| TCGA-E2-A1LI-01A-12R-A157-07 | 10 | 8 | 136 | Primary solid Tumor |
| TCGA-E2-A1LK-01A-21R-A14D-07 | 10 | 8 | 120 | Primary solid Tumor |
| TCGA-E2-A1LL-01A-11R-A144-07 | 2 | 4 | 117 | Primary solid Tumor |
| TCGA-E2-A1LS-01A-12R-A157-07 | 10 | 8 | 136 | Primary solid Tumor |
| TCGA-E2-A2P5-01A-11R-A19W-07 | 9 | NA | 185 | Primary solid Tumor |
| TCGA-E2-A2P6-01A-11R-A19W-07 | 9 | NA | 185 | Primary solid Tumor |
| TCGA-E2-A3DX-01A-21R-A213-07 | 9 | 7 | 202 | Primary solid Tumor |
| TCGA-E2-A56Z-01A-12R-A29R-07 | 9 | NA | 305 | Primary solid Tumor |
| TCGA-E2-A570-01A-11R-A29R-07 | 9 | NA | 305 | Primary solid Tumor |
| TCGA-E2-A572-01A-13R-A31O-07 | 9 | NA | 322 | Primary solid Tumor |
| TCGA-E2-A573-01A-11R-A29R-07 | 10 | NA | 305 | Primary solid Tumor |
| TCGA-E2-A574-01A-11R-A29R-07 | 10 | NA | 305 | Primary solid Tumor |
| TCGA-E2-A576-01A-11R-A31O-07 | 9 | NA | 322 | Primary solid Tumor |
| TCGA-E2-A9RU-01A-11R-A41B-07 | 9 | NA | 379 | Primary solid Tumor |
| TCGA-E5-A2PC-01A-11R-A206-07 | 1 | 4 | 192 | Primary solid Tumor |
| TCGA-E5-A4TZ-01A-11R-A31N-07 | 1 | NA | 317 | Primary solid Tumor |
| TCGA-E5-A4U1-01A-11R-A31N-07 | 1 | NA | 317 | Primary solid Tumor |
| TCGA-E6-A1LX-01A-11R-A14D-07 | 16 | 11 | 121 | Primary solid Tumor |
| TCGA-E6-A1LZ-01A-11R-A144-07 | 16 | 11 | 118 | Primary solid Tumor |
| TCGA-E6-A1M0-01A-11R-A144-07 | 16 | 11 | 118 | Primary solid Tumor |
| TCGA-E6-A2P8-01A-11R-A19W-07 | 3 | NA | 186 | Primary solid Tumor |
| TCGA-E6-A2P9-01A-11R-A19W-07 | 16 | NA | 186 | Primary solid Tumor |
| TCGA-E6-A8L9-01A-21R-A37O-07 | 16 | NA | 381 | Primary solid Tumor |
| TCGA-E7-A3X6-01A-12R-A22U-07 | 1 | 4 | 235 | Primary solid Tumor |
| TCGA-E7-A3Y1-01A-11R-A22U-07 | 1 | 1 | 235 | Primary solid Tumor |
| TCGA-E7-A4IJ-01A-31R-A26T-07 | 1 | NA | 279 | Primary solid Tumor |
| TCGA-E7-A4XJ-01A-11R-A26T-07 | 1 | NA | 279 | Primary solid Tumor |
| TCGA-E7-A519-01A-11R-A26T-07 | 1 | NA | 279 | Primary solid Tumor |
| TCGA-E7-A541-01A-11R-A26T-07 | 1 | NA | 279 | Primary solid Tumor |
| TCGA-E7-A5KE-01A-11R-A28M-07 | 1 | NA | 294 | Primary solid Tumor |
| TCGA-E7-A5KF-01A-11R-A28M-07 | 1 | NA | 294 | Primary solid Tumor |
| TCGA-E7-A677-01A-11R-A30C-07 | 1 | NA | 309 | Primary solid Tumor |
| TCGA-E7-A678-01A-11R-A30C-07 | 1 | NA | 309 | Primary solid Tumor |
| TCGA-E7-A6MD-01A-41R-A352-07 | 1 | NA | 356 | Primary solid Tumor |
| TCGA-E7-A6ME-01A-22R-A32O-07 | 1 | NA | 328 | Primary solid Tumor |
| TCGA-E7-A6MF-01A-12R-A32O-07 | 1 | NA | 328 | Primary solid Tumor |
| TCGA-E7-A7DU-01A-11R-A32O-07 | 1 | NA | 328 | Primary solid Tumor |
| TCGA-E7-A7DV-01A-11R-A33J-07 | 3 | NA | 337 | Primary solid Tumor |
| TCGA-E7-A7PW-01A-11R-A352-07 | 1 | NA | 356 | Primary solid Tumor |
| TCGA-E7-A7XN-01A-11R-A352-07 | 2 | NA | 356 | Primary solid Tumor |
| TCGA-E7-A85H-01A-11R-A352-07 | 1 | NA | 356 | Primary solid Tumor |
| TCGA-E7-A8O7-01A-11R-A36F-07 | 1 | NA | 367 | Primary solid Tumor |
| TCGA-E7-A8O8-01A-11R-A36F-07 | 1 | NA | 367 | Primary solid Tumor |
| TCGA-E7-A97P-01A-11R-A38B-07 | 2 | NA | 391 | Primary solid Tumor |
| TCGA-E7-A97Q-01A-11R-A38B-07 | 1 | NA | 391 | Primary solid Tumor |
| TCGA-E9-A1N3-01A-12R-A157-07 | 9 | 7 | 136 | Primary solid Tumor |
| TCGA-E9-A1N4-01A-11R-A14M-07 | 9 | 7 | 124 | Primary solid Tumor |
| TCGA-E9-A1N5-01A-11R-A14D-07 | 9 | 7 | 120 | Primary solid Tumor |
| TCGA-E9-A1N6-01A-11R-A144-07 | 9 | 7 | 117 | Primary solid Tumor |
| TCGA-E9-A1N8-01A-11R-A144-07 | 10 | 8 | 117 | Primary solid Tumor |
| TCGA-E9-A1N9-01A-11R-A14D-07 | 9 | 7 | 120 | Primary solid Tumor |
| TCGA-E9-A1NA-01A-11R-A144-07 | 9 | 7 | 117 | Primary solid Tumor |
| TCGA-E9-A1NC-01A-21R-A26B-07 | 10 | NA | 136 | Primary solid Tumor |
| TCGA-E9-A1ND-01A-11R-A144-07 | 10 | 8 | 117 | Primary solid Tumor |
| TCGA-E9-A1NE-01A-21R-A14M-07 | 9 | 7 | 124 | Primary solid Tumor |
| TCGA-E9-A1NF-01A-11R-A14D-07 | 9 | 7 | 120 | Primary solid Tumor |
| TCGA-E9-A1NG-01A-21R-A14M-07 | 9 | 7 | 124 | Primary solid Tumor |
| TCGA-E9-A1NH-01A-11R-A14D-07 | 9 | 7 | 120 | Primary solid Tumor |
| TCGA-E9-A1NI-01A-11R-A14D-07 | 9 | 7 | 120 | Primary solid Tumor |
| TCGA-E9-A1QZ-01A-21R-A169-07 | 9 | 7 | 142 | Primary solid Tumor |
| TCGA-E9-A1R0-01A-22R-A16F-07 | 9 | 7 | 147 | Primary solid Tumor |
| TCGA-E9-A1R2-01A-11R-A14D-07 | 9 | 7 | 120 | Primary solid Tumor |
| TCGA-E9-A1R3-01A-31R-A14M-07 | 9 | 7 | 124 | Primary solid Tumor |
| TCGA-E9-A1R4-01A-21R-A14D-07 | 9 | 7 | 120 | Primary solid Tumor |
| TCGA-E9-A1R5-01A-11R-A14M-07 | 9 | 7 | 124 | Primary solid Tumor |
| TCGA-E9-A1R6-01A-11R-A14D-07 | 9 | 7 | 120 | Primary solid Tumor |
| TCGA-E9-A1R7-01A-11R-A14M-07 | 9 | 7 | 124 | Primary solid Tumor |
| TCGA-E9-A1RA-01A-11R-A14D-07 | 9 | NA | 120 | Primary solid Tumor |
| TCGA-E9-A1RB-01A-11R-A157-07 | 9 | 7 | 136 | Primary solid Tumor |
| TCGA-E9-A1RC-01A-11R-A157-07 | 9 | 7 | 136 | Primary solid Tumor |
| TCGA-E9-A1RD-01A-11R-A157-07 | 9 | 7 | 136 | Primary solid Tumor |
| TCGA-E9-A1RE-01A-11R-A157-07 | 9 | 7 | 136 | Primary solid Tumor |
| TCGA-E9-A1RF-01A-11R-A157-07 | 9 | 7 | 136 | Primary solid Tumor |
| TCGA-E9-A1RG-01A-11R-A14D-07 | 9 | NA | 120 | Primary solid Tumor |
| TCGA-E9-A1RH-01A-21R-A169-07 | 9 | 7 | 142 | Primary solid Tumor |
| TCGA-E9-A1RI-01A-11R-A169-07 | 9 | 7 | 142 | Primary solid Tumor |
| TCGA-E9-A226-01A-21R-A157-07 | 9 | 7 | 136 | Primary solid Tumor |
| TCGA-E9-A227-01A-11R-A157-07 | 9 | 7 | 136 | Primary solid Tumor |
| TCGA-E9-A228-01A-31R-A157-07 | 9 | 7 | 136 | Primary solid Tumor |
| TCGA-E9-A229-01A-31R-A157-07 | 9 | 7 | 136 | Primary solid Tumor |
| TCGA-E9-A22A-01A-11R-A157-07 | 9 | 7 | 136 | Primary solid Tumor |
| TCGA-E9-A22B-01A-11R-A157-07 | 9 | 7 | 136 | Primary solid Tumor |
| TCGA-E9-A22D-01A-11R-A157-07 | 9 | 7 | 136 | Primary solid Tumor |
| TCGA-E9-A22E-01A-11R-A157-07 | 9 | 7 | 136 | Primary solid Tumor |
| TCGA-E9-A22G-01A-11R-A157-07 | 10 | 8 | 136 | Primary solid Tumor |
| TCGA-E9-A22H-01A-11R-A157-07 | 9 | 7 | 136 | Primary solid Tumor |
| TCGA-E9-A243-01A-21R-A169-07 | 10 | 8 | 142 | Primary solid Tumor |
| TCGA-E9-A244-01A-11R-A169-07 | 10 | 8 | 142 | Primary solid Tumor |
| TCGA-E9-A245-01A-22R-A16F-07 | 9 | 7 | 147 | Primary solid Tumor |
| TCGA-E9-A247-01A-11R-A169-07 | 9 | 7 | 142 | Primary solid Tumor |
| TCGA-E9-A248-01A-11R-A169-07 | 9 | 7 | 142 | Primary solid Tumor |
| TCGA-E9-A249-01A-11R-A169-07 | 9 | 7 | 142 | Primary solid Tumor |
| TCGA-E9-A24A-01A-11R-A169-07 | 9 | 7 | 142 | Primary solid Tumor |
| TCGA-E9-A295-01A-11R-A16F-07 | 9 | 7 | 147 | Primary solid Tumor |
| TCGA-E9-A2JS-01A-11R-A180-07 | 9 | 7 | 167 | Primary solid Tumor |
| TCGA-E9-A2JT-01A-22R-A18M-07 | 6 | 7 | 177 | Primary solid Tumor |
| TCGA-E9-A3HO-01A-11R-A213-07 | 9 | 7 | 202 | Primary solid Tumor |
| TCGA-E9-A3Q9-01A-11R-A21T-07 | 9 | 7 | 216 | Primary solid Tumor |
| TCGA-E9-A3QA-01A-61R-A22K-07 | 10 | NA | 227 | Primary solid Tumor |
| TCGA-E9-A3X8-01A-31R-A22U-07 | 9 | NA | 234 | Primary solid Tumor |
| TCGA-E9-A54X-01A-11R-A266-07 | 9 | NA | 271 | Primary solid Tumor |
| TCGA-E9-A54Y-01A-11R-A466-07 | 9 | NA | 271 | Primary solid Tumor |
| TCGA-E9-A5FK-01A-11R-A27Q-07 | 9 | NA | 288 | Primary solid Tumor |
| TCGA-E9-A5FL-01A-11R-A27Q-07 | 2 | NA | 288 | Primary solid Tumor |
| TCGA-E9-A5UO-01A-11R-A28M-07 | 9 | NA | 296 | Primary solid Tumor |
| TCGA-E9-A5UP-01A-11R-A28M-07 | 9 | NA | 296 | Primary solid Tumor |
| TCGA-E9-A6HE-01A-11R-A31O-07 | 9 | NA | 322 | Primary solid Tumor |
| TCGA-EC-A1NJ-01A-31R-A14D-07 | 16 | 11 | 121 | Primary solid Tumor |
| TCGA-EC-A1QX-01A-31R-A16F-07 | 16 | 11 | 143 | Primary solid Tumor |
| TCGA-EC-A24G-01A-11R-A16F-07 | 16 | 11 | 143 | Primary solid Tumor |
| TCGA-EF-5830-01A-01R-1660-07 | 7 | NA | 102 | Primary solid Tumor |
| TCGA-EF-5831-01A-01R-1660-07 | 7 | NA | 102 | Primary solid Tumor |
| TCGA-EI-6506-01A-11R-1736-07 | 7 | NA | 122 | Primary solid Tumor |
| TCGA-EI-6507-01A-11R-1736-07 | 7 | NA | 122 | Primary solid Tumor |
| TCGA-EI-6508-01A-11R-1736-07 | 7 | NA | 122 | Primary solid Tumor |
| TCGA-EI-6509-01A-11R-1736-07 | 7 | NA | 122 | Primary solid Tumor |
| TCGA-EI-6510-01A-11R-1736-07 | 7 | NA | 122 | Primary solid Tumor |
| TCGA-EI-6511-01A-11R-1736-07 | 7 | NA | 122 | Primary solid Tumor |
| TCGA-EI-6512-01A-11R-1736-07 | 7 | NA | 122 | Primary solid Tumor |
| TCGA-EI-6513-01A-21R-1736-07 | 7 | NA | 122 | Primary solid Tumor |
| TCGA-EI-6514-01A-11R-1736-07 | 7 | NA | 122 | Primary solid Tumor |
| TCGA-EI-6881-01A-11R-A32Z-07 | 7 | NA | 158 | Primary solid Tumor |
| TCGA-EI-6882-01A-11R-1928-07 | 7 | NA | 158 | Primary solid Tumor |
| TCGA-EI-6883-01A-31R-1928-07 | 7 | NA | 158 | Primary solid Tumor |
| TCGA-EI-6884-01A-11R-1928-07 | 7 | NA | 158 | Primary solid Tumor |
| TCGA-EI-6885-01A-11R-1928-07 | 7 | NA | 158 | Primary solid Tumor |
| TCGA-EI-6917-01A-11R-1928-07 | 7 | NA | 158 | Primary solid Tumor |
| TCGA-EI-7002-01A-11R-1928-07 | 7 | NA | 158 | Primary solid Tumor |
| TCGA-EI-7004-01A-11R-1928-07 | 7 | NA | 158 | Primary solid Tumor |
| TCGA-EO-A1Y5-01A-11R-A157-07 | 16 | 11 | 137 | Primary solid Tumor |
| TCGA-EO-A1Y7-01A-11R-A157-07 | 16 | NA | 137 | Primary solid Tumor |
| TCGA-EO-A1Y8-01A-11R-A157-07 | 16 | 11 | 137 | Primary solid Tumor |
| TCGA-EO-A22R-01A-11R-A18M-07 | 16 | 11 | 178 | Primary solid Tumor |
| TCGA-EO-A22S-01A-11R-A18M-07 | 16 | 11 | 178 | Primary solid Tumor |
| TCGA-EO-A22T-01A-21R-A18M-07 | 16 | 11 | 178 | Primary solid Tumor |
| TCGA-EO-A22U-01A-11R-A180-07 | 16 | NA | 168 | Primary solid Tumor |
| TCGA-EO-A22X-01A-11R-A180-07 | 16 | NA | 168 | Primary solid Tumor |
| TCGA-EO-A22Y-01A-11R-A180-07 | 16 | NA | 168 | Primary solid Tumor |
| TCGA-EO-A2CG-01A-12R-A180-07 | 16 | 11 | 168 | Primary solid Tumor |
| TCGA-EO-A2CH-01A-11R-A180-07 | 16 | 11 | 168 | Primary solid Tumor |
| TCGA-EO-A3AS-01A-11R-A19W-07 | 16 | NA | 186 | Primary solid Tumor |
| TCGA-EO-A3AU-01A-21R-A19W-07 | 16 | NA | 186 | Primary solid Tumor |
| TCGA-EO-A3AV-01A-12R-A19W-07 | 16 | NA | 186 | Primary solid Tumor |
| TCGA-EO-A3AY-01A-12R-A19W-07 | 16 | NA | 186 | Primary solid Tumor |
| TCGA-EO-A3AZ-01A-12R-A19W-07 | 16 | NA | 186 | Primary solid Tumor |
| TCGA-EO-A3B0-01A-12R-A19W-07 | 16 | NA | 186 | Primary solid Tumor |
| TCGA-EO-A3B1-01A-12R-A19W-07 | 16 | NA | 186 | Primary solid Tumor |
| TCGA-EO-A3KU-01A-11R-A22K-07 | 16 | NA | 228 | Primary solid Tumor |
| TCGA-EO-A3KW-01A-11R-A22K-07 | 20 | NA | 228 | Primary solid Tumor |
| TCGA-EO-A3KX-01A-11R-A22K-07 | 16 | NA | 228 | Primary solid Tumor |
| TCGA-EO-A3L0-01A-11R-A22K-07 | 16 | NA | 228 | Primary solid Tumor |
| TCGA-EU-5904-01A-11R-1672-07 | 14 | 9 | 105 | Primary solid Tumor |
| TCGA-EU-5905-01A-11R-1672-07 | 14 | 9 | 105 | Primary solid Tumor |
| TCGA-EU-5906-01A-11R-1672-07 | 14 | 9 | 105 | Primary solid Tumor |
| TCGA-EU-5907-01A-11R-1672-07 | 14 | NA | 105 | Primary solid Tumor |
| TCGA-EW-A1IW-01A-11R-A13Q-07 | 9 | 7 | 109 | Primary solid Tumor |
| TCGA-EW-A1IX-01A-12R-A144-07 | 9 | 7 | 117 | Primary solid Tumor |
| TCGA-EW-A1IY-01A-11R-A13Q-07 | 9 | 7 | 109 | Primary solid Tumor |
| TCGA-EW-A1IZ-01A-11R-A13Q-07 | 9 | NA | 109 | Primary solid Tumor |
| TCGA-EW-A1J1-01A-11R-A13Q-07 | 9 | 7 | 109 | Primary solid Tumor |
| TCGA-EW-A1J2-01A-21R-A13Q-07 | 9 | 7 | 109 | Primary solid Tumor |
| TCGA-EW-A1J3-01A-11R-A13Q-07 | 9 | 7 | 109 | Primary solid Tumor |
| TCGA-EW-A1J5-01A-11R-A13Q-07 | 9 | 7 | 109 | Primary solid Tumor |
| TCGA-EW-A1J6-01A-11R-A13Q-07 | 9 | 7 | 109 | Primary solid Tumor |
| TCGA-EW-A1OV-01A-11R-A144-07 | 9 | 7 | 117 | Primary solid Tumor |
| TCGA-EW-A1OW-01A-21R-A144-07 | 10 | 8 | 117 | Primary solid Tumor |
| TCGA-EW-A1OX-01A-11R-A144-07 | 9 | 7 | 117 | Primary solid Tumor |
| TCGA-EW-A1OY-01A-11R-A144-07 | 9 | 7 | 117 | Primary solid Tumor |
| TCGA-EW-A1OZ-01A-11R-A144-07 | 9 | 7 | 117 | Primary solid Tumor |
| TCGA-EW-A1P0-01A-11R-A144-07 | 9 | 7 | 117 | Primary solid Tumor |
| TCGA-EW-A1P1-01A-31R-A14D-07 | 9 | NA | 120 | Primary solid Tumor |
| TCGA-EW-A1P3-01A-11R-A144-07 | 9 | 7 | 117 | Primary solid Tumor |
| TCGA-EW-A1P4-01A-21R-A144-07 | 10 | 8 | 117 | Primary solid Tumor |
| TCGA-EW-A1P5-01A-11R-A144-07 | 9 | 7 | 117 | Primary solid Tumor |
| TCGA-EW-A1P6-01A-11R-A144-07 | 9 | 7 | 117 | Primary solid Tumor |
| TCGA-EW-A1P7-01A-21R-A144-07 | 10 | 8 | 117 | Primary solid Tumor |
| TCGA-EW-A1P8-01A-11R-A144-07 | 10 | 8 | 117 | Primary solid Tumor |
| TCGA-EW-A1PA-01A-11R-A144-07 | 9 | 7 | 117 | Primary solid Tumor |
| TCGA-EW-A1PB-01A-11R-A144-07 | 10 | 8 | 117 | Primary solid Tumor |
| TCGA-EW-A1PC-01B-11R-A21T-07 | 9 | 7 | 216 | Primary solid Tumor |
| TCGA-EW-A1PD-01A-11R-A144-07 | 9 | 7 | 117 | Primary solid Tumor |
| TCGA-EW-A1PE-01A-11R-A144-07 | 9 | 7 | 117 | Primary solid Tumor |
| TCGA-EW-A1PF-01A-11R-A144-07 | 9 | 7 | 117 | Primary solid Tumor |
| TCGA-EW-A1PG-01A-11R-A144-07 | 9 | 7 | 117 | Primary solid Tumor |
| TCGA-EW-A1PH-01A-11R-A14M-07 | 10 | 8 | 124 | Primary solid Tumor |
| TCGA-EW-A2FR-01A-11R-A21T-07 | 9 | 7 | 216 | Primary solid Tumor |
| TCGA-EW-A2FS-01A-11R-A17B-07 | 9 | 7 | 155 | Primary solid Tumor |
| TCGA-EW-A2FV-01A-11R-A17B-07 | 9 | 7 | 155 | Primary solid Tumor |
| TCGA-EW-A2FW-01A-11R-A17B-07 | 9 | 7 | 155 | Primary solid Tumor |
| TCGA-EW-A3E8-01B-11R-A24H-07 | 9 | NA | 255 | Primary solid Tumor |
| TCGA-EW-A3U0-01A-11R-A22K-07 | 10 | NA | 227 | Primary solid Tumor |
| TCGA-EW-A423-01A-11R-A24H-07 | 9 | NA | 255 | Primary solid Tumor |
| TCGA-EW-A424-01A-11R-A24H-07 | 9 | NA | 255 | Primary solid Tumor |
| TCGA-EW-A6S9-01A-22R-A33J-07 | 9 | NA | 338 | Primary solid Tumor |
| TCGA-EW-A6SA-01A-21R-A32P-07 | 9 | NA | 334 | Primary solid Tumor |
| TCGA-EW-A6SB-01A-12R-A32P-07 | 10 | NA | 334 | Primary solid Tumor |
| TCGA-EW-A6SC-01A-12R-A32P-07 | 9 | NA | 334 | Primary solid Tumor |
| TCGA-EW-A6SD-01A-12R-A33J-07 | 9 | NA | 338 | Primary solid Tumor |
| TCGA-EY-A1G7-01A-11R-A13S-07 | 16 | 11 | 110 | Primary solid Tumor |
| TCGA-EY-A1G8-01A-11R-A13S-07 | 16 | 11 | 110 | Primary solid Tumor |
| TCGA-EY-A1GC-01A-11R-A13S-07 | 16 | 11 | 110 | Primary solid Tumor |
| TCGA-EY-A1GD-01A-11R-A13S-07 | 16 | 11 | 110 | Primary solid Tumor |
| TCGA-EY-A1GE-01A-11R-A13S-07 | 16 | 11 | 110 | Primary solid Tumor |
| TCGA-EY-A1GF-01A-11R-A13S-07 | 16 | 11 | 110 | Primary solid Tumor |
| TCGA-EY-A1GH-01A-11R-A13S-07 | 16 | 11 | 110 | Primary solid Tumor |
| TCGA-EY-A1GI-01A-11R-A13S-07 | 16 | 11 | 110 | Primary solid Tumor |
| TCGA-EY-A1GK-01A-11R-A13S-07 | 16 | 11 | 110 | Primary solid Tumor |
| TCGA-EY-A1GL-01A-11R-A13S-07 | 16 | NA | 110 | Primary solid Tumor |
| TCGA-EY-A1GM-01A-12R-A14D-07 | 16 | 11 | 121 | Primary solid Tumor |
| TCGA-EY-A1GO-01A-11R-A14D-07 | 16 | NA | 121 | Primary solid Tumor |
| TCGA-EY-A1GP-01A-11R-A13S-07 | 16 | NA | 110 | Primary solid Tumor |
| TCGA-EY-A1GQ-01A-21R-A13S-07 | 16 | 11 | 110 | Primary solid Tumor |
| TCGA-EY-A1GR-01A-11R-A13S-07 | 16 | 11 | 110 | Primary solid Tumor |
| TCGA-EY-A1GS-01A-11R-A13S-07 | 16 | 11 | 110 | Primary solid Tumor |
| TCGA-EY-A1GT-01A-11R-A13S-07 | 16 | 11 | 110 | Primary solid Tumor |
| TCGA-EY-A1GU-01A-11R-A13S-07 | 16 | 11 | 110 | Primary solid Tumor |
| TCGA-EY-A1GV-01A-11R-A13S-07 | 16 | 11 | 110 | Primary solid Tumor |
| TCGA-EY-A1GW-01A-22R-A13S-07 | 16 | 11 | 110 | Primary solid Tumor |
| TCGA-EY-A1GX-01A-12R-A13S-07 | 16 | NA | 110 | Primary solid Tumor |
| TCGA-EY-A1H0-01A-11R-A13S-07 | 16 | 11 | 110 | Primary solid Tumor |
| TCGA-EY-A210-01A-11R-A157-07 | 16 | NA | 137 | Primary solid Tumor |
| TCGA-EY-A212-01A-11R-A14M-07 | 16 | 11 | 125 | Primary solid Tumor |
| TCGA-EY-A214-01A-12R-A157-07 | 16 | 11 | 137 | Primary solid Tumor |
| TCGA-EY-A215-01A-11R-A14M-07 | 16 | 15 | 125 | Primary solid Tumor |
| TCGA-EY-A2OM-01A-11R-A18M-07 | 16 | 11 | 178 | Primary solid Tumor |
| TCGA-EY-A2ON-01A-21R-A18M-07 | 16 | NA | 178 | Primary solid Tumor |
| TCGA-EY-A2OO-01A-11R-A19W-07 | 16 | NA | 186 | Primary solid Tumor |
| TCGA-EY-A2OP-01A-11R-A19W-07 | 16 | NA | 186 | Primary solid Tumor |
| TCGA-EY-A2OQ-01A-11R-A19W-07 | 16 | NA | 186 | Primary solid Tumor |
| TCGA-EY-A3L3-01A-11R-A22K-07 | 16 | NA | 228 | Primary solid Tumor |
| TCGA-EY-A3QX-01A-11R-A22K-07 | 16 | NA | 228 | Primary solid Tumor |
| TCGA-EY-A4KR-01A-11R-A27V-07 | 20 | NA | 289 | Primary solid Tumor |
| TCGA-EY-A547-01A-11R-A27V-07 | 16 | NA | 289 | Primary solid Tumor |
| TCGA-EY-A548-01A-11R-A27V-07 | 16 | NA | 289 | Primary solid Tumor |
| TCGA-EY-A549-01A-11R-A27V-07 | 16 | NA | 289 | Primary solid Tumor |
| TCGA-EY-A54A-01A-11R-A27V-07 | 16 | NA | 289 | Primary solid Tumor |
| TCGA-EY-A5W2-01A-11R-A31O-07 | 16 | NA | 324 | Primary solid Tumor |
| TCGA-EY-A72D-01A-12R-A34R-07 | 16 | NA | 354 | Primary solid Tumor |
| TCGA-F4-6459-01A-11R-1774-07 | 7 | NA | 123 | Primary solid Tumor |
| TCGA-F4-6460-01A-11R-1774-07 | 7 | NA | 123 | Primary solid Tumor |
| TCGA-F4-6461-01A-11R-1774-07 | 7 | NA | 123 | Primary solid Tumor |
| TCGA-F4-6463-01A-11R-1723-07 | 7 | NA | 116 | Primary solid Tumor |
| TCGA-F4-6569-01A-11R-1774-07 | 7 | NA | 123 | Primary solid Tumor |
| TCGA-F4-6570-01A-11R-1774-07 | 7 | NA | 123 | Primary solid Tumor |
| TCGA-F4-6703-01A-11R-1839-07 | 7 | NA | 138 | Primary solid Tumor |
| TCGA-F4-6704-01A-11R-1839-07 | 7 | NA | 138 | Primary solid Tumor |
| TCGA-F4-6805-01A-11R-1839-07 | 7 | NA | 138 | Primary solid Tumor |
| TCGA-F4-6806-01A-11R-1839-07 | 7 | NA | 138 | Primary solid Tumor |
| TCGA-F4-6807-01A-11R-1839-07 | 7 | NA | 138 | Primary solid Tumor |
| TCGA-F4-6808-01A-11R-1839-07 | 7 | NA | 138 | Primary solid Tumor |
| TCGA-F4-6809-01A-11R-1839-07 | 7 | NA | 138 | Primary solid Tumor |
| TCGA-F4-6854-01A-11R-1928-07 | 7 | NA | 157 | Primary solid Tumor |
| TCGA-F4-6855-01A-11R-1928-07 | 7 | NA | 157 | Primary solid Tumor |
| TCGA-F4-6856-01A-11R-1928-07 | 7 | NA | 157 | Primary solid Tumor |
| TCGA-F4-6857-01A-11R-1928-07 | 7 | NA | 157 | Primary solid Tumor |
| TCGA-F5-6464-01A-11R-1736-07 | 7 | NA | 122 | Primary solid Tumor |
| TCGA-F5-6465-01A-11R-1736-07 | 7 | NA | 122 | Primary solid Tumor |
| TCGA-F5-6571-01A-12R-1830-07 | 7 | NA | 139 | Primary solid Tumor |
| TCGA-F5-6702-01A-11R-1830-07 | 7 | NA | 139 | Primary solid Tumor |
| TCGA-F5-6810-01A-11R-1830-07 | 7 | NA | 139 | Primary solid Tumor |
| TCGA-F5-6811-01A-11R-1830-07 | 7 | NA | 139 | Primary solid Tumor |
| TCGA-F5-6812-01A-11R-1830-07 | 7 | NA | 139 | Primary solid Tumor |
| TCGA-F5-6813-01A-11R-1830-07 | 7 | NA | 139 | Primary solid Tumor |
| TCGA-F5-6814-01A-31R-1928-07 | 7 | NA | 158 | Primary solid Tumor |
| TCGA-F5-6861-01A-11R-1928-07 | 7 | NA | 158 | Primary solid Tumor |
| TCGA-F5-6863-01A-11R-1928-07 | 7 | NA | 158 | Primary solid Tumor |
| TCGA-F5-6864-01A-11R-1928-07 | 7 | NA | 158 | Primary solid Tumor |
| TCGA-F7-7848-01A-11R-2132-07 | 2 | 4 | 190 | Primary solid Tumor |
| TCGA-F7-8298-01A-11R-2403-07 | 2 | NA | 241 | Primary solid Tumor |
| TCGA-F7-8489-01A-31R-2403-07 | 2 | NA | 241 | Primary solid Tumor |
| TCGA-F7-A50G-01A-11R-A266-07 | 2 | NA | 273 | Primary solid Tumor |
| TCGA-F7-A50I-01A-11R-A28V-07 | 2 | NA | 301 | Primary solid Tumor |
| TCGA-F7-A50J-01A-21R-A28V-07 | 2 | NA | 301 | Primary solid Tumor |
| TCGA-F7-A61S-01A-11R-A28V-07 | 2 | NA | 301 | Primary solid Tumor |
| TCGA-F7-A61V-01A-11R-A28V-07 | 2 | NA | 301 | Primary solid Tumor |
| TCGA-F7-A61W-01A-11R-A28V-07 | 2 | NA | 301 | Primary solid Tumor |
| TCGA-F7-A620-01A-11R-A28V-07 | 2 | NA | 301 | Primary solid Tumor |
| TCGA-F7-A622-01A-11R-A28V-07 | 2 | NA | 301 | Primary solid Tumor |
| TCGA-F7-A623-01A-11R-A28V-07 | 2 | NA | 301 | Primary solid Tumor |
| TCGA-F7-A624-01A-22R-A30B-07 | 3 | NA | 311 | Primary solid Tumor |
| TCGA-FD-A3B3-01A-12R-A206-07 | 2 | 4 | 192 | Primary solid Tumor |
| TCGA-FD-A3B4-01A-12R-A206-07 | 2 | 4 | 192 | Primary solid Tumor |
| TCGA-FD-A3B5-01A-11R-A20F-07 | 2 | 4 | 199 | Primary solid Tumor |
| TCGA-FD-A3B6-01A-21R-A20F-07 | 2 | 4 | 199 | Primary solid Tumor |
| TCGA-FD-A3B7-01A-31R-A20F-07 | 2 | 4 | 199 | Primary solid Tumor |
| TCGA-FD-A3B8-01A-31R-A20F-07 | 5 | 4 | 199 | Primary solid Tumor |
| TCGA-FD-A3N5-01A-11R-A21D-07 | 2 | 4 | 207 | Primary solid Tumor |
| TCGA-FD-A3N6-01A-11R-A21D-07 | 2 | 4 | 207 | Primary solid Tumor |
| TCGA-FD-A3NA-01A-11R-A21D-07 | 1 | 1 | 207 | Primary solid Tumor |
| TCGA-FD-A3SJ-01A-12R-A22U-07 | 1 | 1 | 235 | Primary solid Tumor |
| TCGA-FD-A3SL-01A-21R-A22U-07 | 1 | 1 | 235 | Primary solid Tumor |
| TCGA-FD-A3SM-01A-11R-A22U-07 | 1 | 1 | 235 | Primary solid Tumor |
| TCGA-FD-A3SN-01A-12R-A22U-07 | 1 | 1 | 235 | Primary solid Tumor |
| TCGA-FD-A3SO-01A-11R-A22U-07 | 2 | 4 | 235 | Primary solid Tumor |
| TCGA-FD-A3SP-01A-31R-A22U-07 | 3 | 4 | 235 | Primary solid Tumor |
| TCGA-FD-A3SQ-01A-21R-A22U-07 | 1 | 1 | 235 | Primary solid Tumor |
| TCGA-FD-A3SR-01A-11R-A22U-07 | 1 | 1 | 235 | Primary solid Tumor |
| TCGA-FD-A3SS-01A-12R-A22U-07 | 1 | 1 | 235 | Primary solid Tumor |
| TCGA-FD-A43N-01A-11R-A23W-07 | 1 | NA | 252 | Primary solid Tumor |
| TCGA-FD-A43P-01A-31R-A23W-07 | 1 | NA | 252 | Primary solid Tumor |
| TCGA-FD-A43S-01A-21R-A23W-07 | 1 | NA | 252 | Primary solid Tumor |
| TCGA-FD-A43U-01A-11R-A23W-07 | 1 | NA | 252 | Primary solid Tumor |
| TCGA-FD-A43X-01A-11R-A23W-07 | 1 | NA | 252 | Primary solid Tumor |
| TCGA-FD-A43Y-01A-21R-A26T-07 | 2 | NA | 279 | Primary solid Tumor |
| TCGA-FD-A5BR-01A-11R-A26T-07 | 1 | NA | 279 | Primary solid Tumor |
| TCGA-FD-A5BS-01A-21R-A26T-07 | 6 | NA | 279 | Primary solid Tumor |
| TCGA-FD-A5BT-01A-11R-A26T-07 | 2 | NA | 279 | Primary solid Tumor |
| TCGA-FD-A5BU-01A-31R-A26T-07 | 2 | NA | 279 | Primary solid Tumor |
| TCGA-FD-A5BV-01A-11R-A26T-07 | 1 | NA | 279 | Primary solid Tumor |
| TCGA-FD-A5BX-01A-11R-A26T-07 | 3 | NA | 279 | Primary solid Tumor |
| TCGA-FD-A5BY-01A-31R-A28M-07 | 1 | NA | 294 | Primary solid Tumor |
| TCGA-FD-A5BZ-01A-11R-A28M-07 | 1 | NA | 294 | Primary solid Tumor |
| TCGA-FD-A5C0-01A-11R-A28M-07 | 1 | NA | 294 | Primary solid Tumor |
| TCGA-FD-A5C1-01A-11R-A28M-07 | 2 | NA | 294 | Primary solid Tumor |
| TCGA-FD-A62N-01A-11R-A30C-07 | 2 | NA | 309 | Primary solid Tumor |
| TCGA-FD-A62O-01A-11R-A30C-07 | 1 | NA | 309 | Primary solid Tumor |
| TCGA-FD-A62P-01A-32R-A30C-07 | 1 | NA | 309 | Primary solid Tumor |
| TCGA-FD-A62S-01A-11R-A30C-07 | 2 | NA | 309 | Primary solid Tumor |
| TCGA-FD-A6TA-01A-12R-A33J-07 | 1 | NA | 337 | Primary solid Tumor |
| TCGA-FD-A6TB-01A-12R-A33J-07 | 1 | NA | 337 | Primary solid Tumor |
| TCGA-FD-A6TC-01A-21R-A33J-07 | 1 | NA | 337 | Primary solid Tumor |
| TCGA-FD-A6TD-01A-51R-A33J-07 | 2 | NA | 337 | Primary solid Tumor |
| TCGA-FD-A6TE-01A-12R-A33J-07 | 1 | NA | 337 | Primary solid Tumor |
| TCGA-FD-A6TF-01A-52R-A32O-07 | 1 | NA | 328 | Primary solid Tumor |
| TCGA-FD-A6TG-01A-11R-A32O-07 | 1 | NA | 328 | Primary solid Tumor |
| TCGA-FD-A6TH-01A-11R-A32O-07 | 1 | NA | 328 | Primary solid Tumor |
| TCGA-FD-A6TI-01A-11R-A32O-07 | 1 | NA | 328 | Primary solid Tumor |
| TCGA-FD-A6TK-01A-42R-A33J-07 | 2 | NA | 337 | Primary solid Tumor |
| TCGA-FI-A2CX-01A-11R-A17B-07 | 6 | 13 | 156 | Primary solid Tumor |
| TCGA-FI-A2CY-01A-11R-A17B-07 | 20 | 11 | 156 | Primary solid Tumor |
| TCGA-FI-A2D0-01A-11R-A17B-07 | 16 | 11 | 156 | Primary solid Tumor |
| TCGA-FI-A2D2-01A-11R-A17B-07 | 16 | 11 | 156 | Primary solid Tumor |
| TCGA-FI-A2D4-01A-12R-A17B-07 | 16 | 11 | 156 | Primary solid Tumor |
| TCGA-FI-A2D5-01A-11R-A17B-07 | 16 | 11 | 156 | Primary solid Tumor |
| TCGA-FI-A2D6-01A-11R-A17B-07 | 3 | 6 | 156 | Primary solid Tumor |
| TCGA-FI-A2EU-01A-11R-A17B-07 | 16 | 11 | 156 | Primary solid Tumor |
| TCGA-FI-A2EW-01A-11R-A17B-07 | 16 | 11 | 156 | Primary solid Tumor |
| TCGA-FI-A2EX-01A-11R-A17B-07 | 16 | 11 | 156 | Primary solid Tumor |
| TCGA-FI-A2EY-01A-12R-A18M-07 | 16 | NA | 178 | Primary solid Tumor |
| TCGA-FI-A2F4-01A-11R-A17B-07 | 16 | 11 | 156 | Primary solid Tumor |
| TCGA-FI-A2F8-01A-12R-A17B-07 | 16 | 11 | 156 | Primary solid Tumor |
| TCGA-FI-A2F9-01A-11R-A17B-07 | 16 | 11 | 156 | Primary solid Tumor |
| TCGA-FI-A3PV-01A-11R-A22K-07 | 16 | NA | 228 | Primary solid Tumor |
| TCGA-FI-A3PX-01A-11R-A22K-07 | 16 | NA | 228 | Primary solid Tumor |
| TCGA-FJ-A3Z7-01A-12R-A23N-07 | 1 | NA | 249 | Primary solid Tumor |
| TCGA-FJ-A3Z9-01A-11R-A26T-07 | 1 | NA | 279 | Primary solid Tumor |
| TCGA-FJ-A3ZE-01A-11R-A23N-07 | 1 | NA | 249 | Primary solid Tumor |
| TCGA-FJ-A3ZF-01A-11R-A23N-07 | 1 | NA | 249 | Primary solid Tumor |
| TCGA-FJ-A871-01A-11R-A352-07 | 3 | NA | 356 | Primary solid Tumor |
| TCGA-FT-A3EE-01A-11R-A206-07 | 1 | 1 | 192 | Primary solid Tumor |
| TCGA-FT-A61P-01A-11R-A30C-07 | 2 | NA | 309 | Primary solid Tumor |
| TCGA-G2-A2EC-01A-11R-A180-07 | 1 | 1 | 170 | Primary solid Tumor |
| TCGA-G2-A2EF-01A-12R-A18C-07 | 2 | 4 | 175 | Primary solid Tumor |
| TCGA-G2-A2EJ-01A-11R-A180-07 | 2 | 4 | 170 | Primary solid Tumor |
| TCGA-G2-A2EK-01A-22R-A18C-07 | 1 | 1 | 175 | Primary solid Tumor |
| TCGA-G2-A2EL-01A-12R-A18C-07 | 3 | 6 | 175 | Primary solid Tumor |
| TCGA-G2-A2EO-01A-11R-A180-07 | 1 | 1 | 170 | Primary solid Tumor |
| TCGA-G2-A2ES-01A-11R-A180-07 | 2 | 4 | 170 | Primary solid Tumor |
| TCGA-G2-A3IB-01A-11R-A20F-07 | 2 | 4 | 199 | Primary solid Tumor |
| TCGA-G2-A3IE-01A-11R-A20F-07 | 1 | 1 | 199 | Primary solid Tumor |
| TCGA-G2-A3VY-01A-11R-A22U-07 | 1 | 1 | 235 | Primary solid Tumor |
| TCGA-G2-AA3B-01A-11R-A39I-07 | 1 | NA | 401 | Primary solid Tumor |
| TCGA-G2-AA3C-01A-21R-A39I-07 | 3 | NA | 401 | Primary solid Tumor |
| TCGA-G2-AA3D-01A-11R-A39I-07 | 1 | NA | 401 | Primary solid Tumor |
| TCGA-G2-AA3F-01A-12R-A42T-07 | 1 | NA | 433 | Primary solid Tumor |
| TCGA-G4-6293-01A-11R-1723-07 | 7 | NA | 116 | Primary solid Tumor |
| TCGA-G4-6294-01A-11R-1774-07 | 7 | NA | 123 | Primary solid Tumor |
| TCGA-G4-6295-01A-11R-1723-07 | 7 | NA | 116 | Primary solid Tumor |
| TCGA-G4-6297-01A-11R-1723-07 | 7 | NA | 116 | Primary solid Tumor |
| TCGA-G4-6298-01A-11R-1723-07 | 7 | NA | 116 | Primary solid Tumor |
| TCGA-G4-6299-01A-11R-1774-07 | 7 | NA | 123 | Primary solid Tumor |
| TCGA-G4-6302-01A-11R-1723-07 | 7 | NA | 116 | Primary solid Tumor |
| TCGA-G4-6303-01A-11R-1774-07 | 7 | NA | 123 | Primary solid Tumor |
| TCGA-G4-6304-01A-11R-1928-07 | 7 | NA | 157 | Primary solid Tumor |
| TCGA-G4-6306-01A-11R-1774-07 | 7 | NA | 123 | Primary solid Tumor |
| TCGA-G4-6307-01A-11R-1723-07 | 7 | NA | 116 | Primary solid Tumor |
| TCGA-G4-6309-01A-21R-1839-07 | 7 | NA | 138 | Primary solid Tumor |
| TCGA-G4-6310-01A-11R-1723-07 | 7 | NA | 116 | Primary solid Tumor |
| TCGA-G4-6311-01A-11R-1723-07 | 7 | NA | 116 | Primary solid Tumor |
| TCGA-G4-6314-01A-11R-1723-07 | 7 | NA | 116 | Primary solid Tumor |
| TCGA-G4-6315-01A-11R-1723-07 | 7 | NA | 116 | Primary solid Tumor |
| TCGA-G4-6317-01A-11R-1723-07 | 7 | NA | 116 | Primary solid Tumor |
| TCGA-G4-6320-01A-11R-1723-07 | 7 | NA | 116 | Primary solid Tumor |
| TCGA-G4-6321-01A-11R-1723-07 | 7 | NA | 116 | Primary solid Tumor |
| TCGA-G4-6322-01A-11R-1723-07 | 7 | NA | 116 | Primary solid Tumor |
| TCGA-G4-6323-01A-11R-1723-07 | 7 | NA | 116 | Primary solid Tumor |
| TCGA-G4-6586-01A-11R-1774-07 | 7 | NA | 123 | Primary solid Tumor |
| TCGA-G4-6588-01A-11R-1774-07 | 7 | NA | 123 | Primary solid Tumor |
| TCGA-G4-6625-01A-21R-1774-07 | 7 | NA | 123 | Primary solid Tumor |
| TCGA-G4-6626-01A-11R-1774-07 | 7 | NA | 123 | Primary solid Tumor |
| TCGA-G4-6627-01A-11R-1774-07 | 7 | NA | 123 | Primary solid Tumor |
| TCGA-G4-6628-01A-11R-1839-07 | 7 | NA | 138 | Primary solid Tumor |
| TCGA-G5-6233-01A-11R-1736-07 | 7 | NA | 122 | Primary solid Tumor |
| TCGA-G5-6235-01A-11R-1736-07 | 7 | NA | 122 | Primary solid Tumor |
| TCGA-G5-6572-01A-11R-1830-07 | 7 | NA | 139 | Primary solid Tumor |
| TCGA-G5-6641-01A-11R-A32Z-07 | 7 | NA | 139 | Primary solid Tumor |
| TCGA-G6-A5PC-01A-11R-A33J-07 | 14 | NA | 340 | Primary solid Tumor |
| TCGA-G6-A8L6-01A-11R-A37O-07 | 14 | NA | 387 | Primary solid Tumor |
| TCGA-G6-A8L7-01A-11R-A37O-07 | 14 | NA | 387 | Primary solid Tumor |
| TCGA-G6-A8L8-01A-21R-A37O-07 | 14 | NA | 387 | Primary solid Tumor |
| TCGA-GC-A3BM-01A-11R-A22U-07 | 1 | 1 | 235 | Primary solid Tumor |
| TCGA-GC-A3I6-01A-11R-A20F-07 | 1 | 4 | 199 | Primary solid Tumor |
| TCGA-GC-A3OO-01A-11R-A22U-07 | 1 | 1 | 235 | Primary solid Tumor |
| TCGA-GC-A3RB-01A-12R-A220-07 | 1 | 1 | 223 | Primary solid Tumor |
| TCGA-GC-A3RC-01A-11R-A22U-07 | 2 | 4 | 235 | Primary solid Tumor |
| TCGA-GC-A3RD-01A-12R-A22U-07 | 1 | 1 | 235 | Primary solid Tumor |
| TCGA-GC-A3WC-01A-31R-A22U-07 | 2 | 4 | 235 | Primary solid Tumor |
| TCGA-GC-A3YS-01A-11R-A23N-07 | 2 | NA | 249 | Primary solid Tumor |
| TCGA-GC-A4ZW-01A-11R-A26T-07 | 1 | NA | 279 | Primary solid Tumor |
| TCGA-GC-A6I1-01A-12R-A31N-07 | 2 | NA | 317 | Primary solid Tumor |
| TCGA-GC-A6I3-01A-11R-A31N-07 | 1 | NA | 317 | Primary solid Tumor |
| TCGA-GD-A2C5-01A-12R-A180-07 | 1 | 1 | 170 | Primary solid Tumor |
| TCGA-GD-A3OP-01A-21R-A220-07 | 1 | 1 | 223 | Primary solid Tumor |
| TCGA-GD-A3OQ-01A-32R-A220-07 | 1 | 1 | 223 | Primary solid Tumor |
| TCGA-GD-A3OS-01A-12R-A220-07 | 3 | 6 | 223 | Primary solid Tumor |
| TCGA-GD-A6C6-01A-21R-A31N-07 | 1 | NA | 317 | Primary solid Tumor |
| TCGA-GD-A76B-01A-11R-A32O-07 | 1 | NA | 328 | Primary solid Tumor |
| TCGA-GI-A2C8-01A-11R-A16F-07 | 9 | 7 | 147 | Primary solid Tumor |
| TCGA-GI-A2C9-01A-11R-A21T-07 | 10 | 8 | 216 | Primary solid Tumor |
| TCGA-GK-A6C7-01A-11R-A33J-07 | 14 | NA | 340 | Primary solid Tumor |
| TCGA-GM-A2D9-01A-11R-A18M-07 | 9 | 7 | 177 | Primary solid Tumor |
| TCGA-GM-A2DA-01A-11R-A18M-07 | 9 | 7 | 177 | Primary solid Tumor |
| TCGA-GM-A2DB-01A-31R-A18M-07 | 12 | 7 | 177 | Primary solid Tumor |
| TCGA-GM-A2DC-01A-11R-A18M-07 | 9 | 7 | 177 | Primary solid Tumor |
| TCGA-GM-A2DD-01A-11R-A180-07 | 9 | 7 | 167 | Primary solid Tumor |
| TCGA-GM-A2DF-01A-11R-A180-07 | 10 | 8 | 167 | Primary solid Tumor |
| TCGA-GM-A2DH-01A-11R-A180-07 | 9 | 7 | 167 | Primary solid Tumor |
| TCGA-GM-A2DI-01A-31R-A18M-07 | 9 | 7 | 177 | Primary solid Tumor |
| TCGA-GM-A2DK-01A-21R-A180-07 | 9 | 7 | 167 | Primary solid Tumor |
| TCGA-GM-A2DL-01A-11R-A18M-07 | 9 | 7 | 177 | Primary solid Tumor |
| TCGA-GM-A2DM-01A-11R-A180-07 | 9 | 7 | 167 | Primary solid Tumor |
| TCGA-GM-A2DN-01A-11R-A180-07 | 9 | 7 | 167 | Primary solid Tumor |
| TCGA-GM-A2DO-01A-11R-A18M-07 | 9 | 7 | 177 | Primary solid Tumor |
| TCGA-GM-A3NW-01A-21R-A22K-07 | 9 | NA | 227 | Primary solid Tumor |
| TCGA-GM-A3NY-01A-11R-A21T-07 | 9 | 7 | 216 | Primary solid Tumor |
| TCGA-GM-A3XG-01A-31R-A24H-07 | 9 | NA | 255 | Primary solid Tumor |
| TCGA-GM-A3XL-01A-11R-A22U-07 | 10 | NA | 234 | Primary solid Tumor |
| TCGA-GM-A3XN-01A-12R-A22U-07 | 9 | NA | 234 | Primary solid Tumor |
| TCGA-GM-A4E0-01A-12R-A266-07 | 9 | NA | 271 | Primary solid Tumor |
| TCGA-GM-A5PV-01A-11R-A28M-07 | 9 | NA | 296 | Primary solid Tumor |
| TCGA-GM-A5PX-01A-12R-A28M-07 | 9 | NA | 296 | Primary solid Tumor |
| TCGA-GU-A42P-01A-11R-A23W-07 | 1 | NA | 252 | Primary solid Tumor |
| TCGA-GU-A42Q-01A-11R-A23W-07 | 2 | NA | 252 | Primary solid Tumor |
| TCGA-GU-A42R-01A-11R-A23N-07 | 1 | NA | 249 | Primary solid Tumor |
| TCGA-GU-A762-01A-11R-A33J-07 | 2 | NA | 337 | Primary solid Tumor |
| TCGA-GU-A763-01A-11R-A32O-07 | 1 | NA | 328 | Primary solid Tumor |
| TCGA-GU-A764-01A-11R-A352-07 | 2 | NA | 356 | Primary solid Tumor |
| TCGA-GU-A766-01A-11R-A32O-07 | 2 | NA | 328 | Primary solid Tumor |
| TCGA-GU-A767-01A-11R-A32O-07 | 1 | NA | 328 | Primary solid Tumor |
| TCGA-GU-AATO-01A-11R-A39I-07 | 1 | NA | 401 | Primary solid Tumor |
| TCGA-GU-AATP-01A-11R-A39I-07 | 1 | NA | 401 | Primary solid Tumor |
| TCGA-GU-AATQ-01A-11R-A39I-07 | 1 | NA | 401 | Primary solid Tumor |
| TCGA-GV-A3JV-01A-11R-A220-07 | 1 | 1 | 223 | Primary solid Tumor |
| TCGA-GV-A3JW-01A-11R-A20F-07 | 1 | 1 | 199 | Primary solid Tumor |
| TCGA-GV-A3JX-01A-11R-A20F-07 | 1 | 1 | 199 | Primary solid Tumor |
| TCGA-GV-A3JZ-01A-11R-A21D-07 | 1 | 1 | 207 | Primary solid Tumor |
| TCGA-GV-A3QF-01A-31R-A22U-07 | 1 | 1 | 235 | Primary solid Tumor |
| TCGA-GV-A3QG-01A-11R-A220-07 | 5 | 2 | 223 | Primary solid Tumor |
| TCGA-GV-A3QH-01A-11R-A220-07 | 1 | 1 | 223 | Primary solid Tumor |
| TCGA-GV-A3QI-01A-11R-A220-07 | 1 | 6 | 223 | Primary solid Tumor |
| TCGA-GV-A3QK-01B-11R-A23N-07 | 1 | NA | 249 | Primary solid Tumor |
| TCGA-GV-A40E-01A-12R-A23N-07 | 1 | NA | 249 | Primary solid Tumor |
| TCGA-GV-A40G-01A-11R-A23N-07 | 1 | NA | 249 | Primary solid Tumor |
| TCGA-GV-A6ZA-01A-12R-A33J-07 | 1 | NA | 337 | Primary solid Tumor |
| TCGA-H4-A2HO-01A-11R-A180-07 | 1 | 1 | 170 | Primary solid Tumor |
| TCGA-H4-A2HQ-01A-11R-A180-07 | 1 | 1 | 170 | Primary solid Tumor |
| TCGA-H5-A2HR-01A-11R-A180-07 | 16 | NA | 168 | Primary solid Tumor |
| TCGA-H7-7774-01A-21R-2081-07 | 2 | 4 | 188 | Primary solid Tumor |
| TCGA-H7-8501-01A-11R-2403-07 | 2 | NA | 241 | Primary solid Tumor |
| TCGA-H7-8502-01A-11R-2403-07 | 2 | NA | 241 | Primary solid Tumor |
| TCGA-H7-A6C4-01A-11R-A30B-07 | 2 | NA | 311 | Primary solid Tumor |
| TCGA-H7-A76A-01A-51R-A34R-07 | 2 | NA | 355 | Primary solid Tumor |
| TCGA-HD-7229-01A-11R-2016-07 | 2 | 4 | 164 | Primary solid Tumor |
| TCGA-HD-7753-01A-11R-2081-07 | 2 | 4 | 188 | Primary solid Tumor |
| TCGA-HD-7754-01A-11R-2081-07 | 2 | 4 | 188 | Primary solid Tumor |
| TCGA-HD-7831-01A-11R-2132-07 | 2 | 4 | 190 | Primary solid Tumor |
| TCGA-HD-7832-01A-11R-2132-07 | 2 | 4 | 190 | Primary solid Tumor |
| TCGA-HD-7917-01A-11R-2232-07 | 2 | 4 | 215 | Primary solid Tumor |
| TCGA-HD-8224-01A-11R-2403-07 | 2 | NA | 241 | Primary solid Tumor |
| TCGA-HD-8314-01A-11R-2403-07 | 2 | NA | 241 | Primary solid Tumor |
| TCGA-HD-8634-01A-11R-2403-07 | 2 | NA | 241 | Primary solid Tumor |
| TCGA-HD-8635-01A-11R-2403-07 | 2 | NA | 241 | Primary solid Tumor |
| TCGA-HD-A4C1-01A-11R-A24H-07 | 2 | NA | 260 | Primary solid Tumor |
| TCGA-HD-A633-01A-11R-A28V-07 | 2 | NA | 301 | Primary solid Tumor |
| TCGA-HD-A634-01A-11R-A28V-07 | 3 | NA | 301 | Primary solid Tumor |
| TCGA-HD-A6HZ-01A-12R-A31N-07 | 2 | NA | 318 | Primary solid Tumor |
| TCGA-HD-A6I0-01A-11R-A31N-07 | 2 | NA | 318 | Primary solid Tumor |
| TCGA-HL-7533-01A-11R-2232-07 | 2 | NA | 215 | Primary solid Tumor |
| TCGA-HN-A2NL-01A-11R-A18M-07 | 10 | 8 | 177 | Primary solid Tumor |
| TCGA-HN-A2OB-01A-21R-A27Q-07 | 9 | NA | 288 | Primary solid Tumor |
| TCGA-HQ-A2OE-01A-11R-A206-07 | 1 | 1 | 192 | Primary solid Tumor |
| TCGA-HQ-A2OF-01A-11R-A26T-07 | 7 | NA | 279 | Primary solid Tumor |
| TCGA-HQ-A5ND-01A-11R-A26T-07 | 1 | NA | 279 | Primary solid Tumor |
| TCGA-HQ-A5NE-01A-12R-A28M-07 | 1 | NA | 294 | Primary solid Tumor |
| TCGA-IQ-7630-01A-11R-2081-07 | 2 | 4 | 188 | Primary solid Tumor |
| TCGA-IQ-7631-01A-11R-2081-07 | 13 | 1 | 188 | Primary solid Tumor |
| TCGA-IQ-7632-01A-11R-2081-07 | 2 | 4 | 188 | Primary solid Tumor |
| TCGA-IQ-A61E-01A-22R-A30B-07 | 2 | NA | 311 | Primary solid Tumor |
| TCGA-IQ-A61G-01A-11R-A30B-07 | 2 | NA | 311 | Primary solid Tumor |
| TCGA-IQ-A61H-01A-11R-A30B-07 | 2 | NA | 311 | Primary solid Tumor |
| TCGA-IQ-A61I-01A-11R-A30B-07 | 2 | NA | 311 | Primary solid Tumor |
| TCGA-IQ-A61J-01A-11R-A30B-07 | 2 | NA | 311 | Primary solid Tumor |
| TCGA-IQ-A61O-01A-11R-A30B-07 | 3 | NA | 311 | Primary solid Tumor |
| TCGA-IQ-A6SG-01A-12R-A34R-07 | 2 | NA | 355 | Primary solid Tumor |
| TCGA-IQ-A6SH-01A-12R-A34R-07 | 2 | NA | 355 | Primary solid Tumor |
| TCGA-J1-A4AH-01A-31R-A24H-07 | 2 | NA | 259 | Primary solid Tumor |
| TCGA-J2-8192-01A-11R-2241-07 | 15 | 10 | 213 | Primary solid Tumor |
| TCGA-J2-8194-01A-11R-2241-07 | 15 | 10 | 213 | Primary solid Tumor |
| TCGA-J2-A4AD-01A-11R-A24H-07 | 15 | NA | 258 | Primary solid Tumor |
| TCGA-J2-A4AE-01A-21R-A24H-07 | 15 | NA | 258 | Primary solid Tumor |
| TCGA-J2-A4AG-01A-11R-A24H-07 | 15 | NA | 258 | Primary solid Tumor |
| TCGA-JL-A3YW-01A-12R-A239-07 | 9 | NA | 239 | Primary solid Tumor |
| TCGA-JL-A3YX-01A-11R-A22U-07 | 9 | NA | 234 | Primary solid Tumor |
| TCGA-JU-AAVI-01A-11R-A40A-07 | 16 | NA | 421 | Primary solid Tumor |
| TCGA-K4-A3WS-01A-11R-A22U-07 | 1 | 1 | 235 | Primary solid Tumor |
| TCGA-K4-A3WU-01B-11R-A23N-07 | 1 | NA | 249 | Primary solid Tumor |
| TCGA-K4-A3WV-01A-11R-A22U-07 | 2 | 4 | 235 | Primary solid Tumor |
| TCGA-K4-A4AB-01B-12R-A28M-07 | 1 | NA | 294 | Primary solid Tumor |
| TCGA-K4-A4AC-01A-21R-A26T-07 | 2 | NA | 279 | Primary solid Tumor |
| TCGA-K4-A54R-01A-11R-A26T-07 | 1 | NA | 279 | Primary solid Tumor |
| TCGA-K4-A5RH-01A-11R-A30C-07 | 3 | NA | 309 | Primary solid Tumor |
| TCGA-K4-A5RI-01A-11R-A28M-07 | 1 | NA | 294 | Primary solid Tumor |
| TCGA-K4-A5RJ-01A-11R-A28M-07 | 3 | NA | 294 | Primary solid Tumor |
| TCGA-K4-A6FZ-01A-11R-A31N-07 | 1 | NA | 317 | Primary solid Tumor |
| TCGA-K4-A6MB-01A-11R-A31N-07 | 1 | NA | 317 | Primary solid Tumor |
| TCGA-K4-A83P-01A-11R-A352-07 | 1 | NA | 356 | Primary solid Tumor |
| TCGA-K4-AAQO-01A-11R-A38B-07 | 1 | NA | 391 | Primary solid Tumor |
| TCGA-K6-A3WQ-01A-11R-A22K-07 | 16 | NA | 228 | Primary solid Tumor |
| TCGA-KJ-A3U4-01A-11R-A22K-07 | 20 | NA | 228 | Primary solid Tumor |
| TCGA-KP-A3VZ-01A-11R-A22K-07 | 16 | NA | 228 | Primary solid Tumor |
| TCGA-KP-A3W0-01A-21R-A22K-07 | 16 | NA | 228 | Primary solid Tumor |
| TCGA-KP-A3W1-01A-11R-A22K-07 | 16 | NA | 228 | Primary solid Tumor |
| TCGA-KP-A3W3-01A-11R-A22K-07 | 16 | NA | 228 | Primary solid Tumor |
| TCGA-KP-A3W4-01A-11R-A22K-07 | 16 | NA | 228 | Primary solid Tumor |
| TCGA-KQ-A41N-01A-11R-A33J-07 | 1 | NA | 337 | Primary solid Tumor |
| TCGA-KQ-A41O-01A-12R-A352-07 | 1 | NA | 356 | Primary solid Tumor |
| TCGA-KQ-A41P-01A-12R-A33J-07 | 1 | NA | 337 | Primary solid Tumor |
| TCGA-KQ-A41Q-01A-11R-A33J-07 | 1 | NA | 337 | Primary solid Tumor |
| TCGA-KQ-A41R-01A-21R-A352-07 | 1 | NA | 356 | Primary solid Tumor |
| TCGA-KQ-A41S-01A-12R-A33J-07 | 1 | NA | 337 | Primary solid Tumor |
| TCGA-KU-A66S-01A-21R-A30B-07 | 4 | NA | 311 | Primary solid Tumor |
| TCGA-KU-A66T-01A-11R-A30B-07 | 2 | NA | 311 | Primary solid Tumor |
| TCGA-KU-A6H7-01A-11R-A31N-07 | 2 | NA | 318 | Primary solid Tumor |
| TCGA-KU-A6H8-01A-21R-A34R-07 | 2 | NA | 355 | Primary solid Tumor |
| TCGA-L3-A4E7-01A-11R-A24Z-07 | 2 | NA | 267 | Primary solid Tumor |
| TCGA-L3-A524-01A-11R-A262-07 | 2 | NA | 276 | Primary solid Tumor |
| TCGA-L4-A4E5-01A-11R-A24X-07 | 15 | NA | 264 | Primary solid Tumor |
| TCGA-L4-A4E6-01A-11R-A24H-07 | 15 | NA | 258 | Primary solid Tumor |
| TCGA-L9-A443-01A-12R-A24H-07 | 15 | NA | 258 | Primary solid Tumor |
| TCGA-L9-A444-01A-21R-A24H-07 | 15 | NA | 258 | Primary solid Tumor |
| TCGA-L9-A50W-01A-12R-A39D-07 | 15 | NA | 406 | Primary solid Tumor |
| TCGA-L9-A5IP-01A-21R-A39D-07 | 15 | NA | 406 | Primary solid Tumor |
| TCGA-L9-A743-01A-43R-A39D-07 | 15 | NA | 406 | Primary solid Tumor |
| TCGA-L9-A7SV-01A-11R-A39D-07 | 15 | NA | 406 | Primary solid Tumor |
| TCGA-L9-A8F4-01A-11R-A39D-07 | 15 | NA | 406 | Primary solid Tumor |
| TCGA-LA-A446-01A-21R-A24Z-07 | 2 | NA | 267 | Primary solid Tumor |
| TCGA-LA-A7SW-01A-11R-A405-07 | 2 | NA | 415 | Primary solid Tumor |
| TCGA-LC-A66R-01A-41R-A30C-07 | 2 | NA | 309 | Primary solid Tumor |
| TCGA-LD-A66U-01A-11R-A31O-07 | 9 | NA | 322 | Primary solid Tumor |
| TCGA-LD-A74U-01A-13R-A33J-07 | 9 | NA | 338 | Primary solid Tumor |
| TCGA-LD-A7W5-01A-22R-A352-07 | 9 | NA | 360 | Primary solid Tumor |
| TCGA-LD-A7W6-01A-81R-A352-07 | 9 | NA | 360 | Primary solid Tumor |
| TCGA-LD-A9QF-01A-32R-A41B-07 | 9 | NA | 379 | Primary solid Tumor |
| TCGA-LL-A440-01A-11R-A24H-07 | 9 | NA | 255 | Primary solid Tumor |
| TCGA-LL-A441-01A-11R-A24H-07 | 10 | NA | 255 | Primary solid Tumor |
| TCGA-LL-A442-01A-11R-A24H-07 | 9 | NA | 255 | Primary solid Tumor |
| TCGA-LL-A50Y-01A-11R-A266-07 | 9 | NA | 271 | Primary solid Tumor |
| TCGA-LL-A5YL-01A-12R-A29R-07 | 9 | NA | 305 | Primary solid Tumor |
| TCGA-LL-A5YM-01A-11R-A28M-07 | 9 | NA | 296 | Primary solid Tumor |
| TCGA-LL-A5YN-01A-11R-A28M-07 | 9 | NA | 296 | Primary solid Tumor |
| TCGA-LL-A5YO-01A-21R-A28M-07 | 9 | NA | 296 | Primary solid Tumor |
| TCGA-LL-A5YP-01A-21R-A28M-07 | 10 | NA | 296 | Primary solid Tumor |
| TCGA-LL-A6FP-01A-11R-A31O-07 | 9 | NA | 322 | Primary solid Tumor |
| TCGA-LL-A6FQ-01A-11R-A31O-07 | 9 | NA | 322 | Primary solid Tumor |
| TCGA-LL-A6FR-01A-12R-A31O-07 | 10 | NA | 322 | Primary solid Tumor |
| TCGA-LL-A73Y-01A-11R-A33J-07 | 10 | NA | 338 | Primary solid Tumor |
| TCGA-LL-A73Z-01A-11R-A32P-07 | 9 | NA | 334 | Primary solid Tumor |
| TCGA-LL-A740-01A-21R-A32P-07 | 9 | NA | 334 | Primary solid Tumor |
| TCGA-LL-A7SZ-01A-32R-A352-07 | 9 | NA | 360 | Primary solid Tumor |
| TCGA-LL-A7T0-01A-31R-A352-07 | 9 | NA | 360 | Primary solid Tumor |
| TCGA-LL-A8F5-01A-11R-A36F-07 | 10 | NA | 372 | Primary solid Tumor |
| TCGA-LL-A9Q3-01A-11R-A41B-07 | 9 | NA | 379 | Primary solid Tumor |
| TCGA-LQ-A4E4-01A-11R-A266-07 | 9 | NA | 271 | Primary solid Tumor |
| TCGA-LT-A5Z6-01A-11R-A28M-07 | 1 | NA | 294 | Primary solid Tumor |
| TCGA-LT-A8JT-01A-11R-A36F-07 | 1 | NA | 367 | Primary solid Tumor |
| TCGA-MF-A522-01A-11R-A262-07 | 2 | NA | 276 | Primary solid Tumor |
| TCGA-MM-A563-01A-11R-A266-07 | 14 | NA | 274 | Primary solid Tumor |
| TCGA-MM-A564-01A-11R-A266-07 | 14 | NA | 274 | Primary solid Tumor |
| TCGA-MM-A84U-01A-11R-A37O-07 | 14 | NA | 387 | Primary solid Tumor |
| TCGA-MN-A4N1-01A-11R-A24X-07 | 15 | NA | 264 | Primary solid Tumor |
| TCGA-MN-A4N4-01A-12R-A24X-07 | 15 | NA | 264 | Primary solid Tumor |
| TCGA-MN-A4N5-01A-11R-A24X-07 | 15 | NA | 264 | Primary solid Tumor |
| TCGA-MP-A4SV-01A-11R-A24X-07 | 15 | NA | 264 | Primary solid Tumor |
| TCGA-MP-A4SW-01A-21R-A24X-07 | 15 | NA | 264 | Primary solid Tumor |
| TCGA-MP-A4SY-01A-21R-A24X-07 | 15 | NA | 264 | Primary solid Tumor |
| TCGA-MP-A4T4-01A-11R-A262-07 | 15 | NA | 278 | Primary solid Tumor |
| TCGA-MP-A4T6-01A-32R-A262-07 | 15 | NA | 278 | Primary solid Tumor |
| TCGA-MP-A4T7-01A-11R-A24X-07 | 15 | NA | 264 | Primary solid Tumor |
| TCGA-MP-A4T8-01A-11R-A24X-07 | 15 | NA | 264 | Primary solid Tumor |
| TCGA-MP-A4T9-01A-11R-A24X-07 | 15 | NA | 264 | Primary solid Tumor |
| TCGA-MP-A4TA-01A-21R-A24X-07 | 15 | NA | 264 | Primary solid Tumor |
| TCGA-MP-A4TC-01A-11R-A24X-07 | 15 | NA | 264 | Primary solid Tumor |
| TCGA-MP-A4TD-01A-32R-A262-07 | 15 | NA | 278 | Primary solid Tumor |
| TCGA-MP-A4TE-01A-22R-A466-07 | 15 | NA | 278 | Primary solid Tumor |
| TCGA-MP-A4TF-01A-11R-A262-07 | 15 | NA | 278 | Primary solid Tumor |
| TCGA-MP-A4TH-01A-31R-A262-07 | 15 | NA | 278 | Primary solid Tumor |
| TCGA-MP-A4TI-01A-21R-A24X-07 | 15 | NA | 264 | Primary solid Tumor |
| TCGA-MP-A4TJ-01A-51R-A262-07 | 15 | NA | 278 | Primary solid Tumor |
| TCGA-MP-A4TK-01A-11R-A24X-07 | 15 | NA | 264 | Primary solid Tumor |
| TCGA-MP-A5C7-01A-11R-A262-07 | 15 | NA | 278 | Primary solid Tumor |
| TCGA-MS-A51U-01A-31R-A266-07 | 9 | NA | 271 | Primary solid Tumor |
| TCGA-MT-A51W-01A-21R-A266-07 | 2 | NA | 273 | Primary solid Tumor |
| TCGA-MT-A51X-01A-11R-A266-07 | 2 | NA | 273 | Primary solid Tumor |
| TCGA-MT-A67A-01A-11R-A30B-07 | 2 | NA | 311 | Primary solid Tumor |
| TCGA-MT-A67D-01A-31R-A30B-07 | 2 | NA | 311 | Primary solid Tumor |
| TCGA-MT-A67F-01A-11R-A30B-07 | 2 | NA | 311 | Primary solid Tumor |
| TCGA-MT-A7BN-01A-12R-A34R-07 | 11 | NA | 355 | Primary solid Tumor |
| TCGA-MV-A51V-01A-11R-A26T-07 | 1 | NA | 279 | Primary solid Tumor |
| TCGA-MW-A4EC-01A-11R-A266-07 | 14 | NA | 274 | Primary solid Tumor |
| TCGA-MZ-A5BI-01A-31R-A34R-07 | 2 | NA | 355 | Primary solid Tumor |
| TCGA-MZ-A6I9-01A-11R-A31N-07 | 2 | NA | 318 | Primary solid Tumor |
| TCGA-MZ-A7D7-01A-21R-A34R-07 | 2 | NA | 355 | Primary solid Tumor |
| TCGA-NC-A5HD-01A-11R-A26W-07 | 2 | NA | 283 | Primary solid Tumor |
| TCGA-NC-A5HE-01A-11R-A26W-07 | 2 | NA | 283 | Primary solid Tumor |
| TCGA-NC-A5HF-01A-11R-A26W-07 | 2 | NA | 283 | Primary solid Tumor |
| TCGA-NC-A5HG-01A-11R-A26W-07 | 2 | NA | 283 | Primary solid Tumor |
| TCGA-NC-A5HH-01A-11R-A26W-07 | 2 | NA | 283 | Primary solid Tumor |
| TCGA-NC-A5HI-01A-11R-A26W-07 | 2 | NA | 283 | Primary solid Tumor |
| TCGA-NC-A5HJ-01A-11R-A26W-07 | 15 | NA | 283 | Primary solid Tumor |
| TCGA-NC-A5HK-01A-11R-A26W-07 | 2 | NA | 283 | Primary solid Tumor |
| TCGA-NC-A5HL-01A-11R-A26W-07 | 2 | NA | 283 | Primary solid Tumor |
| TCGA-NC-A5HM-01A-12R-A26W-07 | 2 | NA | 283 | Primary solid Tumor |
| TCGA-NC-A5HN-01A-11R-A26W-07 | 2 | NA | 283 | Primary solid Tumor |
| TCGA-NC-A5HO-01A-11R-A26W-07 | 2 | NA | 283 | Primary solid Tumor |
| TCGA-NC-A5HP-01A-11R-A26W-07 | 2 | NA | 283 | Primary solid Tumor |
| TCGA-NC-A5HQ-01A-11R-A26W-07 | 2 | NA | 283 | Primary solid Tumor |
| TCGA-NC-A5HR-01A-21R-A26W-07 | 2 | NA | 283 | Primary solid Tumor |
| TCGA-NC-A5HT-01A-11R-A26W-07 | 2 | NA | 283 | Primary solid Tumor |
| TCGA-NH-A50T-01A-11R-A28H-07 | 7 | NA | 300 | Primary solid Tumor |
| TCGA-NH-A50U-01A-33R-A37K-07 | 7 | NA | 385 | Primary solid Tumor |
| TCGA-NH-A50V-01A-11R-A28H-07 | 7 | NA | 300 | Primary solid Tumor |
| TCGA-NH-A5IV-01A-42R-A37K-07 | 7 | NA | 385 | Primary solid Tumor |
| TCGA-NH-A6GA-01A-11R-A37K-07 | 7 | NA | 385 | Primary solid Tumor |
| TCGA-NH-A6GB-01A-11R-A37K-07 | 7 | NA | 385 | Primary solid Tumor |
| TCGA-NH-A6GC-01A-12R-A41B-07 | 7 | NA | 422 | Primary solid Tumor |
| TCGA-NH-A8F7-01A-11R-A41B-07 | 7 | NA | 422 | Primary solid Tumor |
| TCGA-NH-A8F8-01A-72R-A41B-07 | 7 | NA | 422 | Primary solid Tumor |
| TCGA-NJ-A4YF-01A-12R-A262-07 | 15 | NA | 278 | Primary solid Tumor |
| TCGA-NJ-A4YG-01A-22R-A262-07 | 15 | NA | 278 | Primary solid Tumor |
| TCGA-NJ-A4YI-01A-11R-A262-07 | 15 | NA | 278 | Primary solid Tumor |
| TCGA-NJ-A4YP-01A-11R-A262-07 | 15 | NA | 278 | Primary solid Tumor |
| TCGA-NJ-A4YQ-01A-11R-A262-07 | 15 | NA | 278 | Primary solid Tumor |
| TCGA-NJ-A55A-01A-11R-A262-07 | 15 | NA | 278 | Primary solid Tumor |
| TCGA-NJ-A55O-01A-11R-A262-07 | 15 | NA | 278 | Primary solid Tumor |
| TCGA-NJ-A55R-01A-11R-A262-07 | 15 | NA | 278 | Primary solid Tumor |
| TCGA-NJ-A7XG-01A-12R-A39D-07 | 15 | NA | 406 | Primary solid Tumor |
| TCGA-NK-A5CR-01A-11R-A26W-07 | 2 | NA | 283 | Primary solid Tumor |
| TCGA-NK-A5CT-01A-31R-A26W-07 | 2 | NA | 283 | Primary solid Tumor |
| TCGA-NK-A5CX-01A-11R-A26W-07 | 2 | NA | 283 | Primary solid Tumor |
| TCGA-NK-A5D1-01A-11R-A26W-07 | 3 | NA | 283 | Primary solid Tumor |
| TCGA-NK-A7XE-01A-12R-A405-07 | 2 | NA | 415 | Primary solid Tumor |
| TCGA-O1-A52J-01A-11R-A262-07 | 15 | NA | 278 | Primary solid Tumor |
| TCGA-O2-A52N-01A-11R-A26W-07 | 2 | NA | 283 | Primary solid Tumor |
| TCGA-O2-A52Q-01A-11R-A26W-07 | 15 | NA | 283 | Primary solid Tumor |
| TCGA-O2-A52S-01A-11R-A262-07 | 2 | NA | 276 | Primary solid Tumor |
| TCGA-O2-A52V-01A-31R-A262-07 | 2 | NA | 276 | Primary solid Tumor |
| TCGA-O2-A52W-01A-11R-A26W-07 | 2 | NA | 283 | Primary solid Tumor |
| TCGA-O2-A5IB-01A-11R-A27Q-07 | 8 | NA | 293 | Primary solid Tumor |
| TCGA-OK-A5Q2-01A-11R-A27Q-07 | 9 | NA | 288 | Primary solid Tumor |
| TCGA-OL-A5D6-01A-21R-A27Q-07 | 9 | NA | 288 | Primary solid Tumor |
| TCGA-OL-A5D7-01A-11R-A27Q-07 | 10 | NA | 288 | Primary solid Tumor |
| TCGA-OL-A5D8-01A-11R-A27Q-07 | 9 | NA | 288 | Primary solid Tumor |
| TCGA-OL-A5DA-01A-11R-A27Q-07 | 9 | NA | 288 | Primary solid Tumor |
| TCGA-OL-A5RU-01A-11R-A28M-07 | 9 | NA | 296 | Primary solid Tumor |
| TCGA-OL-A5RV-01A-12R-A28M-07 | 9 | NA | 296 | Primary solid Tumor |
| TCGA-OL-A5RW-01A-11R-A28M-07 | 10 | NA | 296 | Primary solid Tumor |
| TCGA-OL-A5RX-01A-11R-A28M-07 | 9 | NA | 296 | Primary solid Tumor |
| TCGA-OL-A5RY-01A-21R-A28M-07 | 9 | NA | 296 | Primary solid Tumor |
| TCGA-OL-A5RZ-01A-11R-A28M-07 | 9 | NA | 296 | Primary solid Tumor |
| TCGA-OL-A5S0-01A-11R-A28M-07 | 10 | NA | 296 | Primary solid Tumor |
| TCGA-OL-A66H-01A-11R-A29R-07 | 9 | NA | 305 | Primary solid Tumor |
| TCGA-OL-A66I-01A-21R-A29R-07 | 10 | NA | 305 | Primary solid Tumor |
| TCGA-OL-A66J-01A-11R-A29R-07 | 9 | NA | 305 | Primary solid Tumor |
| TCGA-OL-A66K-01A-11R-A29R-07 | 9 | NA | 305 | Primary solid Tumor |
| TCGA-OL-A66L-01A-12R-A31O-07 | 9 | NA | 322 | Primary solid Tumor |
| TCGA-OL-A66N-01A-12R-A31O-07 | 9 | NA | 322 | Primary solid Tumor |
| TCGA-OL-A66O-01A-11R-A31O-07 | 9 | NA | 322 | Primary solid Tumor |
| TCGA-OL-A66P-01A-11R-A31O-07 | 9 | NA | 322 | Primary solid Tumor |
| TCGA-OL-A6VO-01A-12R-A33J-07 | 10 | NA | 338 | Primary solid Tumor |
| TCGA-OL-A6VQ-01A-12R-A41B-07 | 9 | NA | 379 | Primary solid Tumor |
| TCGA-OL-A6VR-01A-32R-A33J-07 | 9 | NA | 338 | Primary solid Tumor |
| TCGA-OL-A97C-01A-32R-A41B-07 | 3 | NA | 379 | Primary solid Tumor |
| TCGA-OY-A56P-01A-12R-A406-31 | 20 | NA | 409 | Primary solid Tumor |
| TCGA-OY-A56Q-01A-11R-A406-31 | 20 | NA | 409 | Primary solid Tumor |
| TCGA-P3-A5Q5-01A-11R-A28V-07 | 2 | NA | 301 | Primary solid Tumor |
| TCGA-P3-A5Q6-01A-11R-A28V-07 | 2 | NA | 301 | Primary solid Tumor |
| TCGA-P3-A5QA-01A-11R-A28V-07 | 2 | NA | 301 | Primary solid Tumor |
| TCGA-P3-A5QE-01A-11R-A28V-07 | 2 | NA | 301 | Primary solid Tumor |
| TCGA-P3-A5QF-01A-11R-A28V-07 | 2 | NA | 301 | Primary solid Tumor |
| TCGA-P3-A6SW-01A-11R-A34R-07 | 2 | NA | 355 | Primary solid Tumor |
| TCGA-P3-A6SX-01A-11R-A34R-07 | 2 | NA | 355 | Primary solid Tumor |
| TCGA-P3-A6T0-01A-12R-A34R-07 | 2 | NA | 355 | Primary solid Tumor |
| TCGA-P3-A6T2-01A-11R-A34R-07 | 2 | NA | 355 | Primary solid Tumor |
| TCGA-P3-A6T3-01A-11R-A34R-07 | 2 | NA | 355 | Primary solid Tumor |
| TCGA-P3-A6T4-01A-11R-A34R-07 | 2 | NA | 355 | Primary solid Tumor |
| TCGA-P3-A6T5-01A-11R-A34R-07 | 2 | NA | 355 | Primary solid Tumor |
| TCGA-P3-A6T6-01A-11R-A34R-07 | 2 | NA | 355 | Primary solid Tumor |
| TCGA-P3-A6T7-01A-11R-A34R-07 | 2 | NA | 355 | Primary solid Tumor |
| TCGA-P3-A6T8-01A-11R-A34R-07 | 2 | NA | 355 | Primary solid Tumor |
| TCGA-PE-A5DC-01A-12R-A27Q-07 | 9 | NA | 288 | Primary solid Tumor |
| TCGA-PE-A5DD-01A-12R-A27Q-07 | 9 | NA | 288 | Primary solid Tumor |
| TCGA-PE-A5DE-01A-11R-A27Q-07 | 9 | NA | 288 | Primary solid Tumor |
| TCGA-PG-A5BC-01A-12R-A27V-07 | 16 | NA | 289 | Primary solid Tumor |
| TCGA-PG-A6IB-01A-21R-A31O-07 | 3 | NA | 324 | Primary solid Tumor |
| TCGA-PG-A7D5-01A-11R-A34R-07 | 16 | NA | 354 | Primary solid Tumor |
| TCGA-PG-A914-01A-11R-A37O-07 | 16 | NA | 381 | Primary solid Tumor |
| TCGA-PG-A915-01A-11R-A37O-07 | 16 | NA | 381 | Primary solid Tumor |
| TCGA-PG-A916-01A-11R-A37O-07 | 16 | NA | 381 | Primary solid Tumor |
| TCGA-PG-A917-01A-31R-A37O-07 | 16 | NA | 381 | Primary solid Tumor |
| TCGA-PL-A8LV-01A-21R-A41B-07 | 10 | NA | 379 | Primary solid Tumor |
| TCGA-PL-A8LX-01A-11R-A41B-07 | 9 | NA | 379 | Primary solid Tumor |
| TCGA-PL-A8LY-01A-11R-A41B-07 | 9 | NA | 379 | Primary solid Tumor |
| TCGA-PL-A8LZ-01A-31R-A36F-07 | 10 | NA | 372 | Primary solid Tumor |
| TCGA-PQ-A6FI-01A-11R-A31N-07 | 2 | NA | 317 | Primary solid Tumor |
| TCGA-PQ-A6FN-01A-11R-A31N-07 | 2 | NA | 317 | Primary solid Tumor |
| TCGA-QF-A5YS-01A-11R-A31O-07 | 16 | NA | 324 | Primary solid Tumor |
| TCGA-QF-A5YT-01A-11R-A31O-07 | 16 | NA | 324 | Primary solid Tumor |
| TCGA-QG-A5YV-01A-11R-A28H-07 | 7 | NA | 300 | Primary solid Tumor |
| TCGA-QG-A5YW-01A-11R-A28H-07 | 7 | NA | 300 | Primary solid Tumor |
| TCGA-QG-A5YX-01A-11R-A28H-07 | 7 | NA | 300 | Primary solid Tumor |
| TCGA-QG-A5Z1-01A-11R-A28H-07 | 7 | NA | 300 | Primary solid Tumor |
| TCGA-QG-A5Z2-01A-11R-A28H-07 | 7 | NA | 300 | Primary solid Tumor |
| TCGA-QK-A64Z-01A-11R-A30B-07 | 2 | NA | 311 | Primary solid Tumor |
| TCGA-QK-A652-01A-11R-A30B-07 | 2 | NA | 311 | Primary solid Tumor |
| TCGA-QK-A6IF-01A-11R-A31N-07 | 2 | NA | 318 | Primary solid Tumor |
| TCGA-QK-A6IG-01A-11R-A31N-07 | 2 | NA | 318 | Primary solid Tumor |
| TCGA-QK-A6IH-01A-11R-A31N-07 | 2 | NA | 318 | Primary solid Tumor |
| TCGA-QK-A6II-01A-11R-A31N-07 | 2 | NA | 318 | Primary solid Tumor |
| TCGA-QK-A6IJ-01A-11R-A31N-07 | 2 | NA | 318 | Primary solid Tumor |
| TCGA-QK-A6V9-01A-11R-A34R-07 | 2 | NA | 355 | Primary solid Tumor |
| TCGA-QK-A6VB-01A-12R-A34R-07 | 2 | NA | 355 | Primary solid Tumor |
| TCGA-QK-A6VC-01A-23R-A34R-07 | 2 | NA | 355 | Primary solid Tumor |
| TCGA-QK-A8Z7-01A-11R-A39I-07 | 2 | NA | 403 | Primary solid Tumor |
| TCGA-QK-A8Z8-01A-11R-A39I-07 | 2 | NA | 403 | Primary solid Tumor |
| TCGA-QK-A8Z9-01B-11R-A39I-07 | 2 | NA | 403 | Primary solid Tumor |
| TCGA-QK-A8ZA-01A-11R-A39I-07 | 2 | NA | 403 | Primary solid Tumor |
| TCGA-QK-A8ZB-01A-11R-A39I-07 | 2 | NA | 403 | Primary solid Tumor |
| TCGA-QK-AA3J-01A-11R-A39I-07 | 2 | NA | 403 | Primary solid Tumor |
| TCGA-QK-AA3K-01A-11R-A39I-07 | 3 | NA | 403 | Primary solid Tumor |
| TCGA-QL-A97D-01A-12R-A41B-07 | 7 | NA | 422 | Primary solid Tumor |
| TCGA-QS-A5YQ-01A-11R-A31O-07 | 16 | NA | 324 | Primary solid Tumor |
| TCGA-QS-A5YR-01A-31R-A31O-07 | 16 | NA | 324 | Primary solid Tumor |
| TCGA-QS-A744-01A-11R-A34R-07 | 16 | NA | 354 | Primary solid Tumor |
| TCGA-QS-A8F1-01A-21R-A37O-07 | 16 | NA | 381 | Primary solid Tumor |
| TCGA-R3-A69X-01A-22R-A30C-07 | 1 | NA | 309 | Primary solid Tumor |
| TCGA-RS-A6TO-01A-32R-A34R-07 | 2 | NA | 355 | Primary solid Tumor |
| TCGA-RS-A6TP-01A-12R-A34R-07 | 2 | NA | 355 | Primary solid Tumor |
| TCGA-RU-A8FL-01A-11R-A37K-07 | 7 | NA | 385 | Primary solid Tumor |
| TCGA-S2-AA1A-01A-12R-A39D-07 | 15 | NA | 406 | Primary solid Tumor |
| TCGA-S3-A6ZF-01A-32R-A32P-07 | 9 | NA | 334 | Primary solid Tumor |
| TCGA-S3-A6ZG-01A-22R-A32P-07 | 9 | NA | 334 | Primary solid Tumor |
| TCGA-S3-A6ZH-01A-22R-A32P-07 | 9 | NA | 334 | Primary solid Tumor |
| TCGA-S3-AA0Z-01A-11R-A41B-07 | 10 | NA | 379 | Primary solid Tumor |
| TCGA-S3-AA10-01A-21R-A41B-07 | 10 | NA | 379 | Primary solid Tumor |
| TCGA-S3-AA11-01A-31R-A41B-07 | 9 | NA | 379 | Primary solid Tumor |
| TCGA-S3-AA12-01A-11R-A41B-07 | 9 | NA | 379 | Primary solid Tumor |
| TCGA-S3-AA14-01A-11R-A41B-07 | 9 | NA | 379 | Primary solid Tumor |
| TCGA-S3-AA15-01A-11R-A41B-07 | 10 | NA | 379 | Primary solid Tumor |
| TCGA-S3-AA17-01A-11R-A41B-07 | 9 | NA | 379 | Primary solid Tumor |
| TCGA-S5-A6DX-01A-11R-A31N-07 | 1 | NA | 317 | Primary solid Tumor |
| TCGA-S5-AA26-01A-11R-A38B-07 | 1 | NA | 391 | Primary solid Tumor |
| TCGA-SJ-A6ZI-01A-12R-A34R-07 | 16 | NA | 354 | Primary solid Tumor |
| TCGA-SJ-A6ZJ-01A-12R-A34R-07 | 16 | NA | 354 | Primary solid Tumor |
| TCGA-SL-A6J9-01A-11R-A31O-07 | 20 | NA | 324 | Primary solid Tumor |
| TCGA-SL-A6JA-01A-11R-A31O-07 | 16 | NA | 324 | Primary solid Tumor |
| TCGA-SS-A7HO-01A-21R-A37K-07 | 7 | NA | 385 | Primary solid Tumor |
| TCGA-SY-A9G0-01A-12R-A38B-07 | 1 | NA | 391 | Primary solid Tumor |
| TCGA-SY-A9G5-01A-11R-A38B-07 | 2 | NA | 391 | Primary solid Tumor |
| TCGA-T2-A6WX-01A-12R-A34R-07 | 2 | NA | 355 | Primary solid Tumor |
| TCGA-T2-A6WZ-01A-21R-A34R-07 | 2 | NA | 355 | Primary solid Tumor |
| TCGA-T2-A6X0-01A-11R-A34R-07 | 2 | NA | 355 | Primary solid Tumor |
| TCGA-T2-A6X2-01A-12R-A34R-07 | 2 | NA | 355 | Primary solid Tumor |
| TCGA-T3-A92M-01A-31R-A39I-07 | 2 | NA | 403 | Primary solid Tumor |
| TCGA-T3-A92N-01A-11R-A39I-07 | 2 | NA | 403 | Primary solid Tumor |
| TCGA-T7-A92I-01A-11R-A37O-07 | 14 | NA | 387 | Primary solid Tumor |
| TCGA-T9-A92H-01A-11R-A37K-07 | 7 | NA | 385 | Primary solid Tumor |
| TCGA-TN-A7HI-01A-11R-A34R-07 | 2 | NA | 355 | Primary solid Tumor |
| TCGA-TN-A7HJ-01A-12R-A34R-07 | 2 | NA | 355 | Primary solid Tumor |
| TCGA-TN-A7HL-01A-11R-A34R-07 | 2 | NA | 355 | Primary solid Tumor |
| TCGA-UF-A718-01A-22R-A34R-07 | 2 | NA | 355 | Primary solid Tumor |
| TCGA-UF-A719-01A-12R-A34R-07 | 2 | NA | 355 | Primary solid Tumor |
| TCGA-UF-A71A-01A-22R-A34R-07 | 2 | NA | 355 | Primary solid Tumor |
| TCGA-UF-A71B-01A-12R-A34R-07 | 2 | NA | 355 | Primary solid Tumor |
| TCGA-UF-A71D-01A-12R-A34R-07 | 2 | NA | 355 | Primary solid Tumor |
| TCGA-UF-A71E-01A-31R-A34R-07 | 2 | NA | 355 | Primary solid Tumor |
| TCGA-UF-A7J9-01A-12R-A34R-07 | 2 | NA | 355 | Primary solid Tumor |
| TCGA-UF-A7JA-01A-12R-A34R-07 | 2 | NA | 355 | Primary solid Tumor |
| TCGA-UF-A7JC-01A-21R-A34R-07 | 2 | NA | 355 | Primary solid Tumor |
| TCGA-UF-A7JD-01A-11R-A34R-07 | 2 | NA | 355 | Primary solid Tumor |
| TCGA-UF-A7JF-01A-11R-A34R-07 | 2 | NA | 355 | Primary solid Tumor |
| TCGA-UF-A7JH-01A-21R-A34R-07 | 2 | NA | 355 | Primary solid Tumor |
| TCGA-UF-A7JJ-01A-11R-A34R-07 | 2 | NA | 355 | Primary solid Tumor |
| TCGA-UF-A7JK-01A-11R-A34R-07 | 2 | NA | 355 | Primary solid Tumor |
| TCGA-UF-A7JO-01A-11R-A34R-07 | 2 | NA | 355 | Primary solid Tumor |
| TCGA-UF-A7JS-01A-11R-A34R-07 | 2 | NA | 355 | Primary solid Tumor |
| TCGA-UF-A7JT-01A-11R-A34R-07 | 2 | NA | 355 | Primary solid Tumor |
| TCGA-UF-A7JV-01A-11R-A34R-07 | 2 | NA | 355 | Primary solid Tumor |
| TCGA-UL-AAZ6-01A-11R-A41B-07 | 9 | NA | 379 | Primary solid Tumor |
| TCGA-UP-A6WW-01A-12R-A34R-07 | 2 | NA | 355 | Primary solid Tumor |
| TCGA-UU-A93S-01A-21R-A41B-07 | 12 | NA | 379 | Primary solid Tumor |
| TCGA-UY-A78K-01A-11R-A33J-07 | 1 | NA | 337 | Primary solid Tumor |
| TCGA-UY-A78L-01A-12R-A33J-07 | 1 | NA | 337 | Primary solid Tumor |
| TCGA-UY-A78M-01A-21R-A352-07 | 1 | NA | 356 | Primary solid Tumor |
| TCGA-UY-A78N-01A-12R-A33J-07 | 1 | NA | 337 | Primary solid Tumor |
| TCGA-UY-A78O-01A-12R-A33J-07 | 1 | NA | 337 | Primary solid Tumor |
| TCGA-UY-A78P-01A-12R-A36F-07 | 2 | NA | 367 | Primary solid Tumor |
| TCGA-UY-A8OB-01A-12R-A42T-07 | 1 | NA | 433 | Primary solid Tumor |
| TCGA-UY-A8OC-01A-11R-A36F-07 | 2 | NA | 367 | Primary solid Tumor |
| TCGA-UY-A8OD-01A-11R-A36F-07 | 1 | NA | 367 | Primary solid Tumor |
| TCGA-UY-A9PA-01A-11R-A38B-07 | 1 | NA | 391 | Primary solid Tumor |
| TCGA-UY-A9PB-01A-11R-A38B-07 | 2 | NA | 391 | Primary solid Tumor |
| TCGA-UY-A9PD-01A-11R-A38B-07 | 1 | NA | 391 | Primary solid Tumor |
| TCGA-UY-A9PE-01A-11R-A38B-07 | 1 | NA | 391 | Primary solid Tumor |
| TCGA-UY-A9PF-01A-11R-A38B-07 | 1 | NA | 391 | Primary solid Tumor |
| TCGA-UY-A9PH-01A-11R-A38B-07 | 1 | NA | 391 | Primary solid Tumor |
| TCGA-V7-A7HQ-01A-11R-A33J-07 | 9 | NA | 338 | Primary solid Tumor |
| TCGA-VG-A8LO-01A-11R-A406-31 | 20 | NA | 409 | Primary solid Tumor |
| TCGA-W8-A86G-01A-21R-A36F-07 | 9 | NA | 372 | Primary solid Tumor |
| TCGA-WA-A7GZ-01A-11R-A34R-07 | 2 | NA | 355 | Primary solid Tumor |
| TCGA-WA-A7H4-01A-21R-A34R-07 | 2 | NA | 355 | Primary solid Tumor |
| TCGA-WR-A838-01A-12R-A406-31 | 20 | NA | 409 | Primary solid Tumor |
| TCGA-WS-AB45-01A-11R-A41B-07 | 7 | NA | 422 | Primary solid Tumor |
| TCGA-WT-AB41-01A-11R-A41B-07 | 9 | NA | 379 | Primary solid Tumor |
| TCGA-WT-AB44-01A-11R-A41B-07 | 9 | NA | 379 | Primary solid Tumor |
| TCGA-XC-AA0X-01A-32R-A405-07 | 2 | NA | 415 | Primary solid Tumor |
| TCGA-XF-A8HB-01A-11R-A36F-07 | 1 | NA | 367 | Primary solid Tumor |
| TCGA-XF-A8HC-01A-11R-A36F-07 | 1 | NA | 367 | Primary solid Tumor |
| TCGA-XF-A8HD-01A-11R-A36F-07 | 1 | NA | 367 | Primary solid Tumor |
| TCGA-XF-A8HE-01A-11R-A36F-07 | 2 | NA | 367 | Primary solid Tumor |
| TCGA-XF-A8HF-01A-11R-A36F-07 | 1 | NA | 367 | Primary solid Tumor |
| TCGA-XF-A8HG-01A-11R-A36F-07 | 1 | NA | 367 | Primary solid Tumor |
| TCGA-XF-A8HH-01A-11R-A38B-07 | 1 | NA | 391 | Primary solid Tumor |
| TCGA-XF-A8HI-01A-11R-A38B-07 | 1 | NA | 391 | Primary solid Tumor |
| TCGA-XF-A9SH-01A-11R-A39I-07 | 1 | NA | 401 | Primary solid Tumor |
| TCGA-XF-A9SI-01A-11R-A39I-07 | 6 | NA | 401 | Primary solid Tumor |
| TCGA-XF-A9SJ-01A-11R-A39I-07 | 1 | NA | 401 | Primary solid Tumor |
| TCGA-XF-A9SK-01A-11R-A42T-07 | 1 | NA | 433 | Primary solid Tumor |
| TCGA-XF-A9SL-01A-11R-A39I-07 | 1 | NA | 401 | Primary solid Tumor |
| TCGA-XF-A9SM-01A-11R-A42T-07 | 2 | NA | 433 | Primary solid Tumor |
| TCGA-XF-A9SP-01A-11R-A39I-07 | 1 | NA | 401 | Primary solid Tumor |
| TCGA-XF-A9ST-01A-11R-A42T-07 | 1 | NA | 433 | Primary solid Tumor |
| TCGA-XF-A9SU-01A-31R-A39I-07 | 1 | NA | 401 | Primary solid Tumor |
| TCGA-XF-A9SV-01A-21R-A42T-07 | 1 | NA | 433 | Primary solid Tumor |
| TCGA-XF-A9SW-01A-11R-A42T-07 | 1 | NA | 433 | Primary solid Tumor |
| TCGA-XF-A9SX-01A-21R-A39I-07 | 2 | NA | 401 | Primary solid Tumor |
| TCGA-XF-A9SY-01A-21R-A42T-07 | 2 | NA | 433 | Primary solid Tumor |
| TCGA-XF-A9SZ-01A-11R-A39I-07 | 1 | NA | 401 | Primary solid Tumor |
| TCGA-XF-A9T0-01A-11R-A39I-07 | 1 | NA | 401 | Primary solid Tumor |
| TCGA-XF-A9T2-01A-11R-A42T-07 | 4 | NA | 433 | Primary solid Tumor |
| TCGA-XF-A9T3-01A-11R-A42T-07 | 2 | NA | 433 | Primary solid Tumor |
| TCGA-XF-A9T4-01A-11R-A39I-07 | 1 | NA | 401 | Primary solid Tumor |
| TCGA-XF-A9T5-01A-11R-A42T-07 | 2 | NA | 433 | Primary solid Tumor |
| TCGA-XF-A9T6-01A-11R-A42T-07 | 1 | NA | 433 | Primary solid Tumor |
| TCGA-XF-A9T8-01A-11R-A39I-07 | 2 | NA | 401 | Primary solid Tumor |
| TCGA-XF-AAME-01A-12R-A42T-07 | 5 | NA | 433 | Primary solid Tumor |
| TCGA-XF-AAMG-01A-11R-A42T-07 | 1 | NA | 433 | Primary solid Tumor |
| TCGA-XF-AAMH-01A-11R-A42T-07 | 4 | NA | 433 | Primary solid Tumor |
| TCGA-XF-AAMJ-01A-11R-A42T-07 | 1 | NA | 433 | Primary solid Tumor |
| TCGA-XF-AAML-01A-11R-A42T-07 | 1 | NA | 433 | Primary solid Tumor |
| TCGA-XF-AAMQ-01A-11R-A42T-07 | 1 | NA | 433 | Primary solid Tumor |
| TCGA-XF-AAMR-01A-31R-A42T-07 | 1 | NA | 433 | Primary solid Tumor |
| TCGA-XF-AAMT-01A-11R-A42T-07 | 2 | NA | 433 | Primary solid Tumor |
| TCGA-XF-AAMW-01A-11R-A42T-07 | 1 | NA | 433 | Primary solid Tumor |
| TCGA-XF-AAMX-01A-11R-A42T-07 | 1 | NA | 433 | Primary solid Tumor |
| TCGA-XF-AAMY-01A-11R-A42T-07 | 1 | NA | 433 | Primary solid Tumor |
| TCGA-XF-AAMZ-01A-11R-A42T-07 | 1 | NA | 433 | Primary solid Tumor |
| TCGA-XF-AAN0-01A-11R-A42T-07 | 1 | NA | 433 | Primary solid Tumor |
| TCGA-XF-AAN1-01A-31R-A42T-07 | 1 | NA | 433 | Primary solid Tumor |
| TCGA-XF-AAN2-01A-11R-A42T-07 | 1 | NA | 433 | Primary solid Tumor |
| TCGA-XF-AAN3-01A-11R-A42T-07 | 1 | NA | 433 | Primary solid Tumor |
| TCGA-XF-AAN4-01A-11R-A42T-07 | 5 | NA | 433 | Primary solid Tumor |
| TCGA-XF-AAN5-01A-11R-A42T-07 | 2 | NA | 433 | Primary solid Tumor |
| TCGA-XF-AAN7-01A-11R-A42T-07 | 3 | NA | 433 | Primary solid Tumor |
| TCGA-XF-AAN8-01A-11R-A42T-07 | 5 | NA | 433 | Primary solid Tumor |
| TCGA-XX-A899-01A-11R-A36F-07 | 9 | NA | 372 | Primary solid Tumor |
| TCGA-XX-A89A-01A-11R-A36F-07 | 9 | NA | 372 | Primary solid Tumor |
| TCGA-YC-A89H-01A-11R-A36F-07 | 1 | NA | 367 | Primary solid Tumor |
| TCGA-YC-A8S6-01A-31R-A38B-07 | 1 | NA | 391 | Primary solid Tumor |
| TCGA-YC-A9TC-01A-22R-A39I-07 | 8 | NA | 401 | Primary solid Tumor |
| TCGA-YF-AA3L-01A-11R-A38B-07 | 1 | NA | 391 | Primary solid Tumor |
| TCGA-YF-AA3M-01A-11R-A42T-07 | 1 | NA | 433 | Primary solid Tumor |
| TCGA-Z7-A8R5-01A-42R-A41B-07 | 9 | NA | 379 | Primary solid Tumor |
| TCGA-Z7-A8R6-01A-11R-A41B-07 | 9 | NA | 379 | Primary solid Tumor |
| TCGA-ZF-A9R0-01A-11R-A38B-07 | 1 | NA | 391 | Primary solid Tumor |
| TCGA-ZF-A9R1-01A-11R-A39I-07 | 1 | NA | 401 | Primary solid Tumor |
| TCGA-ZF-A9R2-01A-11R-A39I-07 | 1 | NA | 401 | Primary solid Tumor |
| TCGA-ZF-A9R3-01A-11R-A38B-07 | 1 | NA | 391 | Primary solid Tumor |
| TCGA-ZF-A9R4-01A-11R-A38B-07 | 1 | NA | 391 | Primary solid Tumor |
| TCGA-ZF-A9R5-01A-12R-A42T-07 | 1 | NA | 433 | Primary solid Tumor |
| TCGA-ZF-A9R7-01A-11R-A38B-07 | 1 | NA | 391 | Primary solid Tumor |
| TCGA-ZF-A9R9-01A-11R-A38B-07 | 1 | NA | 391 | Primary solid Tumor |
| TCGA-ZF-A9RC-01A-11R-A38B-07 | 1 | NA | 391 | Primary solid Tumor |
| TCGA-ZF-A9RD-01A-11R-A42T-07 | 2 | NA | 433 | Primary solid Tumor |
| TCGA-ZF-A9RE-01A-11R-A38B-07 | 1 | NA | 391 | Primary solid Tumor |
| TCGA-ZF-A9RF-01A-11R-A38B-07 | 3 | NA | 391 | Primary solid Tumor |
| TCGA-ZF-A9RL-01A-11R-A38B-07 | 1 | NA | 391 | Primary solid Tumor |
| TCGA-ZF-A9RM-01A-11R-A38B-07 | 1 | NA | 391 | Primary solid Tumor |
| TCGA-ZF-A9RN-01A-11R-A42T-07 | 2 | NA | 433 | Primary solid Tumor |
| TCGA-ZF-AA4N-01A-11R-A38B-07 | 2 | NA | 391 | Primary solid Tumor |
| TCGA-ZF-AA4R-01A-11R-A38B-07 | 1 | NA | 391 | Primary solid Tumor |
| TCGA-ZF-AA4T-01A-11R-A38B-07 | 1 | NA | 391 | Primary solid Tumor |
| TCGA-ZF-AA4U-01A-11R-A38B-07 | 1 | NA | 391 | Primary solid Tumor |
| TCGA-ZF-AA4V-01A-11R-A38B-07 | 1 | NA | 391 | Primary solid Tumor |
| TCGA-ZF-AA4W-01A-12R-A38B-07 | 2 | NA | 391 | Primary solid Tumor |
| TCGA-ZF-AA4X-01A-11R-A38B-07 | 1 | NA | 391 | Primary solid Tumor |
| TCGA-ZF-AA51-01A-21R-A39I-07 | 1 | NA | 401 | Primary solid Tumor |
| TCGA-ZF-AA52-01A-12R-A39I-07 | 1 | NA | 401 | Primary solid Tumor |
| TCGA-ZF-AA53-01A-11R-A39I-07 | 2 | NA | 401 | Primary solid Tumor |
| TCGA-ZF-AA54-01A-11R-A39I-07 | 3 | NA | 401 | Primary solid Tumor |
| TCGA-ZF-AA56-01A-31R-A39I-07 | 1 | NA | 401 | Primary solid Tumor |
| TCGA-ZF-AA58-01A-12R-A42T-07 | 2 | NA | 433 | Primary solid Tumor |
| TCGA-ZF-AA5H-01A-11R-A39I-07 | 2 | NA | 401 | Primary solid Tumor |
| TCGA-ZF-AA5N-01A-11R-A42T-07 | 1 | NA | 433 | Primary solid Tumor |
| TCGA-ZF-AA5P-01A-11R-A39I-07 | 1 | NA | 401 | Primary solid Tumor |

NA=not available
